# Genetic Stratigraphy of Key Demographic Events in Arabia

**DOI:** 10.1371/journal.pone.0118625

**Published:** 2015-03-04

**Authors:** Verónica Fernandes, Petr Triska, Joana B. Pereira, Farida Alshamali, Teresa Rito, Alison Machado, Zuzana Fajkošová, Bruno Cavadas, Viktor Černý, Pedro Soares, Martin B. Richards, Luísa Pereira

**Affiliations:** 1 Instituto de Investigação e Inovação em Saúde, Universidade do Porto, Porto, Portugal; 2 Instituto de Patologia e Imunologia Molecular da Universidade do Porto (IPATIMUP), Porto, Portugal; 3 School of Biology, Faculty of Biological Sciences, University of Leeds, Leeds, United Kingdom; 4 Instituto de Ciências Biomédicas da Universidade do Porto (ICBAS), Porto, Portugal; 5 General Department of Forensic Sciences and Criminology, Dubai Police General Headquarters, Dubai, United Arab Emirates; 6 Archaeogenetics Laboratory, Institute of Archaeology of the Academy of Sciences of the Czech Republic, Prague, Czech Republic; 7 Department of Biological Sciences, School of Applied Sciences, University of Huddersfield, Huddersfield, United Kingdom; 8 Faculdade de Medicina da Universidade do Porto, Porto, Portugal; Estonian Biocentre, ESTONIA

## Abstract

At the crossroads between Africa and Eurasia, Arabia is necessarily a melting pot, its peoples enriched by successive gene flow over the generations. Estimating the timing and impact of these multiple migrations are important steps in reconstructing the key demographic events in the human history. However, current methods based on genome-wide information identify admixture events inefficiently, tending to estimate only the more recent ages, as here in the case of admixture events across the Red Sea (∼8–37 generations for African input into Arabia, and 30–90 generations for “back-to-Africa” migrations). An mtDNA-based founder analysis, corroborated by detailed analysis of the whole-mtDNA genome, affords an alternative means by which to identify, date and quantify multiple migration events at greater time depths, across the full range of modern human history, albeit for the maternal line of descent only. In Arabia, this approach enables us to infer several major pulses of dispersal between the Near East and Arabia, most likely via the Gulf corridor. Although some relict lineages survive in Arabia from the time of the out-of-Africa dispersal, 60 ka, the major episodes in the peopling of the Peninsula took place from north to south in the Late Glacial and, to a lesser extent, the immediate post-glacial/Neolithic. Exchanges across the Red Sea were mainly due to the Arab slave trade and maritime dominance (from ∼2.5 ka to very recent times), but had already begun by the early Holocene, fuelled by the establishment of maritime networks since ∼8 ka. The main “back-to-Africa” migrations, again undetected by genome-wide dating analyses, occurred in the Late Glacial period for introductions into eastern Africa, whilst the Neolithic was more significant for migrations towards North Africa.

## Introduction

The issue of admixture in human populations is normally addressed by genome-wide (GW) studies, and several approaches have been developed to date admixture events [1,2,3,4,5]. Admixed populations bear chromosomes with segments of DNA from all contributing source groups, the size of which decreases over successive generations until recombination renders them undetectably short. Several algorithms attempt to date admixture events by inferring the size of the nuclear ancestry segments, and these can work well when dating recent episodes in human history, such as the sub-Saharan African input into the New World [6], but they fail to detect several known episodes that took place at earlier times, such as the African input into Iberia [1] and genetic exchanges across the Red Sea [7]. Simulations with the suite of methods available at the ADMIXTOOLS package indicated that these methods could detect admixture events as early as 500 generation ago, but real data did not allow the tracing of such old events [8]. A recent improved algorithm, called GLOBETROTTER, has been used to tackle the detection of the co-occurrence of several mixture events by decomposing each chromosome into a series of haplotypic chunks and then analysing each chunk independently [3], but the problem of detecting ancient events remains. Its application to the systematic screening of worldwide admixture events was able to reveal around 100 events, but all occurring over only the past 4,000 years [3].

The uniparental markers, characterised by the absence of recombination, do make possible the inference of ancestry for the mitochondrial genome and non-recombining, male-specific portion of the Y chromosome (mtDNA and MSY, respectively), and the dating of some demographic events (those which leave a signature in the genealogy), provided that a mutation rate of these molecules is reliably established. For the mtDNA, in the last couple of years, the application of various methods has led to quite reliable mutation rates with which to convert genetic diversities into time [9,10], while the MSY remains prone to more uncertainty [11], although promising advances are being achieved with whole Y chromosomal mutation rate calibrations [12,13,14].

At the same time, it is important to emphasize that the age of an mtDNA haplogroup cannot be directly associated with a migration event, as the diversity that has arisen in the source population, predating the migration event, would be included in the measurement. Founder analysis is an attempt to overcome this limitation. This approach picks out founder sequence types in potential source populations and dates lineage clusters deriving from them in the settlement zone of interest. In a way, the founder analysis allows us to reconstruct the stratigraphy of the migration events responsible for making up a population genetic pool, analogous to the archaeological reconstruction of the history of a site by the analysis of its sequential layers [15,16,17,18].

Some authors have been critical of dating migration events solely based upon the mtDNA evidence, arguing that maternal lineages do not necessarily represent the entire population, and are especially sensitive to drift [19]. Nevertheless, mtDNA-based conclusions for many migrations across various regions of the globe have been subsequently supported by genome-wide results [20,21], despite the limitations of the latter in dating events. In fact, the genealogical approach taken for mtDNA may overcome the effects of drift more effectively than the use of genome-wide SNPs, as we recently demonstrated in the highly-drifted Ashkenazi population: the fine characterisation of mtDNA sequences provided a detailed reconstruction of the maternal Ashkenazi pool, indicating that at least 80% of the lineages had a deep European ancestry [22], an influence not so readily identified in worldwide PCAs based on genome-wide data [23]. Thus, we suggest that for high time-depths, the mtDNA remains at present the most informative genetic system with which to infer past migrations and estimate their time frames, allowing us to disentangle the palimpsest that results from the impact of successive migrations.

Several distinct disciplines, including climatology, archaeology and genetics, are beginning to suggest that Arabia featured a highly dynamic genetic pool over time, since its successful settlement at ∼60 thousand years ago (ka) during the out-of-Africa dispersal [16,24]. The Arabian Peninsula was exposed to several climate change episodes, with fluctuations between arid (leading to population contraction) and humid (population expansion) phases, which conditioned its role as a bridge connecting Africa with Eurasia [25,26].This bridge may have been limited, over long periods or in climatically unfavourable times, to three refuge areas: the Red Sea coastal plain; the Dhofar and Mahra Mountains and adjacent littoral zone in Yemen and Oman; and the emerged floodplain within the Persian Gulf basin [27]. In particular, the latter “Gulf Oasis” may have been fundamental for the survival during arid conditions of the ancient N(xR) mtDNA lineages coalescing at ∼60 ka found in Arabia [24], most likely the relicts of the first migrants; the Gulf was also a preferential contact bridge with the Fertile Crescent.

Since these relict lineages are very minor, however, this signal for the settlement of Arabia during the successful out-of-Africa migration does not clarify if it was a continuous process lasting to the present day. The Pleistocene to Holocene continuity *versus* discontinuity debate has centred on how far the Arabian population was made up from the producers of the Levantine Pre-Pottery Neolithic B (PPNB)-related industry [28]. After rather sparse Late Palaeolithic settlement, the archaeological evidence suggests a significant increase in sites throughout Arabia dating from 9–8 ka [29], but it remains unclear if these were the result of newly arrived people [30] or locals who adopted the new food-producing technology [31]. The scarcity of secure stratigraphic reconstructions in the archaeology of the Peninsula has contributed to the uncertainty in dating the major demographic events. We have shown that some of the most frequent South Arabian mtDNA lineages (such as R0a) display signs of introduction and expansion in the post-glacial period [32], thus pre-dating the Neolithic, although the global contribution of this period to the total Arabian maternal gene pool remains to be evaluated.

The archaeological evidence is clearer regarding the remarkable maritime trade system that Arabia established with Africa, the Near East and India in the ninth to eighth millennia, probably the earliest worldwide [33]. The maritime traffic was intensified in mid-sixth millennium, with the appearance of the Pre-Dynastic Egyptian period, which dominated long-distance trade in the Red Sea [34], while in the Persian Gulf trade was established between communities in present-day Bahrain, the Oman Peninsula, the Indus Valley and Gujarat [35].This trade contributed to commercial, cultural, linguistic and genetic exchanges. In terms of language expansion in the region, by applying a Bayesian approach to Semitic lexical data, Kitchen et al. [36] concluded for a single entrance of early Ethio-Semitic languages in Africa, from southern Arabia, at around 2800 years ago, a period when South Arabia was influential in northern Ethiopia. A well-documented movement of people occurred through the Arab slave trade established between the 6th and 19th centuries AD [37], bringing African people (from Nubia to Zanzibar) into the Near East, Arabian Peninsula and even India and China. Estimates indicate that 2,400,000 African people were enslaved along the Red Sea and Indian Ocean routes [38], with a 2:1 female to male ratio [39]. This has also been proposed to explain the high levels of African L(xMN) lineages observed in Yemen [37,40], but other potential sources for sub-Saharan African (but also Indian and Southeast Asian) mtDNA lineages in Arabia may be the result of Hadrami men spending several generations in diaspora around the Indian Ocean rim and returning to their homeland with women taken from the diaspora [41]. Kivisild et al. [37] also detected a 12% frequency of haplogroup L6 in their Yemeni population sample from Kuwait, which is only being marginally observed in Ethiopia and almost absent elsewhere in Africa, and hypothesised that L6 originated from the successful out-of-Africa migration at ∼60 ka. However, the subsequent characterisation of other Arabian populations, including Yemen and Oman [42,43,44,45,46,47], did not reproduce the high frequency of this mtDNA lineage in South Arabia.

In this work, we use mtDNA to provide a detailed stratigraphic characterisation of key demographic events in Arabia since the first successful out-of-Africa migration ∼60 ka. We performed mtDNA founder analysis for Arabia and neighbouring regions, aiming to ascertain and date the main dispersal episodes. The founder analysis was applied to the unbiased HVS-I database available for the region, and interpreted in the light of the more precise dating information gathered from whole-mtDNA sequences of informative haplogroups [24,32,47,48]. We also updated the phylogenetic trees of haplogroups J, T, L4 and L6, by performing 83 new whole-mtDNA sequences. We further tested our inferences from the HVS-I based founder analysis with a whole-mtDNA founder analysis using haplogroups J and T. The mtDNA information is put in perspective with results from genome-wide analyses of published data [3,23,49,50], focused for the first time on inferring the local Arabian population structure, which has been overlooked in the worldwide context of previous autosomal work.

## Results/Discussion

### Continuity of Pleistocene/Holocene settlement

Previous work has already provided genetic evidence for the exchange of lineages between the Near East and Arabia. This was confirmed with whole-mtDNA sequencing of the Eurasian macrohaplogroup N (including its branches X, I, W, N1a, N1b and some R lineages), which is dominant in Arabia, attaining a frequency of 66%–83% [24,32,47,48]. The obvious missing element in those studies was the whole-mtDNA sequencing of Arabian JT lineages, which we have performed here, providing a detailed phylogeographic analysis in Supplemental Material (outline topology in S1 and S2 Figs.; S1 Text). Following the pattern for the remaining N lineages, the frequency and diversity maps (S3, S4, S5, S7, S12, S13, S16 and S19 Figs.; S3 and S4 Tables) of JT lineages, displaying similarity across the Near East and Arabian Peninsula, as well as the many basal Arabian lineages (S8, S9, S10, S11, S14, S15, S17, S18, S20, S21, S22 and S23 Figs.), suggest that both regions were in close contact throughout the late Pleistocene and Holocene. Haplogroup J assumes a more important role in Arabia overall than haplogroup T, as testified by frequencies (between 7.7–20.6% and 3.2–10.2%, respectively) and the many star-like J sub-clades observed in Arabia, dating to ∼6–7 ka. These expansions in haplogroup J are reflected in the BSP analysis (S6 Fig.), for which the main increase in effective size was between 8–12 ka in Arabia (S6A Fig.), after the expansion observed in the Near East around 11–15 ka (S6B Fig.). Haplogroup J also shows signs of having crossed into eastern Africa, particularly the sub-clade J1d1a1, necessarily after its emergence in Arabia at ∼7.1 ka (S14 Fig.). Thus haplogroup JT indicates that demographic expansion in Southwest Asia was a continuous phenomenon from the Late Glacial period to the Neolithic period.

**Fig 1:**
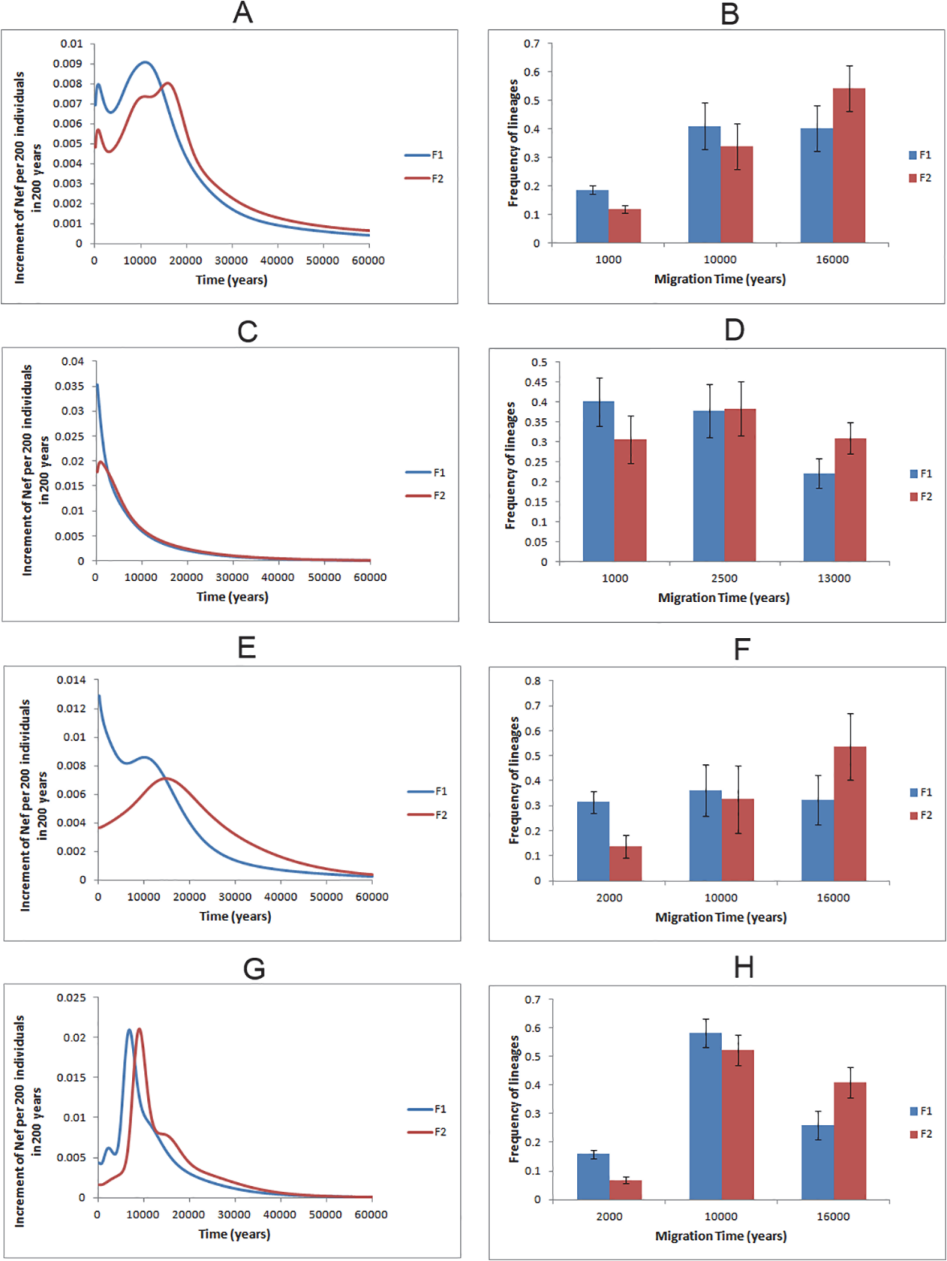
Founder analysis results. Probabilistic distribution of founder clusters across migration times, with time scanned at 200 year intervals from 0–60 ka, using *f1* (blue line) and *f2* criteria (red line), when considering putative migrations: (A) from the Near East, Iran and Pakistan to Arabia; (C) from Africa into Arabia plus the Near East and Iran; (E) Arabia plus the Near East and Iran into eastern Africa; (G) Arabia plus the Near East and Iran into North Africa; and probabilistic proportion of founder clusters considering different migration events, using *f1* (blue bar) and *f2* criteria (red bar), when considering putative migrations: (B) from the Near East, Iran and Pakistan to Arabia; (D) from African into Arabia plus the Near East and Iran; (F) Arabia plus the Near East and Iran into eastern Africa; (H) Arabia plus the Near East and Iran into North Africa.

In order to dissect the apparent continuous genetic exchange between Arabia and the Near East since the late Pleistocene, we performed a founder analysis for all Eurasian haplogroups assuming the Near East, Iran and Pakistan as source and Arabia as sink (identified founders reported in S6 and S7 Tables). Fig. 1A displays the overall pattern, which seems to favour the periods around 1ka, 10 ka and 16 ka for migrations. Based on this information, we further imposed these dates as migration events to represent broadly, respectively, recent events, the Younger Dryas/Neolithic transition and the Late Glacial period. The results indicate that the Late Glacial period (Fig. 1B) was the most important migratory period, responsible for the introduction of 40–54% of the lineages (mainly belonging to the haplogroups K, U2, U3, U4, N1a1a, N1a1b, H5 and HV1; S24 and S25 Figs. and detailed description in S1 Text). At the Younger Dryas/Neolithic boundary, 34–41% of lineages, mainly unclassified HV, R0a, J1b, T1a and M1 migrated to Arabia. The remaining 12–19% moved very recently, ∼1 ka, and consists of derived lineages, (including J1d1a, K1, HV8 and N1a3). Although it is hard to discriminate clearly between the Near Eastern and Pakistan/Iranian influences, due to their largely shared mtDNA pool, the results suggest a higher Pakistan/Iranian impact in the east (41%) than in the west (25%) of Arabia for private founders, but just 14% and 11%, respectively, when considering the overall pool. This seems to indicate that the Pakistan/Iranian contribution was recent, as the lineages introduced from this region did not reach high frequencies, and as expected its impact was higher in the eastern Arabian countries.

**Fig 2:**
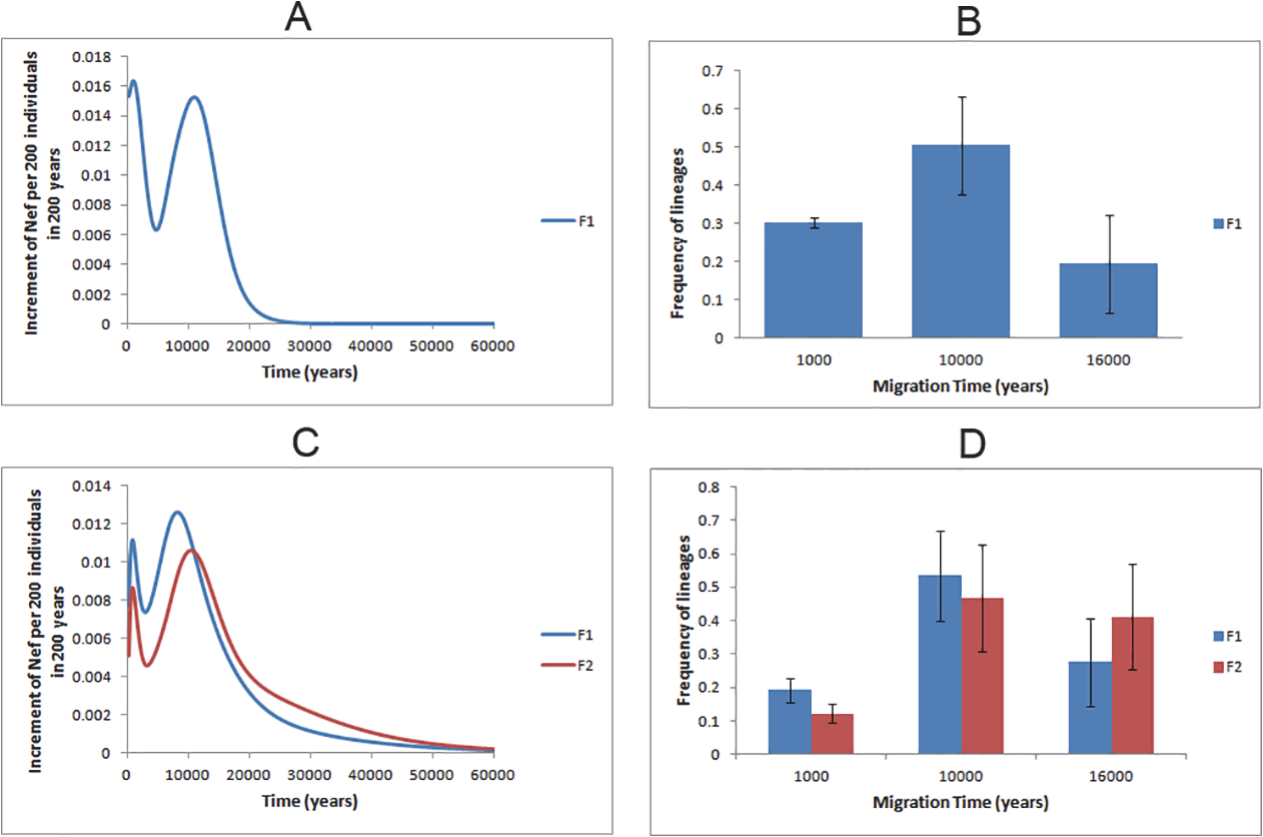
Founder analysis results on JT lineages. Probabilistic distribution of founder clusters across migration times, with time scanned at 200 year intervals from 0–60 ka, using *f1* (blue line) and *f2* criteria (red line), when considering putative migrations from the Near East, Iran and Pakistan to Arabia for (A) whole-mtDNA genomes or (C) HVS-I for haplogroups J and T; and probabilistic proportion of founder clusters considering different migration events, using *f1* (blue bar) and *f2* criteria (red bar), when considering putative migrations from the Near East, Iran and Pakistan to Arabia for (B) whole-mtDNA genomes or (D) HVS-I for haplogroups J and T.

We next tested the robustness of the founder analysis by using whole-mtDNA genomes and HVS-I from haplogroups J and T alone (Fig. 2). The 17 whole-mtDNA founders identified (S8 Table) contributed to the overall pattern of migration displayed in Fig. 2A, which displays two main peaks, at 1 ka and 10 ka. When imposing the model of three migrations (Fig. 2B), 30% of JT lineages were introduced at 1ka, 50% at 10ka and 20% at 16ka. These results match closely the inferences based only on HVS-I information (Fig. 2C,D).

We should emphasize that no one-to-one correspondence of founder types between whole-mtDNA genomes and HVS-I can be expected, as there is no such precise correspondence between the whole-mtDNA and HVS-I trees, due in part to the differences in resolution but also no doubt to the small samples size at present for the whole-mtDNAs. We must also beware that other factors may also confound the analysis in particular circumstances. An extreme—but very unusual—instance is haplogroup J1d1a. Here, the HVS-I based founder analysis dates the founders to 1.0 ka, while the whole-mtDNA analysis indicates that it expanded in Arabia at least 6.1 ka. This discrepancy is due to 18 HVS-I sequences belonging to the root haplotype largely from central Saudi Arabia, an artefact of the sampling location (central Saudi Arabia is extremely arid and has had historically very low population size, with habitation restricted to oases, undoubtedly leading to severe genetic drift), while the remaining more diverse samples are from Yemen (as for most of the whole-mtDNAs). If the Saudi samples are disregarded, a ρ estimate for the founder age in Arabia increases to ∼6–7 ka, fitting more closely with the whole-mtDNA result. Allowing for such inevitable noise effects from the datasets, the similarity between the whole-mtDNA and HVS-I analyses is indeed striking, and we conclude that it is reasonable to infer that the picture suggested by the whole-population HVS-I founder analysis is not giving a very misleading impression of the dispersal history of the region.

**Fig 3:**
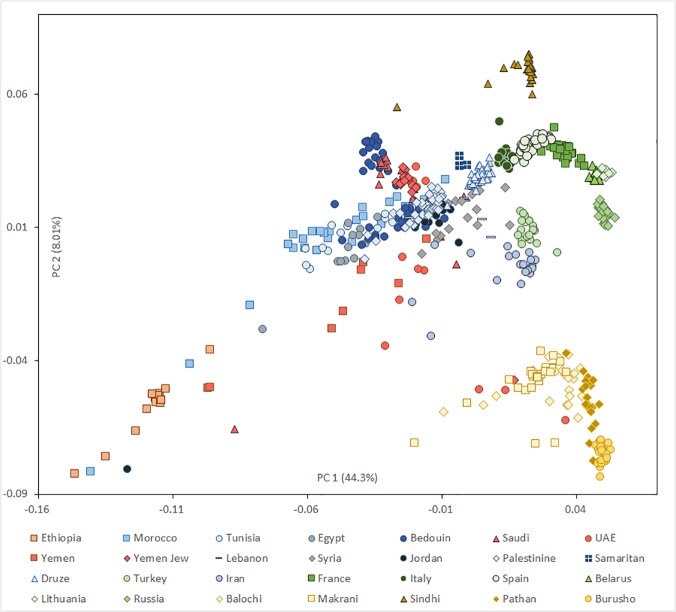
PCA results. Scatter plot of individuals, showing the first two principal components. Each symbol corresponds to one individual and the colour indicates the region of origin.

Although it is not possible to date securely events as old as the ones occurring in the Pleistocene/Holocene transition based on genome-wide data alone, it is interesting to observe how the patterns of shared genome-wide ancestry support the inferences made for the mtDNA. All the Arabian populations form a close group with Near East populations in PC analysis (Fig. 3), with the first component explaining 44% of the diversity and partitioning populations along a west–east axis, and the second component explaining 8% and organising populations on a north–south axis. A few individuals in Arabian populations most probably had recent ancestry within Africa (especially for Yemen) or Pakistan (in the United Arab Emirates; UAE). Yemen shows the highest dispersion along the first axis, testifying again the higher African input in the closest country to the Horn of Africa. We confirmed the clustering of Yemeni Jews with Bedouin and Saudi Arabians, already identified previously [23], and probably indicating that they were less open to recent admixture with non-Arabian populations than their Yemeni Arab/Muslims neighbours.

**Fig 4:**
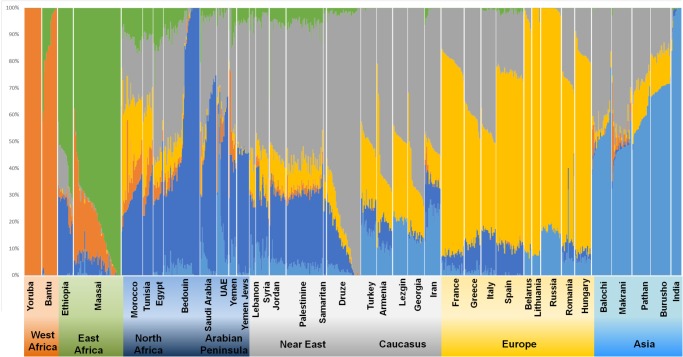
ADMIXTURE results. Population structure inferred by ADMIXTURE analysis. Each individual is represented by a vertical (100%) stacked column of genetic components proportions shown in colour for K = 6.

The ADMIXTURE results indicate that K = 6 (Fig. 4 and Table 1; other K plots are displayed in S38 Fig.) is the number of clusters that best represents the population structure of the analysed populations. Here it is already possible to distinguish between a Southwest Asian/Caucasian and an Arabian/North African component; these two components have similar proportions of ∼30% each in Yemen and UAE, but the Arabian/North African proportion increases to 52–60% in Saudi and Bedouin. In Near Eastern populations, correspondingly, the Southwest Asian/Caucasian component rises to ∼50% and the Arabian/North African cluster decreases to ∼20–30%, even in Palestinians (similar to the Samaritans and some of the Druze), highlighting their primarily indigenous origin, with the most extreme values for the Druze, carrying the Southwest Asian/Caucasian component at ∼80%.

**Table 1:** Estimates of admixture proportions (%) and date of admixture (in generations) calculated in ROLLOFF when using western (Yoruba) and eastern (Maasai) African and Italians + Spanish as ancestral populations.

Population	Sample Size	Western African ancestry proportion (%) ± standard error	Eastern African ancestry proportion (%) ± standard error	Southwest Asian/Caucasian ancestry proportion (%) ± standard error	Arabian/North African ancestry proportion (%) ± standard error	European ancestry proportion (%) ± standard error	South Asian ancestry proportion (%) ± standard error	Estimated date of admixture using ROLLOFF using Western African parental population	Estimated date of admixture using ROLLOFF using Eastern African parental population
Yemen	9*	16.935 ± 15.960	7.747 ± 5.333	30.777 ± 9.896	32.398 ± 6.030	3.217 ± 2.77	8.926 ± 3.727	21.019 ± 7.450	11.556 ± 3.878
Saudi Arabia	20	1.694 ± 5.223	4.033 ± 4.235	34.227 ± 8.955	52.479 ± 18.957	2.722 ± 3.879	4.844. ± 4.975	30.762 ± 4.907	25.430 ± 3.011
Yemen Jews	15	0.001 ± 0.000	5.105 ± 0.826	47.542 ± 1.525	45.693 ± 1.598	0.565 ± 0.699	1.094 ± 1.187	n/a	n/a
UAE	14	6.408 ± 9.118	1.817 ± 2.014	34.432 ± 4.312	34.378± 21.632	1.689 ± 1.931	21.276 ± 17.660	8.900 ± 1.642	8.923 ± 1.795
Bedouin	45	2.005 ± 2.213	4.692 ± 4.246	24.903 ± 19.909	60.057± 30.707	5.400 ± 4.700	2.944 ± 2.285	37.546 ± 3.104	27.734 ± 1.532
Lebanon	7	1.243 ± 4.854	4.670 ± 3.148	51.547 ± 2.519	21.092 ± 4.062	14.543 ± 2.791	6.905 ± 4.854	n/a	n/a
Syria	16	1.586 ± 1.451	3.413 ± 1.952	49.742 ± 4.880	23.260 ± 5.283	12.864 ± 4.532	9.135 ± 3.387	37.334 ± 4.365	26.181 ± 4.428
Jordan	20	3.205 ± 5.629	7.289 ± 6.404	47.833 ± 7.442	25.055 ± 3.209	11.171 ± 2.436	5.447 ± 2.169	32.871± 4.106	29.470 ± 3.671
Samaritan	3	0.001 ± 0.000	0.190 ± 0.777	63.029 ± 2.282	26.358 ± 2.709	8.946 ± 4.104	0.475 ± 0.496	n/a	n/a
Druze	42	0.178 ± 0.365	1.869 ± 1.082	80.100 ± 14.498	9.919 ± 7.730	6.123 ± 5.089	1.812 ± 1.664	n/a	n/a
Palestinian	46	2.222 ± 1.760	6.119 ± 2.147	51.538 ± 4.397	27.396 ± 2.153	9.153 ± 1.826	3.572 ± 1.302	29.008 ± 2.194	11.556 ± 3.878
Iran	20	1.701 ± 3.196	1.022 ± 1.818	50.678 ± 4.259	11.850 ± 5.614	11.135 ± 2.916	23.614 ± 3.944	n/a	n/a
Turkey	19	0.069 ± 0.029	0.194 ± 0.312	49.188 ± 3.258	8.993 ± 2.904	23.798 ± 3.503	17.758 ± 2.504	n/a	n/a
Ethiopia	19	3.911 ± 3.047	58.139 ± 8.479	12.146 ± 5.638	25.469 ± 5.495	0.179 ± 0.442	0.157 ± 0.297	93.223± 9.678	n/a
Maasai	19	15.808 ± 12.911	78.060 ± 15.009	0.412 ± 0.911	4.120 ± 3.043	0.096 ± 0.315	0.736 ± 1.858	47.007± 2.933	n/a
Egypt	12	5.553 ± 1.553	15.117 ± 4.878	39.826 ± 5.130	30.499 ± 6.343	8.380 ± 2.245	0.624 ± 0.630	30.034± 3.233	22.766 ± 2.890
Morocco	25	12.199 ± 10.473	12.066 ± 2.951	21.360 ± 4.827	28.872 ± 5.736	25.502 ± 7.971	0.001 ± 0.000	n/a	n/a
Tunisia	12	9.815 ± 2.927	10.437 ± 1.212	26.002 ± 4.057	30.991 + 6.178	22.754 ± 5.354	0.001 ± 0.000	n/a	n/a

N/A—not assigned.

* By eliminating one individual with a high level of African ancestry.

European background is higher in Near Eastern populations (around 9–15%) than in Arabia (1.5–5%) while the African ancestry is ∼25% in Yemen, and then 4–8% in all Arabian and Near East populations except in Samaritans and Druze, with 0–2%. The UAE has a substantial pool from South Asia (21%) similar to the proportion displayed in Iran (24%), which falls to below 10% in all other Arabian and Near Eastern populations, except Turkey (18%).

**Fig 5:**
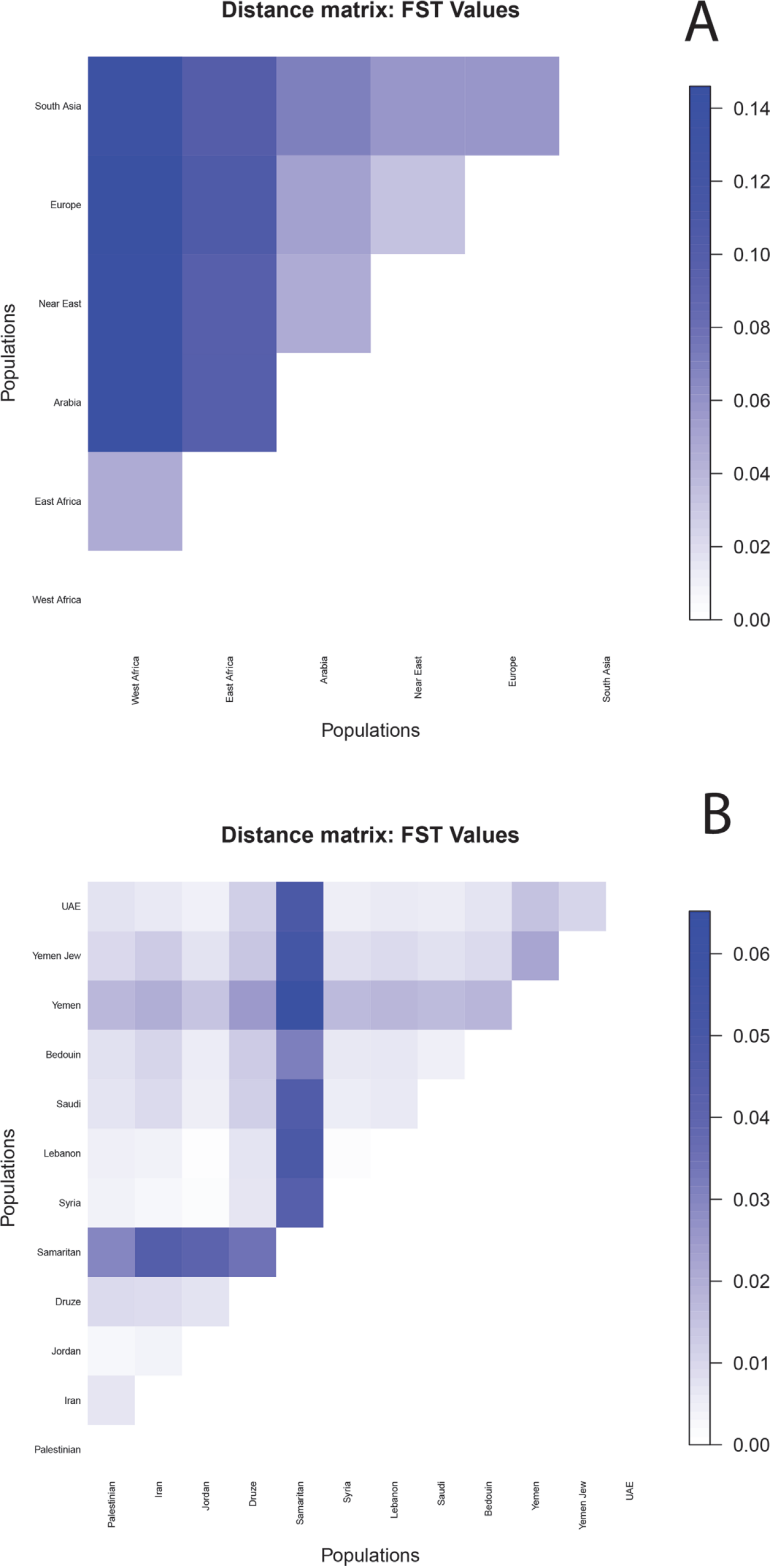
Matrices of *F*_*ST*_ distances. Matrices of *F*_*ST*_ values between ADMIXTURE components (A) and Arabian and Near Eastern populations (B).

ADMIXTURE allows us to calculate *F*_*ST*_ values between the components in order to quantify their similarity (Fig. 5A). For K = 6, Arabia showed a lower distance from the Near East (0.046), than from Europe (0.052), eastern Africa (0.098) and finally western Africa (0.140). Arabia and the Near East have similar genetic distances from eastern African (0.098 and 0.097, respectively), double that of the value between western and eastern Africa (0.046). When evaluating *F*_*ST*_ values in pairwise comparisons between Arabian and Near Eastern populations (Fig. 5B), we see that *F*_*ST*_ values are higher between Yemen and all other populations (and also for comparisons with Samaritans, but these results may be biased by low sample size). The UAE is closer to Jordan, Syria and Lebanon than Saudi Arabia is; while Saudi are closer to Palestinians, Druze and Samaritans than UAE. Thus, *F*_*ST*_ values support lower or similar genetic distances between UAE and Near Eastern populations as between Saudi and Near Eastern populations, while Yemen is clearly more divergent.

### Exchanges across the Red Sea—from Africa into Arabia

Founder analysis of the dispersal of sub-Saharan lineages from Africa into Arabia plus the Near East and Iran (both regions have to be considered together due to the relatively low number of L(xMN) sequences) showed a predominant migration peak at 0–0.8 ka (Fig. 1C). When checking these founders (S9 and S10 Tables), we see that most of them display clearly young ages, but several have ages ∼13 ka (S15 Table). So, we tested a model based on three periods of migration (Fig. 1D), and their impact was: 31–40% for 1 ka (middle of Arab slave trade, 6^th^–19^th^ centuries); 38% for 2.5 ka (Arabian dominance of the Red Sea trade routes); and 22–31% for 13ka (close to the Younger Dryas). As the great majority of lineages migrated in the two very recent putative events, at similar ages, this contributes to the dominant young peak in Fig. 1C, while the approximately one-third of sequences that were introduced later is responsible for the long tail of the curve (instead of a sharper peak). No clear pattern of association between haplogroup and event was observable, probably reflecting high levels of heterogeneity in the source (S32 and S33 Figs. and detailed description in S1 Text). Thus, the Arabian maritime dominance and slave trade (from very recently, back until∼2.5 ka) were the main contributors (∼69–78%) to the African ancestry into Arabia, Near East and Iran, but the entrance seems to have been initiated as early as the end of the Pleistocene. Clearly, no lineages could be assigned to the out-of-Africa migration event.

In order to provide more information to the issue of possible relicts of the out-of-Africa migration, we further investigated two relatively rare African haplogroups (L4 and L6), phylogenetically close to L3, by whole-mtDNA sequencing (outline topology in S26 Fig. and detailed topology in S28, S29 and S30 Figs.; S1 Text). L4 is more frequent nowadays in eastern Africa followed by the Near East (S27A Fig.; S5 Table). The whole-mtDNA-based date points to an origin at ∼87 ka, predating the out-of-Africa dispersal (as well as its sub-clade, L4b, dating to ∼86 ka). So, in theory, this sister haplogroup of L3 could have crossed into Arabia along with L3 during the initial out-of-Africa movement. Phylogenetically, however, the few Arabian L4 lineages are derived, supporting an explanation in more recent exchange networks between eastern Africa and Arabia for their dispersal, concordant with the recent signs of population growth detected for L4 in BSPs (S31A Fig.; and dominating also S31B Fig.; S14 Table). L6, at similarly low frequencies in Yemen and eastern Africa (S27B Fig.), dates to 23.1 [15.8–30.5] ka, and is likely to have migrated from eastern Africa into Arabia after that period, most probably very recently as testified by a very derived L6a sub-clade observed in three Yemenis (sharing the same lineage).

The genome-wide analyses performed here on the available data from Arabian populations provide estimates of African admixture, with disentanglement between western and eastern African gene pool contributions (Table 1). The eastern African background is around 4.0% in Saudi and Bedouin, ∼7.7% in Yemen (although Yemen Jews have a lower admixture of 5.1%), and 1.8% in UAE; this input decreases beyond Jordan, and is negligible in Samaritans, Druze, Turks and Iranians. The western African component also varies between 2.0 and 6.4%, except for Yemen (16.9%) where it has likely been inflated due to indirect recent migration (the Bantu component which is present in many eastern African populations). The ROLLOFF estimates for the event of admixture were 8–27 generations ago when using eastern Africa as parental population, and 8–37 generations using a western African source.

Both date estimates are compatible with the Arab slave trade, which operated between the 6th and 19th centuries AD, mainly from eastern Africa (from Nubia to Zanzibar), although many of these populations bear a significant western African component (as shown in Fig. 4). These values are in agreement with the estimates of Moorjani et al. [1] for Levantine groups, showing a 4–15% African ancestry and about 32 generations ago for the event of admixture, interpreted as consistent with close political, economic, and cultural links with Egypt in the late Middle Ages. They also estimated 72 generations ago for the event leading to 3–5% sub-Saharan ancestry in diverse Jewish populations, arguing that this reflecting descent of these groups from a common ancestral population that already had some African ancestry prior to the Jewish Diaspora.

Hodgson et al. [7] focused on the back-to-Africa migration in the Horn of Africa, and obtained ages from 2.2–4.7 ka for the admixture event when using the ROLLOFF and ALDER methods. The authors relied on other approaches in order to evaluate the hypothesis of two or more distinct episodes of non-African admixture in the Horn of Africa: they identified a non-African Ethio-Somali component in eastern African populations in the ADMIXTURE analysis for which *F*_*ST*_-based dating methods indicated an age of divergence from North African/Arabian populations of 23–25 ka, leading to a possible window of migration pre-LGM. These results fit well with the conclusions we reached in this study through the analysis of the maternal mtDNA pool.

### Exchanges across the Red Sea—from Arabia into Africa

The Bab-el-Mandab strait and the Red Sea were also important for dispersal in the opposite direction, the “back-to-Africa” migrations. Founder analysis (Fig. 1E; S11 and S12 Tables) led to the identification of peaks of migration at ∼10–15 ka. Given these results, we inferred two main migration events, at ∼10 ka (representing the Neolithic and beginning of maritime trade) and at ∼16ka (Late Glacial period), as well as an episode at ∼2 ka which could represent recent times (specifically, Arabian dominance of the Red Sea routes). The proportions (Fig. 1F) for migration contributed by these events were: 14–31% at ∼2 ka (for N1, R0a, T, J, K and X); 33–36% at ∼10ka (U6a1a, J1d1a, M1 and R0a); and 33%–54% at ∼16ka (M1 and HV1). A detailed analysis of these haplogroup distributions in the migration events is provided in S1 Text, S34 and S35 Figs.

Interpreting these results in the light of available whole-mtDNA sequences, only the introduction of N1 seems younger than expected, most probably due to lack of HVS-I resolution for this haplogroup. Two main founders (comprising haplogroups N1a and I) are at the root of N1 sub-clades (dating to 15.9 and 21.8 ka, respectively). Another founder in N1a could be placed in the sub-clade identified in the whole-mtDNA sequencing from Somalia reported by Fernandes et al. [24], bearing the substitution at position 16213; but the HVS-I data show that this is more frequent in Africa (seven individuals) than in Arabia (one individual), so this Arabian individual may be a recent introduction into Arabia of an N1a sub-clade that had evolved within Africa (dating to 0.9 ka [24]).

The phylogenetic analyses for N(xR) lineages performed by Fernandes et al. [24] also provided insights into back-to-Africa movements, evidently at various time periods. Some lineages (I, N1a and N1f) displayed deep branches in eastern Africa, a sign of introduction in Africa which could have begun as early as ∼40 ka (the upper bound defined by the TMRCA of the founder clades) and extending till ∼15 ka (the lower bound defined by the TMRCA of the derived African clades). The migration of J1d1a lineages into eastern Africa in the Neolithic period is confirmed in the whole-mtDNA sequencing (S14 Fig.) and complemented by the frequency interpolation and founder analysis (S13 Fig.) performed here.

From the genome-wide results, we can infer this back-to-Africa migration was considerable, leading to a proportion of 12% of Near Eastern and 26% Arabian ancestry in Ethiopia (Table 1). The ROLLOFF estimate for the date of admixture was 93 generations ago—twice as old as the time of African admixture in Arabia and Near East. For comparison, in the Maasai from Kenya and Tanzania, the Eurasian component is an order of magnitude lower (4.5%), and the time of admixture is 47 generations, reflecting most probably later admixture events.

The parallel introduction of Eurasian lineages from the Near East, Iran and Arabia into North Africa through the Sinai Peninsula revealed two well-defined peaks (Fig. 1G) at ∼2.4 ka and 6.8 ka with the *f1* criterion, and two peaks at ∼9.0 ka and ∼12.4 ka when using the *f2* criterion. This seems to point to a significant role for dispersal in the Neolithic period, consistent with results obtained for the North African MSY pool, interpreted as suggesting a large Neolithic origin [51]. A major Neolithic impact is supported when imposing periods for the migration of founders (Fig. 1H), leading to: 7–16% at ∼2 ka, mainly HV1 and other undefined HV lineages, M1 and U (U6a1, K1a1); 52–58% at ∼10 ka for most of HV, U (U5b, U5 and K), T (some T2c1 and T2b), J (J1d1a, J2a2b and other undefined J), and X; and 26%–41% at ∼16 ka for some HV, T (T1a, T2) and U (U3, U3a, U5b1b, U5a, U6a) lineages (S1 Text, S36 and S37 Figs.). It seems likely that some JT lineages, especially T ones, were introduced into Northeast Africa before the Neolithic, following Late Glacial population expansions in the Near East/Arabia. Then, locally they could have been involved in population expansions in the Neolithic period, leading to signs of autochthonous founder effects, such as the one detected in the El-Hayez oasis (400 km southwest of Cairo) for sub-haplogroup T1a2a [52].

The link between U6 and M1 and the settlement of North Africa from the Near East at ∼45 ka advanced previously [53,54] was recently put into question [55] because their sub-clades do not all seem to display the same history: U6a is ∼10 ka older than M1a and M1b, and sub-clades of the former coalesce before or around the LGM while sub-clades of the latter date to the post-LGM. In our founder analysis for North Africa, a strong Late Glacial signal was detected for U6.

At the genome-wide level, Egypt is quite similar to its Levantine neighbours, displaying a mainly Near Eastern (39.8%) and Arabian/North African (30.5%) background, with slightly higher western (5.6%) and eastern (15.1%) African proportions, and lower European (8.4%) and South Asian (0.6%) proportions. The ROLLOFF estimate for admixture in Egypt (using Africans and Europeans as ancestral populations) was 30 generations, predictably young due to continuous gene flow between the two regions. Morocco and Tunisia presented similar western (9.8–12.2%) and eastern African (10.4–12.1%) components and roughly twice the magnitude for each of the European (22.8–25.5%), Near Eastern (21.4–26.0%) and Arabian (28.9–31.0%) pools. Again these young dates show that simple genome-wide dating approaches based on linkage disequilibrium decay must be applied cautiously in complex scenarios of several migrations occurring over a long span of time, such as the ones which took place across the Red Sea, North Africa [56] and Iberia [57].

### Conclusions

The detailed evaluation of the Arabian and neighbouring mtDNA pools has allowed us to establish a genetic stratigraphy of Arabia’s maternal line of descent, testifying to the pivotal role of the Peninsula at the crossroads between Africa and Eurasia. The successful out-of-Africa migration led to continuous settlement of parts of the Peninsula, most probably centred on the Gulf Oasis, which likely functioned as the cradle for the emergence of the haplogroup N lineages. No haplogroup L(xMN) relicts of this migration into Arabia are detected in mtDNA founder analysis and we have confirmed their absence by whole-mtDNA sequencing of lineages from L3 [16] and its sister clades L4 and L6.

Although it is likely that the Gulf Oasis region eventually formed part of an extended source region together with the Near East, if we assume that the Near East was the main source population for current Arabian diversity, the Late Glacial period was responsible for the introduction of 40–54% of lineages, the Younger Dryas/Neolithic for 34–41%, and recent times (at 1.0 ka) for the remaining 12–19%. The Neolithic in Arabia was more characterised by the expansion in effective size of local haplogroup N lineages, mostly within R0a and J, than by the entrance of new lineages. Arabia, together with the Near East and Iran, was involved in the “back-to-Africa” migration of Eurasian lineages, beginning in the Pleistocene but becoming more significant with the establishment of maritime commercial routes. The Late Glacial period was more important for bringing Eurasian lineages into eastern Africa, probably reflecting the higher impact of this period in the expansion of Arabian populations, while the Neolithic, especially linked to the Near East, affected to a greater extent the dispersals towards North Africa. The biparental genome averaged the African input to 6–25% of the Arabian pool, concordant with the 35% female and 0% male inputs estimated from uniparental systems. ROLLOFF dating of admixture events across the Red Sea suggested recent ages of 8–37 generations for the African input into Arabia, 93 generations for the Arabian/Near Eastern input into eastern Africa and 30 generations for North Africa.

We conclude by emphasising that different parts of the genome of an admixed population often tell different stories—so the strategy must involve independent evaluation of (large) linked blocks. This is precisely what we do when analysing the diverse mtDNA lineages found in a population, but because mtDNA is a single linked locus, the different stories then emerge from the different lineages, carried by different individuals within a population. Probably, regions of the nuclear genome with a low recombination rate will allow estimation of older events, as soon as more complete nuclear genomes are available from more populations, overcoming the limits of molecular resolution of current genome-wide SNPs.

## Materials and Methods

### Samples for whole-mtDNA sequencing and statistical comparisons

We previously characterised the mtDNA diversity in populations from eastern Africa [16], the Arabian Peninsula [42,46,47], and the African Sahel [58], by sequencing the hypervariable segments I and in some cases II (HVS-I and HVS-II) using a procedure described previously [59]. This information was used to assign mtDNA sequences to haplogroups, following the most up-to-date phylogenetic evidence, reported on the PhyloTree website [60],checking the classification against the output of the Haplogrep software [61]. We then selected 26 UAE and 31 Yemen samples belonging to haplogroups J and T, and some belonging to haplogroups L4 and L6 for whole-mtDNA sequencing, amounting into a total of 26 (L4: 1 Burkina Faso, 2 Chad, 2 Dubai, 4 Ethiopia, 2 Kenya, 1 Niger, 1 Nigeria, 1 Nubia, 5 Somalia and Sudan; L6: 2 Ethiopia, 1 Kenya and 2 Somalia) (S1 Table).

We followed the methodology and quality control procedures of Pereira et al. [62], and mutations were scored relative to the revised Cambridge reference sequence [63]. The sequences obtained are reported in S1 Table and have been deposited in GenBank (accession numbers KP316996-KP317078).

For the whole-mtDNA analyses (S1 and S2 Tables), we used a total of 1779 samples of JT whole-mtDNA sequences (57 new, 1722 published) and 57 L4/L6 sequences (26 new, 31 published) in the reconstruction of their phylogenetic trees. We constructed a database of HVS-I and HVS-II sets from African, Arabian, European, Near Eastern, Iranian and Pakistani populations, amounting to 42,485 sequences, for founder analysis; these data are summarised in S6, S7, S8, S9, S10, S11 and S12 Tables. By the Arabian Peninsula, we assumed the territory covered by present-day Oman, UAE (which together we sometimes identified as eastern Arabia), Saudi Arabia and Yemen (western Arabia) countries. In the Near East, we included Iraq, Jordan, Israel/Palestine, Turkey, Lebanon and Syria.

This study obtained ethical approval from the Ethics Committee of the University of Porto, Portugal (11/CEUP/2011). Written informed consent was obtained from all sampled individuals, except from illiterate people who provided oral consent and a fingerprint instead of signature. The Ethics Committee approved this procedure.

### Statistical analyses of mtDNA data

For the phylogenetic reconstructions, preliminary reduced-median network analyses [64] led to a suggested branching order for the trees, which we then constructed most parsimoniously by hand. We used the mtDNA-GeneSyn software [65] to convert files.

In order to estimate the time to the most recent common ancestor (TMRCA) for specific clades in the phylogeny, we used the ρ statistic [18] and maximum likelihood (ML). We used ρ (the mean sequence divergence from the inferred ancestral haplotype of the clade in question) with a mutation rate estimate for the whole-mtDNA sequence of one substitution in every 3624 years, correcting for purifying selection, and a synonymous mutation rate of one substitution in every 7884 years [66]. Standard errors were estimated as before [67]. We obtained the ML estimates of branch lengths using PAML 3.13 [68], assuming the HKY85 mutation model with gamma-distributed rates (approximated by a discrete distribution with 32 categories). We converted mutational distance in ML to time using the same whole-mtDNA genome clock.

In order to investigate the population demography associated with the different haplogroups analyzed (J/T and L4/L6), we obtained Bayesian skyline plots (BSPs) [69] from BEAST 1.4.6 [70] for a total of 1720 and 57 (J/T and L4/L6, respectively) whole-mtDNA sequences with a relaxed molecular clock (lognormal in distribution across branches and uncorrelated between them) and the HKY model of nucleotide substitutions with gamma-distributed rates (10 gamma categories). BSPs estimate the effective population size through time using random sequences from a given population, but have also proved effective with individual haplogroups data [71]. For this analysis, we used a mutation rate of 2.6129 x10^−5^, previously calibrated using internal calibration points within the L3 phylogeny [16]. BEAST uses a Markov-chain Monte-Carlo (MCMC) approach to sample from the posterior distributions of model parameters (branching times in the tree and substitution rates). Specifically, we ran 100,000,000 iterations, with samples drawn every 10,000 MCMC steps, after a discarded burn-in of 10,000,000 steps. We checked for convergence to the stationary distribution and sufficient sampling by inspection of posterior samples. We visualized the Bayesian skyline plots (BSPs) with Tracer v1.3 [69]. We used a generation time of 25 years and forced the larger haplogroups to be monophyletic in the analysis: MCMC updates which violated this assumption were rejected. In order to perform a systematic comparison and description of the increment periods in the effective population size of the BSP, we calculated a rate of population size change through time.

To visualize the geographical distribution of haplogroups J, T, L4 and L6, we constructed interpolation maps using the “Spatial Analyst Extension” of ArcView version 3.2 (www.esri.com/software/arcview/). We used the “Inverse Distance Weighted” (IDW) option with a power of two for the interpolation of the surface. IDW assumes that each input point has a local influence that decreases with distance. The geographic location used is the centre of the distribution area from which the individual samples of each population were collected. The data used are listed in S3, S4 and S5 Tables.

In order to estimate the times of migrations into and from the Arabian Peninsula, we employed founder analysis [15]. This method assumes a strict division between assumed source and sink populations and two criteria (*f1* and *f2*) for identifying founder sequences to partly allow for homoplasy and back migrations, by ensuring that sequence matches are not at the tips of the source phylogeny. Founders must have at least one (*f1*) or two (*f2*) derived branches in the source population. The first step is to reconstruct, haplogroup by haplogroup, the HVS-I networks in the range 16051–16400 bp of the reference sequence [63]; we then identified founders and descendants using an in-house computer tool [72]; and finally we estimated the age of the migration of each founder using the ρ statistic [18], assuming an HVS-I mutation rate of one mutation every 16,677 years [66].

Four paths of migration were tested: (1) from Africa into Arabia plus the Near East and Iran (identified through the L(xMN) haplogroups); (2) from the Near East, Iran and Pakistan into the Arabian Peninsula (N haplogroups); (3) from Arabia plus Near East and Iran into eastern Africa (N and M1 haplogroups); and (4) from Arabia plus Near East and Iran into North Africa (N and M1 haplogroups). We included Pakistan in path (2) as we were also interested in inferring the more eastern contribution into the Arabian Peninsula. In order to assess the error in the Bayesian partitioning across the different migration times realistically, we calculated an effective number of samples for each founder cluster. This was obtained by multiplying the number of samples for each founder cluster by a ratio of the variance assuming a star-like network and the variance calculated as in Saillard et al. [67].

We scanned the distribution of founder ages for each region, defining equally spaced 200-year intervals for each migration from 0–70 ka. For each case, we also investigated the proportion of introduction of lineages during putative migrations occurring in certain periods of time. We selected these migration events by combining three distinct lines of evidence: the peaks detected in the founder analysis; historical/archaeological evidence; and dates from whole-mtDNA sequences belonging to informative haplogroups in the region (such as R0a, JT, N1, N2, I, L3 and L4/L6). We represented the probabilistic proportions of introduction for each lineage at each of the putative migration periods in graphs resembling the images from the STRUCTURE analysis.

In order to further validate the HVS-I founder analysis into Arabia we compared it with the results obtained from a founder analysis using whole-mtDNA genomes belonging to haplogroups J and T. We only used an *f1* criterion (since the sampling from the source was too scarce to allow an *f2* criterion) and we detected 17 founders (S8 Table). The assumptions of the founder method do not allow the use of a time-dependent clock. Therefore, given the relatively small difference between the mutation rate for time zero (average 2562 years for a mutation to happen) and the mutation rate for the oldest founder (average 2667 years for a mutation to happen) we used the intermediate value (2614 years for a mutation to happen) as an estimate for the overall range. As with the HVS-I founder analysis, we performed a preliminary scan analysis and estimated relative contributions of JT lineages in a three-migration model.

### Genome-wide database

We assembled genome-wide data for 790 samples from eight geographic groups (sub-Saharan Africa, North Africa, Arabian Peninsula, Near East, Iran, Europe, Caucasus and South Asia) from previously published data sets (S13 Table). The samples from Behar et al. [23] were genotyped using Illumina the 610K and 660K bead arrays, while those from Li et al. [49] were screened with Illumina 650K bead arrays, and those from Hellenthal et al. [3] with Illumina 660K bead arrays. We obtained the genotypes from Maasai, an ethnic group located in Kenya, from the HapMap phase III release (http://hapmap.ncbi.nlm.nih.gov/). We used PLINK 1.05 [73] to check that individuals and SNPs had a genotyping success of 97%. We used a Python in-house script to merge genotypes from the various chips and ended up with a total of 309,474 common autosomal single nucleotide polymorphisms (SNPs). We pruned the full dataset for linkage disequilibrium (LD), removing SNPs in strong LD (*r* ^2^ > 0.4) with nearby markers in a window of 50 SNPs (advanced by 10 SNPs each time); a total of 215,286 SNPs remained for further analyses.

### Genome-wide statistical analyses

We analysed the 790 samples with the ADMIXTURE software [74] which provides a maximum likelihood estimation of the population structure. We tested several numbers of clusters or ancestral populations, K (from three to six), with termination criteria for independent runs for each K value established when the log-likelihood increased by less than 10^−4^ between iterations. We performed across-validation to check the K value with the lowest cross-validation error, which would represent the most accurate modelling choice.

We carried out the principal component (PC) analysis, which infers worldwide axes of human genetic variation from the allele frequencies of various populations, using the *smartpca* tool, available in the EIGENSOFT package [75]. We evaluated the statistical significance of each PC through the Tracy-Widom statistics, computed at the EIGENSOFT tool *twstats*. As we were focused in Arabia, we did not include all populations in the analysis, especially the western African ones, in order to maximise the resolution.

To estimate the ages of putative admixture events in populations displaying statistical evidence of admixture, we used the ROLLOFF method [1] implemented in the ADMIXTOOLS software package [8]. This method is based on the decay of admixture LD in the target population, performing a local ancestry inference. We ran the ROLLOFF method for Arabia and some Near Eastern populations, using the unpruned set, with Maasai individuals (from the HapMap dataset, selected after the ADMIXTURE analysis, as the ones displaying >80% eastern African ancestry) and Italy plus Spain (extracted from 1000 Genomes database; http://browser.1000genomes.org/index.html) as ancestral populations. We also performed this analysis by replacing Maasai by Yoruba, from western Africa, to check for the influence of the selected African ancestral population, and as some eastern African populations also have a high western African component (such as Luhya in Webuye, Kenya, in the 1000 Genomes database).

We plotted the correlation between SNPs as a function of genetic distance for all chromosomes. Ages (in number of generations) were estimated by fitting an exponential distribution to the decay of these correlation coefficients. The estimated age (in number of generations) for the admixture event is the average of dates for all chromosomes. The *F*_*ST*_ values between pairs of ADMIXTURE components (K = 6) were estimated using ADMIXTURE, while the ones between pairs of populations were performed using vcf tools (http://vcftools.sourceforge.net/).

## Supporting Information

S1 Fig Schematic tree of haplogroup J. Ages (in ka) indicated are maximum likelihood estimates obtained for the whole-mtDNA genome.
(TIF)

S2 Fig Schematic tree of haplogroup T. Ages (in ka) indicated are maximum likelihood estimates obtained for the whole-mtDNA genome.
(TIF)

S3 Fig Frequency maps based on HVS-I data for haplogroups J (A) and T (B). (TIF)

S4 Fig Distribution maps for haplogroup J for the diversity measures π (A) and ρ (B) based on HVS-I data. (TIF)

S5 Fig Distribution maps for haplogroup T for the diversity measures π (A) and ρ (B) based on HVS-I data. (TIF)

S6 Fig Bayesian skyline plot indicating hypothetical effective population size through time based on data from haplogroup J of Arabia (A) and Near East (B) and from haplogroup T of Arabia (C) and Near East (D). (TIF)

S7 Fig Frequency maps based on HVS-I data for haplogroups J1b. (TIF)

S8 Fig Phylogenetic tree of haplogroup J1b. Labels on the branches represent nucleotide positions of transitions, and transversions when followed by a suffix “A,” “G,” “C,” or “T”; insertions are indicated by a dot followed by the number of repetitions and the nucleotide position; reversions by “!”; green indicates synonymous, brown non-synonymous, yellow other coding region, and black control region substitutions. Individual identification is indicated as well as the geographic origin when known (geographic regions are grouped by colour code according to the key). Near the nodes, the TMRCA is indicated (mean and 95% confidence interval) for ρ based on whole-mtDNA sequences (in black), ρ based on synonymous diversity (in green) and for maximum likelihood (in blue).
(TIF)

S9 Fig Phylogenetic tree of haplogroup J1b1. Labels on the branches represent nucleotide positions of transitions, and transversions when followed by a suffix “A,” “G,” “C,” or “T”; reversions by “!”; green indicates synonymous, brown non-synonymous, yellow other coding region, and black control region substitutions. Individual identification is indicated as well as the geographic origin when known (geographic regions are grouped by colour code according to the key). Near the nodes, the TMRCA is indicated (mean and 95% confidence interval) for ρ based on whole-mtDNA sequences (in black), ρ based on synonymous diversity (in green) and for maximum likelihood (in blue).
(TIF)

S10 Fig Phylogenetic tree of haplogroup J1b1a. Labels on the branches represent nucleotide positions of transitions, and transversions when followed by a suffix “A,” “G,” “C,” or “T”; reversions by “!”; green indicates synonymous, brown non-synonymous, yellow other coding region, and black control region substitutions. Individual identification is indicated as well as the geographic origin when known (geographic regions are grouped by colour code according to the key). Near the nodes, the TMRCA is indicated (mean and 95% confidence interval) for ρ based on whole-mtDNA sequences (in black), ρ based on synonymous diversity (in green) and for maximum likelihood (in blue).
(TIF)

S11 Fig Phylogenetic tree of haplogroup J1b2. Labels on the branches represent nucleotide positions of transitions, and transversions when followed by a suffix “A,” “G,” “C,” or “T”; reversions by “!”; green indicates synonymous, brown non-synonymous, yellow other coding region, and black control region substitutions. Individual identification is indicated as well as the geographic origin when known (geographic regions are grouped by colour code according to the key). Near the nodes, the TMRCA is indicated (mean and 95% confidence interval) for ρ based on whole-mtDNA sequences (in black), ρ based on synonymous diversity (in green) and for maximum likelihood (in blue).
(TIF)

S12 Fig Frequency maps based on HVS-I data for lineages within haplogroup J defined by the transition at 16193, which mainly corresponds to haplogroup J1d, but can also include haplogroup J2d. (TIF)

S13 Fig Frequency maps based on HVS-I data for the sub-haplogroup J1d1a. (TIF)

S14 Fig Phylogenetic tree of haplogroup J1d1. Labels on the branches represent nucleotide positions of transitions, and transversions when followed by a suffix “A,” “G,” “C,” or “T”; deletions are indicated “d”; reversions by “!”; green indicates synonymous, brown non-synonymous, yellow other coding region, and black control region substitutions. Individual identification is indicated as well as the geographic origin when known (geographic regions are grouped by colour code according to the key). Near the nodes, the TMRCA is indicated (mean and 95% confidence interval) for ρ based on whole-mtDNA sequences (in black), ρ based on synonymous diversity (in green) and for maximum likelihood (in blue).
(TIF)

S15 Fig Phylogenetic tree of haplogroup J1d2. Labels on the branches represent nucleotide positions of transitions, and transversions when followed by a suffix “A,” “G,” “C,” or “T”; insertions are indicated by a dot followed by the number of repetition and the nucleotide position; reversions by “!”; green indicates synonymous, brown non-synonymous, yellow other coding region, and black control region substitutions. Individual identification is indicated as well as the geographic origin when known (geographic regions are grouped by colour code according to the key). Near the nodes, the TMRCA is indicated (mean and 95% confidence interval) for ρ based on whole-mtDNA sequences (in black), ρ based on synonymous diversity (in green) and for maximum likelihood (in blue).
(TIF)

S16 Fig Frequency maps based on HVS-I data for haplogroup J2. (TIF)

S17 Fig Phylogenetic tree of haplogroup J2a2. Labels on the branches represent nucleotide positions of transitions, and transversions when followed by a suffix “A,” “G,” “C,” or “T”; reversions by “!”; green indicates synonymous, brown non-synonymous, yellow other coding region, and black control region substitutions. Individual identification is indicated as well as the geographic origin when known (geographic regions are grouped by colour code according to the key). Near the nodes, the TMRCA is indicated (mean and 95% confidence interval) for ρ based on whole-mtDNA sequences (in black), ρ based on synonymous diversity (in green) and for maximum likelihood (in blue).
(TIF)

S18 Fig Phylogenetic tree of haplogroup J2a2a. Labels on the branches represent nucleotide positions of transitions, and transversions when followed by a suffix “A,” “G,” “C,” or “T”; insertions are indicated by a dot followed by the number of repetition and the nucleotide position; reversions by “!”; green indicates synonymous, brown non-synonymous, yellow other coding region, and black control region substitutions. Individual identification is indicated as well as the geographic origin when known (geographic regions are grouped by colour code according to the key). Near the nodes, the TMRCA is indicated (mean and 95% confidence interval) for ρ based on whole-mtDNA sequences (in black), ρ based on synonymous diversity (in green) and for maximum likelihood (in blue).
(TIF)

S19 Fig Frequency maps based on HVS-I data for the haplogroup J2a2b. (TIF)

S20 Fig Phylogenetic tree of haplogroup T1a. Labels on the branches represent nucleotide positions of transitions, and transversions when followed by a suffix “A,” “G,” “C,” or “T”; insertions are indicated by a dot followed by the number of repetition and the nucleotide position; reversions by “!”; green indicates synonymous, brown non-synonymous, yellow other coding region, and black control region substitutions. Individual identification is indicated as well as the geographic origin when known (geographic regions are grouped by colour code according to the key). Near the nodes, the TMRCA is indicated (mean and 95% confidence interval) for ρ based on whole-mtDNA sequences (in black), ρ based on synonymous diversity (in green) and for maximum likelihood (in blue).
(TIF)

S21 Fig Phylogenetic tree of haplogroup T2a1. Labels on the branches represent nucleotide positions of transitions, and transversions when followed by a suffix “A,” “G,” “C,” or “T”; insertions are indicated by a dot followed by the number of repetition and the nucleotide position; reversions by “!”; green indicates synonymous, brown non-synonymous, yellow other coding region, and black control region substitutions. Individual identification is indicated as well as the geographic origin when known (geographic regions are grouped by colour code according to the key). Near the nodes, the TMRCA is indicated (mean and 95% confidence interval) for ρ based on whole-mtDNA sequences (in black), ρ based on synonymous diversity (in green) and for maximum likelihood (in blue).
(TIF)

S22 Fig Phylogenetic tree of haplogroup T2c. Labels on the branches represent nucleotide positions of transitions, and transversions when followed by a suffix “A,” “G,” “C,” or “T”; insertions are indicated by a dot followed by the number of repetition and the nucleotide position; reversions by “!”; green indicates synonymous, brown non-synonymous, yellow other coding region, and black control region substitutions. Individual identification is indicated as well as the geographic origin when known (geographic regions are grouped by colour code according to the key). Near the nodes, the TMRCA is indicated (mean and 95% confidence interval) for ρ based on whole-mtDNA sequences (in black), ρ based on synonymous diversity (in green) and for maximum likelihood (in blue).
(TIF)

S23 Fig Phylogenetic tree of haplogroups T2i and T2g. Labels on the branches represent nucleotide positions of transitions, and transversions when followed by a suffix “A,” “G,” “C,” or “T”; insertions are indicated by a dot followed by the number of repetition and the nucleotide position; reversions by “!”; green indicates synonymous, brown non-synonymous, yellow other coding region, and black control region substitutions. Individual identification is indicated as well as the geographic origin when known (geographic regions are grouped by colour code according to the key). Near the nodes, the TMRCA is indicated (mean and 95% confidence interval) for ρ based on whole-mtDNA sequences (in black), ρ based on synonymous diversity (in green) and for maximum likelihood (in blue).
(TIF)

S24 Fig Probabilistic proportion of founder clusters considering three migration periods (1.0, 10.0 and 16.0 ka), using the *f1* criterion and by assuming a Near East, Iran and Pakistan source for migrations into Arabian Peninsula. The haplogroup affiliations of the founders are indicated in the bottom.
(TIF)

S25 Fig Probabilistic proportion of founder clusters considering three migration periods (1.0, 10.0 and 16.0 ka), using the *f2* criterion and by assuming a Near East, Iran and Pakistan source for migrations into Arabian Peninsula. The haplogroup affiliations of the founders are indicated in the bottom.
(TIF)

S26 Fig Schematic tree of haplogroups L4 and L6. Ages (in ka) indicated are maximum likelihood estimates obtained with the whole-mtDNA genome.
(TIF)

S27 Fig Frequency maps based on HVS-I data for haplogroups L4 (A) and L6 (B). (TIF)

S28 Fig Phylogenetic tree of haplogroup L4a. Labels on the branches represent nucleotide positions of transitions, and transversions when followed by a suffix “A,” “G,” “C,” or “T”; reversions by “!”; green indicates synonymous, brown non-synonymous, yellow other coding region, and black control region substitutions. Individual identification is indicated as well as the geographic origin when known (geographic regions are grouped by colour code according to the key). Near the nodes, the TMRCA is indicated (mean and 95% confidence interval) for ρ based on whole-mtDNA sequences (in black), ρ based on synonymous diversity (in green) and for maximum likelihood (in blue).
(TIF)

S29 Fig Phylogenetic tree of haplogroup L4b. Labels on the branches represent nucleotide positions of transitions, and transversions when followed by a suffix “A,” “G,” “C,” or “T”; reversions by “!”; green indicates synonymous, brown non-synonymous, yellow other coding region, and black control region substitutions. Individual identification is indicated as well as the geographic origin when known (geographic regions are grouped by colour code according to the key). Near the nodes, the TMRCA is indicated (mean and 95% confidence interval) for ρ based on whole-mtDNA sequences (in black), ρ based on synonymous diversity (in green) and for maximum likelihood (in blue).
(TIF)

S30 Fig Phylogenetic tree of haplogroup L6. Labels on the branches represent nucleotide positions of transitions, and transversions when followed by a suffix “A,” “G,” “C,” or “T”; reversions by “!”; green indicates synonymous, brown non-synonymous, yellow other coding region, and black control region substitutions. Individual identification is indicated as well as the geographic origin when known (geographic regions are grouped by colour code according to the key). Near the nodes, the TMRCA is indicated (mean and 95% confidence interval) for ρ based on whole-mtDNA sequences (in black), ρ based on synonymous diversity (in green) and for maximum likelihood (in blue).
(TIF)

S31 Fig Bayesian Skyline Plot (BSP), indicating the median of the hypothetical effective population size through time based on data from haplogroup L4 (A) and haplogroups L4 and L6 (B), assuming a generation time of 25 years. (TIF)

S32 Fig Probabilistic proportion of founder clusters considering three migration periods (1.0, 2.5 and 13.0 ka), using the *f1* criterion and assuming an African source for migrations into Arabian Peninsula plus the Near East and Iran. The haplogroup affiliations of the founders are indicated in the bottom.
(TIF)

S33 Fig Probabilistic proportion of founder clusters considering three migration periods (1.0, 2.5 and 13.0 ka), using the *f2* criterion and assuming an African source for migrations into Arabian Peninsula plus Near East and Iran. The haplogroup affiliations of the founders are indicated in the bottom.
(TIF)

S34 Fig Probabilistic proportion of founder clusters considering three migration periods (2.0, 10.0 and 16.0 ka), using the *f1* criterion and assuming Arabian Peninsula plus Near East and Iran migrations into eastern Africa. The haplogroup affiliations of the founders are indicated in the bottom.
(TIF)

S35 Fig Probabilistic proportion of founder clusters considering three migration periods (2.0, 10.0 and 16.0 ka), using the *f2* criterion and assuming Arabian Peninsula plus Near East and Iran migrations into eastern Africa. The haplogroup affiliations of the founders are indicated in the bottom.
(TIF)

S36 Fig Probabilistic proportion of founder clusters considering three migration periods (2.0, 10.0 and 16.0 ka), using *f1* criterion and assuming Arabian Peninsula plus Near East and Iran migrations into North Africa. The haplogroup affiliations of the founders are indicated in the bottom.
(TIF)

S37 Fig Probabilistic proportion of founder clusters considering three migration periods (2.0, 10.0 and 16.0 ka), using the *f2* criterion and assuming Arabian Peninsula plus Near East and Iran migrations into North Africa. The haplogroup affiliations of the founders are indicated in the bottom.
(TIF)

S38 Fig Population structure inferred by ADMIXTURE analysis. Each individual is represented by a vertical (100%) stacked column of genetic components proportions shown in colour for K = 3, 4 and 5.
(TIF)

S1 Table Haplotypes for whole-mtDNA sequences that were fully characterised in this study and the corresponding geographic region. (DOCX)

S2 Table Published whole-mtDNA sequences used in all phylogenetic tree with the corresponding origin and subclade affiliation. (DOCX)

S3 Table Diversity values of ρ and π used for the interpolation maps of the haplogroups J, T and L4. (DOCX)

S4 Table Frequency values used in the reconstruction of the interpolation maps for the haplogroups J, T, J1d1a and J2a2b. (DOCX)

S5 Table Frequency values used in the reconstruction of the interpolation maps for the haplogroups L4 and L6. (DOCX)

S6 Table Founder lineages identified when using *f1* criterion from the Near East, Iran and Pakistan to Arabian Peninsula. (DOCX)

S7 Table Founder lineages identified when using *f2* criterion from the Near East, Iranand Pakistan to Arabian Peninsula. (DOCX)

S8 Table Founder lineages identified when using a *f1* criterion from Near East, Iran and Pakistan to Arabian Peninsula, based on whole-mtDNA JT sequences. (DOCX)

S9 Table Founder lineages identified when using *f1* criterion from Africa to Arabian Peninsula, Near East and Iran. (DOCX)

S10 Table Founder lineages identified when using *f2* criterion from Africa to Arabian Peninsula, Near East and Iran. (DOCX)

S11 Table Founder lineages identified when using *f1* criterion from Arabian Peninsula, Near East and Iran to North Africa and to eastern Africa separately. (DOCX)

S12 Table Founder lineages identified when using *f2* criterion from Arabian Peninsula, Near East and Iran to North Africa and to eastern Africa separately. (DOCX)

S13 Table Samples used for genome-wide autosomal analysis. (DOCX)

S14 Table Peaks of rate of population size change through time as obtained from the BSPs and periods of time where the rate of population size increase was of at least one individual per 100 individuals in a period of 100 years. Increment ratio corresponds to the number of times the effective population size increase during this period.
(DOCX)

S15 Table Ages for the oldest founders in the migration from Africa into the Arabian Peninsula, Near East and Iran. This is a sub-set of S9 Table.
(DOCX)

S1 Text Phylogeographic analyses and supplemental information on founder analyses. Includes 15 tables.
(DOCX)
