# Supplementary figures and images for: Genetic Stratigraphy of Key Demographic Events in Arabia
Source: PLoS One. 2015 Mar 4;10(3):e0118625. doi: 10.1371/journal.pone.0118625 (PMC4349752; doi:10.1371/journal.pone.0118625)

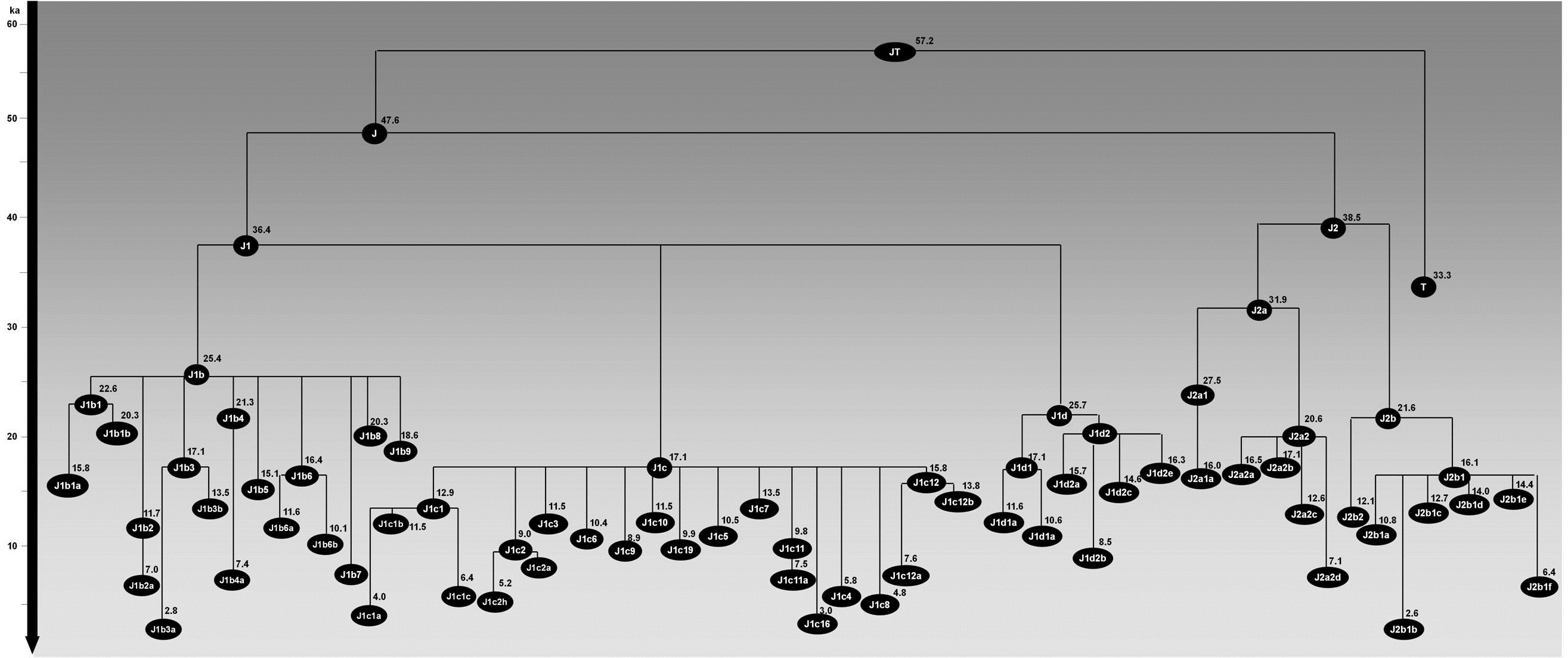

Supplement: S1 Fig — Ages (in ka) indicated are maximum likelihood estimates obtained for the whole-mtDNA genome. (TIF) [file pone.0118625.s001.tif]

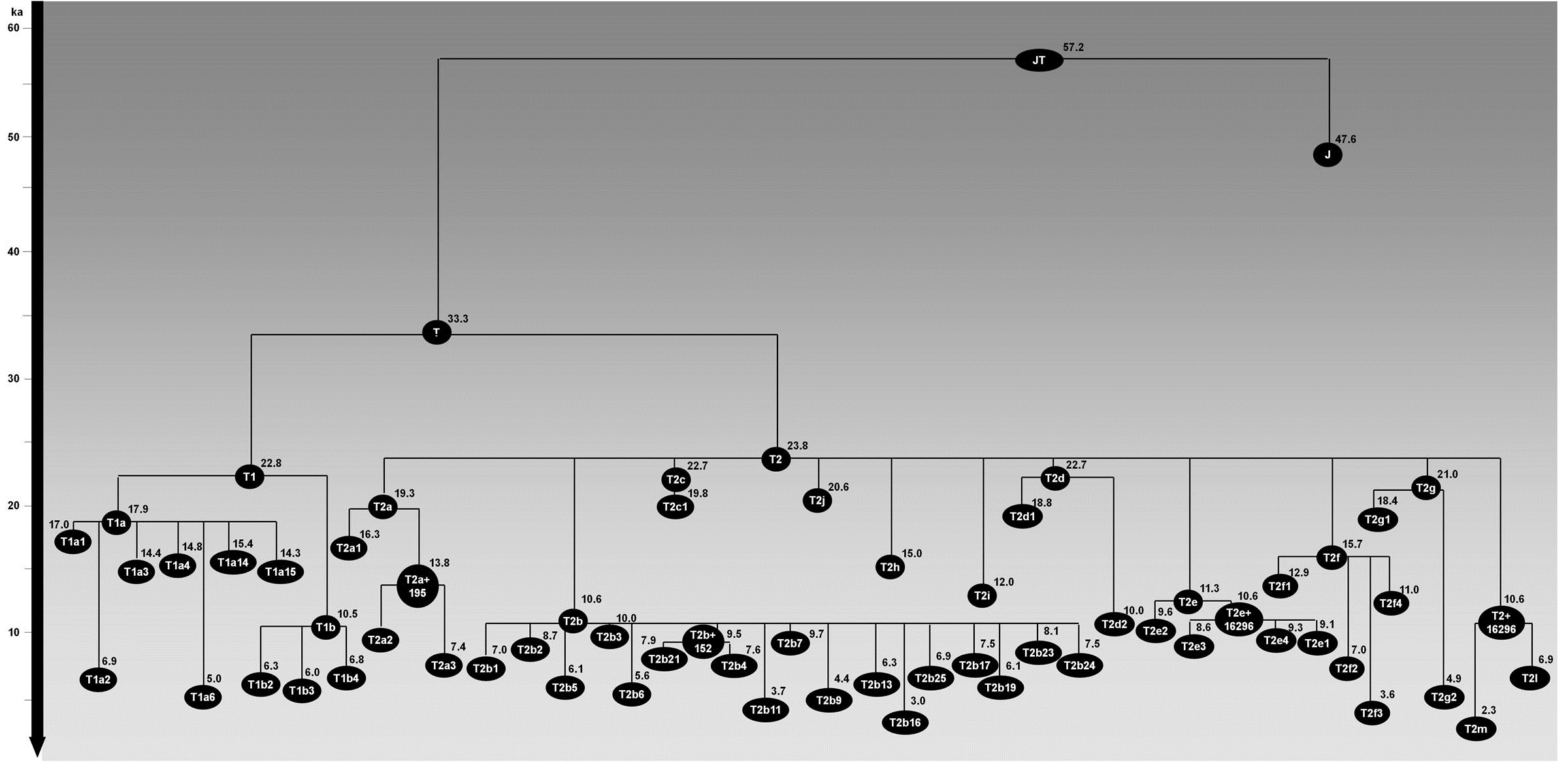

Supplement: S2 Fig — Ages (in ka) indicated are maximum likelihood estimates obtained for the whole-mtDNA genome. (TIF) [file pone.0118625.s002.tif]

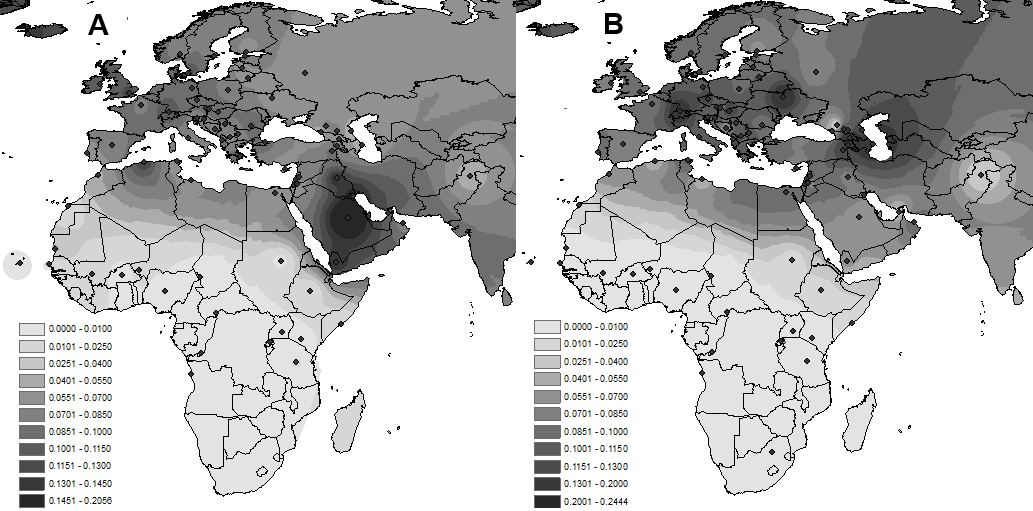

Supplement: S3 Fig — (TIF) [file pone.0118625.s003.tif]

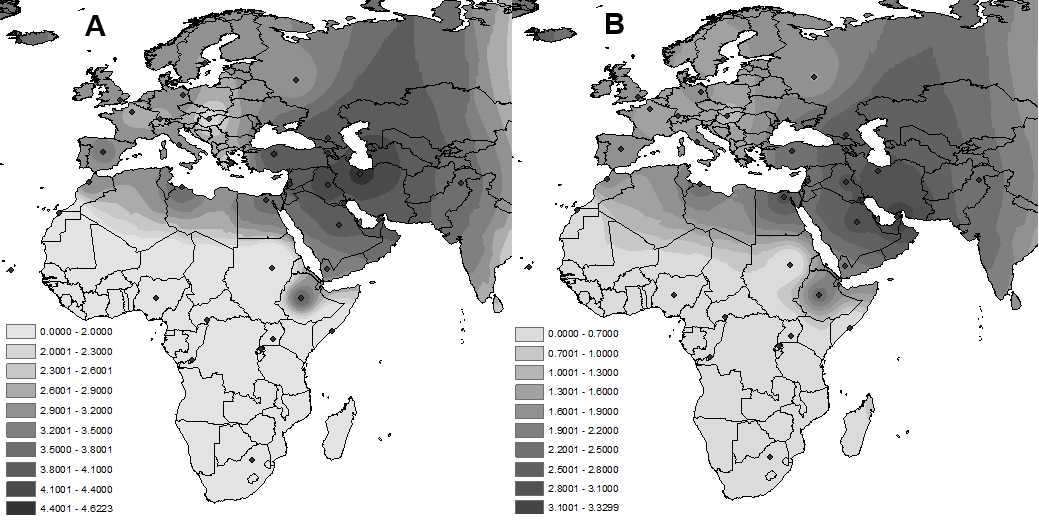

Supplement: S4 Fig — (TIF) [file pone.0118625.s004.tif]

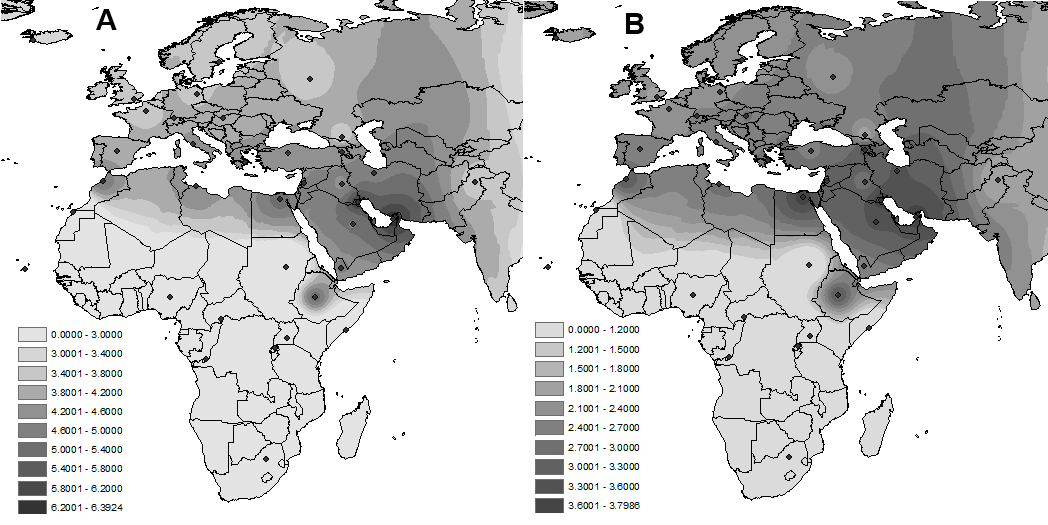

Supplement: S5 Fig — (TIF) [file pone.0118625.s005.tif]

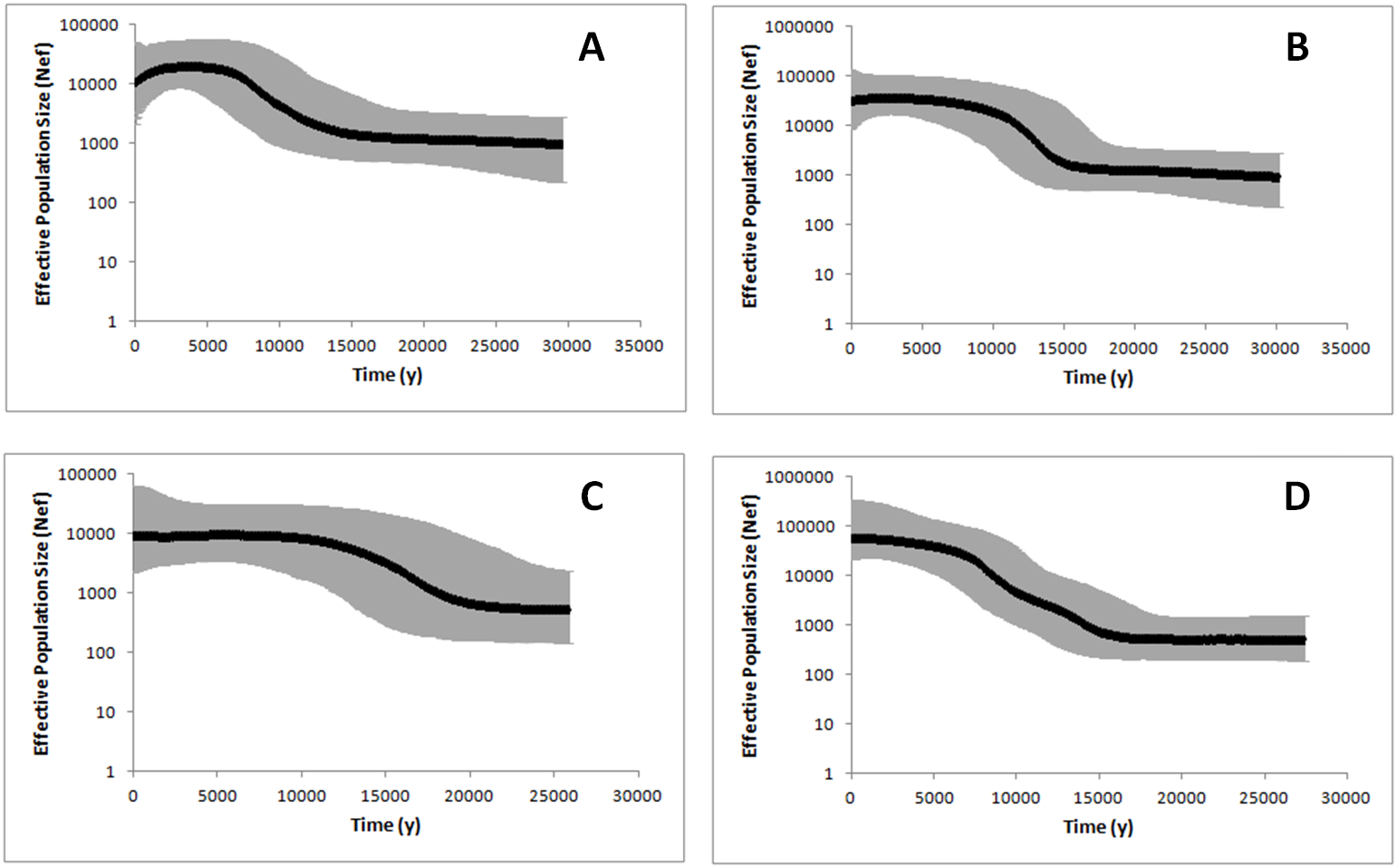

Supplement: S6 Fig — (TIF) [file pone.0118625.s006.tif]

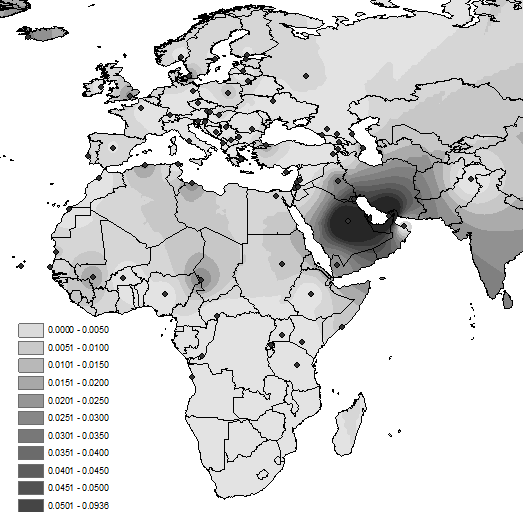

Supplement: S7 Fig — (TIF) [file pone.0118625.s007.tif]

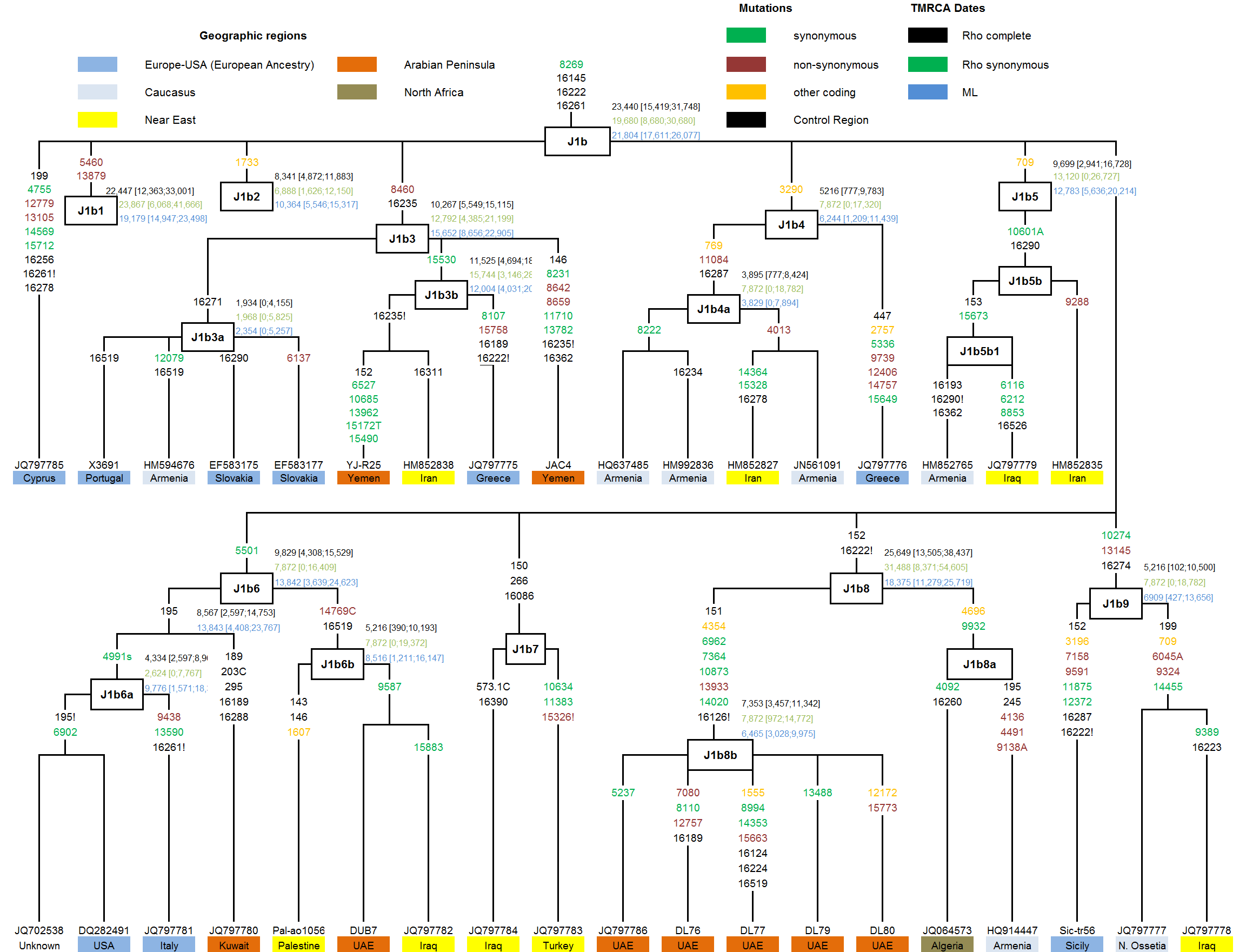

Supplement: S8 Fig — Labels on the branches represent nucleotide positions of transitions, and transversions when followed by a suffix “A,” “G,” “C,” or “T”; insertions are indicated by a dot followed by the number of repetitions and the nucleotide position; reversions by “!”; green indicates synonymous, brown non-synonymous, yellow other coding region, and black control region substitutions. Individual identification is indicated as well as the geographic origin when known (geographic regions are grouped by colour code according to the key). Near the nodes, the TMRCA is indicated (mean and 95% confidence interval) for ρ based on whole-mtDNA sequences (in black), ρ based on synonymous diversity (in green) and for maximum likelihood (in blue). (TIF) [file pone.0118625.s008.tif]

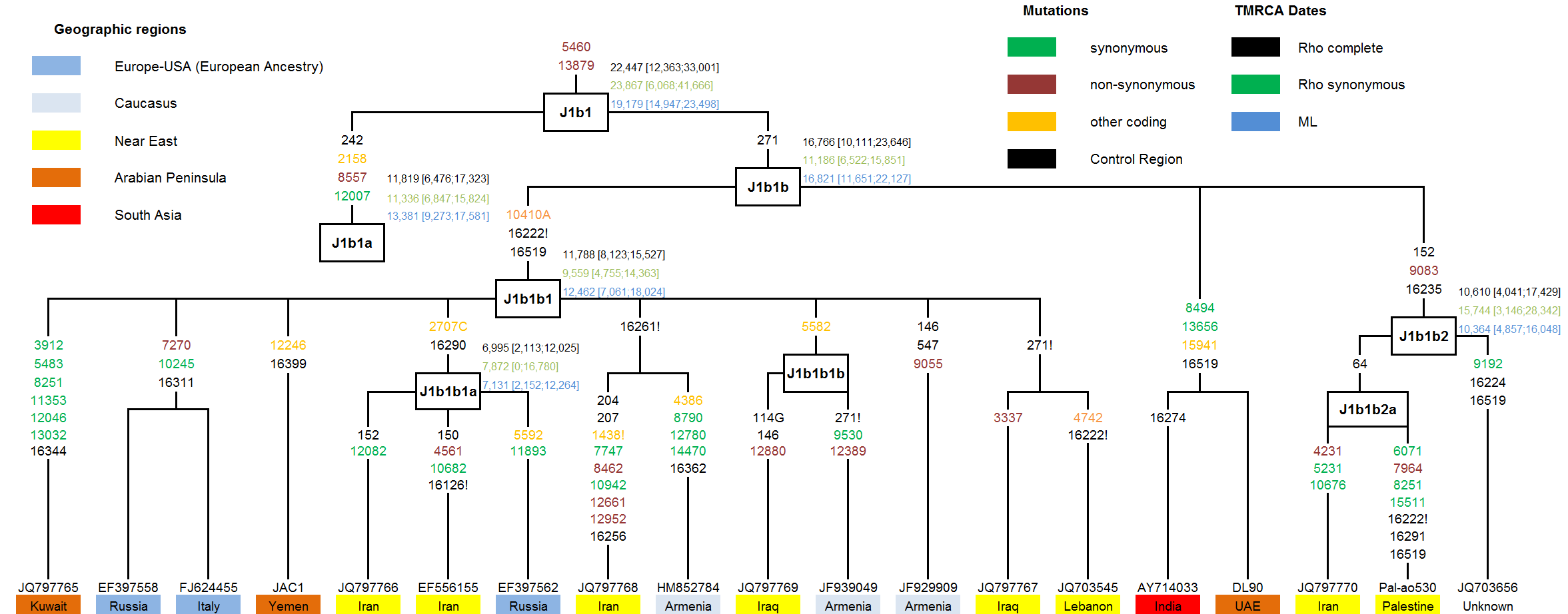

Supplement: S9 Fig — Labels on the branches represent nucleotide positions of transitions, and transversions when followed by a suffix “A,” “G,” “C,” or “T”; reversions by “!”; green indicates synonymous, brown non-synonymous, yellow other coding region, and black control region substitutions. Individual identification is indicated as well as the geographic origin when known (geographic regions are grouped by colour code according to the key). Near the nodes, the TMRCA is indicated (mean and 95% confidence interval) for ρ based on whole-mtDNA sequences (in black), ρ based on synonymous diversity (in green) and for maximum likelihood (in blue). (TIF) [file pone.0118625.s009.tif]

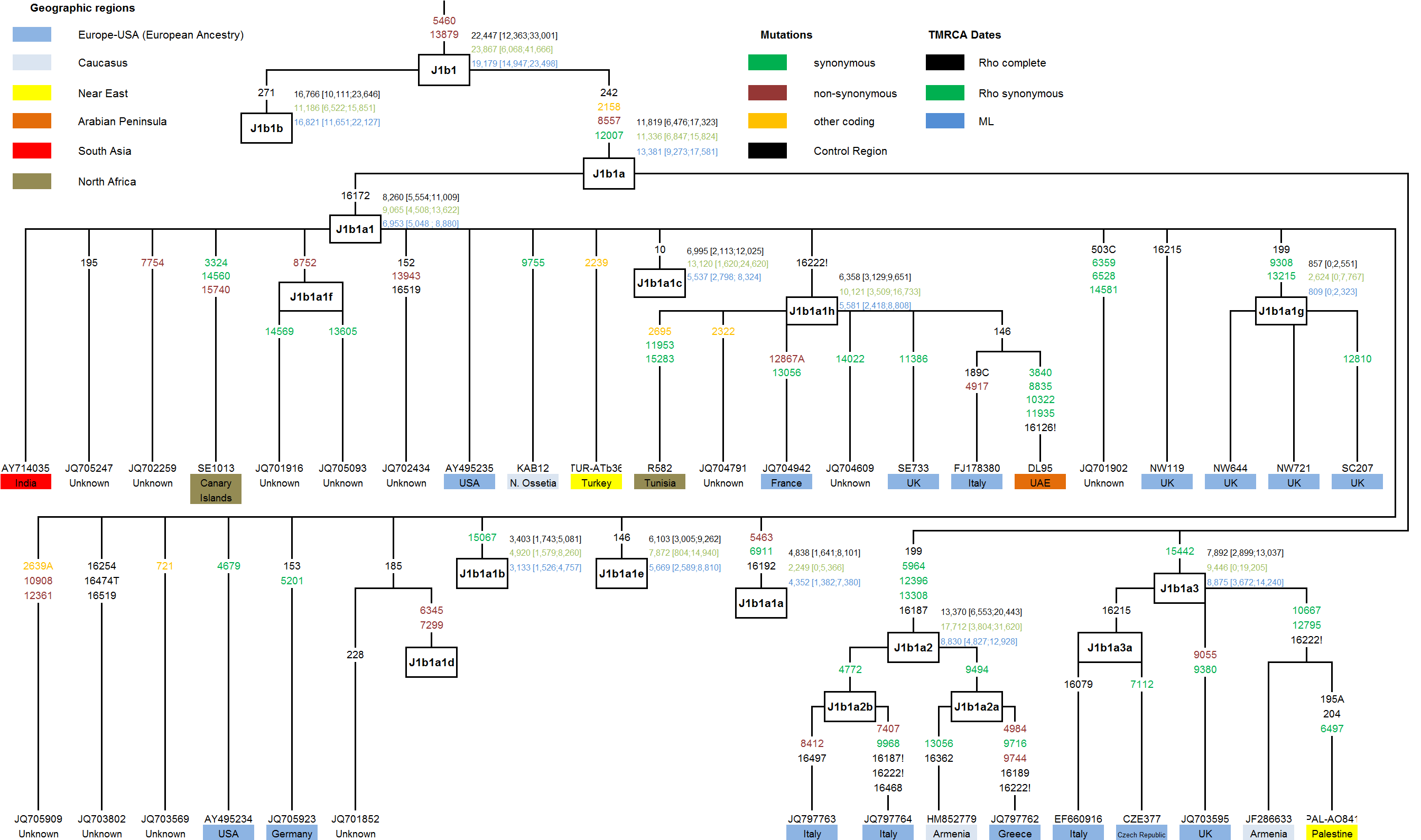

Supplement: S10 Fig — Labels on the branches represent nucleotide positions of transitions, and transversions when followed by a suffix “A,” “G,” “C,” or “T”; reversions by “!”; green indicates synonymous, brown non-synonymous, yellow other coding region, and black control region substitutions. Individual identification is indicated as well as the geographic origin when known (geographic regions are grouped by colour code according to the key). Near the nodes, the TMRCA is indicated (mean and 95% confidence interval) for ρ based on whole-mtDNA sequences (in black), ρ based on synonymous diversity (in green) and for maximum likelihood (in blue). (TIF) [file pone.0118625.s010.tif]

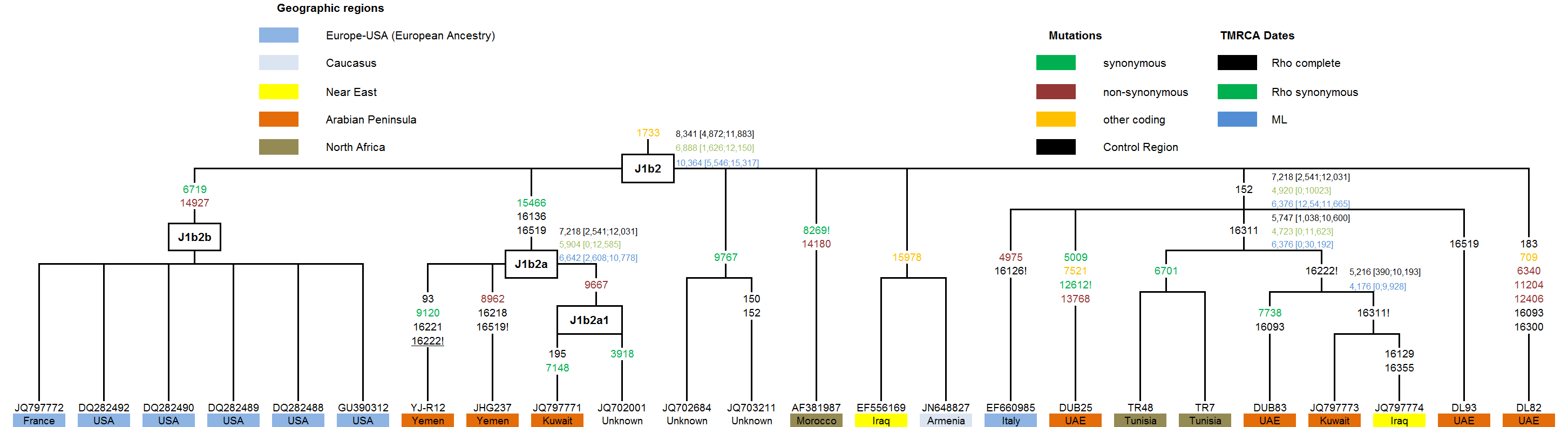

Supplement: S11 Fig — Labels on the branches represent nucleotide positions of transitions, and transversions when followed by a suffix “A,” “G,” “C,” or “T”; reversions by “!”; green indicates synonymous, brown non-synonymous, yellow other coding region, and black control region substitutions. Individual identification is indicated as well as the geographic origin when known (geographic regions are grouped by colour code according to the key). Near the nodes, the TMRCA is indicated (mean and 95% confidence interval) for ρ based on whole-mtDNA sequences (in black), ρ based on synonymous diversity (in green) and for maximum likelihood (in blue). (TIF) [file pone.0118625.s011.tif]

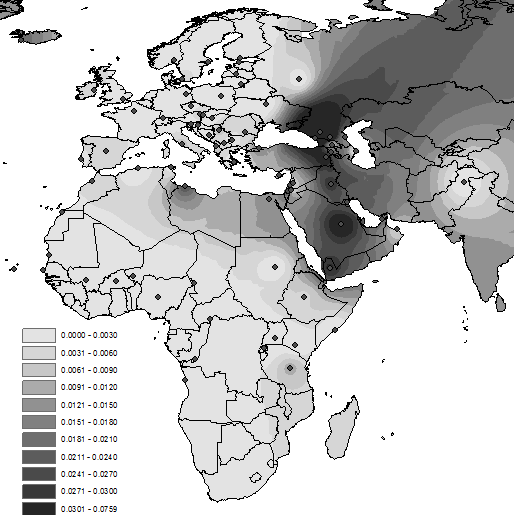

Supplement: S12 Fig — (TIF) [file pone.0118625.s012.tif]

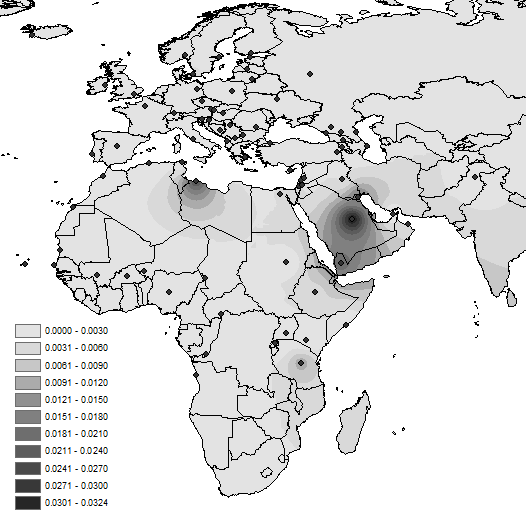

Supplement: S13 Fig — (TIF) [file pone.0118625.s013.tif]

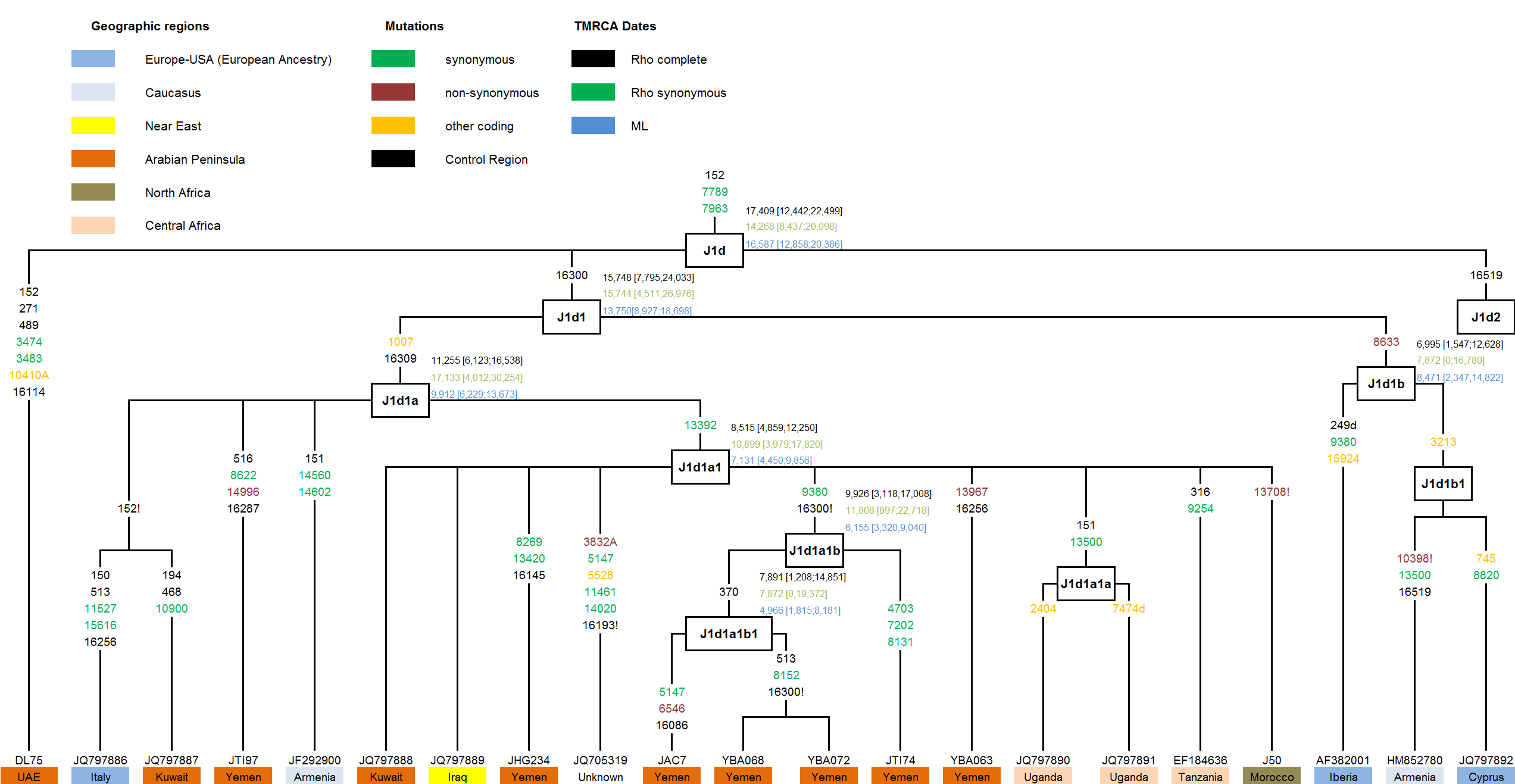

Supplement: S14 Fig — Labels on the branches represent nucleotide positions of transitions, and transversions when followed by a suffix “A,” “G,” “C,” or “T”; deletions are indicated “d”; reversions by “!”; green indicates synonymous, brown non-synonymous, yellow other coding region, and black control region substitutions. Individual identification is indicated as well as the geographic origin when known (geographic regions are grouped by colour code according to the key). Near the nodes, the TMRCA is indicated (mean and 95% confidence interval) for ρ based on whole-mtDNA sequences (in black), ρ based on synonymous diversity (in green) and for maximum likelihood (in blue). (TIF) [file pone.0118625.s014.tif]

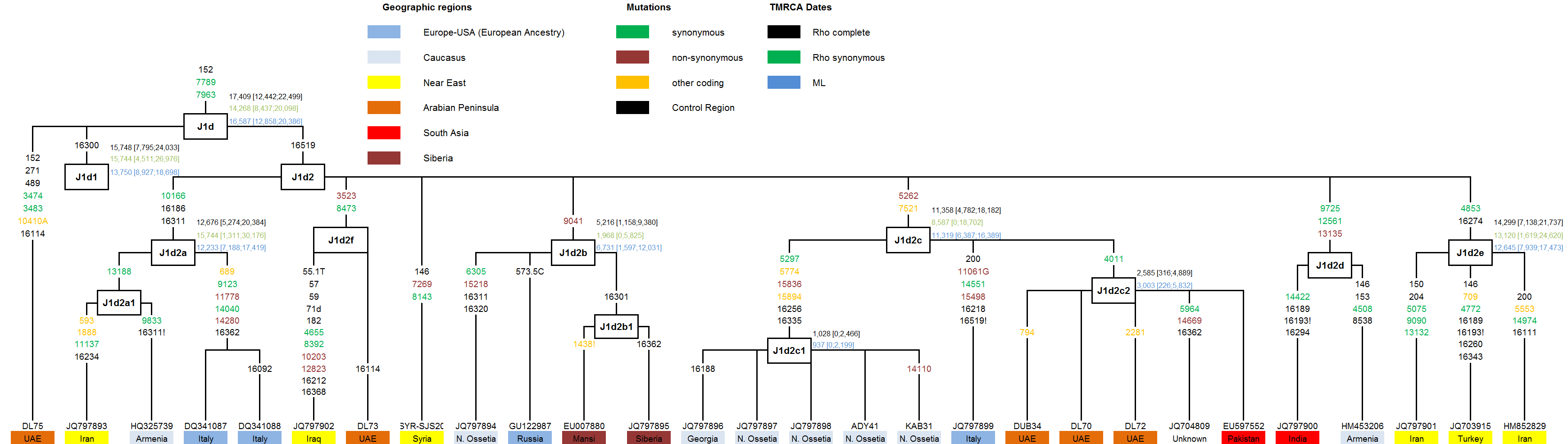

Supplement: S15 Fig — Labels on the branches represent nucleotide positions of transitions, and transversions when followed by a suffix “A,” “G,” “C,” or “T”; insertions are indicated by a dot followed by the number of repetition and the nucleotide position; reversions by “!”; green indicates synonymous, brown non-synonymous, yellow other coding region, and black control region substitutions. Individual identification is indicated as well as the geographic origin when known (geographic regions are grouped by colour code according to the key). Near the nodes, the TMRCA is indicated (mean and 95% confidence interval) for ρ based on whole-mtDNA sequences (in black), ρ based on synonymous diversity (in green) and for maximum likelihood (in blue). (TIF) [file pone.0118625.s015.tif]

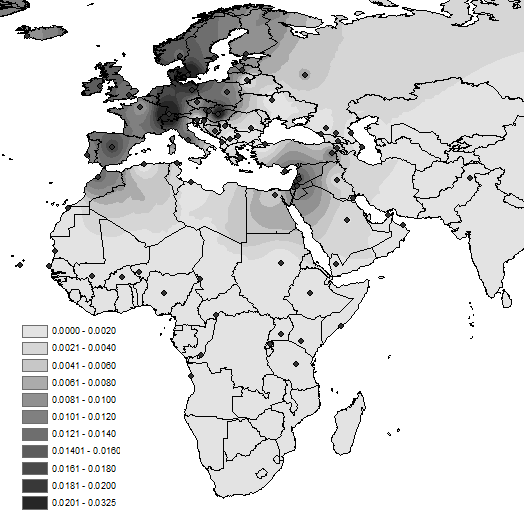

Supplement: S16 Fig — (TIF) [file pone.0118625.s016.tif]

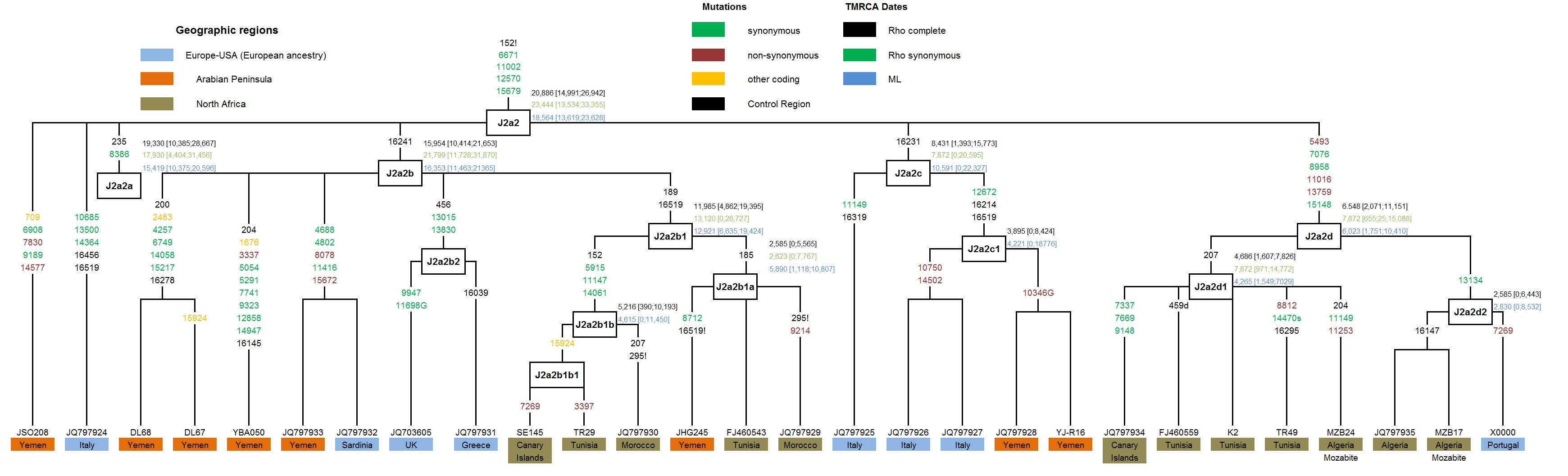

Supplement: S17 Fig — Labels on the branches represent nucleotide positions of transitions, and transversions when followed by a suffix “A,” “G,” “C,” or “T”; reversions by “!”; green indicates synonymous, brown non-synonymous, yellow other coding region, and black control region substitutions. Individual identification is indicated as well as the geographic origin when known (geographic regions are grouped by colour code according to the key). Near the nodes, the TMRCA is indicated (mean and 95% confidence interval) for ρ based on whole-mtDNA sequences (in black), ρ based on synonymous diversity (in green) and for maximum likelihood (in blue). (TIF) [file pone.0118625.s017.tif]

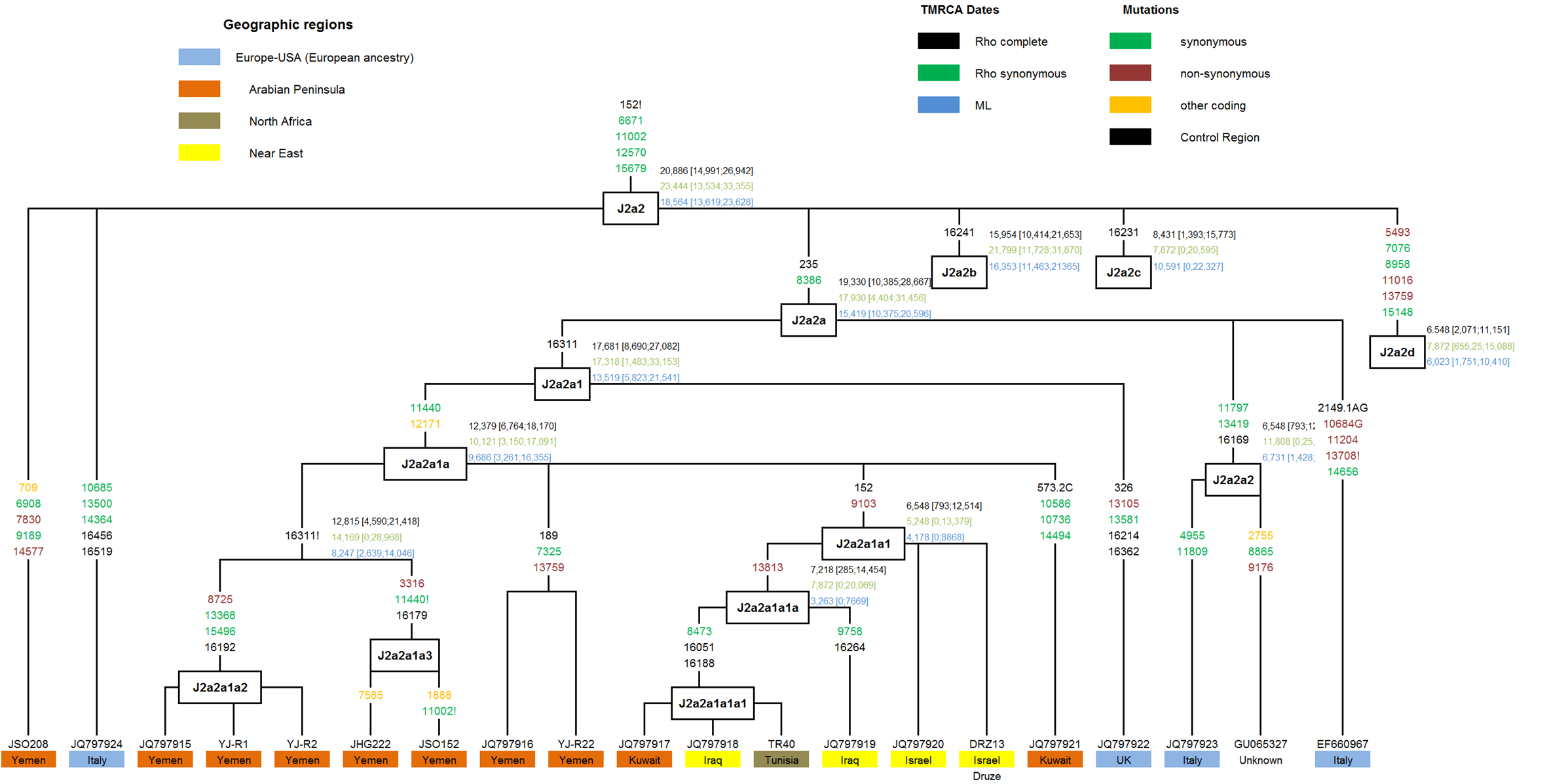

Supplement: S18 Fig — Labels on the branches represent nucleotide positions of transitions, and transversions when followed by a suffix “A,” “G,” “C,” or “T”; insertions are indicated by a dot followed by the number of repetition and the nucleotide position; reversions by “!”; green indicates synonymous, brown non-synonymous, yellow other coding region, and black control region substitutions. Individual identification is indicated as well as the geographic origin when known (geographic regions are grouped by colour code according to the key). Near the nodes, the TMRCA is indicated (mean and 95% confidence interval) for ρ based on whole-mtDNA sequences (in black), ρ based on synonymous diversity (in green) and for maximum likelihood (in blue). (TIF) [file pone.0118625.s018.tif]

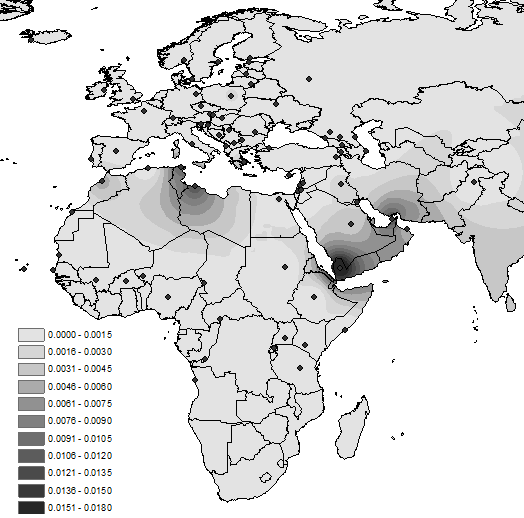

Supplement: S19 Fig — (TIF) [file pone.0118625.s019.tif]

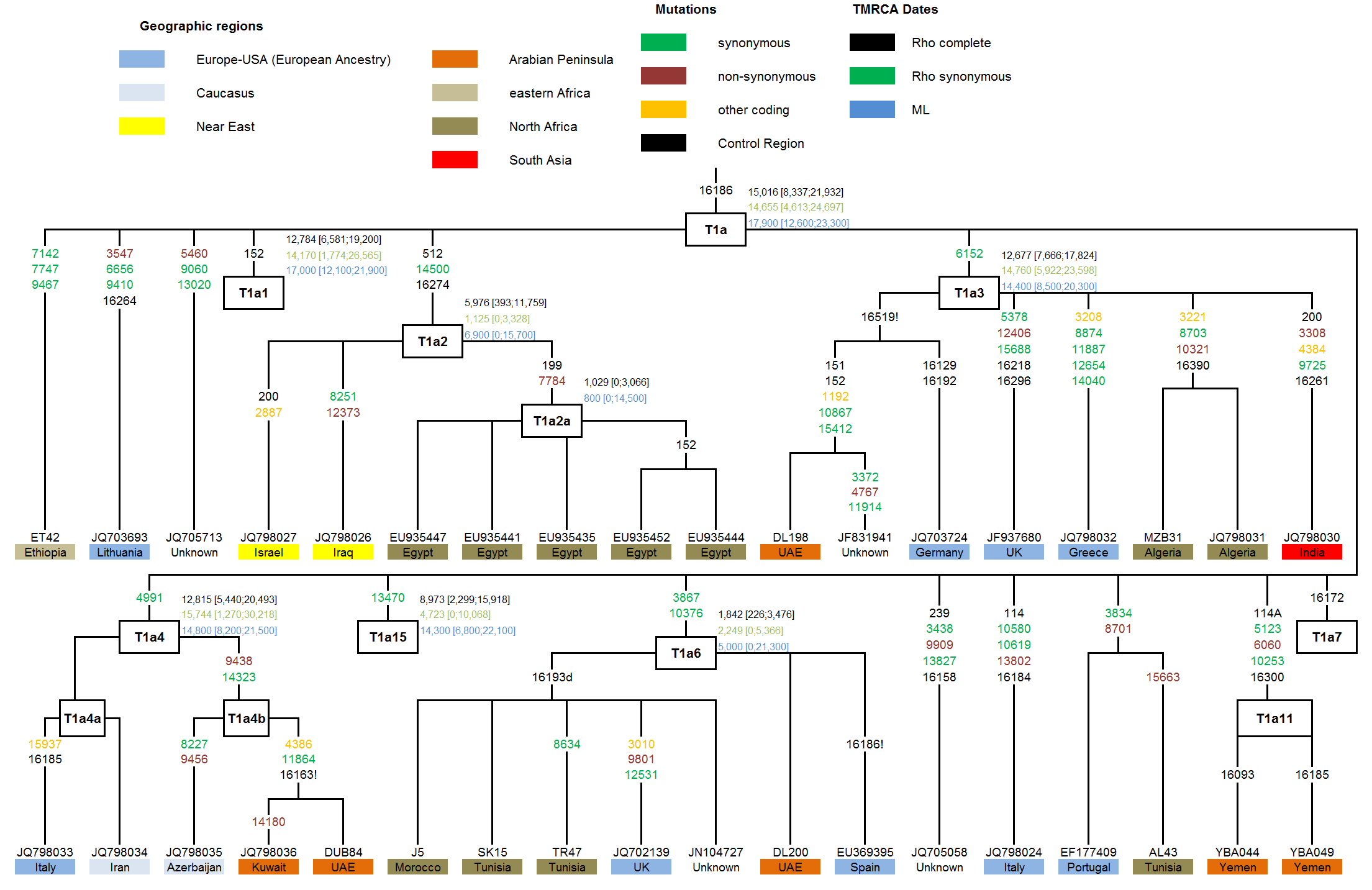

Supplement: S20 Fig — Labels on the branches represent nucleotide positions of transitions, and transversions when followed by a suffix “A,” “G,” “C,” or “T”; insertions are indicated by a dot followed by the number of repetition and the nucleotide position; reversions by “!”; green indicates synonymous, brown non-synonymous, yellow other coding region, and black control region substitutions. Individual identification is indicated as well as the geographic origin when known (geographic regions are grouped by colour code according to the key). Near the nodes, the TMRCA is indicated (mean and 95% confidence interval) for ρ based on whole-mtDNA sequences (in black), ρ based on synonymous diversity (in green) and for maximum likelihood (in blue). (TIF) [file pone.0118625.s020.tif]

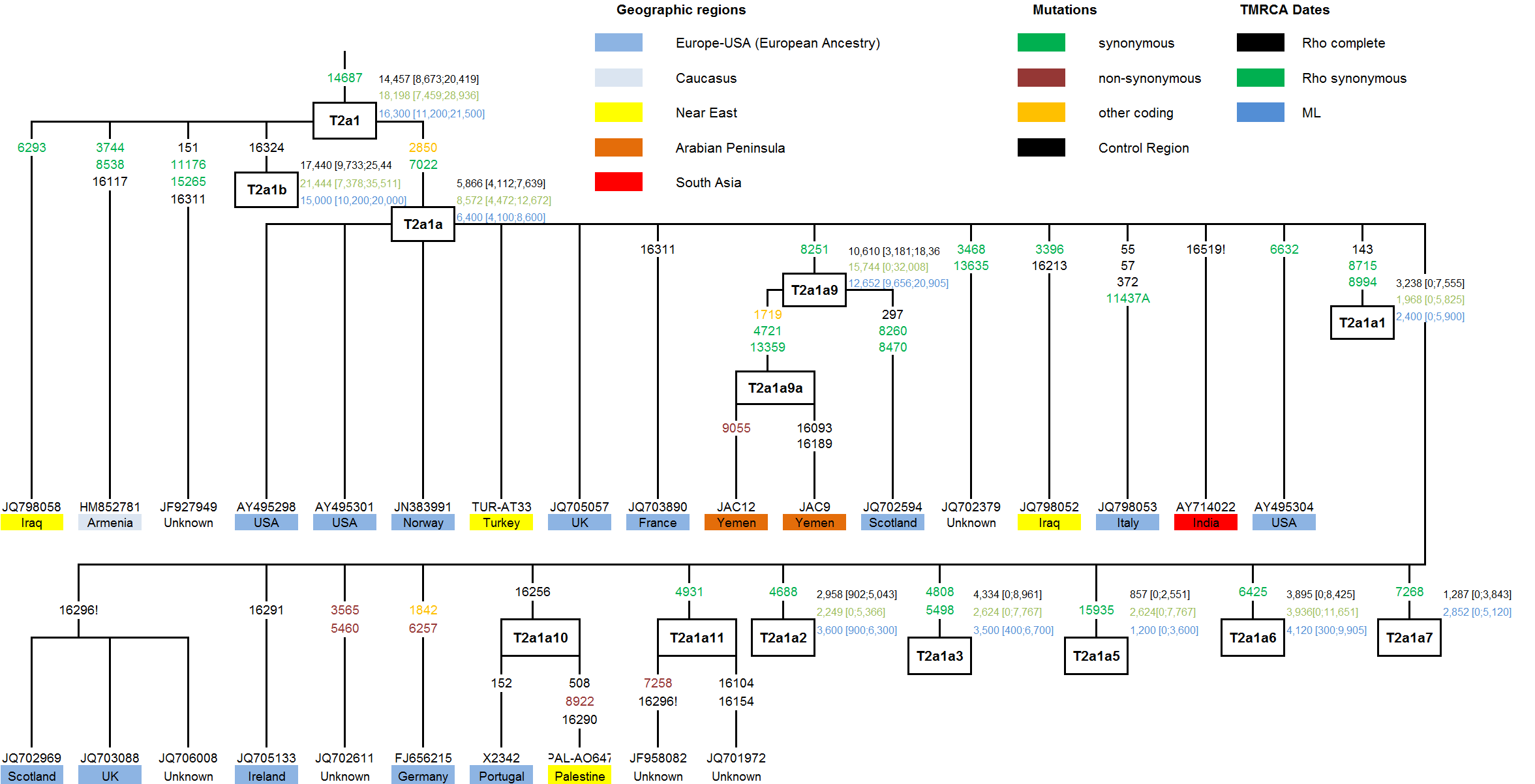

Supplement: S21 Fig — Labels on the branches represent nucleotide positions of transitions, and transversions when followed by a suffix “A,” “G,” “C,” or “T”; insertions are indicated by a dot followed by the number of repetition and the nucleotide position; reversions by “!”; green indicates synonymous, brown non-synonymous, yellow other coding region, and black control region substitutions. Individual identification is indicated as well as the geographic origin when known (geographic regions are grouped by colour code according to the key). Near the nodes, the TMRCA is indicated (mean and 95% confidence interval) for ρ based on whole-mtDNA sequences (in black), ρ based on synonymous diversity (in green) and for maximum likelihood (in blue). (TIF) [file pone.0118625.s021.tif]

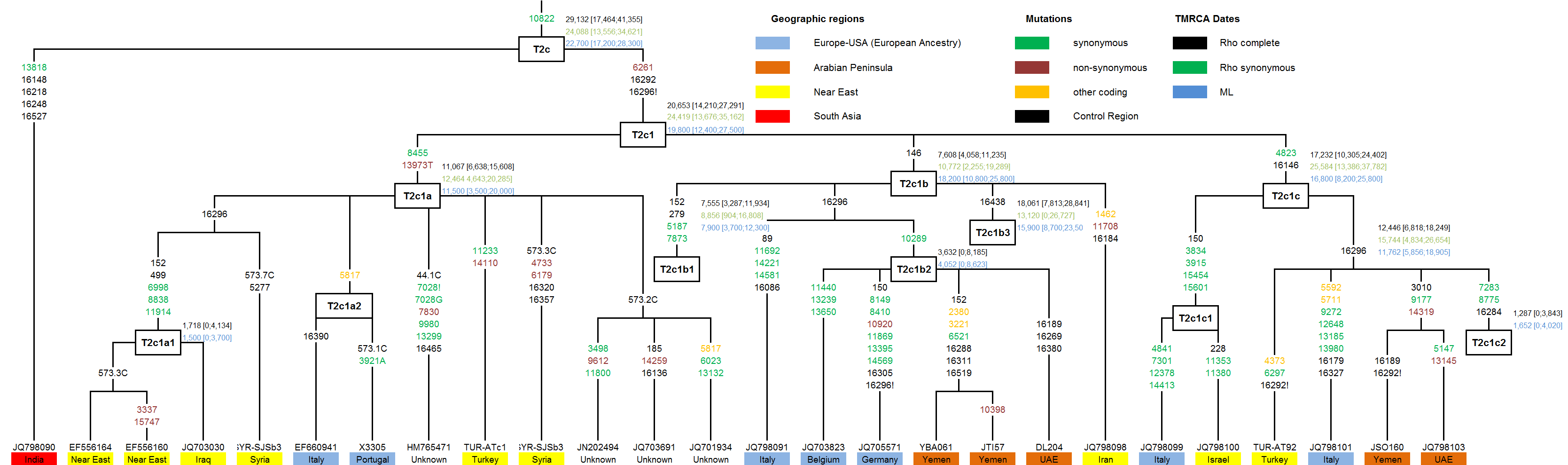

Supplement: S22 Fig — Labels on the branches represent nucleotide positions of transitions, and transversions when followed by a suffix “A,” “G,” “C,” or “T”; insertions are indicated by a dot followed by the number of repetition and the nucleotide position; reversions by “!”; green indicates synonymous, brown non-synonymous, yellow other coding region, and black control region substitutions. Individual identification is indicated as well as the geographic origin when known (geographic regions are grouped by colour code according to the key). Near the nodes, the TMRCA is indicated (mean and 95% confidence interval) for ρ based on whole-mtDNA sequences (in black), ρ based on synonymous diversity (in green) and for maximum likelihood (in blue). (TIF) [file pone.0118625.s022.tif]

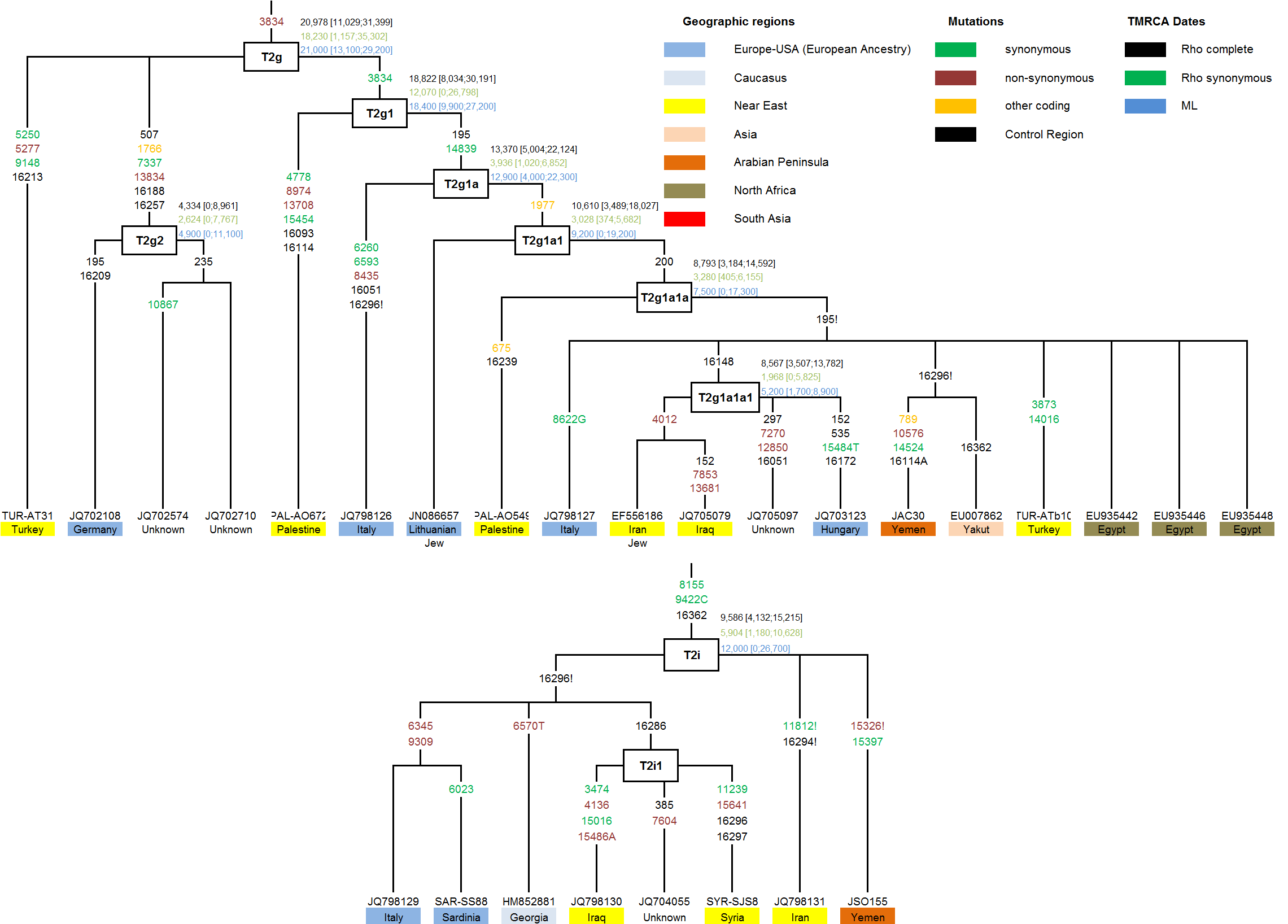

Supplement: S23 Fig — Labels on the branches represent nucleotide positions of transitions, and transversions when followed by a suffix “A,” “G,” “C,” or “T”; insertions are indicated by a dot followed by the number of repetition and the nucleotide position; reversions by “!”; green indicates synonymous, brown non-synonymous, yellow other coding region, and black control region substitutions. Individual identification is indicated as well as the geographic origin when known (geographic regions are grouped by colour code according to the key). Near the nodes, the TMRCA is indicated (mean and 95% confidence interval) for ρ based on whole-mtDNA sequences (in black), ρ based on synonymous diversity (in green) and for maximum likelihood (in blue). (TIF) [file pone.0118625.s023.tif]

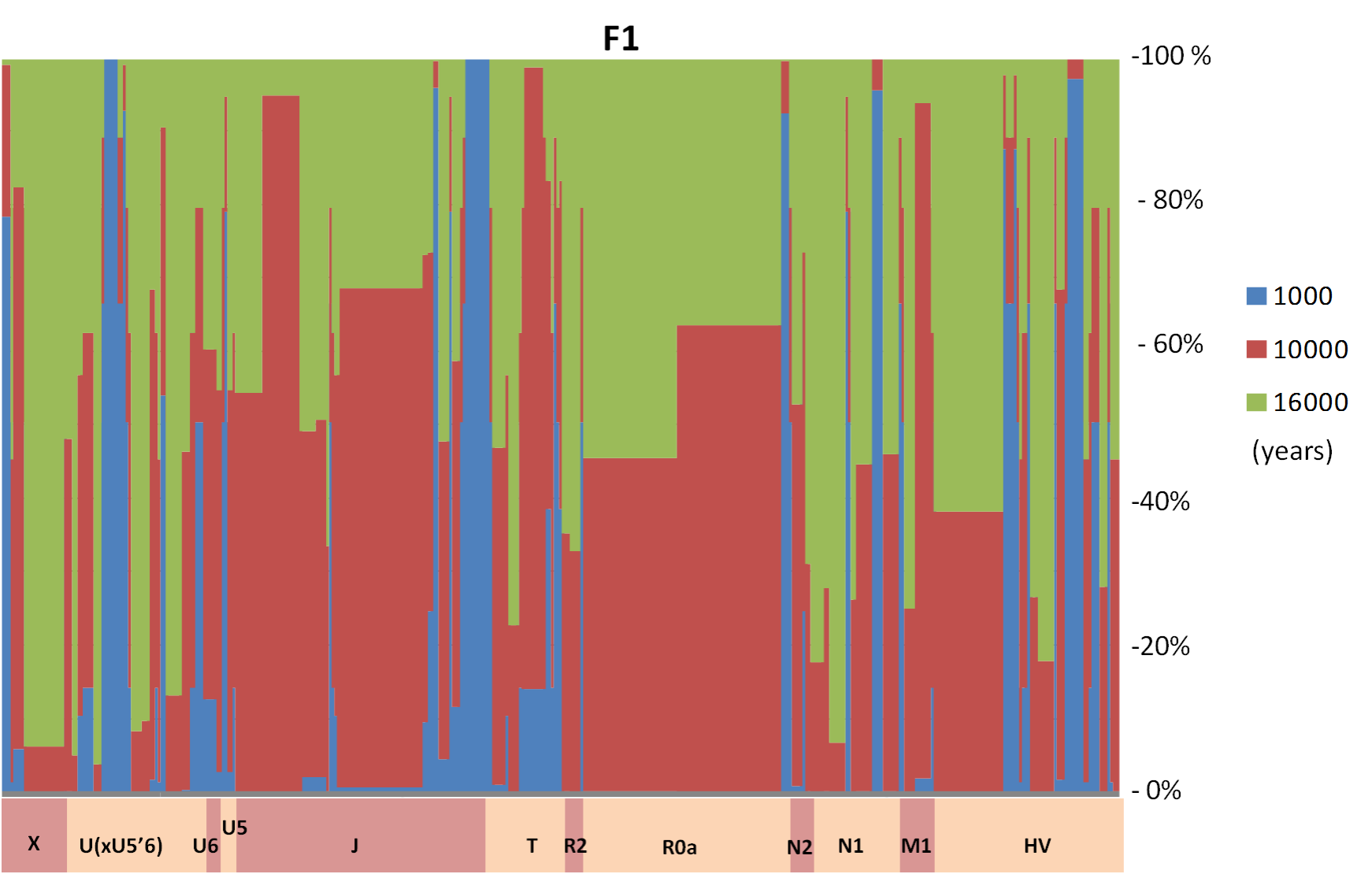

Supplement: S24 Fig — The haplogroup affiliations of the founders are indicated in the bottom. (TIF) [file pone.0118625.s024.tif]

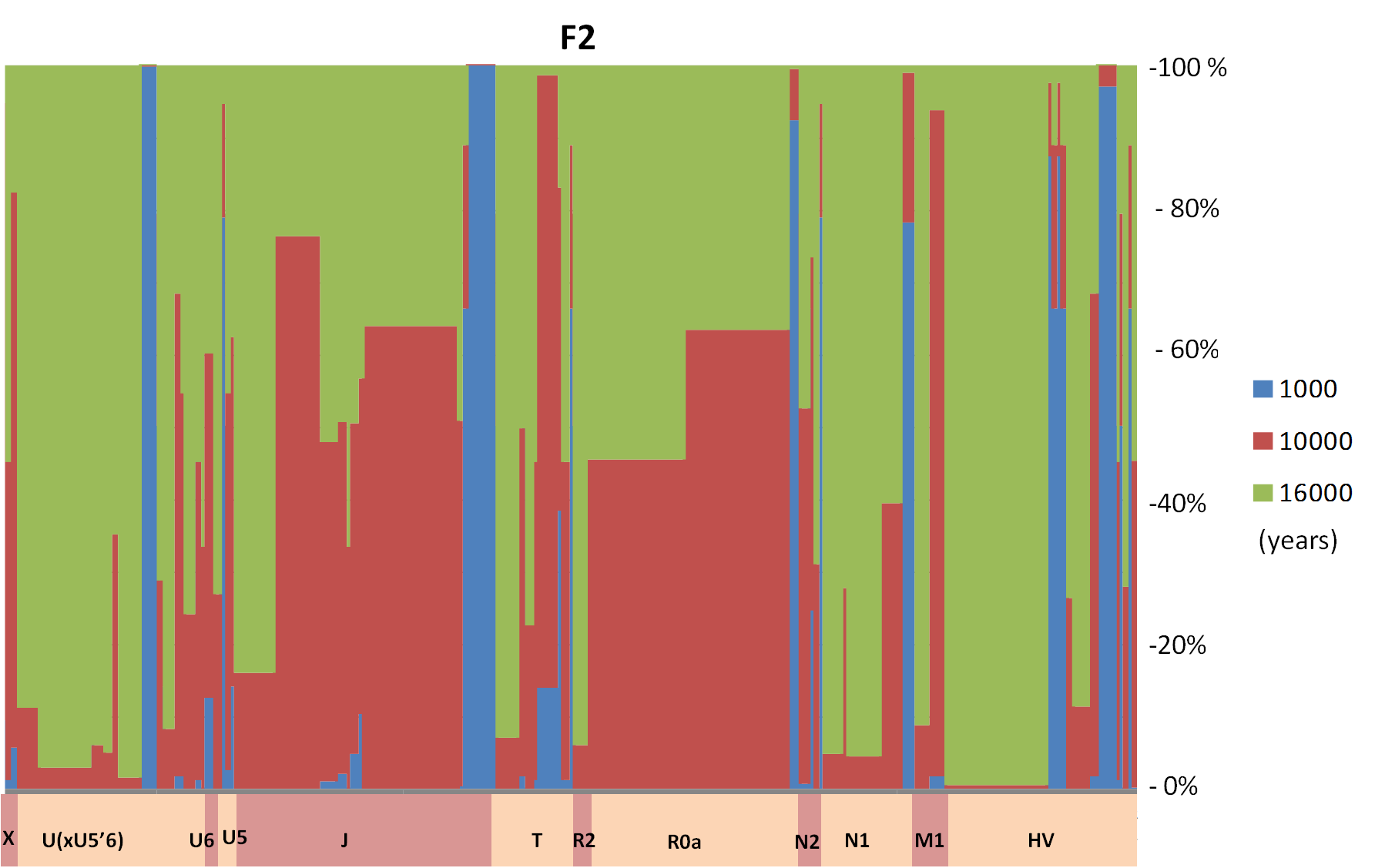

Supplement: S25 Fig — The haplogroup affiliations of the founders are indicated in the bottom. (TIF) [file pone.0118625.s025.tif]

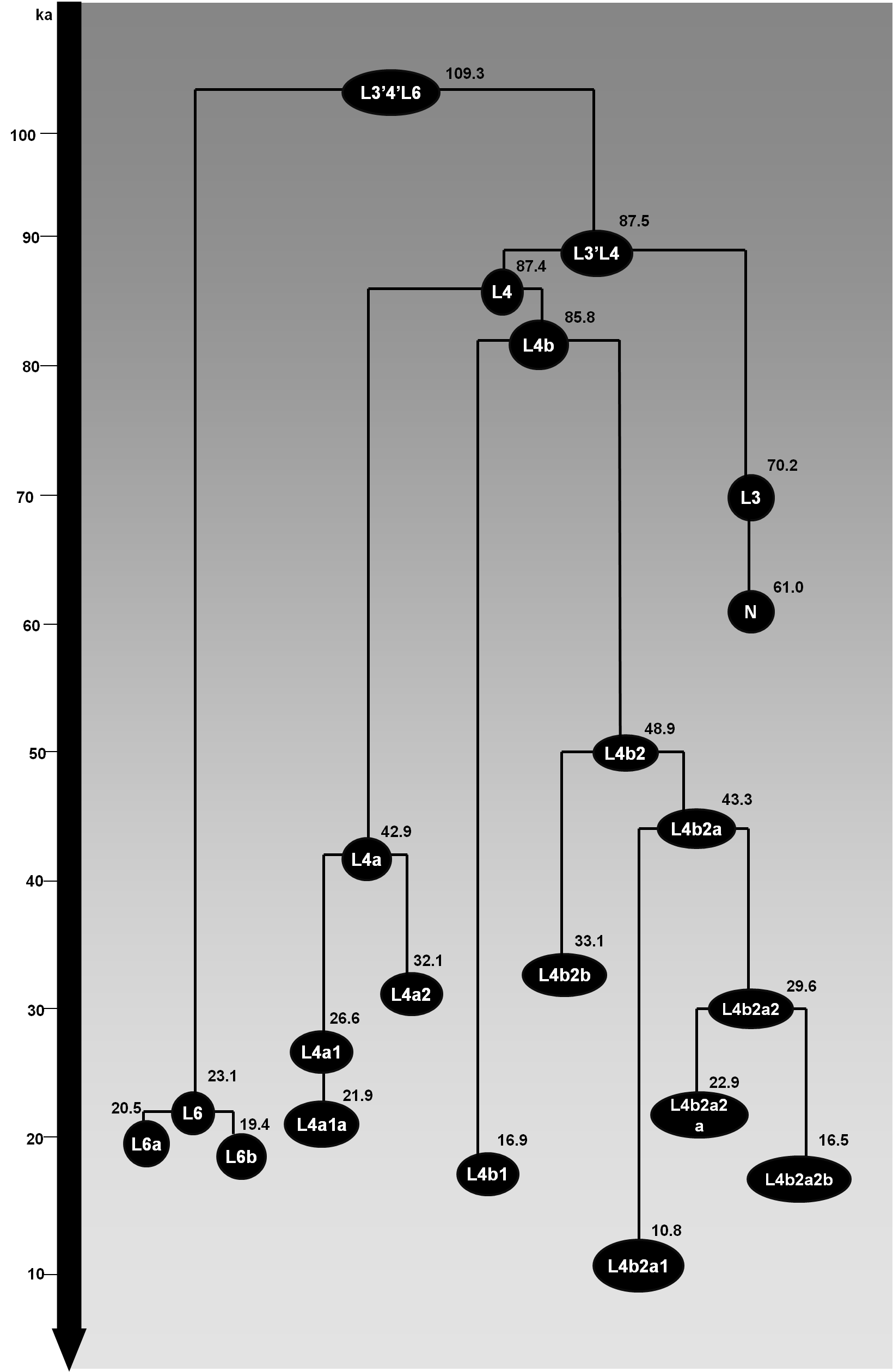

Supplement: S26 Fig — Ages (in ka) indicated are maximum likelihood estimates obtained with the whole-mtDNA genome. (TIF) [file pone.0118625.s026.tif]

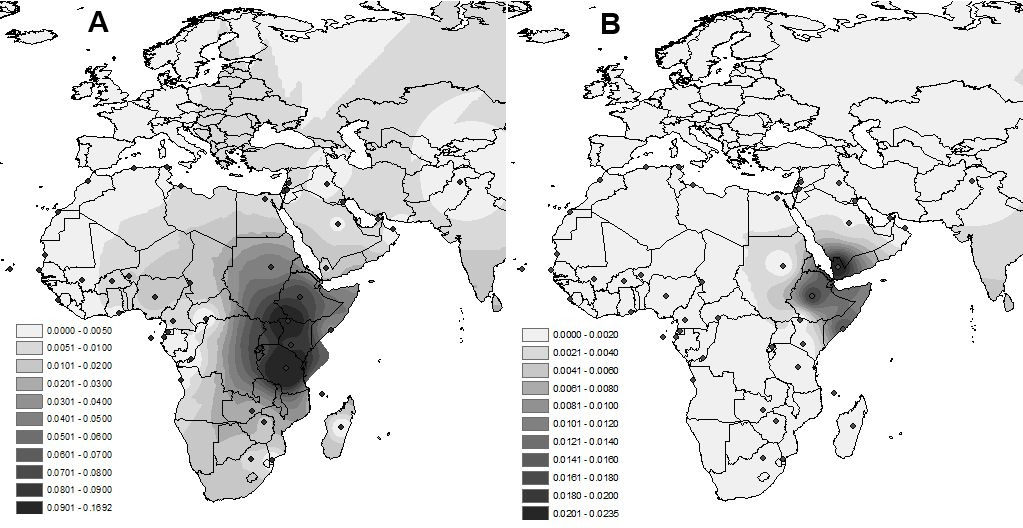

Supplement: S27 Fig — (TIF) [file pone.0118625.s027.tif]

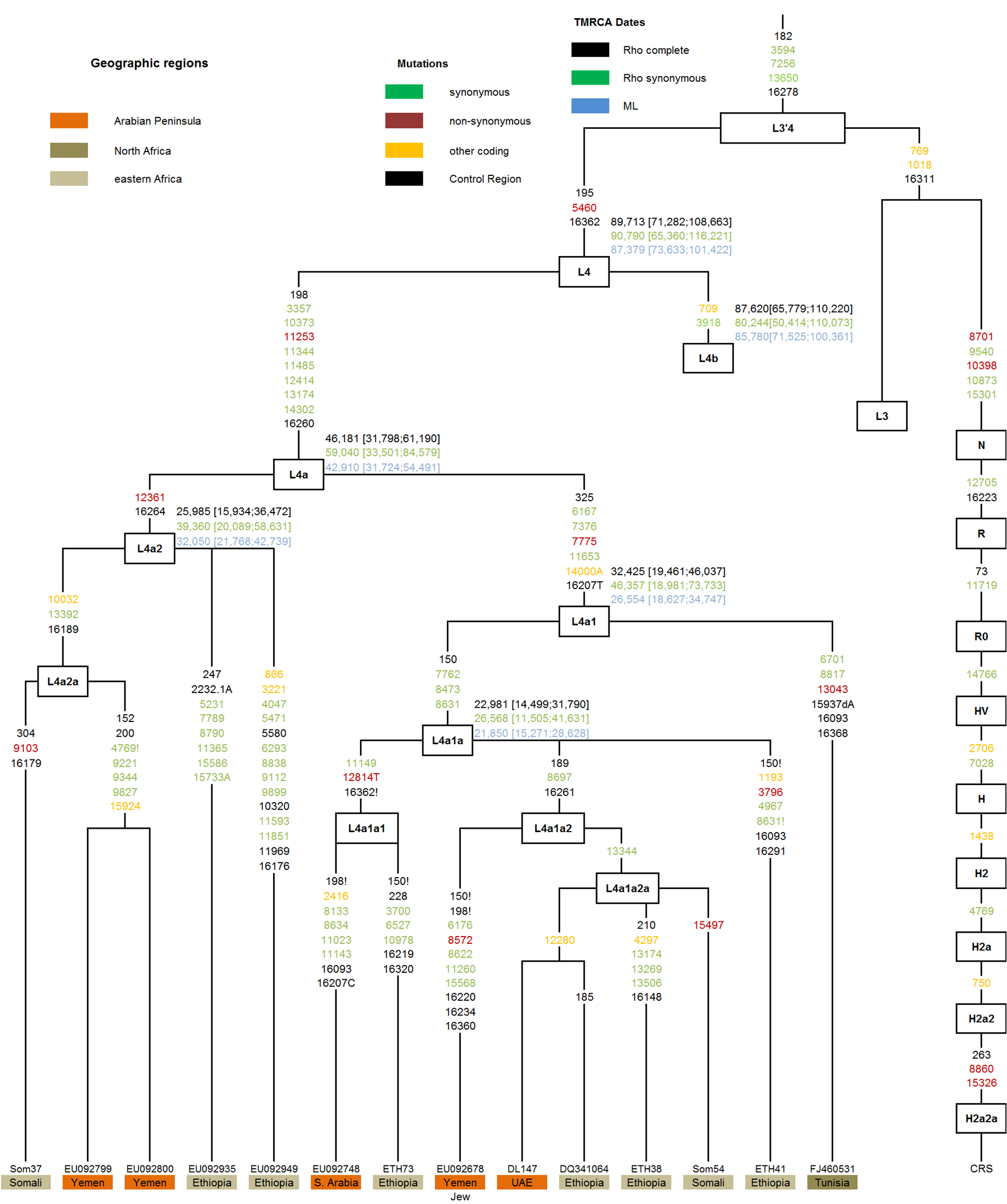

Supplement: S28 Fig — Labels on the branches represent nucleotide positions of transitions, and transversions when followed by a suffix “A,” “G,” “C,” or “T”; reversions by “!”; green indicates synonymous, brown non-synonymous, yellow other coding region, and black control region substitutions. Individual identification is indicated as well as the geographic origin when known (geographic regions are grouped by colour code according to the key). Near the nodes, the TMRCA is indicated (mean and 95% confidence interval) for ρ based on whole-mtDNA sequences (in black), ρ based on synonymous diversity (in green) and for maximum likelihood (in blue). (TIF) [file pone.0118625.s028.tif]

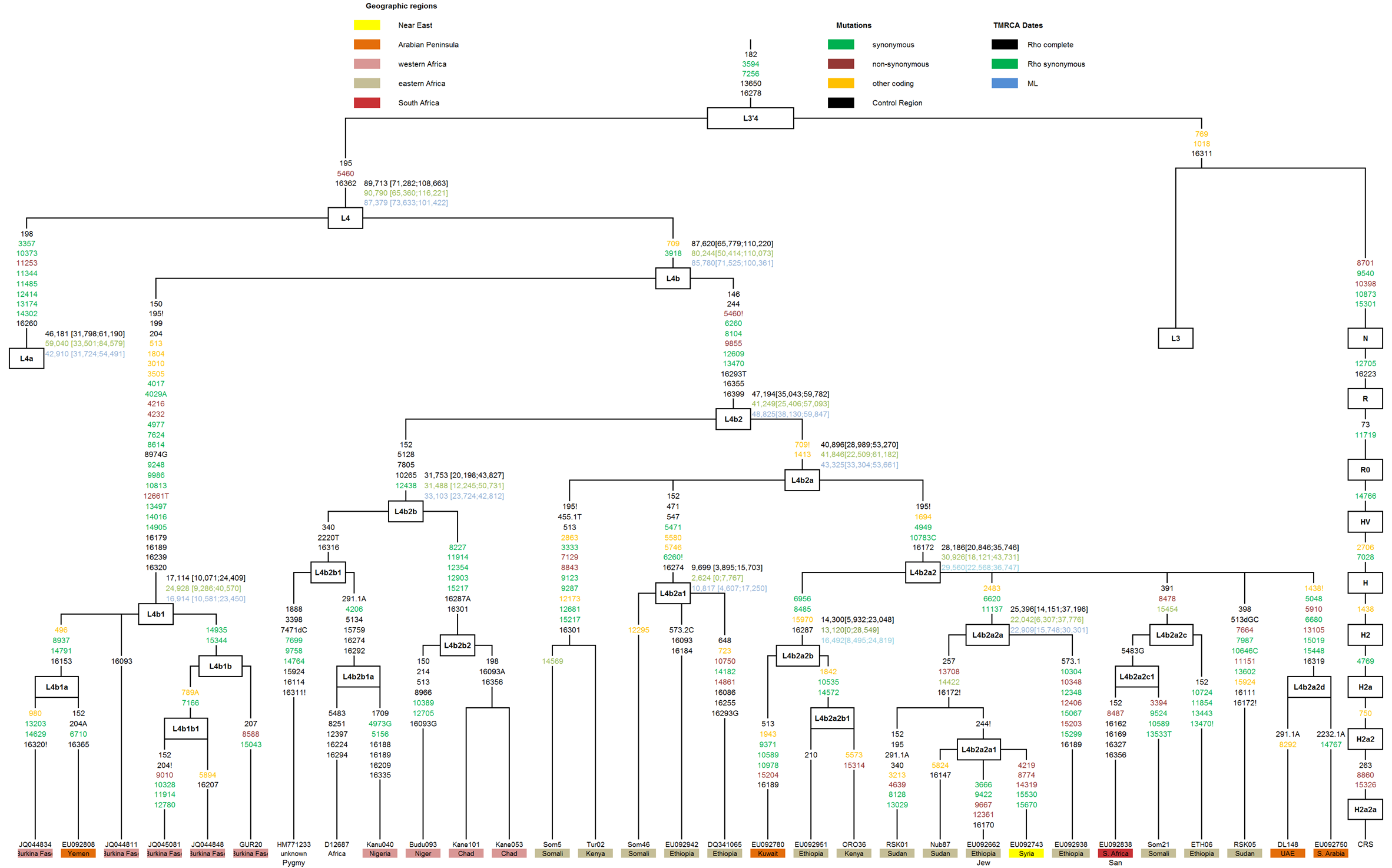

Supplement: S29 Fig — Labels on the branches represent nucleotide positions of transitions, and transversions when followed by a suffix “A,” “G,” “C,” or “T”; reversions by “!”; green indicates synonymous, brown non-synonymous, yellow other coding region, and black control region substitutions. Individual identification is indicated as well as the geographic origin when known (geographic regions are grouped by colour code according to the key). Near the nodes, the TMRCA is indicated (mean and 95% confidence interval) for ρ based on whole-mtDNA sequences (in black), ρ based on synonymous diversity (in green) and for maximum likelihood (in blue). (TIF) [file pone.0118625.s029.tif]

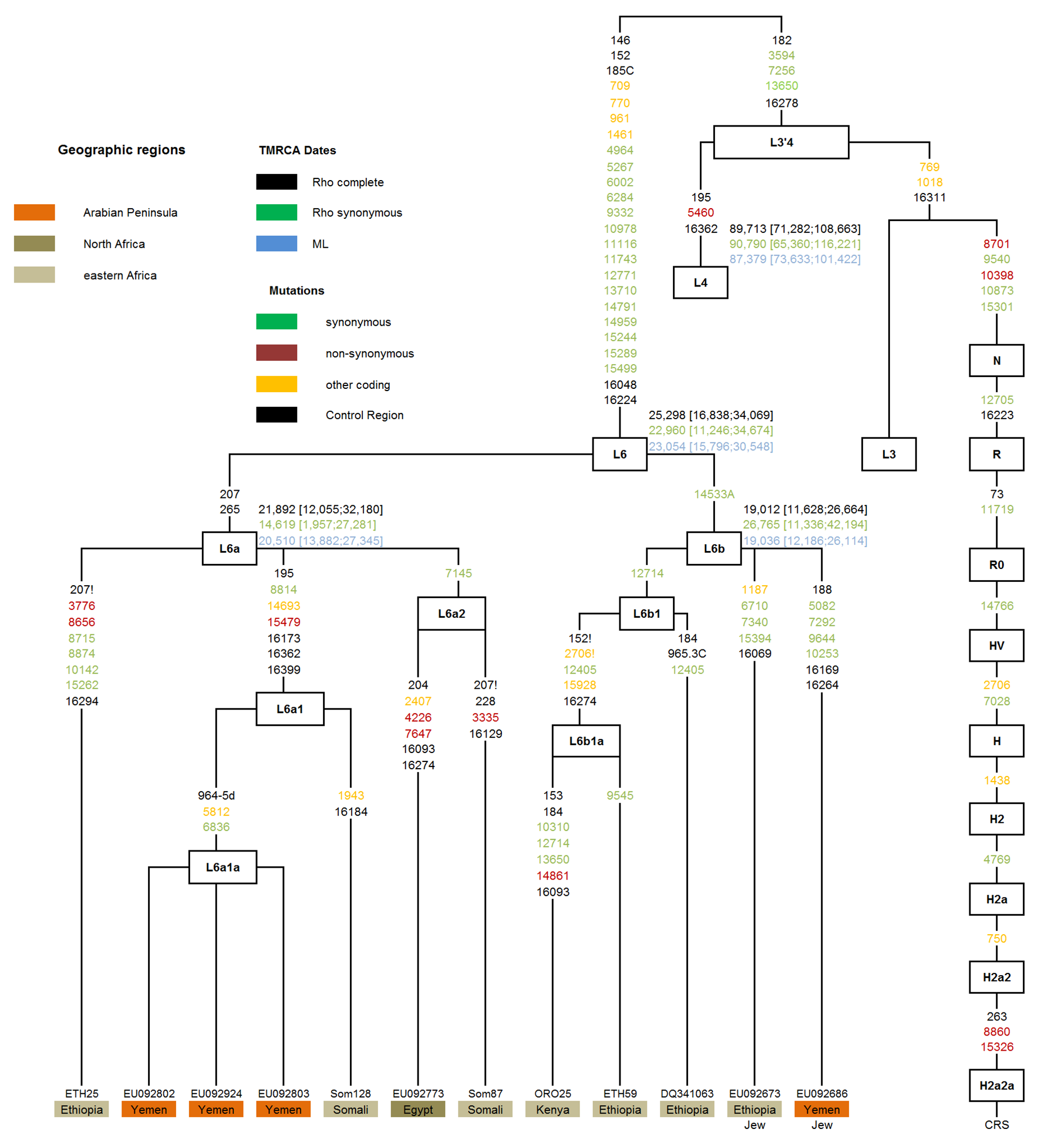

Supplement: S30 Fig — Labels on the branches represent nucleotide positions of transitions, and transversions when followed by a suffix “A,” “G,” “C,” or “T”; reversions by “!”; green indicates synonymous, brown non-synonymous, yellow other coding region, and black control region substitutions. Individual identification is indicated as well as the geographic origin when known (geographic regions are grouped by colour code according to the key). Near the nodes, the TMRCA is indicated (mean and 95% confidence interval) for ρ based on whole-mtDNA sequences (in black), ρ based on synonymous diversity (in green) and for maximum likelihood (in blue). (TIF) [file pone.0118625.s030.tif]

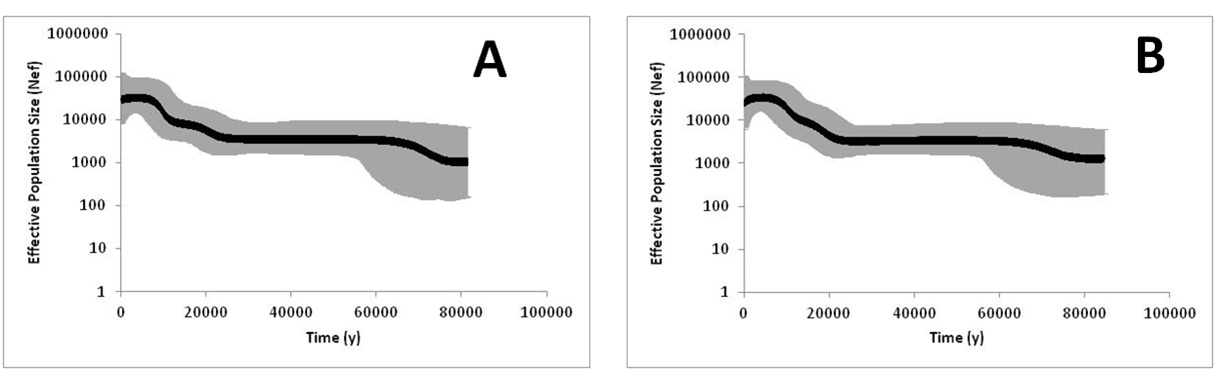

Supplement: S31 Fig — (TIF) [file pone.0118625.s031.tif]

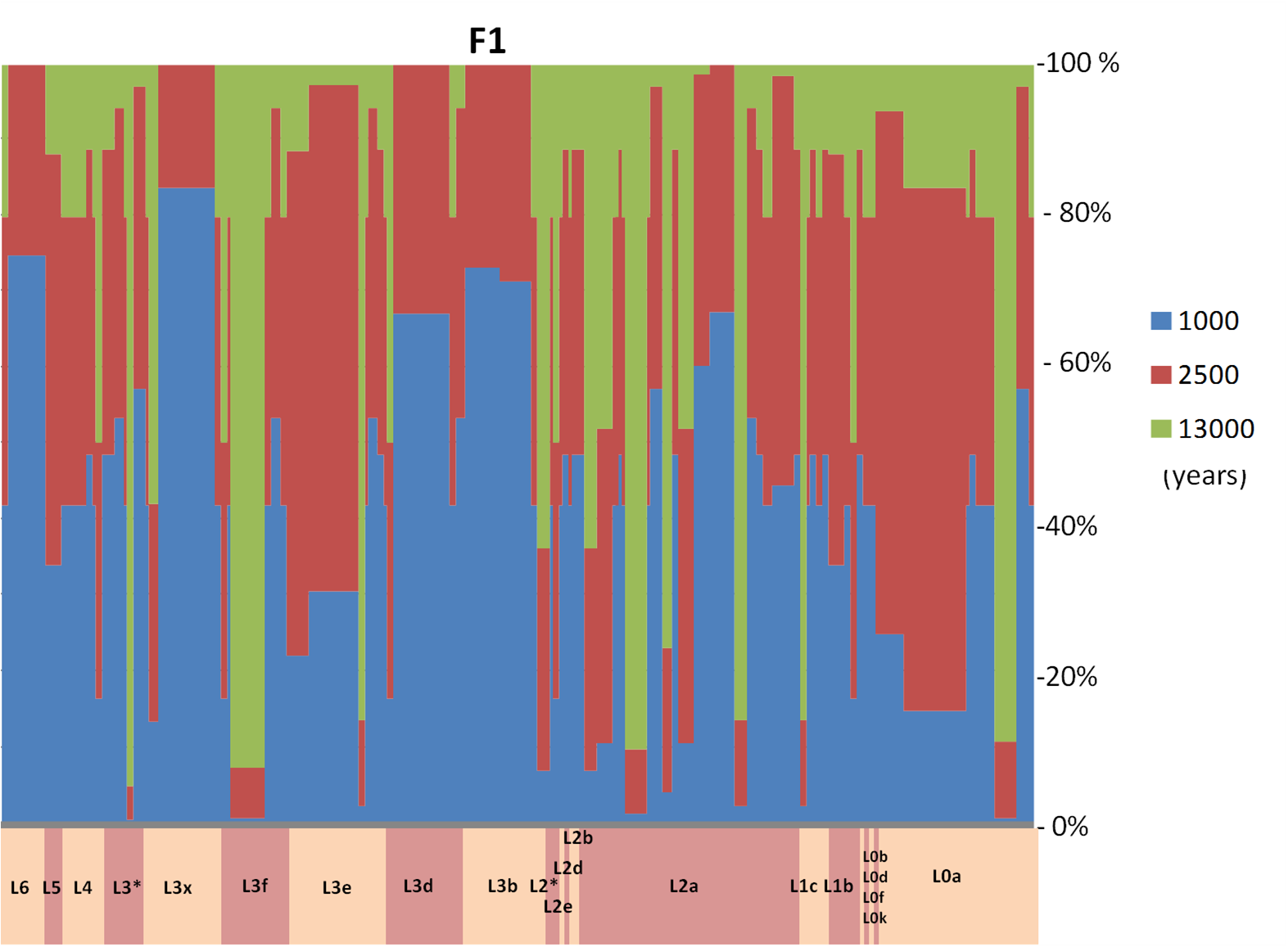

Supplement: S32 Fig — The haplogroup affiliations of the founders are indicated in the bottom. (TIF) [file pone.0118625.s032.tif]

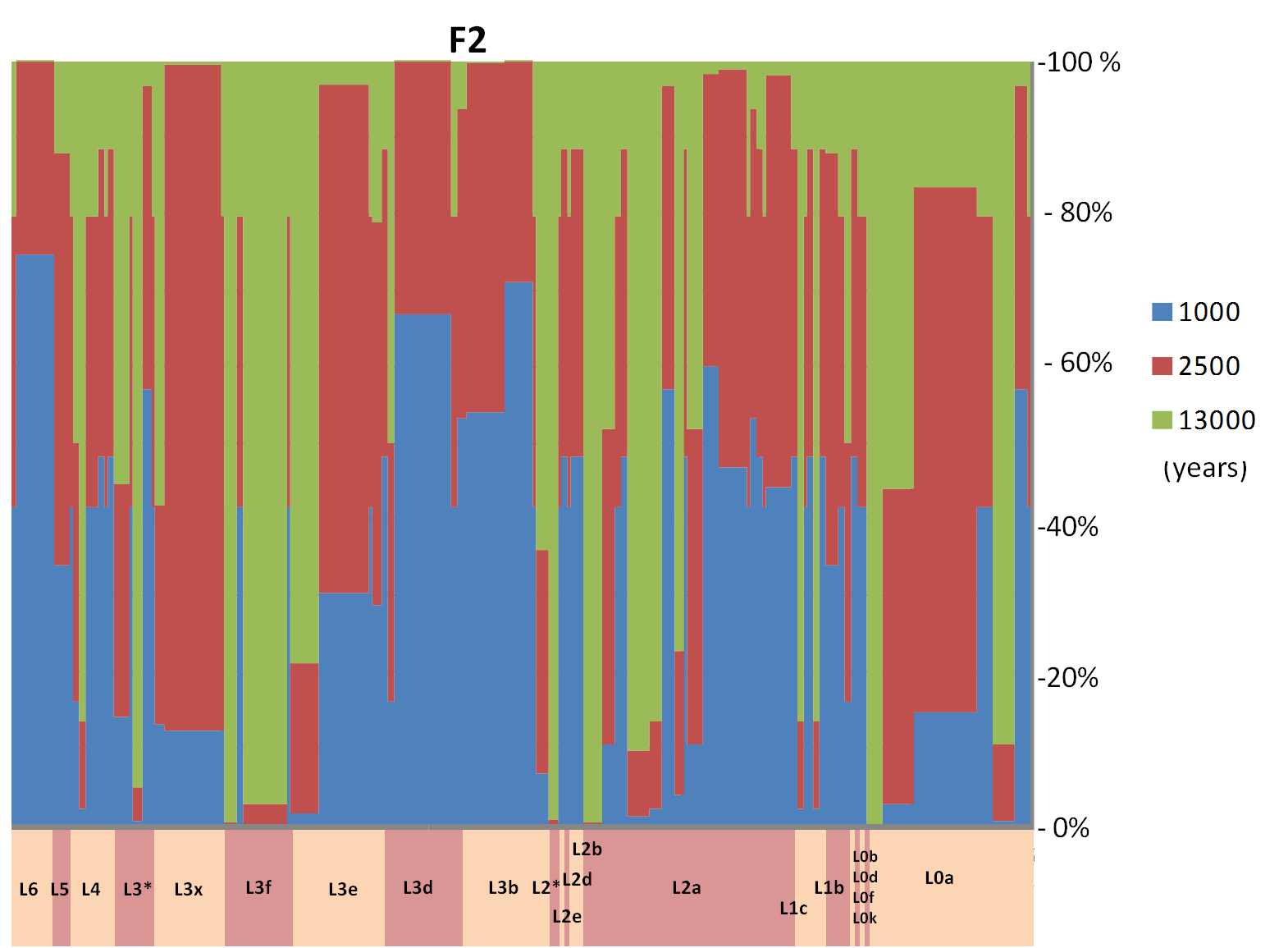

Supplement: S33 Fig — The haplogroup affiliations of the founders are indicated in the bottom. (TIF) [file pone.0118625.s033.tif]

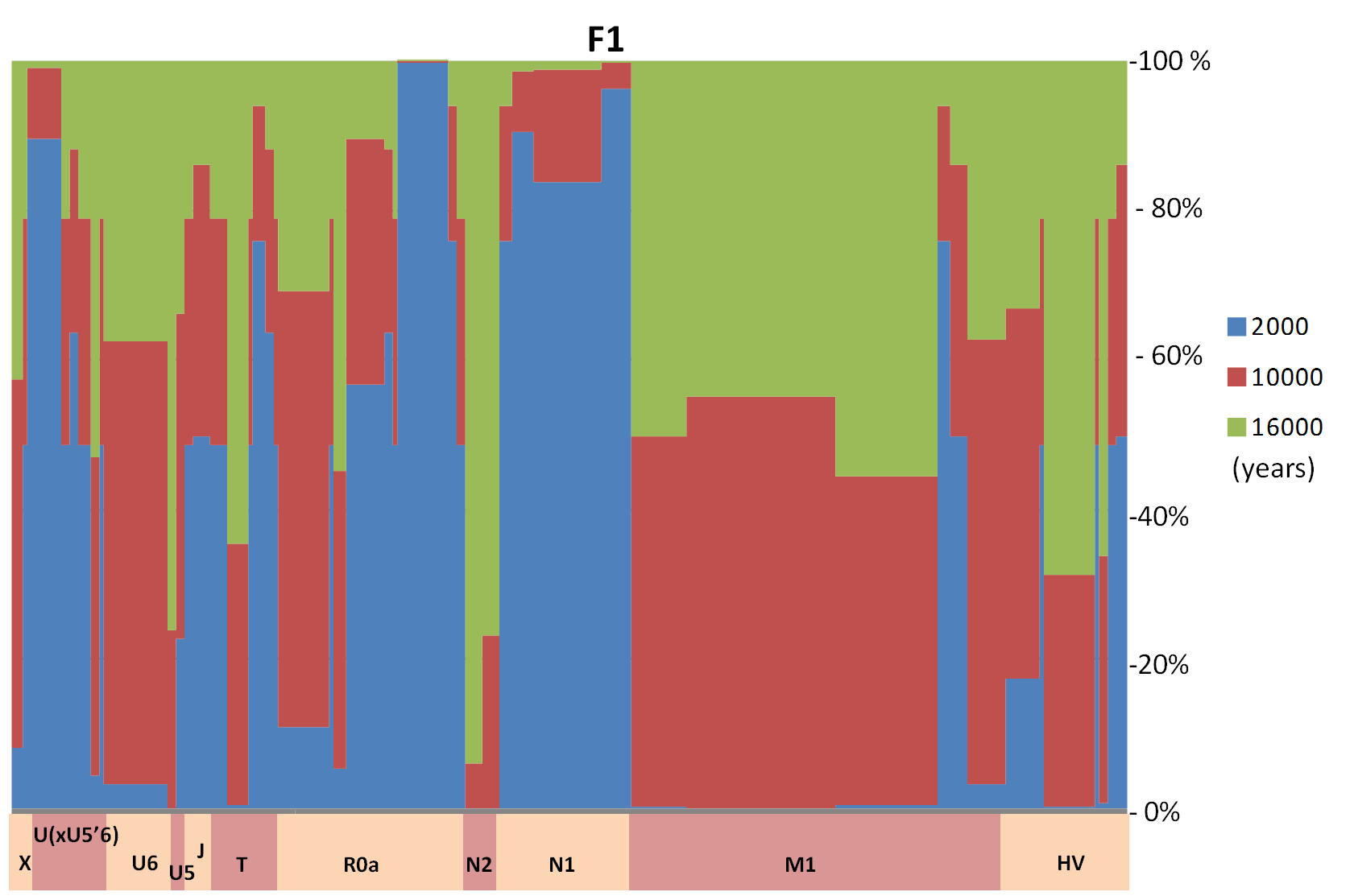

Supplement: S34 Fig — The haplogroup affiliations of the founders are indicated in the bottom. (TIF) [file pone.0118625.s034.tif]

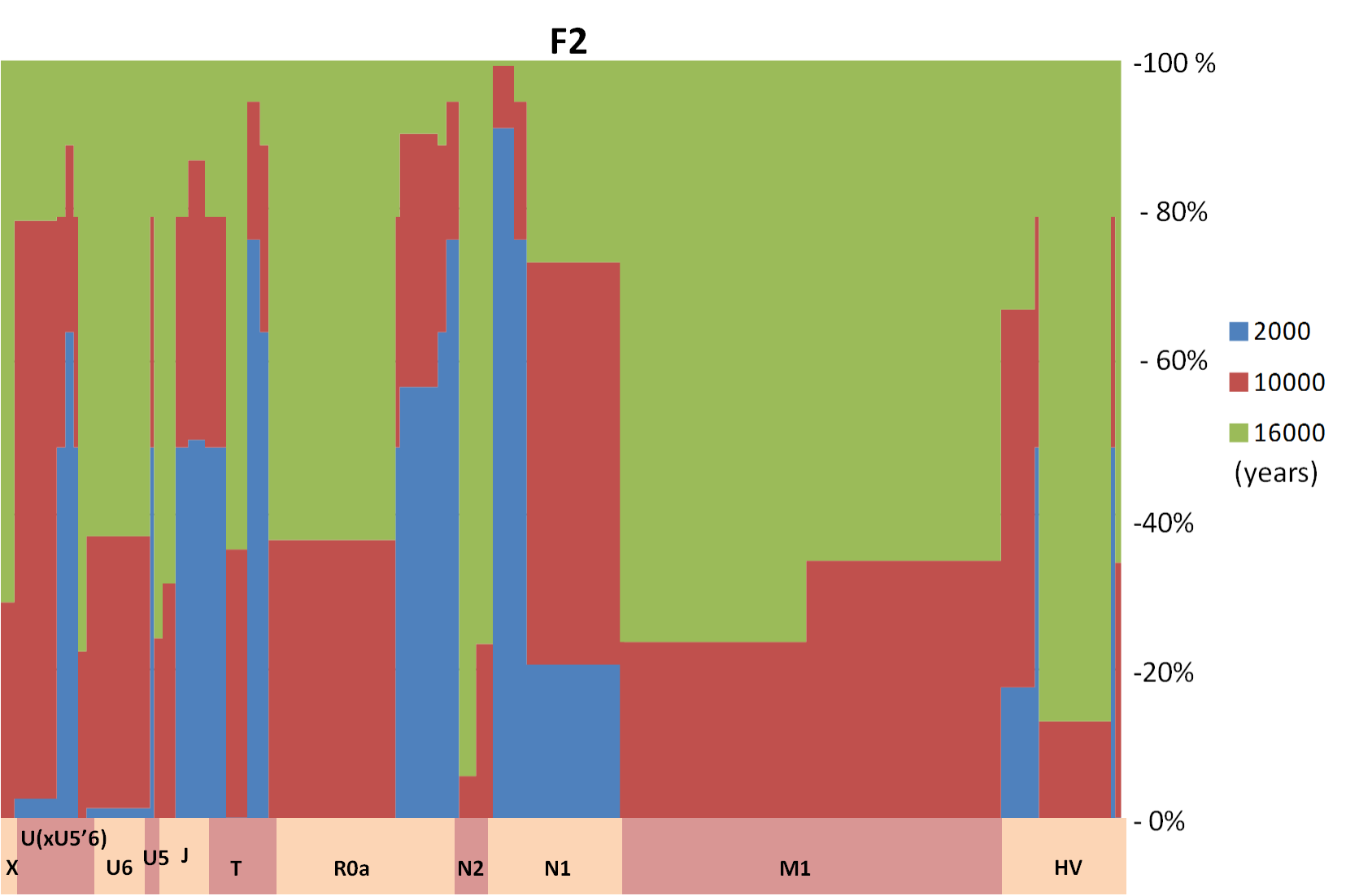

Supplement: S35 Fig — The haplogroup affiliations of the founders are indicated in the bottom. (TIF) [file pone.0118625.s035.tif]

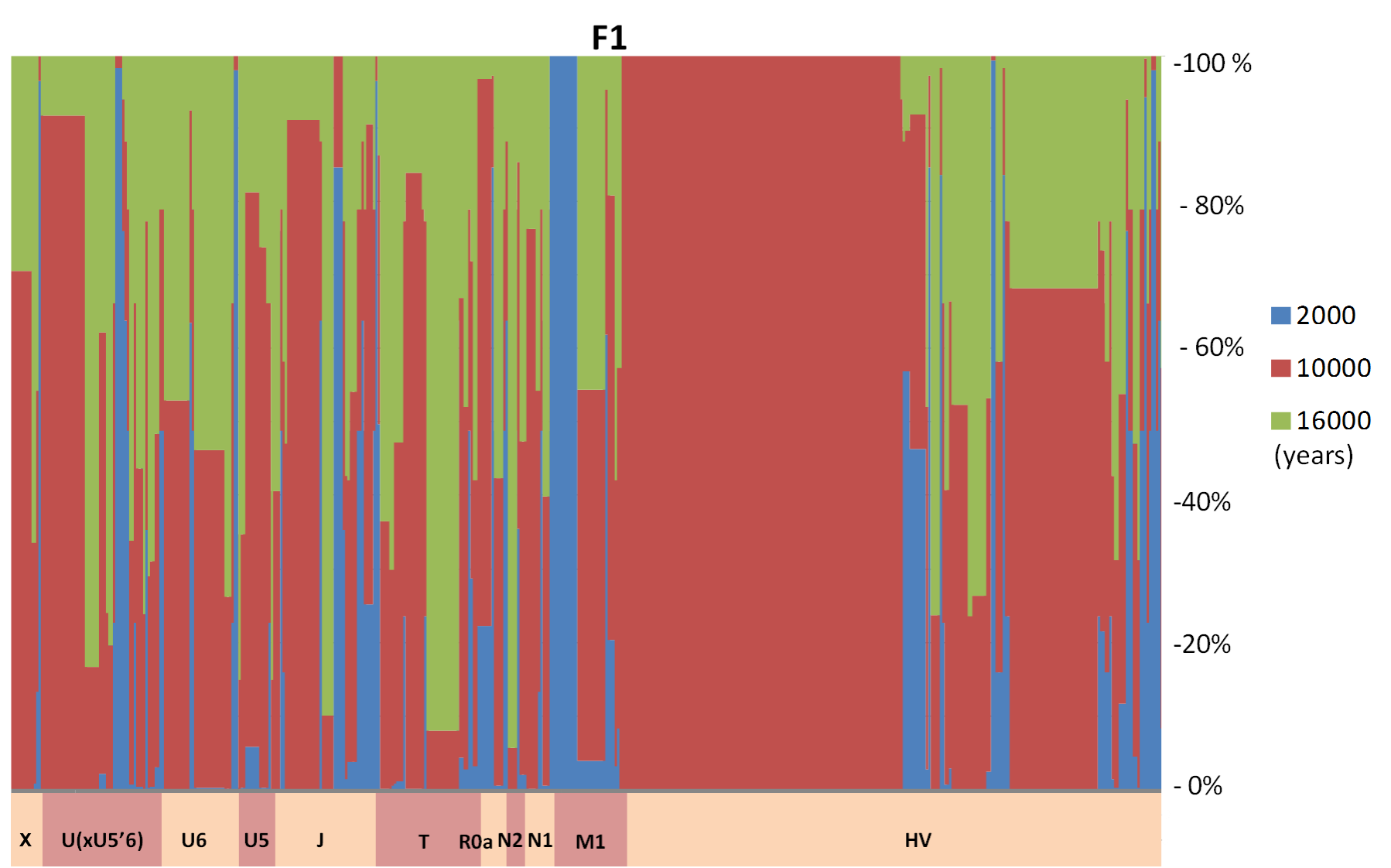

Supplement: S36 Fig — The haplogroup affiliations of the founders are indicated in the bottom. (TIF) [file pone.0118625.s036.tif]

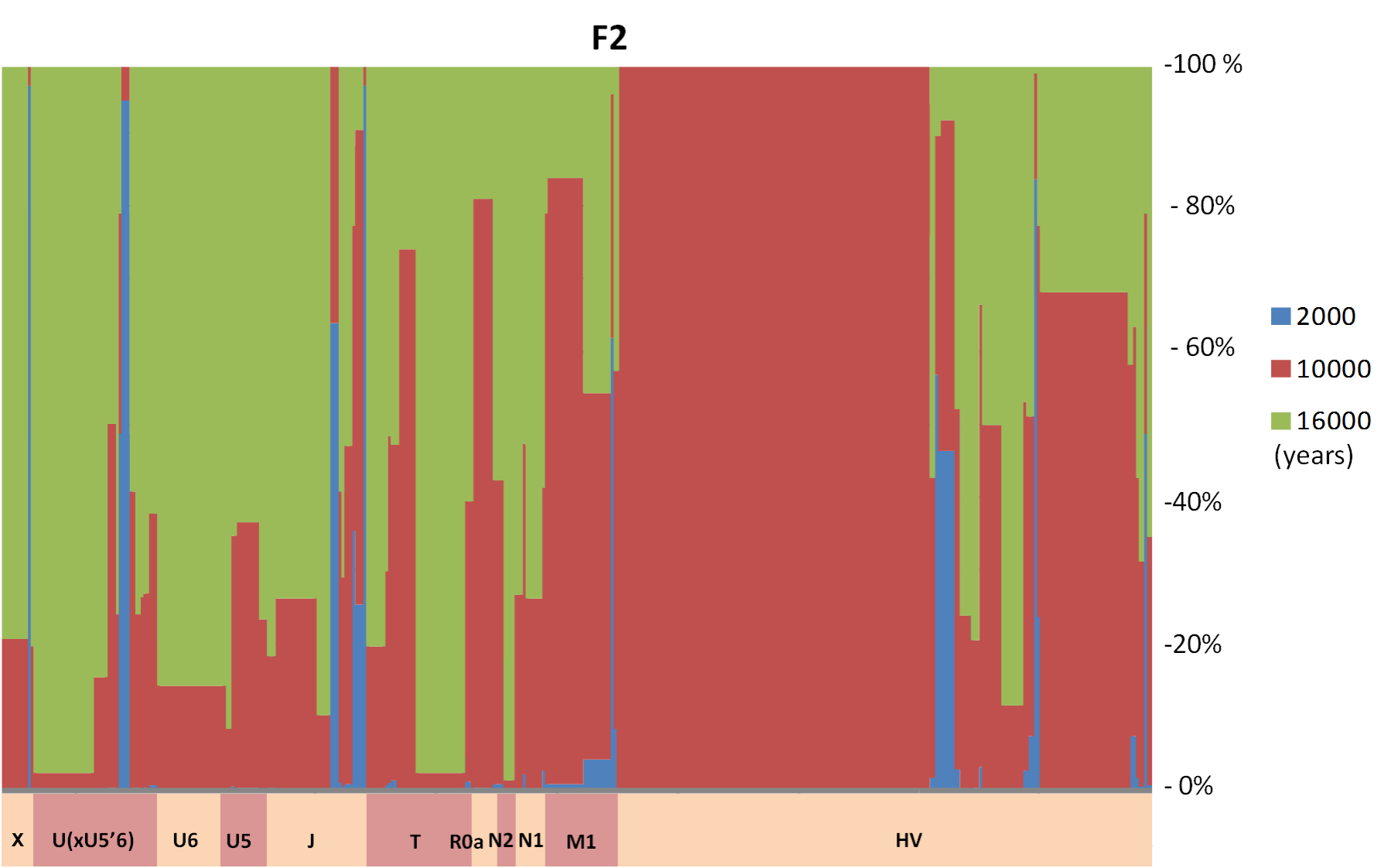

Supplement: S37 Fig — The haplogroup affiliations of the founders are indicated in the bottom. (TIF) [file pone.0118625.s037.tif]

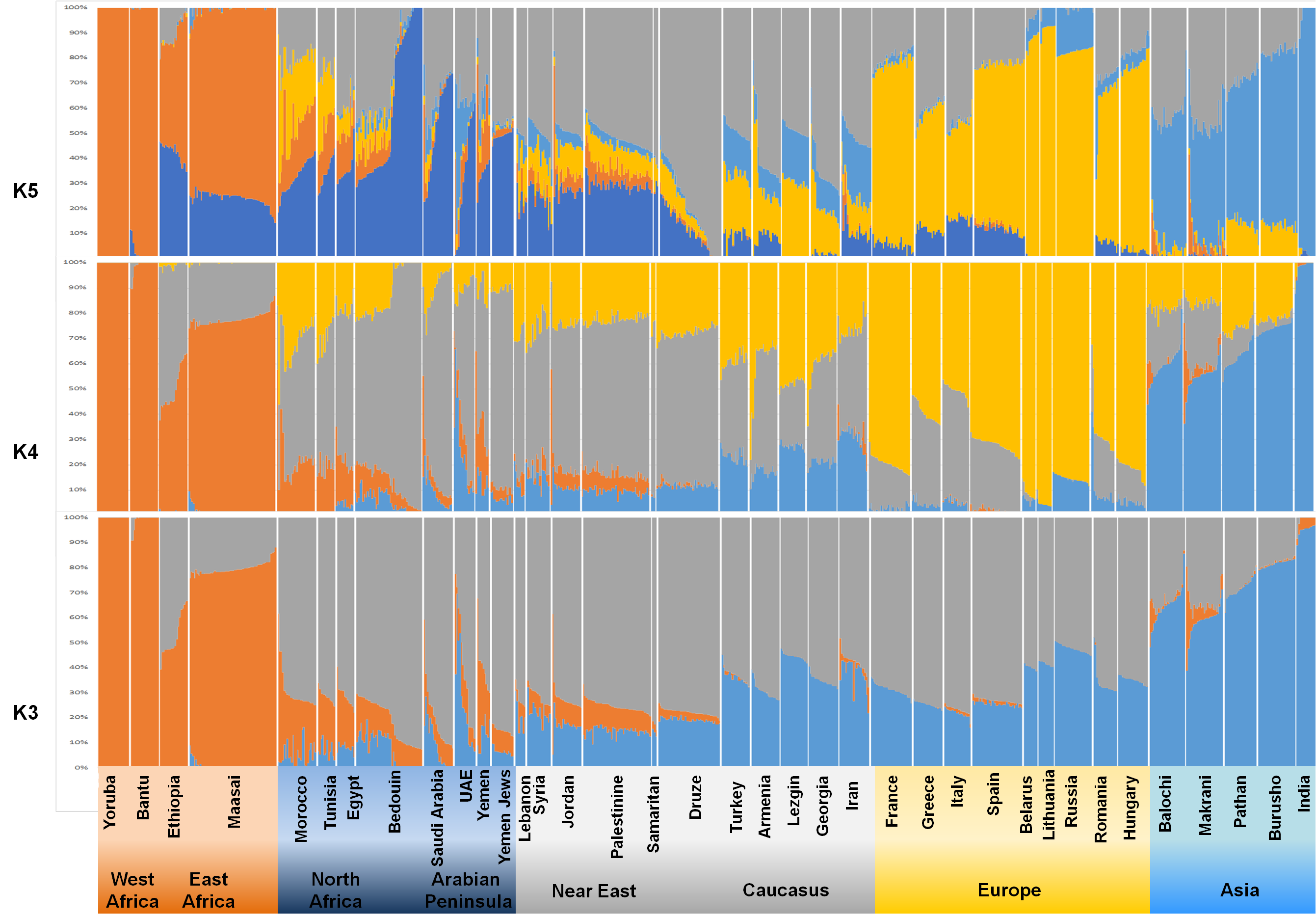

Supplement: S38 Fig — Each individual is represented by a vertical (100%) stacked column of genetic components proportions shown in colour for K = 3, 4 and 5. (TIF) [file pone.0118625.s038.tif]
