# Supplementary material for: Genetic Stratigraphy of Key Demographic Events in Arabia
Source: PLoS One. 2015 Mar 4;10(3):e0118625. doi: 10.1371/journal.pone.0118625 (PMC4349752; doi:10.1371/journal.pone.0118625)
Supplement: S1 Table — (DOCX) [file pone.0118625.s039.docx]

**S1_Table** Mitochondrial haplotypes for the whole-mtDNA sequences that were fully characterised in this study and the corresponding geographic region

| **Sample ID** | **GenBank accession number** | **Geographic region** | **Haplogroup** | **Haplotype (variants from rCRS; transitions unless specified)** |
| --- | --- | --- | --- | --- |
| JAC-1 | KP316996 | Yemen | J1b1b1 | 73 263 271 295 309.1C 315.1C 462 489 523d 750 1438 2706 3010 4216 4769 5460 7028 8860 8269 10398 10410A 11251 11719 12246 12612 13708 13879 14766 15326 15452A 16069 16126 16145 16261 16399 16519 |
| JAC-4 | KP316997 | Yemen | J1b3 | 73 146 263 295 309.1C 315.1C 462 489 750 1438 2706 3010 4216 4769 7028 8231 8269 8460 8642 8659 8860 10398 11251 11710 11719 12612 13708 13782 14766 15326 15452A 16069 16126 16145 16222 16261 16362 |
| JAC-7 | KP316998 | Yemen | J1d1a1 | 73 152 263 295 315.1C 370 462 489 750 1007 1438 2706 3010 4216 4769 5147 6546 7028 7789 7963 8860 9380 10398 11251 11719 12612 13392 13708 14766 15326 15452A 16069 16086 16126 16193 16309 |
| JAC-9 | KP316999 | Yemen | T2a1a | 73 263 309.1C 315.1C 709 750 1438 1719 1888 2706 2850 4216 4721 4769 4917 7022 7028 8251 8697 8860 10463 11251 11719 11812 13359 13368 13965 14233 14687 14766 14905 15326 15452A 15607 15928 16093 16126 16183C 16189 16294 16296 16519 |
| JAC-12 | KP317000 | Yemen | T2a1a | 73 263 309.2C 709 750 1438 1719 1888 2706 2850 4216 4721 4769 4917 7022 7028 8251 8697 8860 9055 10463 11251 11719 11812 13359 13368 13965 14233 14687 14766 14905 15326 15452A 15607 15928 16126 16294 16296 16519 |
| JAC-30 | KP317001 | Yemen | T2g1a1a | 73 200 263 315.1C 709 750 789 1438 1888 1977 2706 3834 4216 4769 4917 7028 8697 8860 10463 10576 11251 11719 11812 13368 14233 14524 14766 14798 14839 14905 15326 15452A 15607 15928 16114A 16126 16294 16519 |
| JTI-57 | KP317002 | Yemen | T2c1b2 | 73 146 152 263 309.1C 315.1C 523d 709 750 1438 1888 2380 2706 3221 4216 4769 4917 6261 6521 7028 8697 8860 10289 10398 10463 10822 11251 11719 11812 13368 14233 14766 14905 15326 15452A 15607 15928 16126 16288 16292 16294 16296 16311 |
| JTI-74 | KP317003 | Yemen | J1d1a1 | 73 152 263 295 315.1C 462 489 750 1007 1438 2706 3010 4216 4703 4769 7028 7202 7789 7963 8131 8860 9380 10398 11251 11719 12612 13392 13708 14766 15326 15452A 16069 16126 16193 16309 |
| JTI-97 | KP317004 | Yemen | J1d1a | 73 152 263 295 315.1C 462 489 514d 516 750 1007 1438 2706 3010 4216 4769 7028 7789 7963 8622 8860 10398 11251 11719 12612 13708 14766 14996 15326 15452A 16069 16126 16193 16287 16300 16309 |
| JHA-134 | KP317005 | Yemen | T2e3 | 73 146 150 195 263 309.1C 315.1C 709 750 1438 1888 2706 4216 4769 4917 7028 8697 8860 9698 10463 11251 11719 11812 12477 13020 13368 13962 14233 14766 14905 15326 15452A 15607 15928 16126 16153 16233C 16257 16294 16325 16399 16519 |
| JSO-152 | KP317006 | Yemen | J2a2a1 | 73 150 195 235 263 295 309.1C 315.1C 489 750 1438 1888 2706 3316 4216 4769 6671 7028 7476 8386 8860 10398 10499 11251 11377 11719 12171 12570 12612 13708 14766 15257 15326 15452A 15679 16069 16126 16179 |
| JSO-155 | KP317007 | Yemen | T2i | 73 263 315.1C 709 750 1438 1888 2706 4216 4769 4917 7028 8155 8697 8860 9422C 10463 11251 11719 11812 13368 14233 14766 14905 15397 15452A 15607 15928 16126 16294 16296 16362 16519 |
| JSO-160 | KP317008 | Yemen | T2c1c | 73 263 315.1C 709 750 1438 1888 2706 3010 4216 4769 4823 4917 6261 7028 8697 8860 9177 10463 10822 11251 11719 11812 13368 14233 14319 14766 14905 15326 15452A 15607 15928 16126 16146 16183C 16189 16294 16296 16519 |
| JSO-208 | KP317009 | Yemen | J2a2 | 73 150 195 263 295 315.1C 489 709 750 1438 2706 4216 4769 6671 6908 7028 7476 7830 8860 9189 10398 10499 11002 11251 11377 11719 12570 12612 13708 14577 14766 15257 15326 15452A 15679 16069 16126 |
| JHG-222 | KP317010 | Yemen | J2a2a1a | 73 150 195 235 263 295 309.2C 315.1C 489 750 1438 2706 3316 4216 4769 6671 7028 7476 7585 8386 8860 10398 10499 11002 11251 11377 11719 12171 12570 12612 13708 14766 15257 15326 15452A 15679 16069 16126 16179 |
| JHG-234 | KP317011 | Yemen | J1d1a1 | 73 152 263 295 315.1C 462 489 750 1007 1438 2706 3010 4216 4769 7028 7789 7963 8269 8860 10398 11251 11719 12612 13392 13420 13708 14766 15326 15452A 16069 16126 16145 16193 16300 16309 |
| JHG-237 | KP317012 | Yemen | J1b2a | 73 263 295 315.1C 462 489 750 1438 1733 2706 3010 4216 4769 7028 8269 8860 8962 10398 11251 11719 12612 13708 14766 15326 15452A 15466 16069 16126 16136 16145 16218 16222 16261 |
| JHG-245 | KP317013 | Yemen | J2a2b1 | 73 150 185 189 195 263 295 309.1C 315.1C 489 522 750 1438 2706 4216 4769 6671 7028 7476 8712 8860 10398 10499 11002 11251 11377 11719 12570 12612 13708 14766 15257 15326 15452A 15679 16069 16126 16241 |
| YBA044 | KP317014 | Yemen | T1a | 73 114A 263 315.1C 709 750 1438 1888 2706 4216 4769 4917 5123 6060 7028 8697 8860 10253 10463 11251 11719 12633A 13368 14766 14905 15326 15452A 15607 15928 16093 16126 16163 16186 16189 16294 16300 16519 |
| YBA049 | KP317015 | Yemen | T1a | 73 114A 263 315.1C 709 750 1438 1888 2706 4216 4769 4917 5123 6060 7028 8697 8860 10253 10463 11251 11719 12633A 13368 14766 14905 15326 15452A 15607 15928 16126 16163 16185 16186 16189 16294 16300 16519 |
| YBA050 | KP317016 | Yemen | J2a2b | 73 150 195 204 263 295 315.1C 489 523iAC 750 1438 1676 2706 3337 4216 4769 5054 5291 6671 7028 7476 7741 8860 9323 10398 10499 11002 11251 11377 11719 12570 12612 12858 13708 14766 14947 15257 15326 15452A 15679 16069 16126 16145 16241 |
| YBA061 | KP317017 | Yemen | T2c1b2 | 73 146 152 263 309.1C 315.1C 523dAC 709 750 1438 1888 2380 2706 3221 4216 4769 4917 6261 6521 7028 8697 8860 10289 10463 10822 11251 11719 11812 13368 14233 14766 14905 15326 15452A 15607 15928 16126 16288 16292 16294 16296 16311 |
| YBA063 | KP317018 | Yemen | J1d1a1 | 73 152 263 295 309.2C 315.1C 462 489 750 1007 1438 2706 3010 4216 4769 7028 7789 7963 8860 10398 11251 11719 12612 13392 13708 13967 14766 15326 15452A 16069 16126 16193 16256 16300 16309 |
| YBA068 | KP317019 | Yemen | J1d1a1 | 73 152 263 295 315.1C 370 462 489 513 750 1007 1438 2706 3010 4216 4769 7028 7789 7963 8152 8860 9380 10398 11251 11719 12612 13392 13708 14766 15326 15452A 16069 16126 16193 16300 16309 |
| YBA072 | KP317020 | Yemen | J1d1a1 | 73 152 263 295 315.1C 370 462 489 513 750 1007 1438 2706 3010 4216 4769 7028 7789 7963 8152 8860 9380 10398 11251 11719 12612 13392 13708 14766 15326 15452A 16069 16126 16193 16300 16309 |
| DL70 | KP317021 | UAE | J1d2c2 | 73 152 263 295 315.1C 462 489 750 1438 2706 3010 4011 4216 4769 5262 7028 7521 7789 7963 8860 10398 11251 11719 12612 13708 14766 15326 15452A 16069 16126 16193 16519 |
| DL90 | KP317022 | UAE | J1b1b | 73 263 271 295 315.1C 462 489 750 1438 2706 3010 4216 4769 5460 7028 8269 8494 8860 10398 11251 11719 12612 13656 13708 13879 14766 15326 15452A 15941 16069 16126 16145 16222 16261 16519 |
| DL68 | KP317023 | UAE | J2a2b | 73 150 195 200 263 295 315.1C 489 750 1438 2483 2706 4216 4257 4769 6671 6749 7028 7476 8860 10398 10499 11002 11251 11377 11719 12570 12612 13708 14058 14766 15217 15257 15326 15452A 15679 16069 16126 16241 16278 |
| DL72 | KP317024 | UAE | J1d2c2 | 73 152 263 295 315.1C 462 489 750 1438 2281C 2706 3010 4011 4216 4769 5262 7028 7521 7789 7963 8860 10398 11251 11719 12612 13708 14766 15326 15452A 16069 16126 16193 16519 |
| DL73 | KP317025 | UAE | J1d2 | 73 152 263 295 309.1C 315.1C 462 489 750 1438 2706 3010 3523 4216 4769 7028 7789 7963 8473 8860 10398 11251 11719 12612 13708 14766 15326 15452A 16069 16114 16126 16193 16519 |
| DL76 | KP317026 | UAE | J1b | 73 151 152 263 295 315.1C 462 489 750 1438 2706 3010 4216 4354 4769 6962 7028 7080 7364 8110 8269 8860 10398 10873 11251 11719 12612 12757 13708 13933 14020 14766 15326 15452A 16069 16145 16189 16261 |
| DL77 | KP317027 | UAE | J1b | 73 151 152 263 295 309.1C 462 489 523d 750 1438 1555 2706 3010 4216 4354 4769 6962 7028 7364 8269 8860 8994 10398 10873 11251 11719 12612 13708 13933 14020 14353 14766 15326 15452A 15663 16069 16124 16145 16224 16261 16519 |
| DL79 | KP317028 | UAE | J1b | 73 151 152 263 295 309.1C 315.1C 462 489 750 1438 2706 3010 4216 4354 4769 6962 7028 7364 8269 8860 10398 10873 11251 11719 12612 13488 13708 13933 14020 14766 15326 15452A 16069 16145 16261 |
| DL82 | KP317029 | UAE | J1b2 | 73 183 263 295 315.1C 462 489 709 750 1438 1733 2706 3010 4216 4769 6340 7028 8269 8860 10398 11204 11251 11719 12406 12612 13708 14766 15326 15452A 16069 16093 16126 16145 16222 16261 16300 |
| DL80 | KP317030 | UAE | J1b | 73 151 152 263 295 309.1C 315.1C 462 489 750 1438 2706 3010 4216 4354 4769 6962 7028 7364 8269 8860 10398 10873 11251 11719 12172 12612 13708 13933 14020 14766 15326 15452A 15773 16069 16145 16261 |
| DL95 | KP317031 | UAE | J1b1a1 | 73 146 242 263 295 315.1C 462 489 523d 750 1438 2158 2706 3010 3840 4216 4769 5460 7028 8269 8557 8835 8860 10322 10398 11251 11719 11935 12007 12612 13708 13879 14766 15326 15452A 16069 16145 16172 16261 |
| DL200 | KP317032 | UAE | T1a6 | 73 263 315.1C 523d 709 750 1438 1888 2706 3867 4216 4769 4917 7028 8697 8860 10376 10463 11251 11719 12633A 13368 14766 14905 15326 15452A 15607 15928 16126 16163 16186 16189 16294 16519 |
| DL67 | KP317033 | UAE | J2a2b | 73 150 195 200 263 295 315.1C 489 750 1438 2483 2706 4216 4257 4769 6671 6749 7028 7476 8860 10398 10499 11002 11251 11377 11719 12570 12612 13708 14058 14766 15217 15257 15326 15452A 15679 15924 16069 16126 16241 16278 |
| DL93 | KP317034 | UAE | J1b2 | 73 152 263 295 309.1C 315.1C 462 489 750 1438 1733 2706 3010 4216 4769 7028 8269 8860 10398 11251 11719 12612 13708 14766 15326 15452A 16069 16126 16145 16222 16261 16519 |
| DL201 | KP317035 | UAE | T1a1a1 | 73 152 195 207 263 309.1C 315.1C 709 750 1438 1888 2706 4065 4216 4769 4917 7028 8697 8860 9899 10463 11251 11719 12633A 13368 14766 14905 15326 15452A 15607 15928 16126 16163 16186 16189 16294 16519 |
| DL204 | KP317036 | UAE | T2c1b2 | 73 146 263 309.1C 315.1C 523d 709 750 1438 1888 2706 4216 4769 4917 6261 7028 8697 8860 10289 10463 10822 11251 11719 11812 13368 14233 14766 14905 15326 15452A 15607 15928 16126 16189 16269 16292 16294 16296 16380 16519 |
| DL205 | KP317037 | UAE | T2b | 73 263 309.1C 315.1C 709 750 930 1438 1888 2706 3394 4216 4769 4890 4917 5147 6299 7028 8697 8860 10463 11251 11719 11812 13368 14233 14766 14905 15326 15452A 15607 15928 16126 16239 16256 16289 16294 16296 16304 16519 |
| DL75 | KP317038 | UAE | J1d | 73 263 271 295 309.1C 315.1C 462 489 523d 462 489 750 1438 2706 3010 3474 3483 4216 4769 7028 7789 7963 8860 10398 10410A 11251 11719 12612 13708 14766 15326 15452A 16069 16114 16126 16193 |
| DL198 | KP317039 | UAE | T1a3 | 73 151 152 263 315.1C 709 750 1192 1438 1888 2706 4216 4769 4917 6152 7028 8697 8860 10463 10867 11251 11719 12633A 13368 14766 14905 15326 15412 15452A 15607 15928 16126 16163 16186 16189 16294 |
| DUB25 | KP317040 | UAE | J1b2 | 73 152 263 295 315.1 462 489 750 1438 1733 2706 3010 4216 4769 5009 7028 7521 8269 8860 10398 11251 11719 13708 13768 14766 15326 15452A 16069 16126 16145 16222 16261 |
| DUB22 | KP317041 | UAE | J2b1 | 73 150 152 263 295 315.1 489 523d 524d 750 1438 2706 4216 4769 5633 7028 7476 8860 9593 10172 10398 11251 11719 12612 13708 14587 14766 15326 15452A 15812 16069 16126 |
| DUB34 | KP317042 | UAE | J1d2c2 | 73 152 263 295 315.1 462 489 750 794 1438 2706 3010 4011 4216 4769 5262 7028 7521 7789 7963 8860 10398 11251 11719 12612 13708 14766 15326 15452A 16069 16126 16193 16519 |
| DUB39 | KP317043 | UAE | T2 | 73 263 309.1 315.1 490 709 750 1438 1888 2706 4216 4769 4917 7028 7440G 8462 8697 8860 9175A 10463 11251 11719 11812 12223 13368 14233 14766 14905 15326 15452A 15607 15928 16126 16294 16296 16519 |
| DUB7 | KP317044 | UAE | J1b6b | 73 143 263 295 315.1 462 489 750 1438 2706 3010 4216 4769 5501 7028 8269 8860 9587 10398 11251 11719 12612 13708 14766 14769 15326 15452A 16069 16126 16145 16222 16261 16519 |
| DUB83 | KP317045 | UAE | J1b2 | 73 152 263 295 309.1 315.1 462 489 523d 524d 750 1438 1733 2706 3010 4216 4769 7028 7738 8269 8860 10398 11251 11719 12612 13708 14766 15326 15452A 16069 16093 16126 16145 16261 16311 |
| DUB84 | KP317046 | UAE | T1a4b | 73 263 315.1 709 750 1438 1888 2706 4216 4386 4769 4917 4991 7028 8697 8860 9438 10463 11251 11719 11864 12633A 13368 14323 14766 14905 15326 15452A 15607 15928 16126 16186 16189 16294 16519 |
| YJ-R1 | KP317047 | Yemen | J2a2a1a | 73 150 195 235 263 295 309.1 315.1 489 750 1438 2706 4216 4769 6671 7028 7476 8386 8725 8860 10398 10499 11002 11251 11377 11440 11719 12171 12570 12612 13368 13708 14766 15257 15326 15452A 15496 15679 16069 16126 16192 |
| YJ-R12 | KP317048 | Yemen | J1b2a | 73 93 263 295 315.1 462 489 750 1438 1733 2706 3010 4216 4769 7028 8269 8860 9120 10398 11251 11719 12612 13708 14766 15326 15452A 15466 16069 16126 16136 16145 16221 16261 16519 |
| YJ-R16 | KP317049 | Yemen | J2a2c1 | 73 150 195 263 295 309.1 315.1 489 750 1438 2706 4216 4769 6671 7028 7476 8860 10346G 10398 10499 11002 11251 11377 11719 12570 12612 12672 13708 14766 15257 15326 15452A 15679 16069 16126 16214 16231 16519 |
| YJ-R2 | KP317050 | Yemen | J2a2a1a | 73 150 195 235 263 295 309.1 315.1 489 750 1438 2706 4216 4769 6671 7028 7476 8386 8725 8860 10398 10499 11002 11251 11377 11440 11719 12171 12570 12612 13368 13708 14766 15257 15326 15452A 15496 15679 16069 16126 16192 |
| YJ-R22 | KP317051 | Yemen | J2a2a1a | 73 150 189 195 235 263 295 309.2 315.1 489 750 1438 2706 4216 4769 6671 7028 7325 7476 8386 8860 10398 10499 11002 11251 11377 11440 11719 12171 12570 12612 13708 13759 14766 15257 15326 15452A 15679 16069 16126 16311 |
| YJ-R25 | KP317052 | Yemen | J1b3b | 73 152 263 295 309.1 315.1 462 489 750 1438 2706 3010 4216 4769 6527 7028 8269 8460 8860 10398 10685 11251 11719 12612 13708 13962 14766 15172T 15326 15452A 15490 15530 16069 16126 16145 16222 16261 |
| Budu093 | KP317053 | Niger | L4b2b | 73 146 150 152 195 214 244 263 315.1C 513 709 750 769 1018 1438 2706 3918 4769 5128 6260 7028 7805 8104 8227 8701 8860 8966 9540 9855 9855 10265 10389 10398 10873 11719 11914 12354 12438 12609 12705 12609 12705 12903 13470 14766 15217 15301 15326 16093G 16223 16287A 16293T 16301 16311 16355 16362 16399 |
| DL147 | KP317054 | UAE | L4a1a | 73 150 189 195 198 263 315.1C 325 750 769 1018 1438 2706 3357 4769 5460 6167 7028 7376 7762 7775 8473 8631 8697 8701 8860 9540 10373 10398 10873 11253 11344 11485 11653 11719 12280 12414 12705 13174 13344 14000A 14302 14766 15301 15326 16207T 16223 16260 16261 16311 16362 16519 |
| DL148 | KP317055 | UAE | L4b2a2 | 73 146 244 263 291.1A 315.1C 750 769 1018 1413 1694 2706 3918 4769 4949 5048 5910 6260 6680 7028 8104 8292 8701 8860 9540 9855 10398 10783C 10873 11719 12609 12705 13105 13470 14766 15019 15301 15326 15448 16172 16223 16293T 16311 16319 16355 16362 16399 |
| ETH06 | KP317056 | Ethiopia | L4b2a2 | 73 146 152 244 263 391 315.1C 750 769 1018 1413 1438 1694 2706 3918 4769 4949 6260 7028 8104 8478 8701 8860 9540 9855 10398 10724 10783C 10873 11719 11854 12609 12705 13443 14766 15301 15326 15454 16172 16223 16293T 16311 16355 16362 16399 16519 |
| ETH25 | KP317057 | Ethiopia | L6a | 73 146 152 182 185C 263 265 309.1C 315.1C 709 750 769 770 961 1018 1438 1461 2706 3594 3776 4769 4964 5267 6002 6284 7028 7256 8656 8701 8715 8860 8874 9332 9540 10142 10398 10873 10978 11116 11719 11743 12705 12771 13650 13710 14766 14791 14959 15244 15262 15289 15301 15326 15499 16048 16223 16224 16278 16294 16311 16519 |
| ETH38 | KP317058 | Ethiopia | L4a1a | 73 150 189 195 198 210 263 309.1C 315.1C 325 750 769 1018 1438 2706 3357 4297 4769 5460 6167 7028 7376 7762 7775 8473 8631 8697 8701 8860 9540 10373 10398 10873 11253 11344 11485 11653 11719 12414 12705 13174 13269 13174 13269 13344 13506 14000A 14302 14766 15301 15326 16148 16207T 16223 16260 16261 16311 16362 16519 |
| ETH41 | KP317059 | Ethiopia | L4a1a | 73 195 198 263 315.1 325 750 769 1018 1193 1438 2706 3357 3796 4769 4967 5460 6167 7028 7376 7762 7775 8473 8701 8860 9540 10373 10398 10873 11253 11344 11485 11653 11719 12414 12705 13174 14000A 14302 14766 15301 15326 16093 16207T 16223 16260 16291 16311 16362 16519 |
| ETH59 | KP317060 | Ethiopia | L6b | 73 146 182 185C 263 309.1C 315.1C 709 750 769 770 961 1018 1438 1461 3594 4769 4964 5267 6002 6284 7028 7256 8701 8860 9332 9540 9545 10398 10873 10978 11116 11719 11743 12405 12705 12714 12771 13650 13710 14533A 14766 14791 14959 15244 15289 15301 15326 15499 15928 16048 16223 16224 16274 16278 16311 16519 |
| ETH73 | KP317061 | Ethiopia | L4a1a | 73 195 198 228 263 315.1C 325 750 769 1018 1438 2706 3357 3700 4769 5460 6167 6527 7028 7376 7762 7775 8473 8631 8701 8860 9540 10373 10398 10873 10978 11149 11253 11344 11485 11653 11719 12414 12705 12814T 13174 14000A 14302 14766 15301 15326 16207T 16219 16223 16260 16311 16320 16519 |
| GUR20 | KP317062 | Burkina Faso | L4b1 | 73 150 199 204 207 263 309.1C 315.1C 513 709 750 769 1018 1438 1804 2706 3010 3505 3918 4017 4029A 4216 4232 4769 4977 5460 7028 7624 8588 8614 8701 8860 8974G 9248 9540 9986 10398 10813 10873 11719 12661T 12705 13497 14016 14766 14905 14935 15043 15301 15326 15344 16179 16189C 16223 16239 16311 16320 16362 16519 |
| Kane101 | KP317063 | Chad | L4b2b | 73 146 152 195 198 244 263 315.1C 514dGC 709 750 769 1018 1438 2706 3918 4769 5128 6260 7028 7805 8104 8227 8701 8860 9540 9855 10265 10398 10873 11719 11914 12354 12438 12609 12705 12903 13470 14766 15217 15301 15326 16093A 16223 16287A 16293T 16301 16311 16355 16356 16362 16399 16519 |
| KANE53 | KP317064 | Chad | L4b2b | 73 146 152 195 198 244 263 315.1C 514dGC 709 750 769 1018 1438 2706 3918 4769 5128 6260 7028 7805 8104 8227 8701 8860 9540 9855 10265 10398 10873 11719 11914 12354 12438 12609 12705 12903 13470 14766 15217 15301 15326 16093A 16223 16287A 16293T 16301 16311 16355 16356 16362 16399 16519 |
| Kanu040 | KP317065 | Nigeria | L4b2b | 73 146 152 195 244 263 291.1A 315.1C 340 523dCA 709 750 769 1018 1438 1709 2220T 2706 3918 4206 4769 4973G 5128 5134 5156 6260 7028 7805 8104 8701 8860 9540 9855 10265 10398 10873 11719 12438 12609 12705 13470 14766 15301 15326 15759 16188 16189 16209 16223 16274 16292 16293T 16311 16316 16335 16355 16362 16399 16519 |
| NUB087 | KP317066 | Nubia | L4b2a2a | 73 146 257 263 315.1C 750 769 1018 1413 1438 1694 2483 2706 3918 4769 4949 5824 6260 6620 7028 8104 8701 8860 9540 9855 10398 10783C 10873 11137 11719 12609 12705 13470 13708 14422 14766 15301 15326 16147 16223 16293T 16311 16355 16362 16399 16519 |
| ORO25 | KP317067 | Kenya | L6b | 73 146 153 182 184 185C 263 315.1C 709 750 769 770 961 1018 1438 1461 3594 4769 4964 5267 6002 6284 7028 7256 8701 8860 9332 9540 10310 10398 10873 10978 11116 11719 11743 12405 12705 12714 12771 13650 13650 13710 14533A 14766 14791 14861 14959 15244 15289 15301 15326 15499 15928 16048 16093 16223 16224 16274 16278 16311 16519 |
| ORO36 | KP317068 | Kenya | L4b2a2b | 73 146 244 263 309.1C 315.1C 750 769 1018 1413 1438 1694 1842 2706 3918 4769 4949 5573 6260 6956 7028 8104 8485 8701 8860 9540 9855 10398 10535 10783C 10873 11719 12609 12705 13470 14572 14766 15301 15314 15326 15970 16172 16223 16287 16293T 16311 16355 16362 16399 |
| RSK_01 | KP317069 | Sudan | L4b2a2a | 73 146 152 195 244 263 257 291.1A 315.1C 340 750 769 1018 1413 1438 1694 2483 2706 3213 3918 4639 4769 4949 6260 6620 7028 8104 8128 8701 8860 9540 9855 10398 10783C 10873 11137 11719 12609 12705 13029 13470 13708 14422 14766 15301 15326 16223 16293T 16311 16355 16362 16399 |
| RSK_05 | KP317070 | Sudan | L4b2a2 | 73 146 244 263 309.1C 315.1C 398 513dGC 750 769 1018 1413 1438 1694 2706 3918 4769 4949 6260 7028 7664 7987 8104 8701 8860 9540 9855 10398 10646C 10783C 10873 11151 11719 12609 12705 13470 13602 14766 15301 15326 15924 16111 16223 16293T 16311 16355 16362 16399 |
| Som128 | KP317071 | Somalia | L6a | 73 146 152 182 185C 195 207 263 265 315.1C 709 750 769 770 961 1018 1438 1461 1943 2706 3594 4769 4964 5267 6002 6284 7028 7256 8701 8814 8860 9332 9540 10398 10873 10978 11116 11719 11743 12705 12771 13650 13710 14693 14766 14791 14959 15244 15289 15301 15326 15479 15499 16048 16173 16184 16223 16224 16278 16311 16362 16399 16519 |
| Som21 | KP317072 | Somalia | L4b2a2 | 73 146 244 263 315.1C 391 750 769 1018 1413 1438 1694 2706 3394 3918 4769 4949 5483G 6260 7028 8104 8478 8701 8860 9524 9540 9855 10398 10589 10783C 10873 11719 12609 12705 13470 13533T 14766 15301 15326 15454 16172 16223 16293T 16311 16355 16362 16399 16519 |
| Som37 | KP317073 | Somalia | L4a2 | 73 195 198 263 304 309.1C 523dCA 750 769 1018 1438 2706 3357 4769 5460 7028 8701 8860 9103 9540 10032 10373 10398 10873 11253 11344 11485 11719 12361 12414 12705 13174 13392 14302 14766 15301 15326 16179 16189 16223 16260 16264 16311 16362 16519 |
| Som46 | KP317074 | Somalia | L4b2a1 | 73 146 152 195 244 263 315.1C 471 547 750 769 1018 1413 1438 2706 3918 4769 5471 5580 5746 7028 8104 8701 8860 9540 9855 10398 10873 11719 12295 12609 12705 13470 14766 15301 15326 16223 16274 16293T 16311 16355 16362 16399 16519 |
| Som5 | KP317075 | Somalia | L4b2a | 73 146 244 263 315.1C 455.1T 513 750 769 1018 1413 1438 2706 2863 3333 3918 4769 6260 7028 7129 8104 8701 8843 8860 9123 9287 9540 9855 10398 10873 11719 12173 12609 12681 12705 13470 14569 14766 15217 15301 15326 16301 16223 16293T 16311 16355 16362 16399 16519 |
| Som54 | KP317076 | Somalia | L4a1a | 73 150 189 195 198 263 315.1C 325 750 769 1018 1438 2706 3357 4769 5460 6167 7028 7376 7762 7775 8473 8631 8697 8701 8860 9540 10373 10398 10873 11253 11344 11485 11653 11719 12414 12705 13174 13344 14000A 14302 14766 15301 15326 15497 16207T 16223 16260 16261 16311 16362 16519 |
| Som87 | KP317077 | Somalia | L6a | 73 146 152 182 185C 228 263 265 315.1C 709 750 769 770 961 1018 1438 1461 2706 3335 3594 4769 4964 5267 6002 6284 7028 7145 7256 8701 8860G 9332 9540 10398 10873 10978 11116 11719 11743 12705 12771 13650 13710 14766 14791 14959 15244 15289 15301 15326 15499 16048 16129 16223 16224 16278 16311 16519 |
| Tur_02 | KP317078 | Kenya | L4b2a | 73 146 244 263 315.1C 455.1T 513 750 769 1018 1413 1438 2706 2863 3333 3918 4769 6260 7028 7129 8104 8701 8843 8860 9123 9287 9540 9855 10398 10873 11719 12173 12609 12681 12705 13470 14766 15217 15301 15326 16223 16293T 16301 16311 16355 16362 16399 16519 |
