# Supplementary material for: Genetic Stratigraphy of Key Demographic Events in Arabia
Source: PLoS One. 2015 Mar 4;10(3):e0118625. doi: 10.1371/journal.pone.0118625 (PMC4349752; doi:10.1371/journal.pone.0118625)
Supplement: S2 Table — (DOCX) [file pone.0118625.s040.docx]

**S2_Table** Published whole-mtDNA sequences used in all phylogenetic trees with the corresponding origin and subclade affiliation.

| **Accession Number/ID** | **Geographic region** | **Country/** | **Haplogroup** | **Ref.** | **Accession Number/ID** | **Geographic region** | **Country/** | **Haplogroup** | **Ref.** |
| --- | --- | --- | --- | --- | --- | --- | --- | --- | --- |
|  |  | **ethnicity** |  |  |  |  | **ethnicity** |  |  |
| JQ797762 | Europe | Greece | J1b1a2a | 34 | JF836809 |  | Unknown | T1a1a1 | 36 |
| HM852779 | South Caucasus | Armenia | J1b1a2a | 2 | JQ705663 | Europe | England | T1a1a1 | 35 |
| JQ797763 | Europe | Italy | J1b1a2b | 34 | JQ702035 |  | Unknown | T1a1a1 | 35 |
| JQ797764 | Europe | Italy | J1b1a2b | 34 | JQ701940 | Europe | Slovakia | T1a1a1 | 35 |
| EF660916 | Europe | Italy | J1b1a3 | 10 | JF830257 |  | Canada | T1a1a1 | 36 |
| JQ703595 | Europe | England | J1b1a3 | 35 | JQ702274 | Europe | England | T1a1a1 | 35 |
| JF286633 | South Caucasus | Armenia | J1b1a3 | 36 | JQ703708 | Europe | Ireland | T1a1a1 | 35 |
| AY714035 | Europe | India | J1b1a1 | 29 | JQ702962 |  | Unknown | T1a1a1 | 35 |
| JQ705247 |  | Unknown | J1b1a1 | 35 | JQ704967 | Europe | Netherlands | T1a1a1 | 35 |
| JQ702259 |  | Unknown | J1b1a1 | 35 | JQ704594 | Europe | Ireland | T1a1a1 | 35 |
| JQ701916 |  | Unknown | J1b1a1 | 35 | JQ705269 |  | Unknown | T1a1a1 | 35 |
| JQ705093 |  | Unknown | J1b1a1 | 35 | JQ705335 | Europe | France | T1a1a1 | 35 |
| JQ702434 | Europe | Spain | J1b1a1 | 35 | JQ705497 |  | Unknown | T1a1a1 | 35 |
| AY495235 |  | USA | J1b1a1 | 37 | JQ705955 |  | Unknown | T1a1a1 | 35 |
| JQ704791 |  | Unknown | J1b1a1 | 35 | JF930640 |  | Unknown | T1a1a1 | 36 |
| JQ704942 | Europe | France | J1b1a1 | 35 | EF645646 |  | Unknown | T1a1a2 | 36 |
| JQ704609 |  | Unknown | J1b1a1 | 35 | JF832384 |  | Unknown | T1a1a2 | 36 |
| FJ178380 | Europe | Italy | J1b1a1 | 38 | JQ705352 |  | Unknown | T1a1a2 | 35 |
| JQ701902 |  | Unknown | J1b1a1 | 35 | AY495296 |  | USA | T1a1a2 | 37 |
| JQ797760 | Europe | Italy | J1b1a1d | 34 | JQ798007 | Europe | Italy | T1a1b | 34 |
| JQ705588 |  | Unknown | J1b1a1d | 35 | JQ798008 | Europe | Italy | T1a1b | 34 |
| AY495238 |  | USA | J1b1a1d | 37 | JQ798009 | South Caucasus | Azerbaijan | T1a1b | 34 |
| JQ701852 |  | Unknown | J1b1a1 | 35 | JQ798023 | South Asia | India | T1a+16362 | 34 |
| JQ797761 | Europe | France | J1b1a1e | 34 | JQ798010 | Europe | Canary Islands | T1a1c1 | 34 |
| JQ705792 |  | Unknown | J1b1a1e | 35 | EF177441 | Europe | Portugal | T1a1c1 | 13 |
| JQ704777 |  | Unknown | J1b1a1e | 35 | JQ798011 | Europe | Crete | T1a1c1 | 34 |
| EU915478 | Europe | Italy | J1b1a1e | 38 | EF660961 | Europe | Italy | T1a1c1 | 10 |
| JQ703585 | Europe | Germany | J1b1a1e | 35 | FJ348220 | Europe | Italy | T1a1c1 | 16 |
| JQ704626 | Europe | United Kingdom | J1b1a1e | 35 | JQ702460 | Europe | France | T1a1c3 | 35 |
| HM856621 |  | USA | J1b1a1e | 36 | JN083377 | South Caucasus | Armenia | T1a1c | 36 |
| JQ703182 | Europe | Czech Republic | J1b1a1e | 35 | JQ798016 | Near East | Iraq | T1a1c | 34 |
| FJ213450 |  | USA | J1b1a1a | 36 | JQ798012 | Europe | Italy | T1a1c | 34 |
| JQ704118 | Europe | Ireland | J1b1a1a | 35 | JQ798013 | Europe | Italy | T1a1c2 | 34 |
| JQ703822 |  | Unknown | J1b1a1a | 35 | HM852775 | South Caucasus | Armenia | T1a1c2 | 2 |
| JQ702976 |  | Unknown | J1b1a1a | 35 | JQ798014 | Europe | Italy | T1a1c | 34 |
| HM600785 | Europe | England | J1b1a1a | 36 | JQ798015 | Arabian Peninsula | Kuwait | T1a1c | 34 |
| HQ286325 |  | USA | J1b1a1a | 36 | JQ798017 | Europe | Italy | T1a1d | 34 |
| JQ703574 |  | Unknown | J1b1a1a | 35 | JQ798018 | Anatolia | Turkey | T1a1d | 34 |
| JQ703249 |  | Unknown | J1b1a1a | 35 | JF831941 |  | Unknown | T1a1 | 36 |
| JQ702695 |  | Unknown | J1b1a1a | 35 | JQ798019 | Europe | Italy | T1a1e1 | 34 |
| JQ703588 | Europe | Ireland | J1b1a1a | 35 | JQ798020 | Europe | Crete | T1a1e1 | 34 |
| JQ701847 | Europe | Scotland | J1b1a1a | 35 | EU979542 |  | USA | T1a1g | 36 |
| JQ705909 |  | Unknown | J1b1a1 | 35 | JQ702040 |  | Unknown | T1a1g | 35 |
| JQ703802 |  | Unknown | J1b1a1 | 35 | JQ702952 | Europe | Scotland | T1a1g | 35 |
| JQ703569 |  | Unknown | J1b1a1 | 35 | JQ798021 | Arabian Peninsula | Kuwait | T1a1f | 34 |
| AY495234 |  | USA | J1b1a1 | 37 | EU935439 | North Africa | Egypt | T1a1f | 22 |
| JQ705923 | Europe | Germany | J1b1a1 | 35 | EU935455 | North Africa | Egypt | T1a1f | 4 |
| AY495231 |  | USA | J1b1a1c | 37 | JQ798022 | Near East | Israel | T1a1 | 34 |
| JQ705964 |  | Unknown | J1b1a1c | 35 | EF177409 | Europe | Portugal | T1a | 13 |
| HQ543056 |  | USA | J1b1a1c | 36 | JQ705058 |  | Unknown | T1a | 35 |
| AY495233 |  | USA | J1b1a1b | 37 | JQ798024 | Europe | Italy | T1a | 34 |
| JQ702866 |  | Unknown | J1b1a1b | 35 | JQ703693 | Europe | Lithuania | T1a | 35 |
| AY495237 |  | USA | J1b1a1b | 37 | JQ705713 |  | Unknown | T1a | 35 |
| AY495232 |  | USA | J1b1a1b | 37 | JQ798025 | Europe | Italy | T1a15 | 34 |
| JQ701907 | Europe | Russia | J1b1a1b | 35 | JQ798027 | Near East | Israel | T1a2 | 34 |
| AY495236 |  | USA | J1b1a1b | 37 | JQ798026 | Near East | Iraq | T1a2 | 34 |
| JQ704097 |  | Unknown | J1b1a1b | 35 | EU935447 | North Africa | Egypt | T1a2a | 4 |
| JQ797765 | Arabian Peninsula | Kuwait | J1b1b1 | 34 | EU935441 | North Africa | Egypt | T1a2a | 4 |
| EF397558 | Europe | Russia | J1b1b1 | 14 | EU935435 | North Africa | Egypt | T1a2a | 4 |
| FJ624455 | Europe | Italy | J1b1b1 | 36 | EU935452 | North Africa | Egypt | T1a2a | 4 |
| JQ797766 | Near East | Iran | J1b1b1a | 34 | EU935444 | North Africa | Egypt | T1a2a | 4 |
| EF556155 | Near East | Iran Jew | J1b1b1a | 1 | JQ798028 | Near East | Iraq | T1a14 | 34 |
| EF397562 | Europe | Russia | J1b1b1a | 14 | JQ798029 | Europe | Crete | T1a14 | 34 |
| JQ797768 | Near East | Iran | J1b1b1 | 34 | JQ798030 | South Asia | India | T1a3 | 34 |
| HM852784 | South Caucasus | Armenia | J1b1b1 | 2 | JQ798031 | North Africa | Algeria | T1a3 | 34 |
| JQ797769 | Near East | Iraq | J1b1b1b | 34 | JQ703724 | Europe | Germany | T1a3 | 35 |
| JF939049 | South Caucasus | Armenia | J1b1b1b | 36 | JQ798032 | Europe | Greece | T1a3 | 34 |
| JF929909 | South Caucasus | Armenia | J1b1b1 | 36 | JF937680 | Europe | England | T1a3 | 36 |
| JQ797767 | Near East | Iraq | J1b1b1 | 34 | JQ798033 | Europe | Italy | T1a4a | 34 |
| JQ703545 | Near East | Lebanon | J1b1b1 | 35 | JQ798034 | Near East | Iran | T1a4a | 34 |
| AY714033 | South Asia | India | J1b1b | 29 | JQ798035 | South Caucasus | Azerbaijan | T1a4b | 34 |
| JQ797770 | Near East | Iran | J1b1b | 34 | JQ798036 | Arabian Peninsula | Kuwait | T1a4b | 34 |
| JQ703656 |  | Unknown | J1b1b2 | 35 | JQ702139 | Europe | United Kingdom | T1a6 | 35 |
| JQ797772 | Europe | France | J1b2 | 34 | JN104727 |  | Unknown | T1a6 | 36 |
| DQ282492 |  | USA | J1b2 | 39 | EU369395 | Europe | Spain | T1a6 | 36 |
| DQ282490 |  | USA | J1b2 | 39 | JQ798037 | South Caucasus | Georgia | T1b1 | 34 |
| DQ282489 |  | USA | J1b2 | 39 | JQ798038 |  | Jordan | T1b1 | 34 |
| DQ282488 |  | USA | J1b2 | 39 | FJ878777 | Europe | United Kingdom | T1b5 | 36 |
| GU390312 |  | USA | J1b2 | 36 | JQ798039 | Europe | Italy | T1b2 | 34 |
| JQ797771 | Arabian Peninsula | Kuwait | J1b2a | 34 | JQ798040 | Europe | Italy | T1b2 | 34 |
| JQ702001 |  | Unknown | J1b2a | 35 | JQ798041 | Europe | Italy | T1b2 | 34 |
| JQ702684 |  | Unknown | J1b2 | 35 | JQ798042 | Near East | Iran | T1b3 | 34 |
| JQ703211 |  | Unknown | J1b2 | 35 | JQ798043 | Anatolia | Turkey | T1b3 | 34 |
| AF381987 | North Africa | Morocco | J1b2 | 5 | JQ705372 |  | Unknown | T1b3 | 35 |
| EF556169 | Near East | Iraq | J1b2b | 1 | JQ798044 | Europe | Karachay-Cherkessia Rep. (Russia) | T1b3 | 34 |
| JN648827 | South Caucasus | Armenia | J1b2b | 36 | JQ798045 | Europe | Kabardino-Balkaria Rep. (Russia) | T1b3 | 34 |
| EF660985 | Europe | Italy | J1b2 | 10 | JQ798046 | Arabian Peninsula | Saudi Arabia | T1b | 34 |
| JQ797773 | Arabian Peninsula | Kuwait | J1b2 | 34 | JQ798047 | North Africa | Egypt | T1b | 34 |
| JQ797774 | Near East | Iraq | J1b2 | 34 | JQ798048 | Europe | Italy | T1b | 34 |
| JQ797783 | Anatolia | Turkey | J1b7 | 34 | JQ798049 | Near East | Iraq | T1b4 | 34 |
| JQ797784 | Near East | Iraq | J1b7 | 34 | JQ798050 | Europe | Greece | T1b4 | 34 |
| JQ797775 | Europe | Greece | J1b3b | 34 | JQ798051 | South Asia | India | T1b | 34 |
| HM852838 | Near East | Iran | J1b3b | 2 | AY495298 |  | USA | T2a1a | 37 |
| EF583177 | Europe | Slovakia | J1b3a | 40 | AY495301 |  | USA | T2a1a | 37 |
| EF583175 | Europe | Slovakia | J1b3a | 40 | JN383991 | Europe | Norway | T2a1a | 36 |
| HM594676 | South Caucasus | Armenia | J1b3a | 36 | JQ705057 | Europe | England | T2a1a | 35 |
| JQ797786 | Arabian Peninsula | UAE | J1b | 34 | JQ703890 | Europe | France | T2a1a | 35 |
| JQ064573 | North Africa | Algeria | J1b8 | 36 | JQ702594 | Europe | Scotland | T2a1a | 35 |
| HQ914447 | South Caucasus | Armenia | J1b8 | 36 | JQ702379 |  | Unknown | T2a1a | 35 |
| JQ797782 | Near East | Iraq | J1b6b | 34 | JQ798052 | Near East | Iraq | T2a1a | 34 |
| JQ797780 | Arabian Peninsula | Kuwait | J1b6 | 34 | JQ798053 | Europe | Italy | T2a1a | 34 |
| JQ797781 | Europe | Italy | J1b6a | 34 | AY714022 | South Asia | India | T2a1a | 29 |
| DQ282491 |  | USA | J1b6a | 39 | AY495302 |  | USA | T2a1a5 | 37 |
| JQ702538 |  | Unknown | J1b6a | 35 | JQ705499 |  | Unknown | T2a1a5 | 35 |
| JQ797777 | North Ossetia | Alania Rep. (Russia) | J1b9a | 34 | JQ702880 |  | Unknown | T2a1a5 | 35 |
| JQ797778 | Near East | Iraq | J1b9a | 34 | AY495304 |  | USA | T2a1a | 37 |
| JQ797785 | Europe | Cyprus | J1b | 34 | JQ045864 | Europe | Denmark | T2a1a7 | 36 |
| JQ797776 | Europe | Greece | J1b4 | 34 | JQ701880 | Europe | Denmark | T2a1a7 | 35 |
| JN561091 | South Caucasus | Armenia | J1b4a1 | 36 | AY495303 |  | USA | T2a1a3 | 37 |
| HM852827 | Near East | Iran | J1b4a1 | 2 | AY495300 |  | USA | T2a1a3 | 37 |
| HM992836 | South Caucasus | Armenia | J1b4a2 | 36 | JF833041 |  | Unknown | T2a1a3 | 36 |
| HQ637485 | South Caucasus | Armenia | J1b4a2 | 36 | JQ702969 | Europe | Scotland | T2a1a | 35 |
| HM852835 | Near East | Iran | J1b5 | 2 | JQ703088 | Europe | England | T2a1a | 35 |
| JQ797779 | Near East | Iraq | J1b5a | 34 | JQ706008 |  | Unknown | T2a1a | 35 |
| HM852765 | South Caucasus | Armenia | J1b5a | 2 | JQ705133 | Europe | Ireland | T2a1a | 35 |
| JQ797787 | Europe | Ukraine | J1c1 | 34 | JQ702611 |  | Unknown | T2a1a | 35 |
| AY495216 |  | USA | J1c1a | 37 | FJ656215 | Europe | Germany | T2a1a | 22 |
| AY495210 |  | USA | J1c1a | 37 | FJ348181 | Europe | Italy | T2a1a1 | 16 |
| JQ705874 |  | Unknown | J1c1a | 35 | FJ348191 | Europe | Italy | T2a1a1 | 16 |
| JQ705114 |  | Unknown | J1c1a | 35 | FJ348180 | Europe | Italy | T2a1a1 | 16 |
| FJ447985 | Europe | Ireland | J1c1a | 36 | FJ348186 | Europe | Italy | T2a1a1 | 16 |
| JQ702620 |  | Unknown | J1c1a | 35 | GU553285 |  | USA | T2a1a2 | 36 |
| FJ348216 | Europe | Italy | J1c1 | 16 | JQ701997 |  | Unknown | T2a1a2 | 35 |
| JQ702440 | Europe | Belarus | J1c1d | 35 | JQ704617 | Europe | United Kingdom | T2a1a2 | 35 |
| JQ705051 |  | Unknown | J1c1d | 35 | JN120787 |  | Unknown | T2a1a2 | 36 |
| JQ797788 | Europe | Austria | J1c1b | 34 | HQ704899 |  | Australia | T2a1a2 | 36 |
| JQ702224 | Europe | England | J1c1b | 35 | JN630630 |  | Unknown | T2a1a2 | 36 |
| AY495202 |  | USA | J1c1b | 37 | JN012468 |  | Unknown | T2a1a2 | 36 |
| JQ705470 |  | Unknown | J1c1b | 35 | JQ702036 |  | Unknown | T2a1a6 | 35 |
| JQ703783 |  | Unknown | J1c1b | 35 | JQ704797 | Europe | Czech Republic | T2a1a6 | 35 |
| JQ705562 | Europe | Ireland | J1c1b | 35 | JF958082 |  | Unknown | T2a1a | 36 |
| HQ166708 |  | USA | J1c1b2a | 36 | JQ701972 |  | Unknown | T2a1a | 35 |
| JQ702020 | Europe | Ireland | J1c1b2a | 35 | JQ798054 | Europe | Italy | T2a1b | 34 |
| JQ704908 | Europe | United Kingdom | J1c1b2a | 35 | JQ702344 | Europe | Greece | T2a1b | 35 |
| JQ702981 |  | Unknown | J1c1b2a | 35 | JQ798056 | Europe | Adygea Rep. (Russia) | T2a1b2b | 34 |
| JQ705141 |  | Unknown | J1c1b2 | 35 | JQ798057 | South Caucasus | Azerbaijan | T2a1b2b | 34 |
| JQ703594 | Europe | United Kingdom | J1c1b2 | 35 | GU123001 | Europe | Russia | T2a1b2a | 20 |
| JQ702081 | Europe | Scotland | J1c1b2 | 35 | JQ704821 |  | Unknown | T2a1b2a | 35 |
| JQ797789 | Europe | Ukraine | J1c1b1a1 | 34 | JQ703730 |  | Unknown | T2a1b1a | 35 |
| JQ797790 | Europe | Romania | J1c1b1a1 | 34 | JQ702356 |  | Unknown | T2a1b1a | 35 |
| JQ703825 |  | Unknown | J1c1b1a | 35 | JQ703776 |  | Unknown | T2a1b1a | 35 |
| JQ703599 | Europe | Spain | J1c1b1a2 | 35 | JQ046361 | Europe | Ireland | T2a1b1a | 36 |
| JQ705164 | Europe | Scotland | J1c1b1a2 | 35 | JQ702972 | Europe | Italy | T2a1b1a | 35 |
| JQ797791 | Europe | Italy | J1c1b1a | 34 | JQ705641 | Europe | Germany | T2a1b1a | 35 |
| AY714034 | South Asia | India | J1c1b1a | 29 | JQ798055 | Europe | England | T2a1b1a1a | 34 |
| FJ502349 |  | Unknown | J1c1b1a | 36 | JQ703692 |  | Unknown | T2a1b1a1a | 35 |
| JQ703029 |  | Unknown | J1c1b1 | 35 | JQ703700 | Europe | Germany | T2a1b1a1a | 35 |
| JQ797792 | Europe | Italy | J1c1b1 | 34 | JQ703868 |  | Unknown | T2a1b1a1a | 35 |
| EF177420 | Europe | Portugal | J1c1b1 | 13 | JQ703149 | Europe | Germany | T2a1b1a1 | 35 |
| JQ703623 | Europe | Ireland | J1c1b1 | 35 | JF930649 |  | Unknown | T2a1b1a1 | 36 |
| EF452293 |  | Unknown | J1c1b1 | 36 | JQ702937 |  | Unknown | T2a1b1a1c | 35 |
| JQ703464 |  | Unknown | J1c1c | 35 | JN024624 | Europe | England | T2a1b1a1b | 36 |
| JQ704867 |  | Unknown | J1c1c | 35 | JN048933 |  | Unknown | T2a1b1a1b | 36 |
| JQ703653 |  | Unknown | J1c1c | 35 | JQ798058 | Near East | Iraq | T2a1 | 34 |
| JQ705770 | Europe | Ireland | J1c1f | 35 | HM852781 | South Caucasus | Armenia | T2a1 | 2 |
| JQ702685 |  | Unknown | J1c1 | 35 | JF927949 |  | Unknown | T2a1 | 36 |
| FJ348177 | Europe | Italy | J1c1 | 16 | JF944823 |  | Unknown | T2a2 | 36 |
| JQ797797 |  | Brazil-France | J1c1 | 34 | FJ238094 | South Caucasus | Armenia | T2a2 | 36 |
| JQ797793 |  | USA | J1c1 | 34 | HM852808 | South Caucasus | Azerbaijan | T2a3 | 2 |
| JQ797794 | Europe | Italy | J1c1 | 34 | AY195767 | Europe | Europe | T2b1 | 23 |
| JQ703557 |  | Unknown | J1c1 | 35 | AY495267 |  | USA | T2b1 | 37 |
| JQ797795 | Europe | Italy | J1c1 | 34 | EF660922 | Europe | Italy | T2b1 | 10 |
| JQ797796 | Europe | Italy | J1c1 | 34 | EF177444 | Europe | Portugal | T2b1 | 13 |
| EF177431 | Europe | Portugal | J1c1 | 13 | HM583750 | Europe | France | T2b1 | 36 |
| JQ797798 | Europe | England | J1c1d | 34 | JQ703293 |  | Unknown | T2b1 | 35 |
| AY195754 |  | Unknown | J1c1d | 23 | AY714016 | South Asia | India | T2b2 | 29 |
| AY495198 |  | USA | J1c1e | 37 | AY495305 |  | USA | T2b2+16304! | 37 |
| AY495208 |  | USA | J1c1e | 37 | EU683970 | Europe | Ireland | T2b2b | 36 |
| EU915479 | Europe | Italy | J1c2c | 38 | JF939816 |  | Unknown | T2b2b | 36 |
| JQ797822 | Europe | Italy | J1c2c | 34 | JF929911 |  | Unknown | T2b2b | 36 |
| JQ702581 |  | Unknown | J1c2 | 35 | EF660978 | Europe | Italy | T2b2b | 10 |
| JQ705421 | Europe | Finland | J1c2 | 35 | JN032298 | Europe | Belgium | T2b2b1 | 36 |
| JN663354 | South Caucasus | Armenia | J1c2 | 36 | JQ705119 |  | Unknown | T2b2b1 | 35 |
| JQ703591 |  | Unknown | J1c2 | 35 | JN084060 |  | Unknown | T2b2b1 | 36 |
| JQ704935 |  | Unknown | J1c2 | 35 | JQ705208 |  | Unknown | T2b2b1 | 35 |
| JQ701851 |  | Unknown | J1c2h | 35 | JQ701832 |  | Unknown | T2b2b1 | 35 |
| JQ705593 | Europe | United Kingdom | J1c2h | 35 | JN035224 |  | Unknown | T2b2+16304! | 36 |
| JQ705996 |  | Unknown | J1c2h | 35 | HM852840 | Near East | Iran | T2b2+16304! | 2 |
| JQ704033 | Europe | Slovakia | J1c2 | 35 | AY495299 |  | USA | T2b2+16304! | 37 |
| JQ797820 | Anatolia | Turkey | J1c2b | 34 | GU183768 | Europe | France | T2b2+16304! | 36 |
| JQ797821 | Europe | Italy | J1c2b | 34 | AY495275 |  | USA | T2b3b | 37 |
| EU155191 |  | Unknown | J1c2a | 36 | JN024623 |  | Unknown | T2b3b | 36 |
| JQ703794 |  | Unknown | J1c2a | 35 | JF937112 | Europe | England | T2b3b | 36 |
| JQ702445 | Europe | Norway | J1c2a | 35 | JF965447 |  | Unknown | T2b3b | 36 |
| JQ702219 | Europe | Norway | J1c2a | 35 | JN043363 |  | Unknown | T2b3b | 36 |
| JQ027716 | Europe | Russia | J1c2a | 36 | HM122274 |  | Tunisia | T2b3 | 36 |
| JQ704432 | Europe | Germany | J1c2a6 | 35 | JQ701927 |  | Unknown | T2b3d | 35 |
| JQ705715 | Europe | Russia | J1c2a6 | 35 | JQ705037 |  | Unknown | T2b3d | 35 |
| JQ705129 | Europe | Czechoslovakia | J1c2a6 | 35 | HM852813 | South Caucasus | Azerbaijan | T2b3+151 | 2 |
| JQ797813 | Europe | Romania | J1c2a7 | 34 | JF837334 | Europe | Ireland | T2b3e | 36 |
| JQ702366 |  | Unknown | J1c2a7a | 35 | JQ702951 | Europe | Scotland | T2b3e | 35 |
| JQ702724 | Europe | Ireland | J1c2a7a | 35 | JQ705417 | Europe | England | T2b3e | 35 |
| JQ797814 |  | Siberia | J1c2a7 | 34 | JQ705199 |  | Unknown | T2b3e | 35 |
| FJ499471 |  | USA | J1c2a8 | 36 | JQ705913 |  | Unknown | T2b3+151 | 35 |
| JQ703785 | Europe | England | J1c2a8 | 35 | JQ703699 | Europe | England | T2b3+151 | 35 |
| AY495223 |  | USA | J1c2a9 | 37 | JQ702932 |  | Unknown | T2b3c | 35 |
| JQ704829 | Europe | England | J1c2a9 | 35 | JQ703099 | Europe | France | T2b3c | 35 |
| JQ797799 | Europe | Ukraine | J1c2a10 | 34 | JQ705786 | Europe | Portugal | T2b3c | 35 |
| AY495225 |  | USA | J1c2a10 | 37 | JQ798063 | Europe | Italy | T2b3a1 | 34 |
| AY495224 |  | USA | J1c2a | 37 | JQ798060 | North Africa | Morocco | T2b3a1 | 34 |
| JQ703870 | Europe | Crete | J1c2a | 35 | JQ798062 | Europe | Italy | T2b3a1 | 34 |
| AY495219 |  | USA | J1c2a | 37 | EF660963 | Europe | Italy | T2b3a1 | 10 |
| FJ499472 | Europe | Norway | J1c2a11 | 36 | JQ798059 | Europe | Italy | T2b3a1 | 34 |
| JQ704965 |  | Unknown | J1c2a11 | 35 | JQ798061 | Europe | Italy | T2b3a1 | 34 |
| DQ358973 | Europe | Germany | J1c2a | 41 | DQ523660 | Europe | Sardinia | T2b3a | 15 |
| JQ705054 | Europe | Germany | J1c2a | 35 | DQ523651 | Europe | Sardinia | T2b3a | 15 |
| HM852774 | South Caucasus | Armenia | J1c2a | 2 | DQ523649 | Europe | Sardinia | T2b3a | 15 |
| JQ705724 |  | Unknown | J1c2a | 35 | JQ705834 |  | Unknown | T2b | 35 |
| JQ702515 |  | Unknown | J1c2a | 35 | HQ696459 |  | USA | T2b9 | 36 |
| JQ797800 | Europe | Italy | J1c2a5 | 34 | JQ703011 | Europe | Germany | T2b9 | 35 |
| JQ703675 | Europe | United Kingdom | J1c2a5 | 35 | JQ702737 |  | Unknown | T2b9 | 35 |
| JQ702311 | Europe | England | J1c2a5 | 35 | EU747356 | Europe | Germany | T2b+152 | 36 |
| JQ797801 | Europe | France | J1c2a5 | 34 | JQ798086 | Near East | Israel | T2b+152 | 34 |
| JQ797802 | Europe | Canary Islands | J1c2a5 | 34 | JQ701867 |  | Unknown | T2b+152 | 35 |
| JQ797803 | Europe | Bulgaria | J1c2a5a | 34 | HQ840646 |  | USA | T2b22 | 36 |
| GU123042 | Europe | Russia | J1c2a5a | 20 | JQ703263 |  | Unknown | T2b22 | 35 |
| JQ797808 |  | Siberia | J1c2a5a | 34 | JQ025225 | Europe | England | T2b21a | 36 |
| JQ797804 | Europe | Greece | J1c2a5 | 34 | JQ705895 |  | Unknown | T2b21a | 35 |
| JQ703746 | Europe | Italy | J1c2a5 | 35 | JF340114 |  | Newfoundland | T2b21a | 36 |
| JQ797805 | Europe | Greece | J1c2a5 | 34 | JQ705338 |  | Unknown | T2b21b | 35 |
| JQ797806 | Europe | Greece | J1c2a5 | 34 | JQ702492 |  | Unknown | T2b21b | 35 |
| JQ797807 | Europe | Greece | J1c2a5 | 34 | HQ917079 | Europe | Norway | T2b21 | 36 |
| JQ797809 | Europe | Albania | J1c2a5 | 34 | AY195745 |  | Unknown | T2b21 | 23 |
| JQ797810 | Europe | Romania | J1c2a5 | 34 | JQ705647 |  | Unknown | T2b21 | 35 |
| EF660952 | Europe | Italy | J1c2a | 10 | JQ702188 | Europe | Lithuania | T2b+152 | 35 |
| JQ797811 | Europe | Estonia | J1c2a12 | 34 | JQ705242 |  | Unknown | T2b4 | 35 |
| FJ190383 |  | USA | J1c2a12 | 36 | JQ798064 |  | Nepal | T2b4e | 34 |
| AY495226 |  | USA | J1c2a | 37 | JQ798065 |  | Nepal | T2b4e | 34 |
| JQ797812 | Europe | Lithuania | J1c2a | 34 | HM852802 | South Caucasus | Azerbaijan | T2b4e | 2 |
| JQ797815 | Europe | Poland | J1c2a13 | 34 | JQ704596 |  | Unknown | T2b4f | 35 |
| JQ705224 |  | Unknown | J1c2a13 | 35 | JQ703508 |  | Unknown | T2b4f | 35 |
| JQ797816 | Europe | Greece | J1c2a13 | 34 | JQ701897 |  | Unknown | T2b4f | 35 |
| JQ797817 | Europe | Greece | J1c2a | 34 | JQ798066 | Arabian Peninsula | Kuwait | T2b4h | 34 |
| JQ703556 |  | Unknown | J1c2a | 35 | EU007872 | Europe | Kazakhstan Rep. (Russia) | T2b4h | 31 |
| JQ701878 | Europe | Germany | J1c2a | 35 | JQ704869 |  | Unknown | T2b4 | 35 |
| JQ797818 | Europe | Greece | J1c2a | 34 | JQ702800 | Europe | England | T2b4 | 35 |
| JQ797819 | Europe | Romania | J1c2a3a | 34 | JQ702464 | Europe | Switzerland | T2b4 | 35 |
| FJ449571 | Europe | England | J1c2a3a | 36 | JQ701999 |  | Unknown | T2b4 | 35 |
| JQ702499 | Europe | British Isles | J1c2a3a | 35 | JQ798067 | Arabian Peninsula | Kuwait | T2b4 | 34 |
| JQ702018 | Europe | Finland | J1c2a3 | 35 | GU123018 | Europe | Russia | T2b4a | 20 |
| JQ703718 | Europe | England | J1c2a3 | 35 | EF177439 | Europe | Portugal | T2b4a | 13 |
| JQ705961 |  | Unknown | J1c2a3 | 35 | JQ704911 | Europe | England | T2b4a | 35 |
| GU949564 | Europe | Russia | J1c2a3 | 36 | JQ702564 |  | Unknown | T2b4a | 35 |
| EF177422 | Europe | Portugal | J1c2a3 | 13 | JQ705600 |  | Unknown | T2b4a | 35 |
| JQ703882 |  | Unknown | J1c2a3 | 35 | JQ798068 | Europe | Italy | T2b4a1 | 34 |
| EU573192 |  | Unknown | J1c2a3b | 36 | JQ704738 |  | Unknown | T2b4a1 | 35 |
| JQ702741 | Europe | Germany | J1c2a3b | 35 | JQ702323 |  | Unknown | T2b4a1 | 35 |
| FJ348202 | Europe | Italy | J1c2a3b | 16 | JF836084 |  | Unknown | T2b4a | 36 |
| HQ336424 | Europe | Ukraine | J1c2a3b1 | 36 | JF937089 | Europe | England | T2b4a | 36 |
| GU592034 | Europe | Austria | J1c2a3b1 | 42 | JQ703501 | Europe | Ireland | T2b4 | 35 |
| GU592019 | Europe | Austria | J1c2a3b1 | 42 | JQ704597 |  | Unknown | T2b4 | 35 |
| AY495230 |  | USA | J1c2a | 37 | JQ702516 | Europe | England | T2b4 | 35 |
| AY495228 |  | USA | J1c2a | 37 | JQ798069 |  | USA | T2b4 | 34 |
| AY495221 |  | USA | J1c2a | 37 | JQ798070 | Europe | Greece | T2b4 | 34 |
| AY495218 |  | USA | J1c2a | 37 | JN037468 |  | Unknown | T2b4 | 36 |
| HM590710 |  | USA | J1c2a | 36 | JN232198 |  | Unknown | T2b4 | 36 |
| HM803933 | Europe | Ukraine | J1c2a | 36 | JQ704746 | Europe | England | T2b4 | 35 |
| HM856585 | Europe | Finland | J1c2a | 36 | JQ798071 | Europe | Italy | T2b4g | 34 |
| JQ705442 |  | Unknown | J1c2a | 35 | EF660982 | Europe | Italy | T2b4g | 10 |
| JQ703461 |  | Unknown | J1c2a | 35 | JQ702012 |  | Unknown | T2b4 | 35 |
| JQ702715 |  | Unknown | J1c2a | 35 | EU492455 |  | USA | T2b4 | 36 |
| JQ702110 |  | Unknown | J1c2a | 35 | JF904739 |  | Unknown | T2b4 | 36 |
| JQ703612 | Europe | Scotland | J1c2a | 35 | JQ705939 | Europe | England | T2b4b | 35 |
| JQ703575 | Europe | Norway | J1c2a14 | 35 | JF900491 |  | Unknown | T2b4b | 36 |
| JQ703789 | Europe | England | J1c2a14 | 35 | JQ705883 |  | Unknown | T2b4b | 35 |
| JQ703818 | Europe | England | J1c2a | 35 | JQ704925 |  | Unknown | T2b4b | 35 |
| HQ260985 |  | USA | J1c2a15 | 36 | JQ704688 | Europe | Germany | T2b4b | 35 |
| JQ704946 |  | Unknown | J1c2a15 | 35 | JQ703842 | Europe | United Kingdom | T2b4b | 35 |
| AY495220 |  | USA | J1c2a1c | 37 | JN899566 |  | Unknown | T2b4 | 36 |
| JQ705006 |  | Unknown | J1c2a1c | 35 | JQ702058 | Europe | France | T2b4 | 35 |
| JQ702742 |  | Unknown | J1c2a1c | 35 | JQ703710 | Europe | Germany | T2b4 | 35 |
| DQ787109 |  | USA | J1c2a1b | 36 | JQ702158 |  | Unknown | T2b4d | 35 |
| JQ703955 |  | Unknown | J1c2a1b | 35 | JQ703734 | Europe | Ukraine | T2b4d | 35 |
| AY495227 |  | USA | J1c2a1a | 37 | JQ702604 |  | Unknown | T2b4c | 35 |
| JQ702390 |  | Unknown | J1c2a1a | 35 | JQ704742 | Europe | Germany | T2b4c | 35 |
| AY495229 |  | USA | J1c2a1a | 37 | JQ702278 |  | Unknown | T2b4 | 35 |
| AY495222 |  | USA | J1c2a2 | 37 | EU494628 |  | Unknown | T2b4 | 36 |
| JQ703778 |  | Unknown | J1c2a2 | 35 | JQ798072 | Near East | Iran | T2b5 | 34 |
| JQ704554 |  | Unknown | J1c2a2 | 35 | JQ798073 | Europe | Italy | T2b5 | 34 |
| JQ703567 |  | Unknown | J1c2a2d | 35 | AY495287 |  | USA | T2b5 | 37 |
| JQ703672 |  | Unknown | J1c2a2d | 35 | JQ702978 | Europe | Germany | T2b5 | 35 |
| JQ704384 |  | Unknown | J1c2a2 | 35 | JQ702884 |  | Unknown | T2b5 | 35 |
| JQ705986 |  | Unknown | J1c2a2 | 35 | JQ702666 |  | Unknown | T2b5 | 35 |
| AY495203 |  | USA | J1c2a2 | 37 | AY495286 |  | USA | T2b5 | 37 |
| HM776018 | Europe | Russia | J1c2a2b | 36 | GQ304744 |  | Unknown | T2b5 | 24 |
| JQ703610 | Europe | United Kingdom | J1c2a2b | 35 | JQ703716 | Europe | Germany | T2b5 | 35 |
| JQ704710 |  | Unknown | J1c2a2a | 35 | AY495283 |  | USA | T2b5+8504 | 37 |
| JQ703613 |  | Unknown | J1c2a2a | 35 | JF958132 |  | Unknown | T2b5a | 36 |
| JQ701968 | Europe | United Kingdom | J1c2a2c | 35 | JQ702982 | Europe | Switzerland | T2b5a | 35 |
| JQ702706 |  | Unknown | J1c2a2c | 35 | JF833039 |  | Unknown | T2b5a | 36 |
| JQ797823 | Europe | Greece | J1c3 | 34 | JQ705432 | Europe | England | T2b5a | 35 |
| AY495215 |  | USA | J1c3 | 37 | JQ703023 | Europe | Sweden | T2b5a | 35 |
| JQ705360 |  | Unknown | J1c3 | 35 | JQ701828 |  | Unknown | T2b5a | 35 |
| JQ701920 |  | Unknown | J1c3 | 35 | JQ703712 | Europe | England | T2b5a | 35 |
| AY495214 |  | USA | J1c3i | 37 | JQ702885 |  | Unknown | T2b5a | 35 |
| JQ703753 |  | Unknown | J1c3i | 35 | AY495284 |  | USA | T2b6 | 37 |
| JQ797824 | Europe | Greece | J1c3a1 | 34 | JF891418 |  | Unknown | T2b6b | 36 |
| AY495211 |  | USA | J1c3a1 | 37 | JQ704785 |  | Unknown | T2b6b | 35 |
| HM560728 |  | USA | J1c3a1 | 36 | JQ705047 |  | Unknown | T2b6b | 35 |
| JQ702747 |  | Unknown | J1c3a1 | 35 | JQ705972 |  | Unknown | T2b6b | 35 |
| JQ797825 | Europe | Greece | J1c3a1 | 34 | JQ705995 |  | Unknown | T2b6b | 35 |
| JQ704745 | Europe | England | J1c3a1 | 35 | JQ705357 | Europe | Germany | T2b6 | 35 |
| HQ839858 |  | Unknown | J1c3a1 | 36 | AY495282 |  | USA | T2b6a | 37 |
| JQ702646 |  | Unknown | J1c3a1 | 35 | JQ705494 | Europe | Czech Republic | T2b6a | 35 |
| AY495217 |  | USA | J1c3a2 | 37 | FJ573258 |  | USA | T2b6a | 36 |
| AY495213 |  | USA | J1c3a2 | 37 | JQ703772 | Europe | Switzerland | T2b6a | 35 |
| JQ704615 | Europe | Scotland | J1c3a2 | 35 | JQ798074 | Europe | Italy | T2b23 | 34 |
| JQ703792 | Europe | Netherlands | J1c3a2 | 35 | JQ798075 | Europe | Italy | T2b23 | 34 |
| JQ703469 | Europe | United Kingdom | J1c3a2 | 35 | JQ703458 | Europe | Belgium | T2b23 | 35 |
| JQ797826 | Europe | Adygea Rep. (Russia) | J1c3k | 34 | JQ798076 | Near East | Iran | T2b23 | 34 |
| JQ702912 |  | Unknown | J1c3k | 35 | FJ348211 | Europe | Italy | T2b23a | 16 |
| FJ445407 | Europe | Ireland | J1c3b1a | 36 | FJ348208 | Europe | Italy | T2b23a | 16 |
| HM627754 |  | USA | J1c3b1a | 36 | JQ704788 |  | Unknown | T2b23a | 35 |
| JQ703586 | Europe | Scotland | J1c3b1a | 35 | HM852879 | South Caucasus | Georgia | T2b | 2 |
| JQ705472 |  | Unknown | J1c3b1a | 35 | JQ704992 | Europe | England | T2b | 35 |
| JQ703671 | Europe | Netherlands | J1c3b1 | 35 | JQ703933 |  | Unknown | T2b | 35 |
| JQ705447 |  | Unknown | J1c3b1 | 35 | JQ703031 |  | Unknown | T2b | 35 |
| HQ696458 |  | USA | J1c3b1 | 36 | JQ702824 | Europe | England | T2b | 35 |
| JQ705085 | Europe | United Kingdom | J1c3b | 35 | JQ798080 | Europe | Italy | T2b29 | 34 |
| JQ702525 | Europe | Ireland | J1c3b | 35 | JQ798081 | Europe | France | T2b29 | 34 |
| JQ705280 |  | Unknown | J1c3b | 35 | JF830105 |  | Unknown | T2b | 36 |
| JQ705636 | Europe | Ireland | J1c3b | 35 | JQ798082 | Near East | Iraq | T2b | 34 |
| HM026752 | Europe | Ireland | J1c3b2 | 36 | AY495272 |  | USA | T2b | 37 |
| JQ702630 |  | Unknown | J1c3b2 | 35 | JQ798083 | Europe | Italy | T2b19 | 34 |
| JQ048704 | Europe | Portugal | J1c3b | 36 | EU744542 |  | USA | T2b19 | 36 |
| JQ703786 | Europe | Scotland | J1c3 | 35 | JQ705899 |  | Unknown | T2b19 | 35 |
| JQ702063 |  | Unknown | J1c3 | 35 | JQ704699 |  | Unknown | T2b19 | 35 |
| EF660962 | Europe | Italy | J1c3 | 10 | JQ703009 | Europe | Ireland | T2b19b | 35 |
| EU597522 |  | USA | J1c3 | 27 | JQ705680 | Europe | Scotland | T2b19b | 35 |
| FJ603099 |  | Unknown | J1c3 | 36 | JQ705664 | Europe | United Kingdom | T2b19 | 35 |
| HQ709108 |  | USA | J1c3f | 36 | JQ701939 | Europe | Germany | T2b | 35 |
| JQ797828 | Europe | Ukraine | J1c3f | 34 | JN603188 | Europe | Norway | T2b | 36 |
| JQ701854 |  | Unknown | J1c3f | 35 | JQ703083 |  | Unknown | T2b | 35 |
| JQ704665 | Europe | Ireland | J1c3f | 35 | JN419194 |  | Unknown | T2b | 36 |
| JQ705635 |  | Unknown | J1c3f | 35 | JN004135 |  | Unknown | T2b | 36 |
| JQ797829 | Europe | Italy | J1c3f | 34 | JQ798084 | Europe | Adygea Rep. (Russia) | T2b | 34 |
| JQ797833 | Europe | Sweden | J1c3f | 34 | JQ798085 | Europe | Kabardino-Balkaria Rep. (Russia) | T2b11 | 34 |
| JQ797831 | Europe | Kabardino-Balkaria Rep. (Russia) | J1c3f | 34 | JQ705009 | Europe | Denmark | T2b11 | 35 |
| JQ797830 | Europe | Ukraine | J1c3f | 34 | JQ703713 | Europe | England | T2b11 | 35 |
| JQ797832 | Europe | Kazakhstan Rep. (Russia) | J1c3f | 34 | JF968593 | Europe | Belarus | T2b11 | 36 |
| JQ701995 |  | Unknown | J1c3f | 35 | JN038393 |  | Unknown | T2b13a | 36 |
| JQ797827 | Europe | Estonia | J1c3f | 34 | JQ702084 |  | Unknown | T2b13a | 35 |
| JQ797836 | Europe | Italy | J1c3e2 | 34 | JQ703762 |  | Unknown | T2b13a | 35 |
| JQ705308 | Europe | England | J1c3e2 | 35 | JQ702435 |  | Unknown | T2b13 | 35 |
| JQ702686 |  | Unknown | J1c3e2 | 35 | JQ702963 | Europe | United Kingdom | T2b13 | 35 |
| JQ704840 | Europe | Sweden | J1c3e2 | 35 | JQ703042 | Europe | Netherlands | T2b13b | 35 |
| AY495195 |  | USA | J1c3e1 | 37 | JQ705492 |  | Unknown | T2b13b | 35 |
| JQ705868 |  | Unknown | J1c3e1 | 35 | JQ701982 |  | Unknown | T2b13 | 35 |
| HM852874 | Anatolia | Turkey | J1c3e1 | 2 | JQ705475 | Europe | Ireland | T2b | 35 |
| JQ703895 | Europe | Norway | J1c3e1 | 35 | JQ798087 | Europe | Italy | T2b+16296! | 34 |
| JQ701822 |  | Unknown | J1c3e1 | 35 | JQ653137 |  | USA | T2b+16296! | 36 |
| JQ702097 |  | Unknown | J1c3e1 | 35 | JQ798089 | Europe | Crete | T2b31 | 34 |
| JQ702691 |  | Unknown | J1c3 | 35 | JQ703715 |  | Unknown | T2b31 | 35 |
| JQ703597 |  | Unknown | J1c3 | 35 | JQ705484 | Europe | Ireland | T2b+16296! | 35 |
| JQ797834 | Europe | France | J1c3 | 34 | JQ798078 | Europe | Italy | T2b+16296! | 34 |
| JQ797835 | Europe | Italy | J1c3 | 34 | JQ702476 |  | Unknown | T2b+16296! | 35 |
| JF703252 |  | USA | J1c3g | 36 | JQ704973 | Europe | Denmark | T2b+16296! | 35 |
| JQ702788 | Europe | Norway | J1c3g | 35 | JQ702980 | Europe | Germany | T2b+16296! | 35 |
| JQ704779 | Europe | United Kingdom | J1c3g | 35 | JQ702965 | Europe | Germany | T2b | 35 |
| JQ704803 |  | Unknown | J1c3g | 35 | JQ798088 | Arabian Peninsula | UAE | T2b | 34 |
| JQ706013 |  | Unknown | J1c3g | 35 | JQ705328 | Europe | Belgium | T2b | 35 |
| JQ705212 |  | Unknown | J1c3g | 35 | HM055613 |  | USA | T2b | 36 |
| HQ709168 | Europe | France | J1c3c1 | 36 | JQ704780 | Europe | United Kingdom | T2b | 35 |
| JQ702395 |  | Unknown | J1c3c1 | 35 | AY495274 |  | USA | T2b | 37 |
| JQ703565 | Europe | Switzerland | J1c3c | 35 | AY495285 |  | USA | T2b | 37 |
| JQ702727 | Europe | England | J1c3c2 | 35 | AY495279 |  | USA | T2b | 37 |
| JQ703602 | Europe | England | J1c3c2 | 35 | AY495277 |  | USA | T2b | 37 |
| JQ797837 | Europe | Italy | J1c3h | 34 | AY495273 |  | USA | T2b | 37 |
| JQ797838 | Europe | Italy | J1c3h | 34 | AY495270 |  | USA | T2b | 37 |
| DQ523659 | Europe | Sardinia | J1c3h | 15 | FJ384436 | Europe | Europe | T2b | 9 |
| AY495201 |  | USA | J1c3d | 37 | FJ384435 | Europe | Europe | T2b | 9 |
| JQ705701 |  | Unknown | J1c3d | 35 | JN032303 | Europe | Finland | T2b | 36 |
| AY495207 |  | USA | J1c3d | 37 | JN106403 |  | Unknown | T2b | 36 |
| JQ701961 |  | Unknown | J1c3 | 35 | HM101563 | Europe | Spain | T2b | 36 |
| JQ704211 |  | Unknown | J1c3 | 35 | JQ702461 | Europe | Ireland | T2b | 35 |
| JQ705922 |  | Unknown | J1c3 | 35 | JQ702979 | Europe | Russia | T2b | 35 |
| DQ358974 | Europe | Germany | J1c3 | 43 | JQ701840 |  | Unknown | T2b | 35 |
| JQ702017 |  | Unknown | J1c3 | 35 | HQ231912 |  | USA | T2b | 36 |
| JQ705804 |  | Unknown | J1c3 | 35 | JQ703953 |  | Unknown | T2b | 35 |
| JQ703047 | Europe | Germany | J1c3 | 35 | JN315800 | Europe | England | T2b | 36 |
| HQ154118 |  | USA | J1c3 | 36 | JN558762 | Europe | England | T2b | 36 |
| JQ702826 | Europe | Ireland | J1c3 | 35 | JQ702312 |  | Unknown | T2b | 35 |
| JQ797839 | Europe | Italy | J1c4 | 34 | EU926622 |  | Unknown | T2b | 36 |
| JQ705388 |  | Unknown | J1c4 | 35 | JQ705948 |  | Unknown | T2b | 35 |
| JQ705414 |  | Unknown | J1c4 | 35 | JQ702953 |  | Unknown | T2b | 35 |
| JQ797840 |  | Siberia | J1c4 | 34 | HQ638221 | South Caucasus | Armenia | T2b | 36 |
| AY495197 |  | USA | J1c4 | 37 | EF660928 | Europe | Italy | T2b | 10 |
| AY495200 |  | USA | J1c4 | 37 | AY495269 |  | USA | T2b | 37 |
| AF346983 |  | USA | J1c4 | 44 | JF975728 |  | Unknown | T2b15 | 36 |
| GU123008 | Europe | Russia | J1c4 | 20 | EU682394 |  | USA | T2b15 | 36 |
| JQ797841 | Europe | Ukraine | J1c4b | 34 | JF979034 |  | USA | T2b15 | 36 |
| AY195774 |  | Unknown | J1c4b | 23 | HQ287876 | Europe | England | T2b | 32 |
| JQ703916 |  | Unknown | J1c4b | 35 | HQ287875 | Europe | England | T2b | 32 |
| AY665667 | Europe | Germany | J1c4b | 45 | AY495280 |  | USA | T2b17a | 37 |
| GU808335 | Europe | Russia | J1c4b | 36 | JQ705106 |  | Unknown | T2b17a | 35 |
| DQ341085 | Europe | Italy | J1c4 | 33 | JQ704783 | Europe | Ireland | T2b17a | 35 |
| DQ341086 | Europe | Italy | J1c4 | 33 | JF957699 | Europe | Ireland | T2b17 | 36 |
| EU915476 | Europe | Italy | J1c4 | 38 | FJ460542 | North Africa | Tunisia | T2b | 6 |
| FJ538285 |  | USA | J1c4 | 36 | FJ460555 | North Africa | Tunisia | T2b | 6 |
| HM016082 |  | USA | J1c4c | 36 | AY495281 |  | USA | T2b | 37 |
| JQ705633 |  | Unknown | J1c4c | 35 | JQ704897 | Europe | Ireland | T2b | 35 |
| JQ705439 |  | Unknown | J1c4c | 35 | AY495276 |  | USA | T2b | 37 |
| JQ797842 |  | Siberia | J1c5 | 34 | AY495278 |  | USA | T2b | 37 |
| JQ703750 | Europe | Poland | J1c5 | 35 | AY495268 |  | USA | T2b | 37 |
| JQ702776 | Europe | France | J1c5c | 35 | AY495271 |  | USA | T2b27 | 37 |
| JQ704051 |  | Unknown | J1c5c | 35 | JQ705444 |  | Unknown | T2b27 | 35 |
| JQ704816 | Europe | Greece | J1c5c | 35 | GU123010 | Europe | Russia | T2b | 20 |
| HQ287873 | Europe | England | J1c5d | 32 | GU170817 | South Asia | India | T2b7 | 21 |
| AY495204 |  | USA | J1c5d | 37 | JQ798079 | Europe | Italy | T2b7a3 | 34 |
| HQ907957 |  | USA | J1c5d | 36 | JQ704643 | Europe | Italy | T2b7a2 | 35 |
| JQ702201 |  | Unknown | J1c5d | 35 | JQ704997 | Europe | France | T2b7a2 | 35 |
| JQ703604 |  | Unknown | J1c5b | 35 | JQ702544 |  | Unknown | T2b7a1 | 35 |
| JQ705397 |  | Unknown | J1c5b | 35 | JQ703062 | Europe | Sweden | T2b7a1 | 35 |
| JQ703639 | Europe | Germany | J1c5b | 35 | JQ703294 | Europe | British Isles | T2b7a1 | 35 |
| JQ797843 | Europe | Italy | J1c5 | 34 | JQ703714 |  | Unknown | T2b7a1 | 35 |
| JQ797844 | South Asia | India | J1c5e | 34 | JQ705882 | Europe | England | T2b25 | 35 |
| JQ703740 |  | Unknown | J1c5e | 35 | JQ704697 | Europe | Romania | T2b25 | 35 |
| JQ797845 | Europe | Ukraine | J1c5a | 34 | JN024625 | Europe | Ireland | T2b30 | 36 |
| JQ703803 | Europe | Ireland | J1c5a | 35 | HQ399469 |  | USA | T2b30 | 36 |
| HQ287874 | Europe | England | J1c5a1 | 32 | JQ704319 | Europe | England | T2b | 35 |
| JQ705488 |  | Unknown | J1c5a1 | 35 | JQ701986 |  | Unknown | T2b | 35 |
| JQ703558 | Europe | Ireland | J1c5a1 | 35 | JQ702239 | Europe | United Kingdom | T2b | 35 |
| JQ702996 | Europe | Germany | J1c5a1 | 35 | JQ702645 |  | Unknown | T2b | 35 |
| JQ701921 |  | Unknown | J1c5a1 | 35 | JN004272 | Europe | Germany | T2b | 36 |
| JQ704736 |  | Unknown | J1c5a1 | 35 | JN419195 | Europe | France | T2b | 36 |
| JQ702386 |  | Unknown | J1c5a1 | 35 | JF980711 |  | Unknown | T2b | 36 |
| JQ705031 |  | Unknown | J1c5 | 35 | JN106183 | Europe | Germany | T2b | 36 |
| JQ703572 |  | Unknown | J1c5 | 35 | JQ798077 | Europe | Estonia | T2b16 | 34 |
| JQ705581 |  | Unknown | J1c5 | 35 | EU007870 | Europe | Kazakhstan Rep. (Russia) | T2b16 | 31 |
| EU007859 |  | Yakut | J1c5 | 31 | GU123025 | Europe | Russia | T2b16 | 20 |
| AY495212 |  | USA | J1c5 | 37 | JN126047 | Europe | Germany | T2b | 36 |
| AY495199 |  | USA | J1c5 | 37 | GU122998 | Europe | Russia | T2b | 20 |
| HM159445 |  | Ashkenazi Jew | J1c5 | 36 | JQ705351 | Europe | Scotland | T2b | 35 |
| JQ703448 | Europe | England | J1c5 | 35 | JQ704505 | Europe | Germany | T2b | 35 |
| AY495209 |  | USA | J1c6 | 37 | JQ703722 |  | Unknown | T2b | 35 |
| JQ703919 | Europe | Italy | J1c6 | 35 | JQ703680 | Europe | Scotland | T2b | 35 |
| AY495196 |  | USA | J1c6 | 37 | JQ702894 | Europe | Finland | T2b | 35 |
| EU073970 |  | Unknown | J1c6 | 36 | JQ703728 |  | Unknown | T2b | 35 |
| JQ705489 |  | Unknown | J1c7 | 35 | JQ705446 | Europe | Ireland | T2b | 35 |
| JQ797847 | Europe | Greece | J1c7 | 34 | JQ705758 |  | Unknown | T2b | 35 |
| JQ797848 | Europe | Greece | J1c7 | 34 | JQ705854 | Europe | Norway | T2b | 35 |
| JQ797849 | Europe | Romania | J1c7 | 34 | JF929201 |  | Unknown | T2b24 | 36 |
| JQ797850 | Europe | Romania | J1c7 | 34 | JQ705038 | Europe | United Kingdom | T2b24 | 35 |
| EF583178 | Europe | Slovakia | J1c7 | 40 | JF946696 |  | Unknown | T2b24a | 36 |
| EU547187 | Europe | Romania | J1c7 | 36 | JQ702547 |  | Unknown | T2b24a | 35 |
| EF459669 |  | Unknown | J1c7 | 36 | JQ798090 | South Asia | India | T2c | 34 |
| JQ797851 | Europe | Greece | J1c7 | 34 | EF556164 |  | Unknown | T2c1a1 | 1 |
| JQ797852 | Europe | Bosnia and Herzegovina | J1c7 | 34 | EF556160 |  | Unknown | T2c1a1 | 1 |
| JQ797853 | Europe | Romania | J1c7 | 34 | JQ703030 | Near East | Iraq | T2c1a1 | 35 |
| JQ797854 | Europe | Greece | J1c7 | 34 | EF660941 | Europe | Italy | T2c1a2 | 10 |
| EU284668 |  | Unknown | J1c7b | 36 | HM765471 |  | Unknown | T2c1a | 11 |
| JQ703516 |  | Unknown | J1c7b | 35 | JN202494 |  | Unknown | T2c1a | 36 |
| JQ704581 |  | Unknown | J1c7b | 35 | JQ703691 |  | Unknown | T2c1a | 35 |
| FJ348222 | Europe | Italy | J1c7b | 16 | JQ701934 |  | Unknown | T2c1a | 35 |
| JQ702088 |  | Unknown | J1c7b | 35 | JQ798091 | Europe | Italy | T2c1b | 34 |
| JQ702208 | Europe | England | J1c7b | 35 | JQ703823 | Europe | Belgium | T2c1b2 | 35 |
| JQ797861 | Europe | Italy | J1c7 | 34 | JQ705571 | Europe | Germany | T2c1b2 | 35 |
| JQ702609 |  | Unknown | J1c7c | 35 | JF951723 |  | Unknown | T2c1b1a | 36 |
| JQ703584 |  | Unknown | J1c7c | 35 | JN252308 |  | Unknown | T2c1b1a | 36 |
| JQ797860 | Europe | Italy | J1c7 | 34 | JN580589 |  | Unknown | T2c1b1a1 | 36 |
| EU151466 | Europe | Spain | J1c7a1 | 30 | JQ705668 |  | Unknown | T2c1b1a1 | 35 |
| JQ703861 | Europe | England | J1c7a1 | 35 | JQ798092 | Europe | Spain | T2c1b1a1 | 34 |
| JQ701821 |  | Unknown | J1c7a1 | 35 | JQ704020 |  | Unknown | T2c1b1a | 35 |
| JQ703682 |  | Unknown | J1c7a1 | 35 | JQ798093 | Arabian Peninsula | Kuwait | T2c1b1a | 34 |
| JQ797855 | Europe | Belarus | J1c7a1 | 34 | JF833037 |  | Unknown | T2c1b1 | 36 |
|  | Europe | Italy | J1c7a1a | 34 | JF892552 |  | Unknown | T2c1b1 | 36 |
| FJ348162 |  | USA | J1c7a1a | 16 | JQ798094 | Europe | Spain | T2c1b1 | 34 |
| FJ348163 |  | USA | J1c7a1a | 16 | JQ702928 |  | Unknown | T2c1b1 | 35 |
| FJ348164 |  | USA | J1c7a1a | 16 | JQ798095 | Europe | Italy | T2c1b1b1 | 34 |
| FJ348165 |  | USA | J1c7a1a | 16 | DQ523629 | Europe | Sardinia | T2c1b1b1 | 15 |
| JQ705332 |  | Unknown | J1c7a1a | 35 | DQ523633 | Europe | Sardinia | T2c1b1b1 | 15 |
| JQ703562 | Europe | Sweden | J1c7a1a | 35 | DQ523667 | Europe | Sardinia | T2c1b1b1 | 15 |
| FJ348161 |  | USA | J1c7a1a | 16 | JQ798096 | Near East | Israel | T2c1b1b | 34 |
| JQ702929 | Europe | Germany | J1c7a1a | 35 | GU048747 |  | Unknown | T2c1b3 | 36 |
| JQ703932 |  | Unknown | J1c7a1a | 35 | JQ798097 | Europe | Czech Republic | T2c1b3 | 34 |
| JQ701859 |  | Unknown | J1c7a1a | 35 | JQ798098 | Near East | Iran | T2c1b | 34 |
| JQ702243 | Europe | Hungary | J1c7a1a | 35 | JQ798099 | Europe | Italy | T2c1c1 | 34 |
| HM627319 |  | Ashkenazi Jew | J1c7a1a | 36 | JQ798100 | Near East | Israel | T2c1c1 | 34 |
| JQ705271 | Europe | England | J1c7a1a | 35 | JQ798101 | Europe | Italy | T2c1c | 34 |
| JQ705072 |  | Unknown | J1c7a1a | 35 | JQ798103 | Arabian Peninsula | UAE | T2c1c | 34 |
| JF812166 |  | Ashkenazi Jew | J1c7a1a | 36 | JQ798102 |  | Crete | T2c1c2 | 34 |
| JQ797856 | Europe | Belarus | J1c7a1a | 34 | JQ702795 | Europe | Finland | T2c1c2 | 35 |
| JQ797857 | Europe | Belarus | J1c7a1a1 | 34 | JQ798105 |  | Siberia | T2d1b1 | 34 |
| JQ705094 |  | Unknown | J1c7a1a1 | 35 | JQ798106 |  | Siberia | T2d1b1 | 34 |
| JQ705322 | Europe | Norway | J1c7a1a1 | 35 | JQ798107 |  | Siberia | T2d1b1 | 34 |
| JQ797858 | Europe | Sweden | J1c7a1a1 | 34 | HM765473 |  | Unknown | T2d1b1 | 11 |
| JQ705394 | Europe | Norway | J1c7a1a1 | 35 | DQ437577 |  | Mongolia | T2d1b | 19 |
| JQ703583 | Europe | Finland | J1c7a1a1 | 35 | AY714037 | South Asia | India | T2d1a | 29 |
| JQ704103 |  | Unknown | J1c7a1a1 | 35 | JQ703754 | Europe | Scotland | T2d1a | 35 |
| JQ702857 |  | Unknown | J1c7a1a1 | 35 | JQ798104 | South Asia | India | T2d1a | 34 |
| JQ702338 |  | Unknown | J1c7a1a1 | 35 | JQ798108 | Near East | Iran | T2d2 | 34 |
| JQ797859 | Europe | Ukraine | J1c7a1a1 | 34 | JQ798109 | Europe | Italy | T2d2 | 34 |
| HM171294 |  | USA | J1c7a1a | 36 | HM852899 | South Caucasus | Georgia | T2d2 | 2 |
| HQ876599 | Europe | Spain | J1c7 | 36 | EF177410 | Europe | Portugal | T2e | 13 |
| JN966735 | Europe | Italy | J1c7 | 36 | GU565218 |  | Unknown | T2e | 36 |
| JQ702486 |  | Unknown | J1c7 | 35 | JQ705673 |  | Unknown | T2e | 35 |
| JQ702176 | Europe | Austria | J1c7d | 35 | JQ702954 | Europe | Italy | T2e | 35 |
| JQ705424 | Europe | Germany | J1c7d | 35 | JQ701853 |  | Unknown | T2e | 35 |
| JQ705811 |  | Unknown | J1c7d | 35 | EF060363 | Europe | Italy | T2e2a | 18 |
| JQ705683 |  | Unknown | J1c7d | 35 | JQ798114 | Near East | Iraq | T2e2a | 34 |
| JQ704569 | Europe | Poland | J1c7d | 35 | AY714029 | South Asia | India | T2e2 | 29 |
| JQ702827 |  | Unknown | J1c7d | 35 | EU597536 | Europe | Czechoslovakia | T2e | 27 |
| JQ705891 |  | Ashkenazi Jew | J1c7d | 35 | HM852862 | Anatolia | Turkey | T2e | 2 |
| JQ797862 | Arabian Peninsula | Kuwait | J1c7e | 34 | JQ798115 | Europe | Italy | T2e | 34 |
| FJ348155 |  | USA | J1c7e | 16 | JQ798116 | Europe | Italy | T2e | 34 |
| FJ348154 |  | USA | J1c7e | 16 | JQ703777 | Europe | Croatia | T2e | 35 |
| FJ348153 |  | USA | J1c7e | 16 | JQ798111 | Europe | Greece | T2e3 | 34 |
| JQ797863 | Europe | Greece | J1c8 | 34 | JQ798112 | Europe | Greece | T2e3 | 34 |
| JQ702228 | Europe | United Kingdom | J1c8 | 35 | EU703624 | Europe | Italy | T2e3 | 36 |
| JQ702868 |  | Unknown | J1c8 | 35 | JN030346 |  | Unknown | T2e4 | 36 |
| JQ797864 |  | Unknown | J1c8b | 34 | JN828512 |  | USA | T2e4 | 36 |
| GU906781 |  | Unknown | J1c8b | 36 | JN819272 |  | Sephardic Jew | T2e4 | 36 |
| JQ797865 | South Asia | India | J1c8 | 34 | AF381985 | North Africa | Morocco | T2e4 | 5 |
| JQ797866 | Europe | Sweden | J1c8 | 34 | JQ705148 |  | Unknown | T2e+16296! | 35 |
| JQ704604 | Europe | England | J1c8 | 35 | JQ798110 | Europe | Italy | T2e+16296! | 34 |
| JQ702876 | Europe | Ireland | J1c8 | 35 | EU700086 |  | Unknown | T2e+16296! | 36 |
| JQ797867 | Europe | Greece | J1c8 | 34 | EF556188 | Europe | Bulgaria Jew | T2e1 | 1 |
| JQ705860 | Europe | England | J1c8 | 35 | JQ705465 | Europe | Ireland | T2e1 | 35 |
| JQ797868 | Europe | England | J1c8 | 34 | FJ178379 | Europe | Italy | T2e1 | 38 |
| JQ704891 |  | Unknown | J1c8 | 35 | JQ702286 |  | Unknown | T2e1 | 35 |
| JQ702068 |  | Unknown | J1c8a | 35 | JQ702731 | Europe | Belgium | T2e1 | 35 |
| JQ705760 | Europe | Germany | J1c8a | 35 | EU258890 |  | Unknown | T2e1 | 36 |
| JQ797869 |  | Unknown | J1c8a | 34 | JQ798113 | Europe | Italy | T2e1 | 34 |
| JQ702554 |  | Unknown | J1c8a | 35 | GU944474 |  | USA | T2e1 | 36 |
| JQ702049 |  | Unknown | J1c8a | 35 | JF893457 |  | Unknown | T2e1 | 36 |
| JQ704576 | Europe | Ireland | J1c8 | 35 | JF903810 |  | Unknown | T2e1 | 36 |
| JQ703402 | Europe | Ireland | J1c8 | 35 | JF831146 | Europe | England | T2e1 | 36 |
| JQ797870 | Europe | Italy | J1c | 34 | JQ702210 |  | Unknown | T2e | 35 |
| GU123016 | Europe | Russia | J1c12 | 20 | JQ701985 |  | Unknown | T2e5 | 35 |
| JQ797875 | Near East | Iraq | J1c12a | 34 | JQ702891 |  | Unknown | T2e5 | 35 |
| JQ797876 | Near East | Israel | J1c12a | 34 | JF965448 |  | Unknown | T2e | 36 |
| JQ701870 |  | Unknown | J1c12 | 35 | JQ703661 | Europe | England | T2e | 35 |
| JQ797879 | Europe | Italy | J1c12 | 34 | JQ704818 | Europe | England | T2e | 35 |
| JQ797877 | Arabian Peninsula | Kuwait | J1c12b | 34 | JF921152 |  | Unknown | T2e | 36 |
| HM852793 | South Caucasus | Azerbaijan | J1c12b1 | 2 | JF831421 |  | Unknown | T2e | 36 |
| JQ797878 | Near East | Iraq | J1c12b1 | 34 | JF937679 |  | Unknown | T2e | 36 |
| JQ797881 | South Caucasus | Azerbaijan | J1c16 | 34 | JQ798117 | Arabian Peninsula | Kuwait | T2e | 34 |
| JQ705083 |  | Unknown | J1c16 | 35 | JQ798118 | Europe | Italy | T2e6 | 34 |
| JQ704306 | Europe | England | J1c16 | 35 | JQ798119 | Europe | Italy | T2e6 | 34 |
| JQ797872 | Europe | Italy | J1c10a | 34 | AF346982 | South Caucasus | Georgia | T2e | 44 |
| JQ797873 | North Africa | Morocco | J1c10a | 34 | JQ703702 | Europe | Germany | T2f+195 | 35 |
| JQ704751 | Europe | England | J1c10 | 35 | JQ798120 | Europe | Ukraine | T2f1 | 34 |
| EF660915 | Europe | Italy | J1c10 | 10 | JQ798121 | Europe | England | T2f1a1a | 34 |
| JQ797874 | Europe | Italy | J1c11a | 34 | JF940522 | Europe | Sweden | T2f1a1a | 36 |
| HM765470 | Europe | Italy | J1c11a | 11 | JQ798122 | Europe | Italy | T2f1a1a | 34 |
| GU592047 | Europe | Austria | J1c11a | 42 | JQ702642 |  | Unknown | T2f1a1a | 35 |
| GU592032 | Europe | Austria | J1c11a | 42 | JQ705857 |  | Unknown | T2f1a1a | 35 |
| JQ702967 | Europe | Germany | J1c11 | 35 | JQ798123 | Europe | Denmark | T2f1a1a | 34 |
| JQ704937 | Europe | Scotland | J1c | 35 | JQ703663 |  | Unknown | T2f1a1a | 35 |
| JQ797880 | Europe | Italy | J1c17 | 34 | JQ705015 | Europe | Denmark | T2f1a1a | 35 |
| JQ706022 |  | Unknown | J1c17 | 35 | JQ705023 |  | Unknown | T2f1a1a | 35 |
| JQ797871 | Europe | Italy | J1c9 | 34 | JQ705644 |  | Unknown | T2f1a1a | 35 |
| JQ703686 |  | Unknown | J1c9 | 35 | JQ703806 |  | Unknown | T2f1a1a | 35 |
| AY495205 |  | USA | J1c9 | 37 | JQ705892 |  | Unknown | T2f1a1a | 35 |
| JQ705977 | Europe | United Kingdom | J1c9 | 35 | JQ705879 |  | Unknown | T2f1a1a | 35 |
| JQ797883 | North Africa | Morocco | J1c | 34 | JQ705897 | Europe | Bosnia and Herzegovina | T2f1a1a | 35 |
| JQ797846 | South Caucasus | Armenia | J1c | 34 | JQ705927 |  | Unknown | T2f1a1a | 35 |
| JQ797882 | Europe | Greece | J1c | 34 | JQ704807 |  | Unknown | T2f1a1a | 35 |
| JQ705449 |  | Unknown | J1c | 35 | GU932663 |  | Unknown | T2f1a1a | 36 |
| JQ701992 |  | Unknown | J1c | 35 | JF905568 |  | Unknown | T2f1a1a | 36 |
| AY495206 |  | USA | J1c | 37 | JF906520 |  | Unknown | T2f1a1a | 36 |
| JQ797884 | Europe | Greece | J1c19 | 34 | JF926842 |  | Unknown | T2f1a1a | 36 |
| HM775495 | South Caucasus | Armenia | J1c19 | 36 | JN203494 |  | Unknown | T2f1a1a | 36 |
| JQ797885 | Europe | Dagestan Rep. (Russia) | J1 | 34 | JN887353 | Europe | Sweden | T2f1a1a | 36 |
| JQ797886 | Europe | Italy | J1d1a | 34 | JQ702350 |  | Unknown | T2f1a1 | 35 |
| JQ797887 | Arabian Peninsula | Kuwait | J1d1a | 34 | JN003847 |  | Unknown | T2f1a1 | 36 |
| JQ797888 | Arabian Peninsula | Kuwait | J1d1a1 | 34 | JQ702975 |  | Unknown | T2f1a1 | 35 |
| JQ797889 | Near East | Iraq | J1d1a1 | 34 | GU123028 | Europe | Russia | T2f1a | 20 |
| JQ705319 |  | Unknown | J1d1a1 | 35 | HQ287877 | Europe | Ireland | T2f1a | 32 |
| JQ797890 | East Africa | Uganda | J1d1a1a | 34 | JN084792 |  | Unknown | T2f | 36 |
| JQ797891 | East Africa | Uganda | J1d1a1a | 34 | JQ705528 |  | Unknown | T2f4 | 35 |
| EF184636 | East Africa | Tanzania | J1d1a1 | 28 | JN034044 |  | Canada | T2f4 | 36 |
| JF292900 | South Caucasus | Armenia | J1d1a | 36 | JQ798124 | Europe | Italy | T2f2 | 34 |
| JQ797892 | Europe | Cyprus | J1d1b1 | 34 | JQ798125 | Europe | Greece | T2f2 | 34 |
| HM852780 | South Caucasus | Armenia | J1d1b1 | 2 | HQ286590 | South Caucasus | Armenia | T2f2 | 36 |
| AF382001 | Europe | Iberia | J1d1b | 5 | JQ702294 |  | Unknown | T2f5 | 35 |
| JQ797893 | Near East | Iran | J1d2a1 | 34 | JQ702957 |  | Unknown | T2f5 | 35 |
| HQ325739 | South Caucasus | Armenia | J1d2a1 | 36 | JQ619780 | Europe | Finland | T2f | 36 |
| DQ341087 |  | Brazil-Italy | J1d2a | 33 | JQ703997 | Europe | Italy | T2f | 35 |
| DQ341088 |  | Brazil-Italy | J1d2a | 33 | JQ703697 |  | Unknown | T2f | 35 |
| JQ797902 | Near East | Iraq | J1d | 34 | JF960209 |  | Unknown | T2f3 | 36 |
| JQ797900 | South Asia | India | J1d2d | 34 | JQ705221 |  | Unknown | T2f3 | 35 |
| HM453206 | South Caucasus | Armenia | J1d2d | 36 | JQ705639 |  | Unknown | T2f3 | 35 |
| JQ797894 | North Ossetia | Alania Rep. (Russia) | J1d2b | 34 | JQ704426 | Europe | United Kingdom | T2f3 | 35 |
| EU007880 |  | Mansi | J1d2b1 | 31 | JQ704129 | Europe | Ireland | T2f | 35 |
| JQ797895 |  | Siberia | J1d2b1 | 34 | JQ798129 | Europe | Italy | T2i | 34 |
| GU122987 | Europe | Russia | J1d2b | 20 | HM852881 | South Caucasus | Georgia | T2i | 2 |
| JQ797896 | South Caucasus | Georgia | J1d2c1 | 34 | JQ798130 | Near East | Iraq | T2i1 | 34 |
| JQ797897 | Europe | Adygea Rep. (Russia) | J1d2c1 | 34 | JQ704055 |  | Unknown | T2i1 | 35 |
| JQ797898 | North Ossetia | Alania Rep. (Russia) | J1d2c1 | 34 | JQ798131 | Near East | Iran | T2i | 34 |
| JQ797899 | Europe | Italy | J1d2c | 34 | JQ702108 | Europe | Germany | T2g2 | 35 |
| JQ704809 |  | Unknown | J1d2c2 | 35 | JQ702574 |  | Unknown | T2g2 | 35 |
| EU597552 | South Asia | Pakistan | J1d2c2 | 27 | JQ702710 |  | Unknown | T2g2 | 35 |
| JQ797901 | Near East | Iran | J1d2e | 34 | JQ798126 | Europe | Italy | T2g1a | 34 |
| JQ703915 | Anatolia | Turkey | J1d2e | 35 | JN086657 | Europe | Lithuanian Jew | T2g1a1 | 36 |
| HM852829 | Near East | Iran | J1d2e | 2 | JQ798127 | Europe | Italy | T2g1a1a | 34 |
| JQ704041 | Europe | Ireland | J2a1 | 35 | EF556186 | Near East | Iran Jew | T2g1a1a1 | 1 |
| JQ797903 | Europe | Italy | J2a1a1 | 34 | JQ705079 | Near East | Iraq | T2g1a1a1 | 35 |
| HQ699438 | Europe | Sweden | J2a1a1 | 36 | JQ705097 |  | Unknown | T2g1a1a1 | 35 |
| JQ705358 |  | Unknown | J2a1a1 | 35 | JQ703123 | Europe | Hungary | T2g1a1a1 | 35 |
| JQ702216 |  | Unknown | J2a1a1 | 35 | EU007862 |  | Yakut | T2g1a1a | 31 |
| JQ702091 |  | Unknown | J2a1a1c | 35 | EU935442 | North Africa | Egypt | T2g1a1a | 4 |
| JQ705512 |  | Unknown | J2a1a1c | 35 | EU935446 | North Africa | Egypt | T2g1a1a | 4 |
| JQ797904 | Europe | France | J2a1a1c | 34 | EU935448 | North Africa | Egypt | T2g1a1a | 4 |
| JQ703552 | Europe | British Isles | J2a1a1c | 35 | JQ798132 | South Asia | India | T2 | 34 |
| JQ797911 | Europe | Italy | J2a1a1 | 34 | JQ798133 | South Caucasus | Azerbaijan | T2 | 34 |
| JQ797905 | Europe | Italy | J2a1a1d | 34 | JN037469 |  | USA | T2h | 36 |
| JQ797906 | Europe | Italy | J2a1a1d | 34 | JQ798128 | Europe | Italy | T2h | 34 |
| EU931680 |  | USA | J2a1a1d | 36 | JQ702170 | Europe | Slovakia | T2h | 35 |
| JQ797912 | Europe | Italy | J2a1a1d | 34 | JN202724 | Europe | Sicily | T2h | 36 |
| JQ702950 | Europe | British Isles | J2a1a1 | 35 | JQ705018 |  | Unknown | T2h | 35 |
| JQ797907 | Europe | Italy | J2a1a1a2 | 34 | HM852818 | Near East | Iran | T2h | 2 |
| JQ703638 | Europe | England | J2a1a1a2 | 35 | HM852796 | South Caucasus | Azerbaijan | T2h | 2 |
| JQ702622 |  | Unknown | J2a1a1a2 | 35 | JQ705300 | Europe | Poland | T2 | 35 |
| JQ702772 |  | Unknown | J2a1a1a2 | 35 | JF707633 |  | USA | T2 | 36 |
| JQ703330 |  | Unknown | J2a1a1a2 | 35 | EF660960 | Europe | Italy | T2+16296! | 10 |
| JQ703524 |  | Unknown | J2a1a1a2 | 35 | JF937088 |  | Unknown | T2+16296! | 36 |
| JQ705214 | Europe | England | J2a1a1a2 | 35 | JQ704762 | Europe | England | T2+16296! | 35 |
| JQ703570 | Europe | Czechoslovakia | J2a1a1a2 | 35 | JQ702704 |  | Unknown | T2+16296! | 35 |
| JQ703566 |  | Unknown | J2a1a1a2 | 35 | GQ304741 | Europe | Italy | T2+16296! | 24 |
| JQ703673 | Europe | Sweden | J2a1a1a2 | 35 | JQ704995 | Europe | United Kingdom | T2+16296! | 35 |
| JQ704906 |  | Unknown | J2a1a1a2 | 35 | JQ798139 | Europe | England | T2+16296! | 34 |
| JQ705625 | Europe | Netherlands | J2a1a1a2 | 35 | JQ798136 | Europe | Greece | T2m | 34 |
| JQ705631 | Europe | Germany | J2a1a1a2 | 35 | JQ798134 | Europe | Italy | T2+16296! | 34 |
| JQ705632 |  | Unknown | J2a1a1a2 | 35 | HQ912960 | Europe | Romania | T2l | 36 |
| JQ705535 |  | Unknown | J2a1a1a2 | 35 | JQ798138 | Europe | Adygea Rep. (Russia) | T2l | 34 |
| HQ436101 |  | Unknown | J2a1a1a2 | 36 | JQ798137 | Europe | Adygea Rep. (Russia) | T2 | 34 |
| FJ348157 |  | USA | J2a1a1a2 | 16 | EF177442 | Europe | Portugal | T2j | 13 |
| HQ104930 |  | USA | J2a1a1a2 | 36 | HQ877824 | Europe | Austria | T2j | 36 |
| FJ194438 | Europe | Sweden | J2a1a1a2 | 36 | JQ704786 | Europe | Italy | T2j | 35 |
| EU721734 |  | USA | J2a1a1a2 | 36 | HM852810 | South Caucasus | Azerbaijan | T2k | 2 |
| JF343122 |  | USA | J2a1a1a2 | 36 | JQ705690 | Europe | Ireland | T2k | 35 |
| HQ424014 |  | USA | J2a1a1a2 | 36 | JQ798135 | Europe | Crete | T2 | 34 |
| JQ702027 |  | Unknown | J2a1a1a2 | 35 | HM852766 | South Caucasus | Armenia | T2 | 2 |
| GU903270 |  | Unknown | J2a1a1a | 36 | ADY17 | Europe | Adygea Rep. (Russia) | T2 | 17 |
| HM485691 |  | USA | J2a1a1a | 36 | ADY18 | Europe | Adygea Rep. (Russia) | J1 | 17 |
| JQ705575 |  | Unknown | J2a1a1a | 35 | ADY20 | Europe | Adygea Rep. (Russia) | T2 | 17 |
| JQ704947 | Europe | England | J2a1a1a | 35 | ADY29 | Europe | Adygea Rep. (Russia) | T2 | 17 |
| JQ704600 |  | Unknown | J2a1a1a | 35 | ADY38 | Europe | Adygea Rep. (Russia) | T1 | 17 |
| JQ703801 | Europe | Czech Republic | J2a1a1a | 35 | ADY41 | Europe | Adygea Rep. (Russia) | J1 | 17 |
| JQ702561 |  | Unknown | J2a1a1a | 35 | ADY46 | Europe | Adygea Rep. (Russia) | T2 | 17 |
| JQ703568 | Europe | Germany | J2a1a1a3 | 35 | ADY5 | Europe | Adygea Rep. (Russia) | T2 | 17 |
| JQ705323 |  | Unknown | J2a1a1a3 | 35 | MZB17 | North Africa | Algeria | J2 | 17 |
| GU123013 | Europe | Russia | J2a1a1a | 20 | MZB24 | North Africa | Algeria | J2 | 17 |
| JQ797908 | Europe | Lithuania | J2a1a1e | 34 | MZB31 | North Africa | Algeria | T1 | 17 |
| JQ797909 | Near East | Israel | J2a1a1e | 34 | AZB11 | South Caucasus | Azerbaijan | T2 | 17 |
| JQ797910 | Europe | Greece | J2a1a1e | 34 | AZB15 | South Caucasus | Azerbaijan | T1 | 17 |
| JQ705774 | Europe | Poland | J2a1a1e | 35 | AZB8 | South Caucasus | Azerbaijan | T2 | 17 |
| JQ702605 |  | Unknown | J2a1a1e | 35 | AZB9 | South Caucasus | Azerbaijan | T2 | 17 |
| JQ701807 |  | Unknown | J2a1a1 | 35 | B5 | Europe | Bulgaria | J1 | 17 |
| JQ797913 | Europe | Italy | J2a1a1 | 34 | B13 | Europe | Bulgaria | T1 | 17 |
| FJ605154 |  | USA | J2a1a1b | 36 | B2 | Europe | Bulgaria | J1 | 17 |
| JQ705042 |  | Unknown | J2a1a1b | 35 | B8 | Europe | Bulgaria | J2 | 17 |
| JQ705390 |  | Unknown | J2a1a1b | 35 | BUL7096 | Europe | Bulgaria | J1 | 17 |
| DQ523640 | Europe | Sardinia | J2a1a1 | 15 | BUL7102 | Europe | Bulgaria | T1 | 17 |
| DQ341089 | Europe | Italy | J2a1a2 | 33 | BUL7107 | Europe | Bulgaria | T2 | 17 |
| JQ797914 | Europe | Greece | J2a1a2a | 34 | BUL7116 | Europe | Bulgaria | J1 | 17 |
| JQ702364 | Europe | Italy | J2a1a2a | 35 | BUL-HJB3 | Europe | Bulgaria | J1 | 17 |
| JQ797915 | Arabian Peninsula | Yemen | J2a2a1a | 34 | SE1013 | Europe | Canary Islands | J1 | 17 |
| JQ797916 | Arabian Peninsula | Yemen | J2a2a1a | 34 | SE145 | Europe | Canary Islands | J2 | 17 |
| JQ797917 | Arabian Peninsula | Kuwait | J2a2a1a1a | 34 | SE155 | Europe | Canary Islands | T2 | 17 |
| JQ797918 | Near East | Iraq | J2a2a1a1a | 34 | CHE9 | Europe | Chechen Rep. (Russia) | T2 | 17 |
| JQ797919 | Near East | Iraq | J2a2a1a1a | 34 | CHV44 | Europe | Chuvashia Rep. (Russia) | T2 | 17 |
| JQ797920 | Near East | Israel | J2a2a1a1 | 34 | CZE377 | Europe | Czech Republic | J1b | 17 |
| JQ797921 | Arabian Peninsula | Kuwait | J2a2a1a | 34 | CZE235 | Europe | Czech Republic | J1 | 17 |
| JQ797922 | Europe | England | J2a2a | 34 | CZE236 | Europe | Czech Republic | J1 | 17 |
| JQ797923 | Europe | Italy | J2a2a2 | 34 | CZE238 | Europe | Czech Republic | T2 | 17 |
| GU065327 |  | Unknown | J2a2a2 | 26 | CZE245 | Europe | Czech Republic | J1 | 17 |
| EF660967 | Europe | Italy | J2a2a | 10 | CZE254 | Europe | Czech Republic | T1 | 17 |
| JQ797924 | Europe | Italy | J2a2 | 34 | CZE257 | Europe | Czech Republic | J1 | 17 |
| JQ797925 | Europe | Italy | J2a2c | 34 | CZE268 | Europe | Czech Republic | T1 | 17 |
| JQ797926 | Europe | Italy | J2a2c1a | 34 | CZE316 | Europe | Czech Republic | T2 | 17 |
| JQ797927 | Europe | Italy | J2a2c1a | 34 | CZE373 | Europe | Czech Republic | J2 | 17 |
| JQ797928 | Arabian Peninsula | Yemen | J2a2c1b | 34 | DRZ13 | Near East | Israel-Druze | J2 | 17 |
| JQ797929 | North Africa | Morocco | J2a2b1a | 34 | DRZ16 | Near East | Israel-Druze | T1 | 17 |
| FJ460543 | North Africa | Tunisia | J2a2b1a | 6 | DRZ42 | Near East | Israel-Druze | J2 | 17 |
| JQ797930 | North Africa | Morocco | J2a2b1c | 34 | DRZ6 | Near East | Israel-Druze | J1 | 17 |
| JQ797931 | Europe | Greece | J2a2b2 | 34 | CE70 | Europe | England | J1 | 17 |
| JQ703605 | Europe | United Kingdom | J2a2b2 | 35 | NE665 | Europe | England | J1 | 17 |
| JQ797932 | Europe | Sardinia | J2a2b | 34 | NE707 | Europe | England | J1 | 17 |
| JQ797933 | Arabian Peninsula | Yemen | J2a2b | 34 | NW119 | Europe | England | J1 | 17 |
| JQ797934 | Europe | Canary Islands | J2a2d1 | 34 | NW644 | Europe | England | J1 | 17 |
| FJ460559 | North Africa | Tunisia | J2a2d1 | 6 | NW721 | Europe | England | J1 | 17 |
| JQ797935 | North Africa | Algeria | J2a2d2 | 34 | SC207 | Europe | England | J1 | 17 |
| GQ249257 |  | USA | J2b1a2 | 36 | SE354 | Europe | England | J1 | 17 |
| JQ797936 |  | Unknown | J2b1a2a | 34 | SE733 | Europe | England | J1 | 17 |
| JF938916 | Europe | Portugal | J2b1a2a | 36 | SE870 | Europe | England | J1 | 17 |
| JQ797937 | Europe | Italy | J2b1a2 | 34 | WA64 | Europe | England | J1 | 17 |
| JQ703587 |  | Unknown | J2b1a2 | 35 | FRA-CE23 | Europe | France | T1 | 17 |
| JQ797938 | Europe | Italy | J2b1a2 | 34 | FRA-NF258 | Europe | France | J2 | 17 |
| JQ705677 |  | Unknown | J2b1a2 | 35 | FRA-NF263 | Europe | France | T2 | 17 |
| JQ705925 |  | Unknown | J2b1a | 35 | FRA-NF298 | Europe | France | J1 | 17 |
| JQ702593 | Europe | England | J2b1a | 35 | FRA-NF331 | Europe | France | T2 | 17 |
| EU673448 |  | Unknown | J2b1a | 36 | FRA-NF345 | Europe | France | J1 | 17 |
| JQ797939 | Europe | Italy | J2b1a | 34 | FRA-NF351 | Europe | France | T2 | 17 |
| JQ702199 |  | Unknown | J2b1a | 35 | FRA-NF66 | Europe | France | T2 | 17 |
| JQ797940 | Europe | Italy | J2b1a3 | 34 | HEL-T062 | Europe | Greece | T2 | 17 |
| JQ702702 |  | Unknown | J2b1a3 | 35 | HEL-T066 | Europe | Greece | T1 | 17 |
| JQ702858 | Europe | Italy | J2b1a3 | 35 | IRL42 | Europe | Ireland | T2 | 17 |
| JQ797941 | Europe | Greece | J2b1a3 | 34 | IRL7 | Europe | Ireland | J1 | 17 |
| AY195778 |  | Unknown | J2b1a3 | 23 | IRL868 | Europe | Ireland | J2 | 17 |
| JQ797942 | North Africa | Morocco | J2b1a | 34 | KAB12 | Europe | Kabardino-Balkaria Rep. (Russia) | J1 | 17 |
| JQ797943 | Europe | Russia | J2b1a6 | 34 | KAB31 | Europe | Kabardino-Balkaria Rep. (Russia) | J1 | 17 |
| JQ704523 | Europe | Poland | J2b1a6 | 35 | KUR71 |  | Kurd | T1 | 17 |
| JQ797944 | Europe | Moldova | J2b1a6 | 34 | J14 | North Africa | Morocco | T2 | 17 |
| DQ523671 | Europe | Sardinia | J2b1a7 | 15 | J41 | North Africa | Morocco | J1 | 17 |
| EU597520 | Europe | Sardinia | J2b1a7 | 27 | J5 | North Africa | Morocco | T1 | 17 |
| JQ797945 | Europe | Italy | J2b1a5 | 34 | J50 | North Africa | Morocco | J1 | 17 |
| JQ797946 | Europe | Italy | J2b1a5 | 34 | J54 | North Africa | Morocco | J1 | 17 |
| DQ523653 | Europe | Sardinia | J2b1a5 | 15 | J58 | North Africa | Morocco | J2 | 17 |
| JF915700 | Europe | Ireland | J2b1a | 36 | J60 | North Africa | Morocco | J2 | 17 |
| JQ702346 |  | Unknown | J2b1a4 | 35 | J76 | North Africa | Morocco | J2 | 17 |
| JQ705466 |  | Unknown | J2b1a4 | 35 | NOS30 | North Ossetia | Alania Rep. (Russia) | T1 | 17 |
| JQ703538 |  | Unknown | J2b1a4 | 35 | NOS51 | North Ossetia | Alania Rep. (Russia) | J2 | 17 |
| JQ703515 |  | Unknown | J2b1a4 | 35 | NOS9 | North Ossetia | Alania Rep. (Russia) | T2 | 17 |
| JQ702196 |  | Unknown | J2b1a | 35 | PAL-AO1036 | Near East | Palestine | J1 | 17 |
| JQ702758 |  | Unknown | J2b1a | 35 | PAL-AO1056 | Near East | Palestine | J1 | 17 |
| JQ702477 | Europe | England | J2b1a | 35 | PAL-AO530 | Near East | Palestine | J1 | 17 |
| DQ341090 | Europe | Italy | J2b1a | 33 | PAL-AO549 | Near East | Palestine | T2 | 17 |
| JQ797947 | Europe | Italy | J2b1a | 34 | PAL-AO593 | Near East | Palestine | T1 | 17 |
| JQ705623 | Europe | England | J2b1a | 35 | PAL-AO647 | Near East | Palestine | T2 | 17 |
| JQ702442 | Europe | United Kingdom | J2b1a | 35 | PAL-AO672 | Near East | Palestine | T2 | 17 |
| JQ702553 |  | Unknown | J2b1a | 35 | PAL-AO783 | Near East | Palestine | J1 | 17 |
| JQ703517 |  | Unknown | J2b1a | 35 | PAL-AO841 | Near East | Palestine | J1 | 17 |
| JQ705021 | Europe | Italy | J2b1a | 35 | PAL-AO947 | Near East | Palestine | T2 | 17 |
| FJ445408 | Europe | Ireland | J2b1a1a | 36 | X0000 | Europe | Portugal | J2 | 17 |
| FJ380056 |  | Unknown | J2b1a1a | 36 | X1384 | Europe | Portugal | J1 | 17 |
| JQ702863 |  | Unknown | J2b1a1a | 35 | X2342 | Europe | Portugal | T2 | 17 |
| JQ705356 |  | Unknown | J2b1a1 | 35 | X2881 | Europe | Portugal | T2 | 17 |
| JQ703580 | Europe | France | J2b1a1 | 35 | X3305 | Europe | Portugal | T2 | 17 |
| JQ705064 | Europe | Finland | J2b1 | 35 | X3368 | Europe | Portugal | J1 | 17 |
| JQ702424 |  | Unknown | J2b1 | 35 | X3590 | Europe | Portugal | J1 | 17 |
| JQ797949 | Near east | Syria | J2b1 | 34 | X369 | Europe | Portugal | T2 | 17 |
| EU862198 | Europe | Ireland | J2b1b | 36 | X3691 | Europe | Portugal | J1 | 17 |
| JQ703784 | Europe | Netherlands | J2b1b | 35 | X4059 | Europe | Portugal | J1 | 17 |
| JQ702042 |  | Unknown | J2b1b | 35 | X4062 | Europe | Portugal | T2 | 17 |
| JQ797950 | Europe | Greece | J2b1c | 34 | X4064 | Europe | Portugal | T2 | 17 |
| JQ797951 | Europe | Greece | J2b1c | 34 | X4084 | Europe | Portugal | J1 | 17 |
| JQ797952 | Europe | Greece | J2b1c | 34 | X4118 | Europe | Portugal | T2 | 17 |
| EU807741 |  | Siberia | J2b1c1 | 25 | X4229 | Europe | Portugal | T2 | 17 |
| JQ702563 |  | Unknown | J2b1 | 35 | X4381 | Europe | Portugal | J1 | 17 |
| JQ797953 | Europe | Italy | J2b1 | 34 | X4644 | Europe | Portugal | J1 | 17 |
| JQ797954 | Europe | Italy | J2b1 | 34 | X4646 | Europe | Portugal | T2 | 17 |
| JQ797955 | Europe | Italy | J2b1 | 34 | X5399 | Europe | Portugal | T1 | 17 |
| JQ797956 | Europe | Italy | J2b1 | 34 | X5459 | Europe | Portugal | J2 | 17 |
| JQ797957 | Europe | Italy | J2b1d1 | 34 | X5516 | Europe | Portugal | T2 | 17 |
| JQ797958 | Europe | Russia | J2b1d1 | 34 | X5577 | Europe | Portugal | J1 | 17 |
| JQ797959 | Near East | Syria | J2b1d1 | 34 | X5591 | Europe | Portugal | J2 | 17 |
| JQ797960 | Europe | Italy | J2b1d | 34 | X5595 | Europe | Portugal | T2 | 17 |
| FJ445409 |  | USA | J2b1d | 36 | X5679 | Europe | Portugal | T1 | 17 |
| JQ797961 | North Africa | Algeria | J2b1 | 34 | X5688 | Europe | Portugal | T1 | 17 |
| JQ797962 | Europe | Italy | J2b1 | 34 | X5703 | Europe | Portugal | T1 | 17 |
| JQ797963 | Europe | Slovakia | J2b1 | 34 | ROM-MA66 | Europe | Romania | J1 | 17 |
| JQ702459 |  | Unknown | J2b1 | 35 | ROM-MA68 | Europe | Romania | T1 | 17 |
| JQ702488 |  | Unknown | J2b1 | 35 | ROM-MA7 | Europe | Romania | T1 | 17 |
| JQ701981 |  | Unknown | J2b1e | 35 | ROM-MA70 | Europe | Romania | J1 | 17 |
| JQ797964 | Europe | Italy | J2b1e1 | 34 | ROM-MA8 | Europe | Romania | T1 | 17 |
| JQ797965 | Near East | Lebanon | J2b1e1 | 34 | ROM-MA81 | Europe | Romania | T2 | 17 |
| JQ797966 | North Ossetia | Alania Rep. (Russia) | J2b1 | 34 | ROM-MA90 | Europe | Romania | J1 | 17 |
| JQ797967 | Europe | Kabardino-Balkaria Rep. (Russia) | J2b1 | 34 | ROM-VR203 | Europe | Romania | T1 | 17 |
| JQ797968 | Near East | Syria | J2b1f | 34 | ROM-VR210 | Europe | Romania | J1 | 17 |
| HQ727682 | South Caucasus | Armenia | J2b1f | 36 | ROM-VR224 | Europe | Romania | T2 | 17 |
| EF556152 | South Caucasus | Azerbaijan Jew | J2b1f | 1 | ROM-VR230 | Europe | Romania | J1 | 17 |
| JQ797969 | Anatolia | Turkey | J2b1f | 34 | ROM-VR236 | Europe | Romania | T1 | 17 |
| JQ797970 | Anatolia | Turkey | J2b1f | 34 | ROM-VR238 | Europe | Romania | T2 | 17 |
| JQ797971 | North Africa | Egypt | J2b1 | 34 | RUS7 | Europe | Russia | T2 | 17 |
| JQ797948 | Europe | Italy | J2b1 | 34 | SAR-SS110 | Europe | Sardinia | T2 | 17 |
| JQ797972 | Europe | Romania | J2b2 | 34 | SAR-SS133 | Europe | Sardinia | T2 | 17 |
| JQ797973 | Near East | Iran | J2b2 | 34 | SAR-SS56 | Europe | Sardinia | J1 | 17 |
| JQ797974 | Europe | Albania | J2b2 | 34 | SAR-SS64 | Europe | Sardinia | J2 | 17 |
| FJ213765 |  | USA | J2b2 | 36 | SAR-SS77 | Europe | Sardinia | T2 | 17 |
| HQ889848 |  | USA | J2b2 | 36 | SAR-SS88 | Europe | Sardinia | T2 | 17 |
| JQ797975 | South Caucasus | Azerbaijan | T1 | 34 | SIC-TO509 | Europe | Sicily | T2 | 17 |
| JQ797976 | Near East | Iraq | T1+16189 | 34 | SIC-TO515 | Europe | Sicily | T1 | 17 |
| JQ797977 | North Africa | Morocco | T1a1a1 | 34 | SIC-TO582 | Europe | Sicily | J1 | 17 |
| JQ797978 | Europe | Estonia | T1a1a1 | 34 | SIC-TO608 | Europe | Sicily | T2 | 17 |
| JQ797979 | Europe | Sweden | T1a1a1 | 34 | SIC-TR129 | Europe | Sicily | T2 | 17 |
| JQ797980 | Europe | Kabardino-Balkaria Rep. (Russia) | T1a1a1 | 34 | SIC-TR21 | Europe | Sicily | T2 | 17 |
| JQ797981 | Anatolia | Turkey | T1a1a1 | 34 | SIC-TR26 | Europe | Sicily | J2 | 17 |
| AY495293 |  | USA | T1a1a1 | 37 | SIC-TR4 | Europe | Sicily | T1 | 17 |
| AY495289 |  | USA | T1a1a1 | 37 | SIC-TR51 | Europe | Sicily | J1 | 17 |
| AF382006 | Europe | Iberia | T1a1a1 | 5 | SIC-TR56 | Europe | Sicily | J | 17 |
| HQ167734 | Europe | Ukraine | T1a1a1 | 36 | SUD78 | North Africa | Sudan | T2 | 17 |
| GU122980 | Europe | Russia | T1a1a1 | 20 | SYR-SJS17 | Near east | Syria | T2 | 17 |
| GU361780 |  | USA | T1a1a1 | 36 | SYR-SJS20 | Near east | Syria | J1 | 17 |
| JN880467 | Europe | Ireland | T1a1a1 | 36 | SYR-SJS8 | Near east | Syria | T2 | 17 |
| JQ704249 | Europe | Belarus | T1a1a1 | 35 | SYR-SJSb11 | Near east | Syria | T2 | 17 |
| JQ704735 |  | Unknown | T1a1a1 | 35 | SYR-SJSb2 | Near east | Syria | T1 | 17 |
| JQ705463 |  | Unknown | T1a1a1 | 35 | SYR-SJSb33 | Near east | Syria | T2 | 17 |
| JQ705353 |  | Unknown | T1a1a1 | 35 | SYR-SJSb41 | Near east | Syria | T2 | 17 |
| JQ702716 | Europe | Ireland | T1a1a1 | 35 | AL21 | North Africa | Tunisia | J2 | 17 |
| JQ702556 | Europe | England | T1a1a1 | 35 | AL23 | North Africa | Tunisia | T2 | 17 |
| JQ702340 |  | Unknown | T1a1a1 | 35 | AL34 | North Africa | Tunisia | T2 | 17 |
| JQ797982 | North Africa | Morocco | T1a1a1f | 34 | AL35 | North Africa | Tunisia | T1 | 17 |
| JQ797983 | Europe | Greece | T1a1a1f | 34 | AL43 | North Africa | Tunisia | T1 | 17 |
| JQ702643 |  | Unknown | T1a1a1f | 35 | AL8 | North Africa | Tunisia | T2 | 17 |
| JQ797984 | North Africa | Morocco | T1a1a1 | 34 | ET42 | North Africa | Tunisia | T1 | 17 |
| JQ797985 | Europe | Italy | T1a1a1 | 34 | K19 | North Africa | Tunisia | T1 | 17 |
| JQ702173 |  | Unknown | T1a1a1 | 35 | K2 | North Africa | Tunisia | J2 | 17 |
| JQ797986 | Europe | Estonia | T1a1a1 | 34 | QL18 | North Africa | Tunisia | T2 | 17 |
| GQ304742 |  | Unknown | T1a1a1 | 53 | R187 | North Africa | Tunisia | T2 | 17 |
| JQ705207 |  | Unknown | T1a1a1 | 35 | R582 | North Africa | Tunisia | J1 | 17 |
| JQ701804 |  | Unknown | T1a1a1 | 35 | SK15 | North Africa | Tunisia | T1 | 17 |
| JQ797987 | Europe | Italy | T1a1a1 | 34 | SK25 | North Africa | Tunisia | J1 | 17 |
| JQ797988 | Europe | Kabardino-Balkaria Rep. (Russia) | T1a1a1 | 34 | SL15 | North Africa | Tunisia | T2 | 17 |
| JQ797989 | North Ossetia | Alania Rep. (Russia) | T1a1a1 | 34 | SL17 | North Africa | Tunisia | T2 | 17 |
| JQ797990 | Europe | Karachay-Cherkessia Rep. (Russia) | T1a1a1 | 34 | SL20 | North Africa | Tunisia | T1 | 17 |
| EU597578 | Europe | French Basque | T1a1a1 | 27 | TR29 | North Africa | Tunisia | J2 | 17 |
| HM184912 |  | USA | T1a1a1b | 36 | TR35 | North Africa | Tunisia | J1 | 17 |
| JQ797991 | Anatolia | Turkey | T1a1a1b | 34 | TR39 | North Africa | Tunisia | T2 | 17 |
| JQ797992 | Europe | Crete | T1a1a1b | 34 | TR40 | North Africa | Tunisia | J2 | 17 |
| JQ702958 | Europe | Ukraine | T1a1a1b | 35 | TR47 | North Africa | Tunisia | T1 | 17 |
| DQ358975 | Europe | Germany | T1a1a1b | 43 | TR48 | North Africa | Tunisia | J1 | 17 |
| JQ797994 | Europe | Italy | T1a1a1b1 | 34 | TR49 | North Africa | Tunisia | J2 | 17 |
| JQ797993 | Near East | Iran | T1a1a1b2 | 34 | TR7 | North Africa | Tunisia | J1 | 17 |
| HM852798 | South Caucasus | Azerbaijan | T1a1a1b3 | 3 | Z12 | North Africa | Tunisia | T2 | 17 |
| JQ797995 | Near East | Iran | T1a1a1b4 | 34 | TUR-AT21 | Anatolia | Turkey | J2 | 17 |
| AY714036 | South Asia | India | T1a1a1b5 | 29 | TUR-AT26 | Anatolia | Turkey | T2 | 17 |
| JQ702784 |  | Unknown | T1a1a1 | 35 | TUR-AT27 | Anatolia | Turkey | J1 | 17 |
| JQ702853 | Europe | Finland | T1a1a1 | 35 | TUR-AT31 | Anatolia | Turkey | T2 | 17 |
| JQ797996 | Anatolia | Turkey | T1a1a1 | 34 | TUR-AT33 | Anatolia | Turkey | T2 | 17 |
| JQ703240 |  | Unknown | T1a1a1 | 35 | TUR-AT51 | Anatolia | Turkey | T2 | 17 |
| JQ797997 | Near East | Iraq | T1a1a1 | 34 | TUR-AT78 | Anatolia | Turkey | T1 | 17 |
| JQ703670 |  | Unknown | T1a1a1 | 35 | TUR-AT80 | Anatolia | Turkey | T1 | 17 |
| JQ705778 | Europe | Scotland | T1a1a1 | 35 | TUR-AT92 | Anatolia | Turkey | T2 | 17 |
| JQ797998 | Near East | Iran | T1a1a1 | 34 | TUR-ATb10 | Anatolia | Turkey | T2 | 17 |
| JQ797999 | Near East | Iran | T1a1a1 | 34 | TUR-ATb13 | Anatolia | Turkey | T2 | 17 |
| JQ798000 | Near East | Iran | T1a1a1 | 34 | TUR-ATb18 | Anatolia | Turkey | T2 | 17 |
| JQ798001 | South Asia | India | T1a1a1 | 34 | TUR-ATb28 | Anatolia | Turkey | T2 | 17 |
| JQ703739 |  | Unknown | T1a1a1 | 35 | TUR-ATb29 | Anatolia | Turkey | T1 | 17 |
| JQ798002 | South Asia | India | T1a1a1 | 34 | TUR-ATb3 | Anatolia | Turkey | J1 | 17 |
| JQ798003 | Europe | Greece | T1a1a1l | 34 | TUR-ATb31 | Anatolia | Turkey | T1 | 17 |
| JQ798004 | Europe | Greece | T1a1a1l | 34 | TUR-ATb36 | Anatolia | Turkey | J1 | 17 |
| JF979131 |  | Unknown | T1a1a1l | 36 | TUR-ATc1 | Anatolia | Turkey | T2 | 17 |
| JQ798005 |  | Siberia | T1a1a1d | 34 | TUR-ATc11 | Anatolia | Turkey | T1 | 17 |
| EU007876 |  | Kchanti | T1a1a1d | 31 | TUR-ATc2 | Anatolia | Turkey | T2 | 17 |
| FJ348197 | Europe | Italy | T1a1a1 | 16 | TUR-ATc8 | Anatolia | Turkey | J1 | 17 |
| JQ798006 | Europe | Sweden | T1a1a1e | 34 | JF905566 | Europe | Ireland | T1a1a1c | 36 |
| FJ480957 | Europe | Sweden | T1a1a1e | 36 | JQ705630 |  | Unknown | T1a1a1c | 35 |
| EF661000 | Europe | Italy | T1a1a1 | 10 | JQ705856 | Europe | Ireland | T1a1a1c | 35 |
| AY714015 | South Asia | India | T1a1a1 | 29 | JQ704801 | Europe | Germany | T1a1a1c | 35 |
| AY495288 |  | USA | T1a1a1 | 37 | JQ703894 |  | Unknown | T1a1a1c | 35 |
| AY495297 |  | USA | T1a1a1 | 37 | JQ702923 |  | Unknown | T1a1a1c | 35 |
| AY495290 |  | USA | T1a1a1 | 37 | JF833038 |  | Unknown | T1a1a1 | 36 |
| EF177406 | Europe | Portugal | T1a1a1 | 13 | JQ702986 | Europe | Scotland | T1a1a1 | 35 |
| JQ702825 |  | Unknown | T1a1a1 | 35 | JQ704732 | Europe | Switzerland | T1a1a1g | 35 |
| AY495292 |  | USA | T1a1a1 | 37 | EU092802 | Arabian Peninsula | Yemen | L6a | 12 |
| JQ705266 |  | Unknown | T1a1a1 | 35 | EU092803 | Arabian Peninsula | Yemen | L6a | 12 |
| AY495291 |  | USA | T1a1a1j | 37 | EU092924 | Arabian Peninsula | Yemen | L6a | 12 |
| JQ702925 | Europe | Greece | T1a1a1j | 35 | EU092748 | Arabian Peninsula | Saudi Arabia | L4a1a | 12 |
| JQ703446 |  | Unknown | T1a1a1j | 35 | EU092799 | Arabian Peninsula | Yemen | L4a2 | 12 |
| EU367994 |  | Unknown | T1a1a1 | 36 | EU092800 | Arabian Peninsula | Yemen | L4a2 | 12 |
| AY495294 |  | USA | T1a1a1a1 | 37 | EU092808 | Arabian Peninsula | Yemen | L4b1 | 12 |
| EU882063 |  | USA | T1a1a1a1 | 36 | EU092750 | Arabian Peninsula | Saudi Arabia | L4b2a2 | 12 |
| JQ703032 | Europe | Switzerland | T1a1a1a1 | 35 | EU092780 | Arabian Peninsula | Kuwait | L4b2a2b | 12 |
| JQ705982 |  | Unknown | T1a1a1a | 35 | EU092743 | Near East | Syria | L4b2a2a | 12 |
| JQ703395 | Europe | Scotland | T1a1a1 | 35 | EU092773 | North Africa | Egypt | L6a | 12 |
| AY495295 |  | USA | T1a1a1 | 37 | DQ341063 | North Africa | Ethiopia | L6b | 8 |
| GU944473 |  | USA | T1a1a1 | 36 | EU092935 | North Africa | Ethiopia | L4a2 | 12 |
| JQ705441 | Europe | Czech Republic | T1a1a1i | 35 | EU092949 | North Africa | Ethiopia | L4a2 | 12 |
| JQ705542 |  | Unknown | T1a1a1i | 35 | FJ460531 | North Africa | Tunisia | L4a1 | 6 |
| JN021256 |  | Unknown | T1a1a1i | 36 | EU092662 |  | Israel; Ethiopian Jew | L4b2a2a | 12 |
| JN127414 |  | Unknown | T1a1a1i | 36 | DQ341064 | North Africa | Ethiopia | L4a1a | 8 |
| JF929200 | Europe | England | T1a1a1 | 36 | EU092938 | North Africa | Ethiopia | L4b2a2a | 12 |
| JQ702988 | Europe | British Isles | T1a1a1 | 35 | EU092942 | North Africa | Ethiopia | L4b2a1 | 12 |
| JQ702343 |  | Unknown | T1a1a1 | 35 | EU092951 | North Africa | Ethiopia | L4b2a2b | 12 |
| JF905569 |  | Unknown | T1a1a1 | 36 | DQ341065 | North Africa | Ethiopia | L4b2a1 | 8 |
| JF833040 | Europe | British Isles | T1a1a1g | 36 | EU092838 | South Africa | South Africa; San | L4b2a2 | 12 |
| JF837819 | Europe | Finland | T1a1a1 | 36 | JQ044811 | Western Africa | Burkina Faso | L4b1 | 7 |
| JN089342 | Europe | England | T1a1a1h | 36 | JQ044834 | Western Africa | Burkina Faso | L4b1 | 7 |
| JQ702680 |  | Unknown | T1a1a1h | 35 | JQ044848 | Western Africa | Burkina Faso | L4b1 | 7 |
| JF905570 |  | USA | T1a1a1k | 36 | JQ045081 | Western Africa | Burkina Faso | L4b1 | 7 |
| JF926125 |  | Unknown | T1a1a1k | 36 | EU092673 |  | Israel; Ethiopian Jew | L6b | 12 |
| JQ702959 |  | Unknown | T1a1a1k | 35 | EU092686 |  | Israel; Yemenite Jew | L6b | 12 |
| JQ705707 |  | Unknown | T1a1a1k | 35 | EU092678 |  | Israel; Yemenite Jew | L4a1a | 12 |
| JQ702728 |  | Unknown | T1a1a1k | 35 | HM771233 |  | Pygmy | L4b2b | 3 |
| JF830642 | Europe | England | T1a1a1 | 36 | JQ702504 |  | Africa | L4b2b | Family Tree DNA |

References:

1. Behar DM, et al. (2008) Counting the founders: the matrilineal genetic ancestry of the Jewish Diaspora. PLoS One 3, e2062.

2. Schönberg A, et al. (2011) High-throughput sequencing of complete human mtDNA genomes from the Caucasus and West Asia: high diversity and demographic inferences. Eur J Hum Genet 19, 988-94.

3. Batini C, et al. (2011) Insights into the demographic history of African Pygmies from complete mitochondrial genomes. Mol Biol Evol 28, 1099-110.

4. Kujanová M, et al. (2009) Near eastern Neolithic genetic input in a small oasis of the Egyptian Western Desert. Am J Phys Anthropol 140, 336-46.

5. Maca-Meyer N, et al. (2001) Major genomic mitochondrial lineages delineate early human expansions. BMC Genet 2, 13.

6. Costa MD, et al. (2009) Data from complete mtDNA sequencing of Tunisian centenarians: testing haplogroup association and the "golden mean" to longevity. Mech Ageing Dev 130, 222-6.

7. Barbieri C, et al. (2012) Contrasting maternal and paternal histories in the linguistic context of Burkina Faso. Mol Biol Evol 29, 1213-23.

8. Torroni A, et al. (2006) Harvesting the fruit of the human mtDNA tree. Trends Genet 22, 339-45.

9. Fendt L, et al. (2008) Sequencing strategy for the whole mitochondrial genome resulting in high quality sequences. BMC Genomics 10, 139.

10. Gasparre G, et al. (2007) Disruptive mitochondrial DNA mutations in complex I subunits are markers of oncocytic phenotype in thyroid tumors. Proc Natl Acad Sci U S A 104, 9001-6.

11. Zaragoza MV, et al. (2010) Mitochondrial DNA variant discovery and evaluation in human Cardiomyopathies through next-generation sequencing. PLoS One 5, e12295.

12. Behar DM, et al. (2008) The dawn of human matrilineal diversity. Am J Hum Genet 82, 1130-40.

13. Pereira L, et al. (2007) No evidence for an mtDNA role in sperm motility: data from complete sequencing of asthenozoospermic males. Mol Biol Evol 24, 868-74.

14. Derenko M, et al. (2007) Phylogeographic analysis of mitochondrial DNA in northern Asian populations. Am J Hum Genet 81, 1025-41.

15. Fraumene C, et al. (2006) High resolution analysis and phylogenetic network construction using complete mtDNA sequences in Sardinian genetic isolates. Mol Biol Evol 23, 2101-11.

16. Pichler I, et al. (2010) Drawing the history of the Hutterite population on a genetic landscape: inference from Y-chromosome and mtDNA genotypes. Eur J Hum Genet 18, 463-70.

17. Pereira JB (2013) Genetic characterisation of modern human dispersals in the Greater Mediterranean. PhD Thesis submitted in the University of Leeds.

18. La Morgia C, et al. (2008) Rare mtDNA variants in Leber hereditary optic neuropathy families with recurrence of myoclonus. Neurology 70, 762-770.

19. Li SB. (Direct Submission).

20. Malyarchuk B, et al. (2010b) Mitogenomic diversity in Tatars from the Volga-Ural region of Russia. Mol Biol Evol 27, 2220-6.

21. Rani DS, et al. (2010) Mitochondrial DNA haplogroup 'R' is associated with Noonan syndrome of south India. Mitochondrion 10, 166-173.

22. Rogaev EI, et al. (2009) Genomic identification in the historical case of the Nicholas II royal family. Proc Natl Acad Sci U S A 106, 5258-5263.

23. Mishmar D, et al. (2003) Natural selection shaped regional mtDNA variation in humans. Proc Natl Acad Sci USA 100, 171-6.

24. Ghelli A, et al. (2009) The background of mitochondrial DNA haplogroup J increases the sensitivity of Leber's hereditary optic neuropathy cells to 2,5-hexanedione toxicity. PLoS One 4, e7922.

25. Brown DT, et al. (2001) Random genetic drift determines the level of mutant mtDNA in human primary oocytes. Am J Hum Genet 68, 533-536.

26. Desquiret V, et al. (Direct Submission).

27. Hartmann A, et al. (2008) Validation of microarray-based resequencing of 93 worldwide mitochondrial genomes. Hum Mutat 30, 115-22.

28. Gonder MK, et al. (2007) Whole-mtDNA genome sequence analysis of ancient African lineages. Mol Biol Evol 24, 757-768.

29. Palanichamy MG, et al. (2004) Phylogeny of mitochondrial DNA macrohaplogroup N in India, based on complete sequencing: implications for the peopling of South Asia. Am J Hum Genet 75, 966-78.

30. Amati-Bonneau. (Direct submission).

31. Ingman M, et al. (2007) A recent genetic link between Sami and the Volga-Ural region of Russia. Eur J Hum Genet 15, 115-120.

32. Pope AM, et al. (2011) Mitogenomic and microsatellite variation in descendants of the founder population of Newfoundland: high genetic diversity in an historically isolated population. Genome 54, 110-119.

33. Carelli V, et al. (2006) Haplogroup effects and recombination of mitochondrial DNA: novel clues from the analysis of Leber hereditary optic neuropathy pedigrees. Am J Hum Genet 78, 564-574.

34. Pala M, et al. (2012) Mitochondrial DNA signals of Late Glacial recolonization of Europe from Near Eastern refugia. Am J Hum Genet 90, 915-924.

35. Behar DM, et al. (2012) The Basque paradigm: genetic evidence of a maternal continuity in the Franco-Cantabrian region since pre-Neolithic times. Am J Hum Genet 90, 486-493.

36. Greenspan, T. FTDNA Direct submission. GenBank.

37. Coble MD, et al. (2004) Single nucleotide polymorphisms over the entire mtDNA genome that increase the power of forensic testing in Caucasians. Int J Legal Med 118, 137-146.

38. Pello R, et al. (2008) Mitochondrial DNA background modulates the assembly kinetics of OXPHOS complexes in a cellular model of mitochondrial disease. Hum Mol Genet 17, 4001-4011.

39. Just RS, et al. (2008) Complete mitochondrial genome sequences for 265 African American and U.S. "Hispanic" individuals. Forensic Sci Int Genet 2, e45-48.

40. Malyarchuk BA, et al. (2008) Mitochondrial DNA variability in Slovaks, with application to the Roma origin. Ann Hum Genet 72, 228-240.

41. Detjen AK, et al. (2007) Analysis of mitochondrial DNA in discordant monozygotic twins with neurofibromatosis type 1. Twin Res Hum Genet 10, 486-495.

42. Fendt L, et al. (2011) Accumulation of mutations over the entire mitochondrial genome of breast cancer cells obtained by tissue microdissection. Breast Cancer Res Treat 128, 327-336.

43. Detjen AK. (direct submission).

44. Ingman M, et al. (2000) Mitochondrial genome variation and the origin of modern humans. Nature 408, 708-713.

45. Zsurka. (Direct submission).
