# Supplementary material for: Genetic Stratigraphy of Key Demographic Events in Arabia
Source: PLoS One. 2015 Mar 4;10(3):e0118625. doi: 10.1371/journal.pone.0118625 (PMC4349752; doi:10.1371/journal.pone.0118625)
Supplement: S3 Table — (DOCX) [file pone.0118625.s041.docx]

**S3_Table** Diversity values of ρ and π used for the interpolation maps of the haplogroups J, T and L4.

| **Geographic region** | **Haplogroup J** | | **Haplogroup T** | | **Haplogroup L4** | |
| --- | --- | --- | --- | --- | --- | --- |
|  | **ρ** | π | **ρ** | π | **ρ** | π |
| Algeria | 0.0000 | 0.0000 | 0.0000 | 0.0000 | 4.2000 | 6.3700 |
| Armenia | 2.6500 | 4.0050 | 3.4884 | 4.6420 | 0.0000 | 0.0000 |
| Cameroon | 0.0000 | 0.0000 | 0.0000 | 0.0000 | 4.9750 | 7.3920 |
| UAE | 3.3333 | 3.8940 | 3.7500 | 6.4000 | 0.0000 | 0.0000 |
| Egypt | 2.9189 | 3.5980 | 3.7692 | 5.2500 | 0.0000 | 0.0000 |
| Ethiopia | 2.4000 | 3.9110 | 3.3333 | 5.2310 | 3.6694 | 6.0380 |
| France | 1.4752 | 2.7920 | 2.2069 | 3.6000 | 0.0000 | 0.0000 |
| Germany | 1.6989 | 3.1350 | 2.2761 | 3.6410 | 0.0000 | 0.0000 |
| Greece | 1.3053 | 2.3970 | 2.7000 | 4.2450 | 0.0000 | 0.0000 |
| Hungary | 1.0783 | 2.0660 | 2.7217 | 4.0940 | 0.0000 | 0.0000 |
| Iceland | 2.6567 | 3.8080 | 2.0259 | 3.0970 | 0.0000 | 0.0000 |
| Iraq | 2.8462 | 4.4260 | 2.8571 | 4.5000 | 0.0000 | 0.0000 |
| Iran | 2.8879 | 4.6300 | 3.3393 | 4.8760 | 0.0000 | 0.0000 |
| Israel | 0.0000 | 0.0000 | 0.0000 | 0.0000 | 1.5000 | 4.6670 |
| Italy | 1.6929 | 3.1550 | 2.5661 | 4.4570 | 0.0000 | 0.0000 |
| Kuwait | 2.8197 | 4.0480 | 3.8077 | 6.1820 | 0.0000 | 0.0000 |
| Libya | 2.3704 | 3.7780 | 2.7333 | 4.5710 | 0.0000 | 0.0000 |
| Morocco | 1.7143 | 3.2320 | 3.3243 | 5.0200 | 0.0000 | 0.0000 |
| Niger | 0.0000 | 0.0000 | 0.0000 | 0.0000 | 2.2500 | 4.4000 |
| North Ossetia | 2.6786 | 3.9870 | 1.8188 | 3.3180 | 0.0000 | 0.0000 |
| Pakistan | 2.2222 | 3.6110 | 1.8333 | 3.6430 | 0.0000 | 0.0000 |
| Russia | 1.5805 | 2.9220 | 2.2476 | 3.4810 | 0.0000 | 0.0000 |
| Saudi Arabia | 2.9386 | 3.9150 | 3.3333 | 4.9840 | 4.1111 | 5.8890 |
| Sierra Leone | 0.0000 | 0.0000 | 0.0000 | 0.0000 | 5.3330 | 8.1670 |
| Spain | 1.7815 | 3.3390 | 2.5260 | 4.1480 | 0.0000 | 0.0000 |
| Sweden | 1.5342 | 2.8930 | 2.2381 | 3.6410 | 0.0000 | 0.0000 |
| Switzerland | 1.4681 | 2.8420 | 2.4085 | 3.7930 | 0.0000 | 0.0000 |
| Syria | 2.1786 | 3.8600 | 3.0959 | 4.6160 | 0.0000 | 0.0000 |
| Turkey ‎ | 2.5088 | 4.1350 | 2.5932 | 4.2570 | 0.0000 | 0.0000 |
| United Kingdom | 1.7326 | 3.1410 | 2.0259 | 3.7860 | 0.0000 | 0.0000 |
| Yemen | 2.1884 | 3.3580 | 2.6800 | 4.6800 | 0.0000 | 0.0000 |
| Zambia | 0.0000 | 0.0000 | 0.0000 | 0.0000 | 3.4000 | 5.3450 |
