# Supplementary material for: Genetic Stratigraphy of Key Demographic Events in Arabia
Source: PLoS One. 2015 Mar 4;10(3):e0118625. doi: 10.1371/journal.pone.0118625 (PMC4349752; doi:10.1371/journal.pone.0118625)
Supplement: S4 Table — (DOCX) [file pone.0118625.s042.docx]

**S4_Table** Frequency values used in the reconstruction of the interpolation maps for the haplogroups J, T, J1d1a and J2a2b.

| **Geographic region** | ***n* total** | **Frequency Haplogroup J** | **Frequency Haplogroup J1d1a** | **Frequency Haplogroup J2a2b** | **Frequency Haplogroup T** |
| --- | --- | --- | --- | --- | --- |
| Abkhazia | 27 | 0.0370 | 0.0000 | 0.0000 | 0.0370 |
| Albania | 102 | 0.0980 | 0.0000 | 0.0000 | 0.1274 |
| Algeria | 47 | 0.1277 | 0.0000 | 0.0000 | 0.0425 |
| Angola | 519 | 0.0019 | 0.0000 | 0.0000 | 0.0000 |
| Armenia | 191 | 0.0838 | 0.0000 | 0.0000 | 0.1152 |
| Austria | 374 | 0.0909 | 0.0000 | 0.0000 | 0.1230 |
| Azerbaijan | 48 | 0.0417 | 0.0000 | 0.0000 | 0.1875 |
| Balkaria | 14 | 0.0000 | 0.0000 | 0.0000 | 0.1429 |
| Bosnia | 144 | 0.0694 | 0.0000 | 0.0000 | 0.0486 |
| Bulgaria | 138 | 0.0725 | 0.0000 | 0.0000 | 0.1015 |
| Burkina Faso | 119 | 0.0000 | 0.0000 | 0.0000 | 0.0000 |
| Cabo verde | 292 | 0.0034 | 0.0000 | 0.0000 | 0.0034 |
| Caucasus | 137 | 0.0291 | 0.0000 | 0.0000 | 0.1165 |
| Central African Republic | 56 | 0.0000 | 0.0000 | 0.0000 | 0.0000 |
| Chad | 118 | 0.0000 | 0.0000 | 0.0000 | 0.0000 |
| Congo | 13 | 0.0000 | 0.0000 | 0.0000 | 0.0000 |
| Croatia | 245 | 0.0735 | 0.0000 | 0.0000 | 0.1143 |
| Cyprus | 91 | 0.0549 | 0.0000 | 0.0000 | 0.0769 |
| Czech Republic | 175 | 0.0914 | 0.0000 | 0.0000 | 0.1200 |
| Denmark | 201 | 0.1244 | 0.0050 | 0.0000 | 0.0846 |
| Druze | 433 | 0.0485 | 0.0000 | 0.0000 | 0.0600 |
| UAE | 249 | 0.1165 | 0.0000 | 0.012 | 0.0321 |
| Egypt | 594 | 0.0606 | 0.0034 | 0.0017 | 0.0976 |
| Estonia | 266 | 0.1053 | 0.0000 | 0.0000 | 0.1015 |
| Ethiopia | 559 | 0.0089 | 0.0054 | 0.0000 | 0.0197 |
| Finland | 661 | 0.0454 | 0.0000 | 0.0000 | 0.0378 |
| France | 1285 | 0.0755 | 0.0000 | 0.0000 | 0.0903 |
| Georgia | 45 | 0.0444 | 0.0000 | 0.0000 | 0.2444 |
| Germany | 1841 | 0.0918 | 0.0000 | 0.0000 | 0.1157 |
| Greece | 706 | 0.0949 | 0.0000 | 0.0057 | 0.0991 |
| Hungary | 211 | 0.0995 | 0.0000 | 0.0000 | 0.1422 |
| Iceland | 985 | 0.1360 | 0.0000 | 0.0000 | 0.1178 |
| Iran | 738 | 0.1450 | 0.0027 | 0.0027 | 0.0759 |
| Iraq | 167 | 0.1557 | 0.0120 | 0.0000 | 0.0838 |
| Ireland | 266 | 0.1090 | 0.0000 | 0.0000 | 0.0789 |
| Israel | 216 | 0.0787 | 0.0093 | 0.0000 | 0.1065 |
| Italy | 1712 | 0.0765 | 0.0012 | 0.0006 | 0.1104 |
| Jordan | 142 | 0.0493 | 0.0000 | 0.0000 | 0.0704 |
| Karachay-Cherkess | 13 | 0.0769 | 0.0000 | 0.0000 | 0.0000 |
| Kenya | 188 | 0.0053 | 0.0000 | 0.0000 | 0.0000 |
| Kurd | 160 | 0.0938 | 0.0000 | 0.0000 | 0.1250 |
| Kuwait | 381 | 0.1549 | 0.0157 | 0.0026 | 0.0682 |
| Latvia | 413 | 0.0605 | 0.0000 | 0.0000 | 0.0920 |
| Libya | 418 | 0.0622 | 0.0239 | 0.0120 | 0.0383 |
| Lithuania | 343 | 0.0641 | 0.0000 | 0.0000 | 0.1020 |
| Macedonia | 308 | 0.0584 | 0.0000 | 0.0000 | 0.1169 |
| Mali | 262 | 0.0191 | 0.0000 | 0.0000 | 0.0000 |
| Mauritania | 94 | 0.0425 | 0.0000 | 0.0000 | 0.0000 |
| Morocco | 1103 | 0.0526 | 0.0018 | 0.0054 | 0.0571 |
| Niger | 165 | 0.0182 | 0.0000 | 0.0000 | 0.0000 |
| Nigeria | 1425 | 0.0035 | 0.0000 | 0.0000 | 0.0014 |
| North Ossetia | 231 | 0.0822 | 0.0000 | 0.0000 | 0.0649 |
| Norway | 305 | 0.0754 | 0.0000 | 0.0000 | 0.0885 |
| Oman | 196 | 0.0765 | 0.0051 | 0.0051 | 0.1020 |
| Pakistan | 189 | 0.0476 | 0.0000 | 0.0000 | 0.0265 |
| Poland | 882 | 0.0782 | 0.0000 | 0.0023 | 0.0952 |
| Portugal | 1612 | 0.0651 | 0.0000 | 0.0006 | 0.0961 |
| Romania | 600 | 0.1050 | 0.0000 | 0.0000 | 0.0883 |
| Russia | 1110 | 0.0586 | 0.0000 | 0.0000 | 0.0703 |
| Rwanda | 42 | 0.0000 | 0.0000 | 0.0000 | 0.0000 |
| Saudi Arabia | 553 | 0.2061 | 0.0326 | 0.0036 | 0.0615 |
| Senegal | 280 | 0.0071 | 0.0000 | 0.0000 | 0.0000 |
| Serbia | 104 | 0.0865 | 0.0000 | 0.0000 | 0.1058 |
| Slovakia | 710 | 0.0958 | 0.0000 | 0.0000 | 0.0930 |
| Slovenia | 232 | 0.0733 | 0.0000 | 0.0000 | 0.0991 |
| Somalia | 183 | 0.0109 | 0.0000 | 0.0000 | 0.0109 |
| South Africa | 637 | 0.0000 | 0.0000 | 0.0000 | 0.0031 |
| Spain | 2062 | 0.0737 | 0.0000 | 0.0000 | 0.0834 |
| Sudan | 178 | 0.0056 | 0.0000 | 0.0000 | 0.0168 |
| Sweden | 634 | 0.0789 | 0.0000 | 0.0000 | 0.0899 |
| Switzerland | 153 | 0.1176 | 0.0000 | 0.0000 | 0.1634 |
| Syria | 116 | 0.0948 | 0.0000 | 0.0000 | 0.1207 |
| Tanzania | 96 | 0.0104 | 0.0104 | 0.0000 | 0.0000 |
| Tunisia | 551 | 0.0563 | 0.0000 | 0.0109 | 0.1034 |
| Turkey | 448 | 0.0938 | 0.0000 | 0.0000 | 0.0870 |
| Uganda | 32 | 0.0000 | 0.0000 | 0.0000 | 0.0000 |
| United Kingdom | 3907 | 0.1211 | 0.0000 | 0.0003 | 0.0906 |
| Ukraine | 18 | 0.0556 | 0.0000 | 0.0000 | 0.1667 |
| West Saharan | 110 | 0.0091 | 0.0000 | 0.0000 | 0.0119 |
| Yemen | 552 | 0.1467 | 0.0199 | 0.0181 | 0.0580 |
