# Supplementary material for: Genetic Stratigraphy of Key Demographic Events in Arabia
Source: PLoS One. 2015 Mar 4;10(3):e0118625. doi: 10.1371/journal.pone.0118625 (PMC4349752; doi:10.1371/journal.pone.0118625)
Supplement: S5 Table — (DOCX) [file pone.0118625.s043.docx]

**S5_Table** Frequency values used in the reconstruction of the interpolation maps for the haplogroups L4 and L6.

| **Geographic region** | ***n* total** | **Frequency Haplogroup L4** | **Frequency Haplogroup L6** |
| --- | --- | --- | --- |
| Saudi Arabia | 553 | 0.0036 | 0.0018 |
| Angola | 519 | 0.0039 | 0.0000 |
| Burkina Faso | 119 | 0.0084 | 0.0000 |
| Cameroon | 737 | 0.0095 | 0.0000 |
| Chad | 118 | 0.0171 | 0.0000 |
| UAE | 249 | 0.0120 | 0.0000 |
| Egypt | 594 | 0.0084 | 0.0000 |
| Ethiopia | 636 | 0.0802 | 0.0189 |
| Gabon | 833 | 0.0048 | 0.0000 |
| Ghana | 238 | 0.0042 | 0.0000 |
| Israel | 216 | 0.0046 | 0.0000 |
| Kenya | 329 | 0.0851 | 0.0000 |
| Kuwait | 381 | 0.0079 | 0.0000 |
| Libya | 527 | 0.0057 | 0.0000 |
| Morocco | 1103 | 0.0027 | 0.0000 |
| Niger | 195 | 0.0102 | 0.0000 |
| Nigeria | 1425 | 0.0182 | 0.0000 |
| Rwanda | 42 | 0.0714 | 0.0000 |
| Somali | 186 | 0.0376 | 0.0161 |
| South Africa | 637 | 0.0078 | 0.0000 |
| Sudan | 178 | 0.0449 | 0.0000 |
| Syria | 116 | 0.0086 | 0.0000 |
| Tanzania | 171 | 0.1696 | 0.0000 |
| Tunisia | 551 | 0.0018 | 0.0018 |
| Turkana | 24 | 0.1250 | 0.0000 |
| Yemen | 552 | 0.0072 | 0.0235 |
| Zambia | 78 | 0.0256 | 0.0000 |
| Zimbabwe | 59 | 0.0169 | 0.0000 |
