# Supplementary material for: Genetic Stratigraphy of Key Demographic Events in Arabia
Source: PLoS One. 2015 Mar 4;10(3):e0118625. doi: 10.1371/journal.pone.0118625 (PMC4349752; doi:10.1371/journal.pone.0118625)
Supplement: S6 Table — (DOCX) [file pone.0118625.s044.docx]

**S6_Table** Founder lineages identified when using *f1* criterion from Near East, Iran and Pakistan to Arabian Peninsula.

| **f1** |  |  | **From Near East, Iran and Pakistan to Arabian Peninsula** | | |
| --- | --- | --- | --- | --- | --- |
| **Clade** | **Founder** | **HVS-I variants (-16,000)** | **n** | **rho** | **se** |
| HV1 | F1 | 67 292 354 | 1 | 0.0000 | 0.0000 |
| H | F2 | 316 | 2 | 1.0000 | 0.7071 |
| HV1a1 | F3 | 67 355 | 2 | 0.0000 | 0.0000 |
| H6b | F4 | 300 362 | 8 | 0.8750 | 0.4841 |
| HV | F5 | 69 | 3 | 0.0000 | 0.0000 |
| H | F6 | 266 | 1 | 0.0000 | 0.0000 |
| H | F7 | 92 | 2 | 0.0000 | 0.0000 |
| HV | F8 | 210 | 1 | 0.0000 | 0.0000 |
| H | F9 | 168 | 2 | 0.0000 | 0.0000 |
| HV2 | F10 | 217 | 5 | 1.4000 | 0.6633 |
| HV | F11 | 114 | 1 | 0.0000 | 0.0000 |
| HV | F12 | 145 | 1 | 0.0000 | 0.0000 |
| H15a1b | F13 | 248 | 1 | 0.0000 | 0.0000 |
| H15a1b | F14 | 319 | 2 | 0.5000 | 0.5000 |
| HV | F15 | 243 | 2 | 0.5000 | 0.5000 |
| HV | F16 | 172 | 1 | 0.0000 | 0.0000 |
| HV | F17 | 355 | 2 | 1.0000 | 0.7071 |
| HV | F18 | 220C 292 | 2 | 0.0000 | 0.0000 |
| HV | F19 | 298 | 1 | 0.0000 | 0.0000 |
| H2a1 | F20 | 354 | 15 | 0.1333 | 0.0943 |
| HV | F21 | 153 | 2 | 0.0000 | 0.0000 |
| H | F22 | 218 | 6 | 0.5000 | 0.2887 |
| HV1 | F23 | 67 | 13 | 1.5385 | 0.6057 |
| HV | F24 | 221 | 1 | 0.0000 | 0.0000 |
| H5 | F25 | 304 | 5 | 1.2000 | 0.4899 |
| H6 | F26 | 362 | 2 | 0.0000 | 0.0000 |
| H | F27 | 261 | 2 | 0.0000 | 0.0000 |
| H1 | F28 | 278 | 2 | 0.5000 | 0.5000 |
| H2a3 | F29 | 274 | 2 | 1.0000 | 0.7071 |
| HV | F30 | 240 | 1 | 0.0000 | 0.0000 |
| HV | F31 | 192 | 4 | 0.0000 | 0.0000 |
| H | F32 | 189 | 2 | 0.0000 | 0.0000 |
| H | F33 | 93 | 2 | 0.0000 | 0.0000 |
| H | F34 | 242 | 1 | 0.0000 | 0.0000 |
| H | F35 | 239 | 4 | 0.0000 | 0.0000 |
| HV | F36 | root | 56 | 0.8036 | 0.1722 |
| M1a3 | F37 | 223 311 | 1 | 0.0000 | 0.0000 |
| M1b1 | F38 | 185 | 2 | 0.5000 | 0.5000 |
| M1a3 | F39 | 223 | 1 | 0.0000 | 0.0000 |
| M1a1 | F40 | 359 | 14 | 0.3571 | 0.1597 |
| M1 | F41 | root | 8 | 1.1250 | 0.4146 |
| N1b1 | F42 | 145 176G 309 390 | 2 | 0.0000 | 0.0000 |
| N1 | F43 | 301 | 3 | 0.0000 | 0.0000 |
| N1a1a1 | F44 | 147G 172 248 355 | 12 | 0.8333 | 0.4082 |
| N1a1a1 | F45 | 147A 172 248 355 | 14 | 2.4286 | 0.8512 |
| N1a3 | F46 | 201 265 | 10 | 0.1000 | 0.1000 |
| N1a3 | F47 | 189 201 265 | 1 | 0.0000 | 0.0000 |
| N1a3 | F48 | 265 | 5 | 2.4000 | 1.3856 |
| N1b1 | F49 | 145 176G 390 | 13 | 0.8462 | 0.3846 |
| N1b1 | F50 | 93 145 176G 390 | 2 | 0.0000 | 0.0000 |
| I5a | F51 | 129 148 391 | 4 | 1.5000 | 0.7071 |
| I1 | F52 | 129 311 391 | 1 | 0.0000 | 0.0000 |
| I | F53 | 129 391 | 10 | 2.4000 | 1.0954 |
| W | F54 | 292 | 7 | 0.7143 | 0.4286 |
| W6a'b | F55 | 192 292 325 | 3 | 1.3333 | 0.6667 |
| W6 | F56 | 292 325 | 3 | 0.3333 | 0.3333 |
| N2a | F57 | 153 319 | 2 | 0.0000 | 0.0000 |
| R0a | F58 | 189 | 9 | 0.1111 | 0.1111 |
| R0a1a | F59 | 355 | 85 | 0.6588 | 0.2445 |
| R0a | F60 | root | 78 | 0.8462 | 0.4205 |
| R0a | F61 | 114 | 1 | 0.0000 | 0.0000 |
| R2 | F62 | 325 355 357 | 1 | 0.0000 | 0.0000 |
| R2c | F63 | 320 | 1 | 0.0000 | 0.0000 |
| R2 | F64 | 234 | 1 | 0.0000 | 0.0000 |
| R2 | F65 | root | 8 | 1.1250 | 0.5154 |
| T2 | F66 | 146 292 296! | 4 | 0.2500 | 0.2500 |
| T2 | F67 | 288 292 296 | 2 | 0.0000 | 0.0000 |
| T2b7a2 | F68 | 239 296 304 | 1 | 0.0000 | 0.0000 |
| T2 | F69 | 93 296 | 2 | 0.5000 | 0.5000 |
| T1b | F70 | 163 189 243 | 2 | 0.0000 | 0.0000 |
| T2b | F71 | 296! 304 | 1 | 0.0000 | 0.0000 |
| T2c1c | F72 | 146 292 296 | 2 | 0.5000 | 0.5000 |
| T2c1 | F73 | 292 296! | 4 | 0.2500 | 0.2500 |
| T1a | F74 | 163 186 189 | 18 | 0.2778 | 0.1242 |
| T2e | F75 | 153 296 | 1 | 0.0000 | 0.0000 |
| T2b7a1 | F76 | 153 257 296! | 5 | 1.2000 | 0.6325 |
| T2b | F77 | 296 304 | 1 | 0.0000 | 0.0000 |
| T2c1 | F78 | 292 296 | 6 | 2.0000 | 0.9718 |
| T2 | F79 | 296! | 2 | 0.0000 | 0.0000 |
| T1 | F80 | 163 189 | 3 | 0.6667 | 0.6667 |
| T2 | F81 | 296 | 9 | 0.8889 | 0.5879 |
| J | F82 | 188 311 | 8 | 1.1250 | 1.0078 |
| J2a1a1 | F83 | 145 231 261 | 1 | 0.0000 | 0.0000 |
| J1b1b2 | F84 | 145 222 235 261 | 2 | 0.0000 | 0.0000 |
| J1d1a | F85 | 193 300 309 | 22 | 0.0455 | 0.0455 |
| J1d1 | F86 | 193 287 300 | 1 | 0.0000 | 0.0000 |
| J1b | F87 | 145 222 261! | 1 | 0.0000 | 0.0000 |
| J1b | F88 | 145 222 261 311 | 3 | 0.0000 | 0.0000 |
| J1b | F89 | 145 222 256 261 278 | 2 | 0.0000 | 0.0000 |
| J1b | F90 | 145 222 261 300 | 5 | 0.6000 | 0.6000 |
| J1d5 | F91 | 193 274 | 1 | 0.0000 | 0.0000 |
| J | F92 | 231 319 | 6 | 0.0000 | 0.0000 |
| J | F93 | 319 | 1 | 0.0000 | 0.0000 |
| J1b | F94 | 126 145 261 | 5 | 0.4000 | 0.2828 |
| J1d1 | F95 | 193 300 | 3 | 0.3333 | 0.3333 |
| J1b | F96 | 145 222 261 | 68 | 0.5294 | 0.2767 |
| J | F97 | 145 | 3 | 0.6667 | 0.6667 |
| J | F98 | 371 | 2 | 0.0000 | 0.0000 |
| J | F99 | 231 | 2 | 0.5000 | 0.5000 |
| J | F100 | 69 | 2 | 1.5000 | 0.8660 |
| J2a2b | F101 | 241 | 9 | 0.7778 | 0.5556 |
| J1d | F102 | 193 | 12 | 0.8333 | 0.6009 |
| J1b | F103 | 145 261 | 32 | 0.5000 | 0.1466 |
| J | F104 | root | 22 | 0.7273 | 0.2727 |
| U5a1 | F105 | 192 256 399 | 2 | 0.5000 | 0.5000 |
| U5a | F106 | 192 256 | 1 | 0.0000 | 0.0000 |
| U5 | F107 | 192 | 3 | 0.6667 | 0.4714 |
| U5a | F108 | 256 | 1 | 0.0000 | 0.0000 |
| U5 | F109 | root | 3 | 0.0000 | 0.0000 |
| U8b1 | F110 | 189 234 257 259 290 | 2 | 0.5000 | 0.5000 |
| U6a2'3 | F111 | 172 189 219 278 | 3 | 0.6667 | 0.4714 |
| U2b | F112 | 51 239 288 353 | 1 | 0.0000 | 0.0000 |
| U1a | F113 | 189 249 288 362 | 1 | 0.0000 | 0.0000 |
| U2e | F114 | 51 129C 189 362 | 9 | 0.5556 | 0.5556 |
| U2d1 | F115 | 51 184 189! 234 294 342 | 1 | 0.0000 | 0.0000 |
| U1 | F116 | 184A 249 355 | 1 | 0.0000 | 0.0000 |
| U6a | F117 | 172 219 278 | 1 | 0.0000 | 0.0000 |
| U2b2 | F118 | 51 353 | 11 | 4.0000 | 1.7953 |
| U7 | F119 | 207 309 318C | 1 | 0.0000 | 0.0000 |
| U7 | F120 | 309 318T 362 | 1 | 0.0000 | 0.0000 |
| U1a'c | F121 | 145 189 249 | 1 | 0.0000 | 0.0000 |
| U6a'b'd | F122 | 172 219 | 2 | 0.0000 | 0.0000 |
| U2 | F123 | 51 247 254 | 1 | 0.0000 | 0.0000 |
| U1a'c | F124 | 189 249 | 7 | 0.8571 | 0.4949 |
| U9a | F125 | 51 129 259 278 | 5 | 0.2000 | 0.2000 |
| U1b | F126 | 111 214A 249 327 | 1 | 0.0000 | 0.0000 |
| K1a4c1 | F127 | 224 246T 311 | 2 | 0.0000 | 0.0000 |
| U7 | F128 | 309 318T | 2 | 0.5000 | 0.5000 |
| U7 | F129 | 309 318C | 1 | 0.0000 | 0.0000 |
| U8b1 | F130 | 189 234 | 1 | 0.0000 | 0.0000 |
| U8b1a1 | F131 | 129 189 234 | 1 | 0.0000 | 0.0000 |
| K | F132 | 192 224 311 | 5 | 0.0000 | 0.0000 |
| U3a | F133 | 343 390 | 9 | 2.1111 | 0.7454 |
| U3b3 | F134 | 168 343 | 2 | 0.5000 | 0.5000 |
| U7 | F135 | 129 318T | 2 | 1.0000 | 0.7071 |
| U4a1 | F136 | 134 356 | 6 | 0.5000 | 0.2887 |
| U3b1a | F137 | 86 343 | 5 | 2.6000 | 1.0000 |
| K1b1a | F138 | 224 311 319 | 2 | 0.0000 | 0.0000 |
| K1 | F139 | 93 224 311 | 13 | 0.0000 | 0.0000 |
| U1 | F140 | 249 | 1 | 0.0000 | 0.0000 |
| U2 | F141 | 51 | 5 | 3.2000 | 1.0583 |
| U9a | F142 | 51 278 | 10 | 0.5000 | 0.5000 |
| U7 | F143 | 318T | 3 | 0.6667 | 0.6667 |
| U4 | F144 | 356 | 5 | 2.6000 | 0.8718 |
| U3 | F145 | 343 | 5 | 0.8000 | 0.4000 |
| U7a4 | F146 | 126 209 309 318T 390 | 1 | 0.0000 | 0.0000 |
| U7a4 | F147 | 126 209 309 318T | 1 | 0.0000 | 0.0000 |
| K | F148 | 224 311 | 33 | 1.2424 | 0.3193 |
| X2j | F149 | 179 | 1 | 0.0000 | 0.0000 |
| X | F150 | 362 | 1 | 0.0000 | 0.0000 |
| X | F151 | 344 | 8 | 0.3750 | 0.2165 |
| X2 | F152 | 248 | 2 | 1.0000 | 0.7071 |
| X | F153 | root | 11 | 0.1818 | 0.1286 |
