# Supplementary material for: Genetic Stratigraphy of Key Demographic Events in Arabia
Source: PLoS One. 2015 Mar 4;10(3):e0118625. doi: 10.1371/journal.pone.0118625 (PMC4349752; doi:10.1371/journal.pone.0118625)
Supplement: S7 Table — (DOCX) [file pone.0118625.s045.docx]

**S7_Table** Founder lineages identified when using *f2* criterion from Near East, Iran and Pakistan to Arabian Peninsula.

| **f2** |  |  | **From Near East, Iran and Pakistan to Arabian Peninsula** | | |
| --- | --- | --- | --- | --- | --- |
| **Clade** | **Founder** | **HVS-I variants (-16,000)** | **n** | **rho** | **se** |
| HV1a1 | F1 | 67 355 | 2 | 0.0000 | 0.0000 |
| H6 | F2 | 300 362 | 8 | 0.8750 | 0.4841 |
| H | F3 | 92 | 2 | 0.0000 | 0.0000 |
| HV2 | F4 | 217 | 5 | 1.4000 | 0.6633 |
| HV | F5 | 114 | 1 | 0.0000 | 0.0000 |
| H15a1b | F6 | 248 | 1 | 0.0000 | 0.0000 |
| HV | F7 | 355 | 2 | 1.0000 | 0.7071 |
| HV | F8 | 298 | 1 | 0.0000 | 0.0000 |
| H2a1 | F9 | 354 | 15 | 0.1333 | 0.0943 |
| H | F10 | 218 | 6 | 0.5000 | 0.2887 |
| HV1 | F11 | 67 | 14 | 1.6429 | 0.5759 |
| H5 | F12 | 304 | 5 | 1.2000 | 0.4899 |
| H6 | F13 | 362 | 2 | 0.0000 | 0.0000 |
| H | F14 | 261 | 2 | 0.0000 | 0.0000 |
| HV | F15 | 192 | 4 | 0.0000 | 0.0000 |
| H | F16 | 189 | 2 | 0.0000 | 0.0000 |
| H | F17 | 93 | 2 | 0.0000 | 0.0000 |
| H | F18 | 239 | 4 | 0.0000 | 0.0000 |
| HV | F19 | root | 82 | 1.0122 | 0.1518 |
| M1a1 | F20 | 359 | 14 | 0.3571 | 0.1597 |
| M1 | F21 | root | 12 | 1.3333 | 0.3909 |
| N1a1a1 | F22 | 147A 172 248 355 | 26 | 2.1538 | 0.6772 |
| N1a3 | F23 | 201 265 | 11 | 0.1818 | 0.1286 |
| N1a3 | F24 | 265 | 5 | 2.4000 | 1.3856 |
| N1b1 | F25 | 145 176G 390 | 17 | 0.8824 | 0.3379 |
| I | F26 | 129 391 | 15 | 2.4667 | 0.8138 |
| N1 | F27 | root | 3 | 0.0000 | 0.0000 |
| W | F28 | 292 | 7 | 0.7143 | 0.4286 |
| N2a | F29 | 153 319 | 2 | 0.0000 | 0.0000 |
| W6a'b | F30 | 192 292 325 | 3 | 1.3333 | 0.6667 |
| W6 | F31 | 292 325 | 3 | 0.3333 | 0.3333 |
| R0a | F32 | 189 | 9 | 0.1111 | 0.1111 |
| R0a1a | F33 | 355 | 85 | 0.6588 | 0.2445 |
| R0a | F34 | 114 | 1 | 0.0000 | 0.0000 |
| R0a | F35 | root | 78 | 0.8462 | 0.4205 |
| R2 | F36 | root | 11 | 1.5455 | 0.4545 |
| T2b | F37 | 296! 304 | 1 | 0.0000 | 0.0000 |
| T2 | F38 | 288 292 296 | 2 | 0.0000 | 0.0000 |
| T2c1c | F39 | 146 292 296 | 6 | 1.0000 | 0.7071 |
| T2c1 | F40 | 292 296 296 | 4 | 0.2500 | 0.2500 |
| T1a | F41 | 163 186 189 | 18 | 0.2778 | 0.1242 |
| T2b | F42 | 296 304 | 2 | 1.0000 | 0.7071 |
| T2c1 | F43 | 292 296 | 6 | 2.0000 | 0.9718 |
| T2 | F44 | 296 296 | 2 | 0.0000 | 0.0000 |
| T1 | F45 | 163 189 | 5 | 0.8000 | 0.5657 |
| T2 | F46 | 296 | 17 | 2.0000 | 0.6707 |
| J2a1a1 | F47 | 145 231 261 | 1 | 0.0000 | 0.0000 |
| J1d1a | F48 | 193 300 309 | 22 | 0.0455 | 0.0455 |
| J1b | F49 | 145 222 256 261 278 | 2 | 0.0000 | 0.0000 |
| J1b1b2 | F50 | 145 222 235 261 | 2 | 0.0000 | 0.0000 |
| J1d1 | F51 | 193 300 | 4 | 0.7500 | 0.4330 |
| J1b | F52 | 145 222 261 | 77 | 0.6364 | 0.2594 |
| J | F53 | 145 | 3 | 0.6667 | 0.6667 |
| J | F54 | 231 | 8 | 0.8750 | 0.7603 |
| J | F55 | 69 | 2 | 1.5000 | 0.8660 |
| J2a2b | F56 | 241 | 9 | 0.7778 | 0.5556 |
| J1d | F57 | 193 | 13 | 0.8462 | 0.5600 |
| J1b | F58 | 145 261 | 37 | 0.6216 | 0.1892 |
| J | F59 | root | 33 | 1.3333 | 0.4635 |
| U5a1 | F60 | 192 256 399 | 2 | 0.5000 | 0.5000 |
| U5a | F61 | 192 256 | 1 | 0.0000 | 0.0000 |
| U5 | F62 | 192 | 3 | 0.6667 | 0.4714 |
| U5a | F63 | 256 | 1 | 0.0000 | 0.0000 |
| U5 | F64 | root | 3 | 0.0000 | 0.0000 |
| U2d1 | F65 | 51 184 189 234 294 342 | 1 | 0.0000 | 0.0000 |
| U2e | F66 | 51 129C 189 362 | 9 | 0.5556 | 0.5556 |
| U6a | F67 | 172 219 278 | 4 | 1.7500 | 0.9014 |
| U5b2c2b | F68 | 189 249 288 | 1 | 0.0000 | 0.0000 |
| U7a4 | F69 | 126 209 309 318T | 2 | 1.5000 | 0.8660 |
| U1a'c | F70 | 189 249 | 8 | 1.2500 | 0.5000 |
| U1b | F71 | 111 249 327 | 1 | 0.0000 | 0.0000 |
| U7 | F72 | 309 318T | 3 | 0.6667 | 0.4714 |
| U7 | F73 | 309 318C 318T | 2 | 1.0000 | 0.7071 |
| U3a | F74 | 343 390 | 9 | 2.1111 | 0.7454 |
| U3b3 | F75 | 168 343 | 2 | 0.5000 | 0.5000 |
| U8b | F76 | 189 234 257 259 | 2 | 2.5000 | 1.5000 |
| U8b1 | F77 | 189 234 | 1 | 0.0000 | 0.0000 |
| K | F78 | 129 189 234 | 1 | 0.0000 | 0.0000 |
| K1 | F79 | 93 224 311 | 13 | 0.0000 | 0.0000 |
| U1 | F80 | 249 | 1 | 0.0000 | 0.0000 |
| U1 | F81 | 249 355 | 1 | 0.0000 | 0.0000 |
| U2 | F82 | 51 | 18 | 4.3333 | 1.3240 |
| U7 | F83 | 318T | 5 | 1.2000 | 0.6325 |
| U4 | F84 | 356 | 5 | 2.6000 | 0.8718 |
| U4a1 | F85 | 134 356 | 6 | 0.5000 | 0.2887 |
| U3 | F86 | 343 | 10 | 2.2000 | 0.7348 |
| K | F87 | 224 311 | 42 | 1.3095 | 0.3095 |
| U | F88 | root | 17 | 3.2941 | 1.3745 |
| X2 | F89 | 248 | 2 | 1.0000 | 0.7071 |
| X | F90 | 344 | 8 | 0.3750 | 0.2165 |
| X | F91 | root | 13 | 0.3077 | 0.1538 |
