# Supplementary material for: Genetic Stratigraphy of Key Demographic Events in Arabia
Source: PLoS One. 2015 Mar 4;10(3):e0118625. doi: 10.1371/journal.pone.0118625 (PMC4349752; doi:10.1371/journal.pone.0118625)
Supplement: S8 Table — (DOCX) [file pone.0118625.s046.docx]

**S8_Table** Founder lineages identified when using *f1* criterion from Near East, Iran and Pakistan to Arabian Peninsula, based on whole-mtDNA JT sequences.

| ***f1*** |  | **From Near East, Iran and Pakistan to Arabian Peninsula** | | |
| --- | --- | --- | --- | --- |
| **Clade** | **Founder** | ***n*** | **ρ** | **se** |
| J1d | F1 | 2 | 4.5000 | 1.5000 |
| J1d1a | F2 | 2 | 4.0000 | 1.4142 |
| J1d1a1 | F3 | 7 | 4.0000 | 1.1429 |
| J1d2_3523 | F4 | 1 | 0.0000 | 0.0000 |
| J1d | F5 | 1 | 0.0000 | 0.0000 |
| J1d2c2 | F6 | 3 | 0.6667 | 0.4714 |
| J1b1b | F7 | 1 | 0.0000 | 0.0000 |
| J1b1b1 | F8 | 2 | 4.5000 | 1.5000 |
| J1b2 | F9 | 8 | 4.6250 | 1.0078 |
| J1b3b_16235! | F10 | 1 | 0.0000 | 0.0000 |
| J1b3 | F11 | 1 | 0.0000 | 0.0000 |
| J1b_152 | F12 | 5 | 2.8000 | 0.7483 |
| J1b6b_9587 | F13 | 1 | 0.0000 | 0.0000 |
| J1b6 | F14 | 1 | 0.0000 | 0.0000 |
| T1a4b | F15 | 2 | 0.5000 | 0.5000 |
| T1a3 | F16 | 1 | 0.0000 | 0.0000 |
| T1a | F17 | 3 | 4.6667 | 1.6330 |
