# Supplementary material for: Genetic Stratigraphy of Key Demographic Events in Arabia
Source: PLoS One. 2015 Mar 4;10(3):e0118625. doi: 10.1371/journal.pone.0118625 (PMC4349752; doi:10.1371/journal.pone.0118625)
Supplement: S9 Table — (DOCX) [file pone.0118625.s047.docx]

**S9_Table** Founder lineages identified when using *f1* criterion from Africa to Arabian Peninsula, Near East and Iran.

| ***f1*** |  |  | **From Africa to Arabian Peninsula and Near East** | | |
| --- | --- | --- | --- | --- | --- |
| **Clade** | **Founder** | **HVS-I variants (-16,000)** | ***n*** | **ρ** | **se** |
| L0a | F1 | 187 188A 278 390 | 1 | 0 | 0 |
|  | F2 | 51 188G 278 320 | 1 | 0 | 0 |
|  | F3 | 114A 168 293 | 1 | 0 | 0 |
|  | F4 | 168 287 293 | 1 | 0 | 0 |
|  | F5 | 168 293 320 | 4 | 0 | 0 |
|  | F6 | 168 278 293 | 7 | 0.5714 | 0.2857 |
|  | F7 | 129 188A 214 234 320 | 1 | 0 | 0 |
|  | F8 | 168 287 | 1 | 0 | 0 |
|  | F9 | 168 344 | 1 | 0 | 0 |
|  | F10 | 168 320 | 1 | 0 | 0 |
|  | F11 | 168 278 | 1 | 0 | 0 |
|  | F12 | 355 | 1 | 0 | 0 |
|  | F13 | 93 | 2 | 0 | 0 |
|  | F14 | 129 209 | 1 | 0 | 0 |
|  | F15 | 129 | 21 | 0.2857 | 0.1782 |
|  | F16 | 168 | 9 | 0.2222 | 0.1571 |
|  | F17 | root | 1 | 0 | 0 |
| L0b | F18 | root | 1 | 0 | 0 |
| L0d | F19 | 290 300 399 | 1 | 0 | 0 |
| L0f | F20 | 52 290 354 325 | 1 | 0 | 0 |
| L0k | F21 | 223 291A | 2 | 0 | 0 |
| L1b | F22 | 239 | 2 | 0.5 | 0.5 |
|  | F23 | 289 | 1 | 0 | 0 |
|  | F24 | 293 | 1 | 0 | 0 |
|  | F25 | root | 5 | 0.2 | 0.2 |
| L1c2 | F26 | 71 145 213 234 | 2 | 0 | 0 |
|  | F27 | 78 187 278 320 | 1 | 0 | 0 |
|  | F28 | 78 278 320 | 1 | 0 | 0 |
| L1c | F29 | 172 173 187 188A 256 274 368 | 2 | 0 | 0 |
| L1c3a | F30 | 93 360 | 1 | 0 | 0 |
|  | F31 | root | 2 | 1 | 0.7071 |
| L2a | F32 | 92 189 286 | 2 | 0 | 0 |
|  | F33 | 189 286 | 8 | 0.125 | 0.125 |
|  | F34 | 189 234 249 295 309 | 1 | 0 | 0 |
|  | F35 | 192 209 | 1 | 0 | 0 |
|  | F36 | 192 292 309 | 1 | 0 | 0 |
|  | F37 | 93 192 309 | 2 | 0 | 0 |
|  | F38 | 189 209 301 309 354 | 3 | 0 | 0 |
|  | F39 | 189 239 | 2 | 1 | 0.7071 |
|  | F40 | 145 309 | 2 | 1 | 0.7071 |
|  | F41 | 192 292 | 8 | 0 | 0 |
|  | F42 | 189 192 | 5 | 0 | 0 |
|  | F43 | 192 309 | 5 | 0.4 | 0.2828 |
|  | F44 | 93 192 | 2 | 0 | 0 |
|  | F45 | 189 309 | 3 | 0.6667 | 0.4714 |
|  | F46 | 129 189 | 4 | 0 | 0 |
|  | F47 | 187 | 1 | 0 | 0 |
|  | F48 | 192 | 7 | 0.8571 | 0.5345 |
|  | F49 | 93 | 1 | 0 | 0 |
|  | F50 | 290 | 2 | 0 | 0 |
|  | F51 | 193 | 1 | 0 | 0 |
|  | F52 | 344 | 1 | 0 | 0 |
|  | F53 | 189 | 5 | 0.4 | 0.2828 |
|  | F54 | root | 4 | 0.5 | 0.3536 |
| L2b | F55 | 355 362 | 2 | 0 | 0 |
|  | F56 | 145 | 2 | 0 | 0 |
|  | F57 | 362 | 1 | 0 | 0 |
| L2d | F58 | 399 | 2 | 0 | 0 |
| L2e | F59 | root | 1 | 0 | 0 |
| L2* | F60 | 264 266 | 2 | 0.5 | 0.5 |
|  | F61 | 264 | 1 | 0 | 0 |
| L3b | F62 | 124 | 4 | 0.5 | 0.3536 |
|  | F63 | 86 | 1 | 0 | 0 |
|  | F64 | 311 | 1 | 0 | 0 |
|  | F65 | 93 | 10 | 0 | 0 |
|  | F66 | root | 11 | 0 | 0 |
| L3d | F67 | 189 278 304 311 | 3 | 0 | 0 |
|  | F68 | 256 368 | 1 | 0 | 0 |
|  | F69 | 300 319 | 1 | 0 | 0 |
|  | F70 | 256 | 1 | 0 | 0 |
|  | F71 | 319 | 18 | 0.0556 | 0.0556 |
|  | F72 | root | 2 | 0.5 | 0.5 |
| L3e | F73 | 185 209 311 327 | 1 | 0 | 0 |
|  | F74 | 172 189 320 | 2 | 0 | 0 |
|  | F75 | 185 209 327 | 3 | 0 | 0 |
|  | F76 | 51 264 | 1 | 0 | 0 |
|  | F77 | 256 327 | 2 | 1 | 0.7071 |
|  | F78 | 265T | 16 | 0.1875 | 0.1398 |
|  | F79 | 327 | 8 | 0.25 | 0.1768 |
| L3f | F80 | 292 295 | 1 | 0 | 0 |
|  | F81 | 189 292 | 1 | 0 | 0 |
|  | F82 | 93 292 | 3 | 0 | 0 |
|  | F83 | 327 | 1 | 0 | 0 |
|  | F84 | 224 | 1 | 0 | 0 |
|  | F85 | 292 | 11 | 0.8182 | 0.4724 |
|  | F86 | 111A | 1 | 0 | 0 |
|  | F87 | root | 2 | 0.5 | 0.5 |
| L3x | F88 | 223 278 298 311 | 1 | 0 | 0 |
|  | F89 | 172 223 278 | 1 | 0 | 0 |
|  | F90 | 223 278 | 18 | 0 | 0 |
|  | F91 | 86 193 195 | 3 | 0.6667 | 0.6667 |
|  | F92 | 193 195 | 1 | 0 | 0 |
| L3* | F93 | 148 192 399 | 4 | 0 | 0 |
|  | F94 | 111 184 304 311 | 3 | 1 | 0.5774 |
|  | F95 | 311 | 1 | 0 | 0 |
|  | F96 | 362 | 3 | 0 | 0 |
|  | F97 | root | 2 | 0 | 0 |
| L4 | F98 | 207T 220 260 261 | 2 | 0 | 0 |
|  | F99 | 189 260 264 | 2 | 0.5 | 0.5 |
|  | F100 | 93 207T 260 | 1 | 0 | 0 |
|  | F101 | 207T 260 261 | 2 | 0 | 0 |
|  | F102 | 179 189 239 320 | 1 | 0 | 0 |
|  | F103 | 86 293T 355 399 | 1 | 0 | 0 |
|  | F104 | 287G 293T 355 399 | 1 | 0 | 0 |
|  | F105 | 172 293T 355 399 | 1 | 0 | 0 |
|  | F106 | 274 293T 355 399 | 1 | 0 | 0 |
|  | F107 | 207T 260 | 1 | 0 | 0 |
|  | F108 | root | 1 | 0 | 0 |
| L5 | F109 | 111 254 311 360 | 1 | 0 | 0 |
|  | F110 | 355 362 | 5 | 0.2 | 0.2 |
| L6 | F111 | 173 362 | 12 | 0 | 0 |
|  | F112 | 362 | 1 | 0 | 0 |
|  | F113 | root | 1 | 0 | 0 |
