# Supplementary material for: Genetic Stratigraphy of Key Demographic Events in Arabia
Source: PLoS One. 2015 Mar 4;10(3):e0118625. doi: 10.1371/journal.pone.0118625 (PMC4349752; doi:10.1371/journal.pone.0118625)
Supplement: S11 Table — (DOCX) [file pone.0118625.s049.docx]

**S11_Table** Founder lineages identified when using *f1* criterion from Arabian Peninsula, Near East and Iran to North Africa and to eastern Africa separately.

| ***f1*** |  |  | **From Arabian Peninsula and Near East to North Africa** | | | **From Arabian Peninsula and Near East to eastern Africa** | | |
| --- | --- | --- | --- | --- | --- | --- | --- | --- |
| **Clade** | **Founder** | **HVS-I variants (-16,000)** | ***n*** | **ρ** | **se** | ***n*** | **ρ** | **se** |
| HV1a3 | F1 | 67 183 327A | 1 | 0.0000 | 0.0000 |  |  |  |
| HV1d | F2 | 67 278 362 |  |  |  | 4 | 0.2500 | 0.2500 |
| H6 | F3 | 67 129 368 | 1 | 0.0000 | 0.0000 |  |  |  |
| H6b | F4 | 104 300 362 | 1 | 0.0000 | 0.0000 |  |  |  |
| H | F5 | 86 129 189 | 1 | 0.0000 | 0.0000 |  |  |  |
| H1b | F6 | 189 356 | 3 | 0.6667 | 0.4714 |  |  |  |
| H | F7 | 189 293 | 2 | 0.0000 | 0.0000 |  |  |  |
| H8 | F8 | 288 362 | 3 | 0.0000 | 0.0000 |  |  |  |
| H1b1b | F9 | 355 362 | 2 | 0.0000 | 0.0000 |  |  |  |
| H8c2 | F10 | 153 362 | 1 | 0.0000 | 0.0000 |  |  |  |
| HV1 | F11 | 67 354 | 1 | 0.0000 | 0.0000 |  |  |  |
| HV1a3 | F12 | 67 327A | 8 | 0.0000 | 0.0000 | 1 | 0.0000 | 0.0000 |
| HV1a1 | F13 | 67 355 | 1 | 0.0000 | 0.0000 |  |  |  |
| H5 | F14 | 189 304 | 2 | 0.0000 | 0.0000 |  |  |  |
| H6b | F15 | 300 362 | 4 | 0.5000 | 0.5000 |  |  |  |
| H13b1 | F16 | 261 262 | 6 | 0.0000 | 0.0000 |  |  |  |
| HV | F17 | 159 | 1 | 0.0000 | 0.0000 |  |  |  |
| HV | F18 | 245 | 1 | 0.0000 | 0.0000 |  |  |  |
| H | F19 | 266 | 2 | 0.0000 | 0.0000 |  |  |  |
| H39 | F20 | 299 | 1 | 0.0000 | 0.0000 |  |  |  |
| H4 | F21 | 287 | 3 | 1.3333 | 0.6667 |  |  |  |
| H | F22 | 92 | 1 | 0.0000 | 0.0000 |  |  |  |
| H | F23 | 293 | 2 | 1.0000 | 0.7071 |  |  |  |
| HV2 | F24 | 217 | 2 | 0.0000 | 0.0000 |  |  |  |
| HV | F25 | 288 | 2 | 0.0000 | 0.0000 |  |  |  |
| H7c | F26 | 265 | 2 | 1.0000 | 0.7071 |  |  |  |
| HV | F27 | 114 | 1 | 0.0000 | 0.0000 |  |  |  |
| HV | F28 | 145 | 6 | 0.0000 | 0.0000 |  |  |  |
| H15a1b | F29 | 248 | 1 | 0.0000 | 0.0000 |  |  |  |
| H15a1b | F30 | 184 | 3 | 0.0000 | 0.0000 |  |  |  |
| HV | F31 | 357 | 13 | 0.9231 | 0.8496 |  |  |  |
| HV | F32 | 193 | 2 | 0.0000 | 0.0000 |  |  |  |
| HV | F33 | 243 | 3 | 1.3333 | 0.6667 |  |  |  |
| HV | F34 | 172 | 6 | 1.0000 | 0.5774 |  |  |  |
| HV | F35 | 355 | 5 | 0.4000 | 0.2828 | 1 | 0.0000 | 0.0000 |
| HV | F36 | 260 | 4 | 0.7500 | 0.7500 |  |  |  |
| H3p | F37 | 222 | 2 | 0.5000 | 0.5000 |  |  |  |
| H13a1a6 | F38 | 207 | 7 | 0.4286 | 0.3194 |  |  |  |
| HV | F39 | 147 | 5 | 0.4000 | 0.2828 |  |  |  |
| HV | F40 | 298 | 145 | 0.6759 | 0.1936 | 2 | 1.5000 | 0.8660 |
| H2a1 | F41 | 354 | 5 | 0.4000 | 0.2828 |  |  |  |
| HV | F42 | 290 | 1 | 0.0000 | 0.0000 |  |  |  |
| HV | F43 | 187 | 7 | 0.1429 | 0.1429 |  |  |  |
| HV | F44 | 153 | 8 | 0.7500 | 0.7500 |  |  |  |
| HV | F45 | 111 | 10 | 0.0000 | 0.0000 |  |  |  |
| H | F46 | 218 | 11 | 0.7273 | 0.4066 | 1 | 0.0000 | 0.0000 |
| HV1 | F47 | 67 | 22 | 1.0000 | 0.3278 | 12 | 1.4167 | 0.7407 |
| HV | F48 | 221 | 4 | 1.7500 | 0.8292 |  |  |  |
| H5 | F49 | 304 | 28 | 0.7500 | 0.2448 |  |  |  |
| H6 | F50 | 362 | 7 | 0.5714 | 0.2857 |  |  |  |
| H | F51 | 261 | 5 | 1.0000 | 0.5292 |  |  |  |
| H1 | F52 | 278 | 2 | 0.5000 | 0.5000 |  |  |  |
| H2a3 | F53 | 274 | 7 | 0.1429 | 0.1429 |  |  |  |
| H | F54 | 256 | 14 | 1.2143 | 0.4684 |  |  |  |
| HV | F55 | 240 | 4 | 0.0000 | 0.0000 |  |  |  |
| HV | F56 | 192 | 4 | 0.7500 | 0.4330 |  |  |  |
| H | F57 | 189 | 27 | 0.2963 | 0.1960 | 1 | 0.0000 | 0.0000 |
| H | F58 | 93 | 9 | 0.2222 | 0.2222 |  |  |  |
| HV | F59 | 86 | 2 | 0.0000 | 0.0000 |  |  |  |
| H | F60 | 239 | 3 | 0.0000 | 0.0000 |  |  |  |
| HV | F61 | root | 465 | 0.4000 | 0.0763 | 8 | 0.5000 | 0.3953 |
| M1a3 | F62 | 223 311 | 3 | 0.6667 | 0.4714 |  |  |  |
| M1b2 | F63 | 399 | 3 | 0.6667 | 0.6667 |  |  |  |
| M1a5 | F64 | 129 | 3 | 1.3333 | 0.9428 | 24 | 0.8750 | 0.4390 |
| M1b1 | F65 | 185 | 10 | 0.4000 | 0.2449 |  |  |  |
| M1a3 | F66 | 223 | 8 | 0.2500 | 0.1768 |  |  |  |
| M1a1 | F67 | 359 | 45 | 0.7111 | 0.4309 | 36 | 0.7222 | 0.2664 |
| M1a1c'd | F68 | 93 359 |  |  |  | 10 | 0.6000 | 0.3162 |
| M1 | F69 | 357 |  |  |  | 4 | 0.2500 | 0.2500 |
| M1 | F70 | 240 |  |  |  | 3 | 0.0000 | 0.0000 |
| M1 | F71 | root | 46 | 0.1304 | 0.0532 | 13 | 0.7692 | 0.3264 |
| N1a | F72 | 147G 172 248 263 266 355 | 1 | 0.0000 | 0.0000 |  |  |  |
| N1b1 | F73 | 126 145 176G 390 | 1 | 0.0000 | 0.0000 |  |  |  |
| N1b1 | F74 | 145 176G 362 390 | 1 | 0.0000 | 0.0000 |  |  |  |
| N1b1 | F75 | 145 176G 311 390 | 9 | 1.0000 | 0.5092 |  |  |  |
| N1a1 | F76 | 147A 172 248 320 355 | 1 | 0.0000 | 0.0000 |  |  |  |
| N1a1 | F77 | 147A 172 248 355 | 3 | 1.0000 | 1.0000 | 16 | 0.1875 | 0.1398 |
| N1a | F78 | 147G 172 213 248 355 |  |  |  | 7 | 0.0000 | 0.0000 |
| N1a3 | F79 | 201 265 | 1 | 0.0000 | 0.0000 |  |  |  |
| N1b1 | F80 | 145 176G 390 | 15 | 0.6000 | 0.2000 |  |  |  |
| N1b | F81 | 176G 390 | 1 | 0.0000 | 0.0000 | 5 | 0.0000 | 0.0000 |
| I5a | F82 | 148 129 294 391 | 1 | 0.0000 | 0.0000 |  |  |  |
| I5a | F83 | 129 148 391 | 1 | 0.0000 | 0.0000 |  |  |  |
| I1c | F84 | 129 311 319 391 | 1 | 0.0000 | 0.0000 |  |  |  |
| I1a | F85 | 129 172 311 391 | 2 | 0.0000 | 0.0000 |  |  |  |
| I | F86 | 93 129 391 | 3 | 0.3333 | 0.3333 |  |  |  |
| I1 | F87 | 129 311 391 | 7 | 0.8571 | 0.4949 |  |  |  |
| I | F88 | 129 391 | 6 | 0.3333 | 0.2357 | 3 | 0.0000 | 0.0000 |
| W | F89 | 292! | 1 | 0.0000 | 0.0000 |  |  |  |
| W | F90 | 292 295 | 1 | 0.0000 | 0.0000 |  |  |  |
| W | F91 | 292 | 12 | 1.5833 | 0.4640 | 4 | 2.7500 | 0.9682 |
| N2a | F92 | 153 319 |  |  |  | 4 | 2.2500 | 1.1456 |
| R0a | F93 | 189 232A | 2 | 0.0000 | 0.0000 |  |  |  |
| R0a1a | F94 | 185 355 | 2 | 0.0000 | 0.0000 |  |  |  |
| R0a | F95 | 145 | 3 | 0.0000 | 0.0000 | 1 | 0.0000 | 0.0000 |
| R0a | F96 | 189 | 1 | 0.0000 | 0.0000 | 2 | 0.0000 | 0.0000 |
| R0a2i | F97 | 92 | 2 | 0.0000 | 0.0000 |  |  |  |
| R0a1a | F98 | 355 | 13 | 0.9231 | 0.4615 | 9 | 0.2222 | 0.2222 |
| R0a | F99 | 266 | 1 | 0.0000 | 0.0000 | 3 | 1.3333 | 1.0541 |
| R0a2c | F100 | 304 | 4 | 0.0000 | 0.0000 | 1 | 0.0000 | 0.0000 |
| R0a | F101 | root | 26 | 0.3462 | 0.1490 | 12 | 0.5000 | 0.3118 |
| R0a | F102 | 93 |  |  |  | 1 | 0.0000 | 0.0000 |
| R0a | F103 | 114 |  |  |  | 3 | 0.0000 | 0.0000 |
| R0a2b | F104 | 305T |  |  |  | 12 | 0.0000 | 0.0000 |
| R0a1a1a | F105 | 172 184A |  |  |  | 1 | 0.0000 | 0.0000 |
| T1a | F106 | 163 172 186 189 298 | 3 | 1.3333 | 0.9428 |  |  |  |
| T2 | F107 | 146 292 296! | 1 | 0.0000 | 0.0000 |  |  |  |
| T2 | F108 | 146 147 292 296 | 5 | 0.4000 | 0.4000 |  |  |  |
| T2 | F109 | 288 292 296 |  |  |  | 2 | 0.0000 | 0.0000 |
| T1a | F110 | 163 186 189 287 | 1 | 0.0000 | 0.0000 |  |  |  |
| T1a | F111 | 163 186 189 271 | 1 | 0.0000 | 0.0000 |  |  |  |
| T1a | F112 | 163 186 189 390 | 1 | 0.0000 | 0.0000 |  |  |  |
| T1b | F113 | 163 189 243 |  |  |  | 1 | 0.0000 | 0.0000 |
| T2b | F114 | 209 296 304 | 1 | 0.0000 | 0.0000 |  |  |  |
| T2b | F115 | 292 296 304 | 1 | 0.0000 | 0.0000 | 1 | 0.0000 | 0.0000 |
| T2b | F116 | 296! 304 | 1 | 0.0000 | 0.0000 |  |  |  |
| T2c1c | F117 | 146 292 296 | 4 | 0.7500 | 0.4330 |  |  |  |
| T2c1 | F118 | 292 296! | 9 | 0.5556 | 0.2940 | 3 | 0.0000 | 0.0000 |
| T2c1c | F119 | 146 296! | 2 | 0.0000 | 0.0000 |  |  |  |
| T1a | F120 | 163 186 189 | 69 | 1.3043 | 0.3672 | 5 | 1.2000 | 0.6325 |
| T2e | F121 | 153 296 |  |  |  | 1 | 0.0000 | 0.0000 |
| T2k | F122 | 291 296 | 1 | 0.0000 | 0.0000 |  |  |  |
| T2 | F123 | 256 296 | 5 | 0.4000 | 0.2828 |  |  |  |
| T2f | F124 | 189 296 | 1 | 0.0000 | 0.0000 |  |  |  |
| T2b | F125 | 296 304 | 30 | 0.5667 | 0.1795 |  |  |  |
| T2a1b | F126 | 296 324 | 5 | 0.4000 | 0.2828 |  |  |  |
| T2c1 | F127 | 292 296 | 12 | 0.8333 | 0.4410 | 1 | 0.0000 | 0.0000 |
| T2 | F128 | 296! | 5 | 0.8000 | 0.4000 |  |  |  |
| T1 | F129 | 163 189 | 3 | 2.0000 | 1.1547 |  |  |  |
| T2 | F130 | 296 | 17 | 1.0000 | 0.4441 | 1 | 0.0000 | 0.0000 |
| J | F131 | 51 188 311 | 4 | 0.2500 | 0.2500 | 1 | 0.0000 | 0.0000 |
| J2a1a1 | F132 | 145 231 261 | 7 | 0.0000 | 0.0000 |  |  |  |
| J1d1 | F133 | 129 193 300 | 1 | 0.0000 | 0.0000 |  |  |  |
| J1d1a | F134 | 193 300 309 | 14 | 0.3571 | 0.1890 | 4 | 0.2500 | 0.2500 |
| J1b | F135 | 145 222 261! | 1 | 0.0000 | 0.0000 |  |  |  |
| J1b | F136 | 145 222 261 311 | 3 | 0.0000 | 0.0000 |  |  |  |
| J1b2a | F137 | 136 145 222 261 | 2 | 0.0000 | 0.0000 |  |  |  |
| J1b1a1 | F138 | 145 172 222 261 | 1 | 0.0000 | 0.0000 |  |  |  |
| J | F139 | 231 319 | 1 | 0.0000 | 0.0000 |  |  |  |
| J | F140 | 69 145 | 2 | 0.0000 | 0.0000 |  |  |  |
| J2 | F141 | 241 311 | 1 | 0.0000 | 0.0000 |  |  |  |
| J1d1 | F142 | 193 300 | 2 | 0.0000 | 0.0000 |  |  |  |
| J1b | F143 | 145 261 355 | 1 | 0.0000 | 0.0000 |  |  |  |
| J1b | F144 | 129 145 261 | 1 | 0.0000 | 0.0000 |  |  |  |
| J1b | F145 | 145 222 261 | 7 | 0.7143 | 0.4286 | 1 | 0.0000 | 0.0000 |
| J1b1a1 | F146 | 145 172 261 | 1 | 0.0000 | 0.0000 |  |  |  |
| J | F147 | 271 | 1 | 0.0000 | 0.0000 |  |  |  |
| J | F148 | 256 | 2 | 0.0000 | 0.0000 |  |  |  |
| J | F149 | 231 | 2 | 1.5000 | 1.1180 | 1 | 0.0000 | 0.0000 |
| J | F150 | 69 | 3 | 1.0000 | 0.5774 |  |  |  |
| J | F151 | 362 | 3 | 0.3333 | 0.3333 |  |  |  |
| J2a2b | F152 | 241 | 17 | 0.2353 | 0.1176 |  |  |  |
| J1d | F153 | 193 | 19 | 1.6842 | 0.5766 |  |  |  |
| J1c7 | F154 | 261 | 2 | 0.0000 | 0.0000 |  |  |  |
| J1b | F155 | 145 261 | 3 | 0.0000 | 0.0000 | 1 | 0.0000 | 0.0000 |
| J | F156 | root | 54 | 0.5741 | 0.1493 | 2 | 0.5000 | 0.5000 |
| U5b1b1b | F157 | 189 192 320 | 2 | 1.0000 | 0.7071 |  |  |  |
| U5a1b1 | F158 | 291 256 399 | 4 | 0.7500 | 0.7500 |  |  |  |
| U5a1 | F159 | 192 256 399 | 1 | 0.0000 | 0.0000 |  |  |  |
| U5b2a1 | F160 | 189 270 | 3 | 0.0000 | 0.0000 |  |  |  |
| U5 | F161 | 145 192 | 1 | 0.0000 | 0.0000 |  |  |  |
| U5b1b1 | F162 | 189 192 | 12 | 0.8333 | 0.2635 |  |  |  |
| U5a1 | F163 | 256 399 | 3 | 2.0000 | 0.8165 |  |  |  |
| U5a | F164 | 192 256 | 3 | 0.3333 | 0.3333 |  |  |  |
| U5b3 | F165 | 304 | 2 | 0.5000 | 0.5000 |  |  |  |
| U5b2a | F166 | 189 | 12 | 0.5833 | 0.2205 |  |  |  |
| U5 | F167 | 192 | 26 | 0.4615 | 0.2176 | 2 | 2.0000 | 1.0000 |
| U5a | F168 | 256 | 8 | 1.1250 | 0.5449 |  |  |  |
| U5 | F169 | root | 3 | 2.0000 | 0.8165 |  |  |  |
| U6a1a | F170 | 172 189 219 278 295 | 8 | 0.0000 | 0.0000 |  |  |  |
| U6a1a | F171 | 147 172 189 219 278 | 2 | 0.0000 | 0.0000 |  |  |  |
| U1a | F172 | 129 189 249 288 362 | 2 | 0.5000 | 0.5000 |  |  |  |
| U2e2 | F173 | 51 92 129C 189 362 | 1 | 0.0000 | 0.0000 |  |  |  |
| U6b'd | F174 | 172 219 311 362 | 1 | 0.0000 | 0.0000 |  |  |  |
| U6a1b | F175 | 219 172 235 278 | 8 | 1.5000 | 0.7071 |  |  |  |
| U6a2'3 | F176 | 172 189 219 278 | 49 | 0.8163 | 0.3487 | 15 | 0.6000 | 0.3197 |
| U2b2 | F177 | 51 209 239 352 353 | 1 | 0.0000 | 0.0000 |  |  |  |
| U2e | F178 | 51 129C 189 362 | 4 | 0.0000 | 0.0000 |  |  |  |
| U2d | F179 | 51 189 234 294 | 1 | 0.0000 | 0.0000 | 2 | 1.0000 | 0.7071 |
| U6b'd | F180 | 172 311 219 | 5 | 0.2000 | 0.2000 |  |  |  |
| U6a | F181 | 172 219 278 | 40 | 0.7500 | 0.2179 |  |  |  |
| U1a | F182 | 189 249 362 | 1 | 0.0000 | 0.0000 |  |  |  |
| U1a | F183 | 189 249 311 | 1 | 0.0000 | 0.0000 |  |  |  |
| U2b2 | F184 | 51 352 353 | 1 | 0.0000 | 0.0000 |  |  |  |
| U2 | F185 | 51 189 362 | 2 | 0.0000 | 0.0000 |  |  |  |
| U7 | F186 | 309 318T 362 | 1 | 0.0000 | 0.0000 |  |  |  |
| U3a | F187 | 260 343 390 | 2 | 0.0000 | 0.0000 |  |  |  |
| U3b3 | F188 | 168 343 355 | 2 | 0.0000 | 0.0000 |  |  |  |
| K1 | F189 | 93 189 224 311 | 1 | 0.0000 | 0.0000 |  |  |  |
| U6a'b'd | F190 | 172 219 | 7 | 0.8571 | 0.5345 |  |  |  |
| U1a'c | F191 | 189 249 | 6 | 1.5000 | 0.7993 |  |  |  |
| U1b | F192 | 111 214A 249 327 |  |  |  | 1 | 0.0000 | 0.0000 |
| U7 | F193 | 318C 318T | 1 | 0.0000 | 0.0000 |  |  |  |
| U7 | F194 | 309 318T | 3 | 1.6667 | 0.8819 |  |  |  |
| U4c2a | F195 | 261 356 | 3 | 0.3333 | 0.3333 |  |  |  |
| U4a2b | F196 | 223 356 | 1 | 0.0000 | 0.0000 |  |  |  |
| U3c | F197 | 193 343 | 2 | 2.5000 | 1.3229 |  |  |  |
| U3 | F198 | 104 343 | 2 | 0.0000 | 0.0000 |  |  |  |
| U3a | F199 | 343 390 | 8 | 0.8750 | 0.4146 |  |  |  |
| U3b3 | F200 | 168 343 | 1 | 0.0000 | 0.0000 | 1 | 0.0000 | 0.0000 |
| U8b1b | F201 | 189 234 324 | 1 | 0.0000 | 0.0000 |  |  |  |
| K2c | F202 | 210 224 311 | 2 | 0.5000 | 0.5000 |  |  |  |
| K | F203 | 167 224 311 | 1 | 0.0000 | 0.0000 |  |  |  |
| K | F204 | 158 224 311 | 1 | 0.0000 | 0.0000 |  |  |  |
| K | F205 | 224 290 311 | 3 | 0.0000 | 0.0000 |  |  |  |
| K | F206 | 224 242 311 | 2 | 1.5000 | 0.8660 |  |  |  |
| K | F207 | 224 278 311 | 1 | 0.0000 | 0.0000 | 1 | 0.0000 | 0.0000 |
| K1b1a | F208 | 224 311 319 | 2 | 1.5000 | 0.8660 |  |  |  |
| K1 | F209 | 224 234 311 | 3 | 0.0000 | 0.0000 |  |  |  |
| K | F210 | 224 311 360 | 1 | 0.0000 | 0.0000 |  |  |  |
| K1 | F211 | 224 311 362 | 2 | 0.0000 | 0.0000 |  |  |  |
| K1a27 | F212 | 176 224 311 | 2 | 0.0000 | 0.0000 |  |  |  |
| K | F213 | 189 224 311 | 3 | 0.0000 | 0.0000 | 1 | 0.0000 | 0.0000 |
| K1 | F214 | 93 224 311 | 15 | 0.1333 | 0.0943 | 2 | 0.0000 | 0.0000 |
| K | F215 | 224 304 311 | 2 | 0.5000 | 0.5000 |  |  |  |
| U6 | F216 | 172 | 4 | 2.7500 | 1.3463 |  |  |  |
| U1 | F217 | 249 | 1 | 0.0000 | 0.0000 |  |  |  |
| U2 | F218 | 51 | 1 | 0.0000 | 0.0000 |  |  |  |
| U9a | F219 | 51 278 | 1 | 0.0000 | 0.0000 | 1 | 0.0000 | 0.0000 |
| U7 | F220 | 318T | 2 | 2.0000 | 1.0000 |  |  |  |
| U4 | F221 | 356 | 13 | 0.6154 | 0.3077 |  |  |  |
| U3 | F222 | 343 | 21 | 1.0476 | 0.2935 | 1 | 0.0000 | 0.0000 |
| K | F223 | 224 311 | 74 | 0.6216 | 0.1324 | 8 | 0.1250 | 0.1250 |
| X1 | F224 | 104 278 | 7 | 0.0000 | 0.0000 |  |  |  |
| X1 | F225 | 104 |  |  |  | 1 | 0.0000 | 0.0000 |
| X2j | F226 | 179 | 4 | 1.0000 | 1.0000 |  |  |  |
| X2h | F227 | 223 | 3 | 1.6667 | 1.0000 |  |  |  |
| X4 | F228 | 274 | 1 | 0.0000 | 0.0000 |  |  |  |
| X2 | F229 | 248 | 1 | 0.0000 | 0.0000 |  |  |  |
| X | F230 | root | 35 | 0.6571 | 0.1959 | 3 | 0.6667 | 0.4714 |
