# Supplementary material for: Genetic Stratigraphy of Key Demographic Events in Arabia
Source: PLoS One. 2015 Mar 4;10(3):e0118625. doi: 10.1371/journal.pone.0118625 (PMC4349752; doi:10.1371/journal.pone.0118625)
Supplement: S12 Table — (DOCX) [file pone.0118625.s050.docx]

**S12_Table** Founder lineages identified when using *f2* criterion from Arabian Peninsula, Near East and Iran to North Africa and to eastern Africa separately.

| ***f2*** |  |  | **From Arabian Peninsula and Near East to North Africa** | | | **From Arabian Peninsula and Near East to eastern Africa** | | |
| --- | --- | --- | --- | --- | --- | --- | --- | --- |
| **Clade** | **Founder** | **HVS-I variants (-16,000)** | ***n*** | **ρ** | **se** | ***n*** | **ρ** | **se** |
| H1b | F1 | 189 356 | 3 | 0.6667 | 0.4714 |  |  |  |
| HV1a1 | F2 | 67 355 | 1 | 0.0000 | 0.0000 |  |  |  |
| H6 | F3 | 300 362 | 5 | 0.8000 | 0.4899 |  |  |  |
| H | F4 | 266 | 2 | 0.0000 | 0.0000 |  |  |  |
| H4 | F5 | 287 | 3 | 1.3333 | 0.6667 |  |  |  |
| H | F6 | 92 | 1 | 0.0000 | 0.0000 |  |  |  |
| H | F7 | 293 | 4 | 1.2500 | 0.6614 |  |  |  |
| HV2 | F8 | 217 | 2 | 0.0000 | 0.0000 |  |  |  |
| H7c | F9 | 265 | 2 | 1.0000 | 0.7071 |  |  |  |
| HV | F10 | 114 | 1 | 0.0000 | 0.0000 |  |  |  |
| HV | F11 | 145 | 6 | 0.0000 | 0.0000 |  |  |  |
| H15a1b | F12 | 248 | 1 | 0.0000 | 0.0000 |  |  |  |
| HV | F13 | 243 | 3 | 1.3333 | 0.6667 |  |  |  |
| HV | F14 | 172 | 6 | 1.0000 | 0.5774 |  |  |  |
| HV | F15 | 355 | 7 | 0.5714 | 0.3499 | 1 | 0.0000 | 0.0000 |
| HV | F16 | 260 | 4 | 0.7500 | 0.7500 |  |  |  |
| H3p | F17 | 222 | 2 | 0.5000 | 0.5000 |  |  |  |
| HV | F18 | 298 | 145 | 0.6759 | 0.1936 | 2 | 1.5000 | 0.8660 |
| H2a1 | F19 | 354 | 5 | 0.4000 | 0.2828 |  |  |  |
| HV | F20 | 290 | 1 | 0.0000 | 0.0000 |  |  |  |
| HV | F21 | 187 | 7 | 0.1429 | 0.1429 |  |  |  |
| HV | F22 | 153 | 9 | 0.8889 | 0.6849 |  |  |  |
| H | F23 | 218 | 11 | 0.7273 | 0.4066 | 1 | 0.0000 | 0.0000 |
| HV1 | F24 | 67 | 33 | 1.3636 | 0.4318 | 17 | 1.7059 | 0.6308 |
| H5 | F25 | 304 | 30 | 0.7667 | 0.2380 |  |  |  |
| H6 | F26 | 362 | 7 | 0.5714 | 0.2857 |  |  |  |
| H | F27 | 261 | 11 | 2.0909 | 0.9749 |  |  |  |
| H2a3 | F28 | 274 | 7 | 0.1429 | 0.1429 |  |  |  |
| H | F29 | 256 | 14 | 1.2143 | 0.4684 |  |  |  |
| HV | F30 | 240 | 4 | 0.0000 | 0.0000 |  |  |  |
| HV | F31 | 192 | 4 | 0.7500 | 0.4330 |  |  |  |
| H | F32 | 189 | 27 | 0.2963 | 0.1960 | 1 | 0.0000 | 0.0000 |
| H | F33 | 93 | 9 | 0.2222 | 0.2222 |  |  |  |
| HV | F34 | 86 | 3 | 1.0000 | 0.5774 |  |  |  |
| H | F35 | 239 | 3 | 0.0000 | 0.0000 |  |  |  |
| HV | F36 | root | 519 | 0.5318 | 0.0823 | 8 | 0.5000 | 0.3953 |
| M1a3 | F37 | 223 311 | 3 | 0.6667 | 0.4714 |  |  |  |
| M1a3 | F38 | 223 | 8 | 0.2500 | 0.1768 |  |  |  |
| M1a1 | F39 | 359 | 45 | 0.7111 | 0.4309 | 46 | 0.9130 | 0.3090 |
| M1 | F40 | root | 62 | 0.5161 | 0.1922 | 44 | 1.4318 | 0.6145 |
| N1b1 | F41 | 126 145 176G 390 | 1 | 0.0000 | 0.0000 |  |  |  |
| N1a1 | F42 | 147A 172 248 320 355 | 1 | 0.0000 | 0.0000 |  |  |  |
| N1a1 | F43 | 147A 172 248 355 | 4 | 1.2500 | 0.8292 | 23 | 0.4348 | 0.3195 |
| N1a3 | F44 | 201 265 | 1 | 0.0000 | 0.0000 |  |  |  |
| N1b1 | F45 | 145 176G 390 | 25 | 1.1200 | 0.4233 |  |  |  |
| I5a | F46 | 129 148 391 | 2 | 1.0000 | 0.7071 |  |  |  |
| I1 | F47 | 129 311 391 | 7 | 0.8571 | 0.4949 |  |  |  |
| I | F48 | 129 391 | 12 | 1.0833 | 0.3997 | 3 | 0.0000 | 0.0000 |
| N1 | F49 | root | 1 | 0.0000 | 0.0000 | 5 | 0.0000 | 0.0000 |
| W | F50 | 292 | 14 | 1.7857 | 0.4345 | 4 | 2.7500 | 0.9682 |
| N2a | F51 | 153 319 |  |  |  | 4 | 2.2500 | 1.1456 |
| R0a1a | F52 | 185 355 | 2 | 0.0000 | 0.0000 |  |  |  |
| R0a | F53 | 189 | 3 | 0.6667 | 0.6667 | 2 | 0.0000 | 0.0000 |
| R0a1a | F54 | 355 | 13 | 0.9231 | 0.4615 | 9 | 0.2222 | 0.2222 |
| R0a | F55 | 114 |  |  |  | 3 | 0.0000 | 0.0000 |
| R0a2c | F56 | 304 | 4 | 0.0000 | 0.0000 | 1 | 0.0000 | 0.0000 |
| R0a | F57 | root | 32 | 0.5625 | 0.1926 | 30 | 1.0000 | 0.4497 |
| T2 | F58 | 146 292 296 296 | 1 | 0.0000 | 0.0000 |  |  |  |
| T2b | F59 | 296! 304 | 1 | 0.0000 | 0.0000 |  |  |  |
| T2 | F60 | 288 292 296 |  |  |  | 2 | 0.0000 | 0.0000 |
| T2c1c | F61 | 146 292 296 | 9 | 1.1111 | 0.6285 |  |  |  |
| T2c1 | F62 | 292 296 296 | 9 | 0.5556 | 0.2940 | 3 | 0.0000 | 0.0000 |
| T1a | F63 | 163 186 189 | 75 | 1.4533 | 0.3485 | 5 | 1.2000 | 0.6325 |
| T2k | F64 | 291 296 | 1 | 0.0000 | 0.0000 |  |  |  |
| T2b | F65 | 296 304 | 32 | 0.6563 | 0.1795 | 1 | 0.0000 | 0.0000 |
| T2c1 | F66 | 292 296 | 12 | 0.8333 | 0.4410 | 1 | 0.0000 | 0.0000 |
| T2 | F67 | 146 296 | 2 | 0.0000 | 0.0000 |  |  |  |
| T2 | F68 | 296 296 | 5 | 0.8000 | 0.4000 |  |  |  |
| T2e | F69 | 153 296 |  |  |  | 1 | 0.0000 | 0.0000 |
| T1 | F70 | 163 189 | 3 | 2.0000 | 1.1547 | 1 | 0.0000 | 0.0000 |
| T2 | F71 | 296 | 28 | 1.1429 | 0.3780 | 1 | 0.0000 | 0.0000 |
| J2a1a1 | F72 | 145 231 261 | 7 | 0.0000 | 0.0000 |  |  |  |
| J1d1a | F73 | 193 300 309 | 14 | 0.3571 | 0.1890 | 4 | 0.2500 | 0.2500 |
| J1b | F74 | 145 222 261 261 | 1 | 0.0000 | 0.0000 |  |  |  |
| J1b2a | F75 | 136 145 222 261 | 2 | 0.0000 | 0.0000 |  |  |  |
| J1d1 | F76 | 193 300 | 3 | 0.3333 | 0.3333 |  |  |  |
| J1b | F77 | 145 222 261 | 11 | 0.8182 | 0.3963 | 1 | 0.0000 | 0.0000 |
| J1b1a1 | F78 | 145 172 261 | 1 | 0.0000 | 0.0000 |  |  |  |
| J | F79 | 231 | 3 | 1.6667 | 0.8819 | 1 | 0.0000 | 0.0000 |
| J | F80 | 69 | 5 | 1.0000 | 0.5292 |  |  |  |
| J2a2b | F81 | 241 | 18 | 0.2778 | 0.1242 |  |  |  |
| J1d | F82 | 193 | 19 | 1.6842 | 0.5766 |  |  |  |
| J1b | F83 | 145 261 | 5 | 0.6000 | 0.3464 | 1 | 0.0000 | 0.0000 |
| J | F84 | root | 66 | 0.8485 | 0.1780 | 3 | 1.3333 | 0.6667 |
| U5a1 | F85 | 192 256 399 | 1 | 0.0000 | 0.0000 |  |  |  |
| U5b2a1 | F86 | 189 270 | 3 | 0.0000 | 0.0000 |  |  |  |
| U5a1 | F87 | 256 399 | 7 | 1.8571 | 0.7954 |  |  |  |
| U5a | F88 | 192 256 | 3 | 0.3333 | 0.3333 |  |  |  |
| U5b2a | F89 | 189 | 14 | 1.0000 | 0.3030 |  |  |  |
| U5 | F90 | 192 | 39 | 0.9231 | 0.3516 | 2 | 2.0000 | 1.0000 |
| U5a | F91 | 256 | 8 | 1.1250 | 0.5449 |  |  |  |
| U5 | F92 | root | 5 | 2.0000 | 0.6928 |  |  |  |
| U2e2 | F93 | 51 92 129C 189 362 | 1 | 0.0000 | 0.0000 |  |  |  |
| U2b2 | F94 | 51 209 239 352 353 | 1 | 0.0000 | 0.0000 |  |  |  |
| U2e | F95 | 51 129C 189 362 | 4 | 0.0000 | 0.0000 |  |  |  |
| U6a | F96 | 172 219 278 | 107 | 1.4860 | 0.5284 | 15 | 1.6000 | 1.0499 |
| U5b2c2b | F97 | 189 249 288 | 2 | 3.5000 | 1.8028 |  |  |  |
| U2b2 | F98 | 51 352 353 | 1 | 0.0000 | 0.0000 |  |  |  |
| U2 | F99 | 51 189 362 | 2 | 0.0000 | 0.0000 |  |  |  |
| U3b3 | F100 | 168 343 355 | 2 | 0.0000 | 0.0000 |  |  |  |
| U6a'b'd | F101 | 172 219 | 13 | 1.0769 | 0.5547 |  |  |  |
| U1a'c | F102 | 189 249 | 8 | 1.3750 | 0.6250 |  |  |  |
| U1b | F103 | 111 214A 249 327 |  |  |  | 1 | 0.0000 | 0.0000 |
| U2c'd | F104 | 51 234 | 1 | 0.0000 | 0.0000 | 2 | 3.0000 | 1.5811 |
| U7 | F105 | 318C 318T | 1 | 0.0000 | 0.0000 |  |  |  |
| U7 | F106 | 309 318T | 4 | 1.5000 | 0.7071 |  |  |  |
| U3c | F107 | 193 343 | 2 | 2.5000 | 1.3229 |  |  |  |
| U3a | F108 | 343 390 | 10 | 0.9000 | 0.3873 |  |  |  |
| U3b3 | F109 | 168 343 | 1 | 0.0000 | 0.0000 | 1 | 0.0000 | 0.0000 |
| U8b1b | F110 | 189 234 324 | 1 | 0.0000 | 0.0000 |  |  |  |
| K | F111 | 167 224 311 | 1 | 0.0000 | 0.0000 |  |  |  |
| K1 | F112 | 93 224 311 | 16 | 0.1875 | 0.1083 | 2 | 0.0000 | 0.0000 |
| U1 | F113 | 249 | 1 | 0.0000 | 0.0000 |  |  |  |
| U2 | F114 | 51 | 1 | 0.0000 | 0.0000 |  |  |  |
| U9a | F115 | 51 278 | 1 | 0.0000 | 0.0000 | 1 | 0.0000 | 0.0000 |
| U7 | F116 | 318T | 2 | 2.0000 | 1.0000 |  |  |  |
| U4 | F117 | 356 | 17 | 0.7647 | 0.3057 |  |  |  |
| U3 | F118 | 343 | 23 | 1.0435 | 0.2818 | 1 | 0.0000 | 0.0000 |
| K | F119 | 224 311 | 98 | 0.9388 | 0.1414 | 10 | 0.5000 | 0.2236 |
| U | F120 | root | 4 | 2.7500 | 1.3463 |  |  |  |
| X1 | F121 | 104 278 | 7 | 0.0000 | 0.0000 |  |  |  |
| X2 | F122 | 248 | 1 | 0.0000 | 0.0000 |  |  |  |
| X | F123 | root | 43 | 0.9302 | 0.2302 | 4 | 1.2500 | 0.5590 |
