# Supplementary material for: Genetic Stratigraphy of Key Demographic Events in Arabia
Source: PLoS One. 2015 Mar 4;10(3):e0118625. doi: 10.1371/journal.pone.0118625 (PMC4349752; doi:10.1371/journal.pone.0118625)
Supplement: S13 Table — (DOCX) [file pone.0118625.s051.docx]

S13_Table Samples used for genome-wide autosomal analysis.

| **Region** | **Populations** | ***n*** | **Reference** |
| --- | --- | --- | --- |
| Sub-Saharan Africa | Maasai, Kenya | 59 | HapMap |
|  | Ethiopia | 19 | 1 |
|  | Yoruba, Nigeria | 21 | 2 |
|  | Bantu, Kenya | 19 | 2 |
| North Africa | Egypt | 12 | 1 |
|  | Morocco | 25 | 3 |
|  | Tunisia | 12 | 3 |
| Arabian Peninsula | Yemen | 10 | 1 |
|  | Yemen Jews | 15 | 1 |
|  | Saudi Arabia | 20 | 1 |
|  | UAE | 14 | 3 |
| Near East | Bedouin, Israel | 45 | 2 |
|  | Lebanon | 7 | 1 |
|  | Syria | 16 | 1 |
|  | Jordan | 20 | 1 |
|  | Samaritan, Israel | 3 | 1 |
|  | Druze, Israel | 42 | 2 |
|  | Palestinian, Israel | 46 | 2 |
|  | Iran | 20 | 1 |
|  | Turkey | 19 | 1 |
| Caucasus | Armenia | 19 | 1 |
|  | Lezgin | 18 | 1 |
|  | Georgia | 20 | 1 |
| Europe | Belarus | 9 | 1 |
|  | Lithuania | 10 | 1 |
|  | France | 28 | 2 |
|  | Russia | 25 | 2 |
|  | Spain | 34 | 3 |
|  | Italy | 18 | 3 |
|  | Greece | 20 | 3 |
|  | Romania | 16 | 1 |
|  | Hungary | 20 | 1 |
| South Asia | Balochi, Pakistan | 24 | 2 |
|  | Makrani, Pakistan | 25 | 2 |
|  | Pathan, Pakistan | 22 | 2 |
|  | Burusho, Pakistan | 25 | 2 |
|  | India | 13 | 1 |
| **Total** |  | 790 |  |

**References:**1. Behar DM, et al. (2010) The genome-wide structure of the Jewish people. Nature 466, 238-242.

2. Li JZ, et al. (2008) Worldwide human relationships inferred from genome-wide patterns of variation. Science 319, 1100-1104.

3. Hellenthal G, et al. (2014) A genetic atlas of human admixture history. Science 343, 747-751.
