# Supplementary material for: Genetic Stratigraphy of Key Demographic Events in Arabia
Source: PLoS One. 2015 Mar 4;10(3):e0118625. doi: 10.1371/journal.pone.0118625 (PMC4349752; doi:10.1371/journal.pone.0118625)
Supplement: S14 Table — Increment ratio corresponds to the number of times the effective population size increase during this period. (DOCX) [file pone.0118625.s052.docx]

S14_Table Peaks of rate of population size change through time as obtained from the BSPs and periods of time where the rate of population size increase was of at least one individual per 100 individuals in a period of 100 years. Increment ratio corresponds to the number of times the effective population size increase during this period.

|  | **Peak** | **Range of increment** | **Increment ratio** |
| --- | --- | --- | --- |
| **L4** | 9.4 | 6.9;12.4 | 3.25 |
|  | 19.4 | 18.4; 22.6 | 1.6 |
|  | 71.5 | 68.7; 75.6 | 2.1 |
| **L4_L6** | 9.9 | 7.4;12.9 | 2.8 |
|  | 18.3 | 14.5; 21.1 | 2.4 |
|  | 73.8 | 70; 74.6 | 1.5 |
