# Supplementary material for: Genetic Stratigraphy of Key Demographic Events in Arabia
Source: PLoS One. 2015 Mar 4;10(3):e0118625. doi: 10.1371/journal.pone.0118625 (PMC4349752; doi:10.1371/journal.pone.0118625)
Supplement: S15 Table — This is a sub-set of S9 Table. (DOCX) [file pone.0118625.s053.docx]

**S15_Table** Ages for the oldest founders in the migration from Africa into Arabian Peninsula, Near East and Iran. This is a sub-set of S9_Table.

| **Founder** | **Founder** | ***n*** | **ρ** | **se** | **Age estimate** | **95% ci lower bound** | **95% ci upper bound** |
| --- | --- | --- | --- | --- | --- | --- | --- |
| L3f | 292 | 11 | 0.8182 | 0.4724 | 13643.0 | -1798 | 29085.0 |
| L0a | 168 278 293 | 7 | 0.571429 | 0.285714 | 9529.7 | 190.6 | 18868.8 |
| L1b | 239 | 2 | 0.5 | 0.5 | 8338.5 | -8004.9 | 24681.9 |
| L1c3a | root | 2 | 1 | 0.707107 | 16677.0 | -6436.1 | 39790.1 |
| L2a1 | 189 239 | 2 | 1 | 0.707107 | 16677.0 | -6436.1 | 39790.1 |
| L2a1 | 145 309 | 2 | 1 | 0.707107 | 16677.0 | -6436.1 | 39790.1 |
| L2a1 | 189 309 | 3 | 0.666667 | 0.471405 | 11118.0 | -4290.8 | 26526.8 |
| L2a1 | 192 | 7 | 0.857143 | 0.534522 | 14294.6 | -3177.3 | 31766.5 |
| L2a1 | root | 4 | 0.5 | 0.353553 | 8338.5 | -3218.1 | 19895.1 |
| L2 | 264 266 | 2 | 0.5 | 0.5 | 8338.5 | -8004.9 | 24681.9 |
| L3b | 124 | 4 | 0.5 | 0.353553 | 8338.5 | -3218.1 | 19895.1 |
| L3d | root | 2 | 0.5 | 0.5 | 8338.5 | -8004.9 | 24681.9 |
| L3e | 256 327 | 2 | 1 | 0.707107 | 16677.0 | -6436.1 | 39790.1 |
| L3f | root | 2 | 0.5 | 0.5 | 8338.5 | -8004.9 | 24681.9 |
| L3x | 86 193 195 | 3 | 0.666667 | 0.666667 | 11118.0 | -10673.3 | 32909.2 |
| L3* | 111 184 304 311 | 3 | 1 | 0.57735 | 16677.0 | -2194.8 | 35548.8 |
| L4 | 189 260 264 | 2 | 0.5 | 0.5 | 8338.5 | -8004.9 | 24681.9 |
