# Supplementary material for: Genetic Stratigraphy of Key Demographic Events in Arabia
Source: PLoS One. 2015 Mar 4;10(3):e0118625. doi: 10.1371/journal.pone.0118625 (PMC4349752; doi:10.1371/journal.pone.0118625)
Supplement: S1 Text — Includes 15 tables. (DOCX) [file pone.0118625.s054.docx]

**Genetic stratigraphy of key demographic events in Arabia**

Verónica Fernandes^1,2,3^, Petr Triska^1,2,4^, Joana B. Pereira^1,2,3^, Farida Alshamali^5^, Teresa Rito^2^, Alison Machado^2^, Zuzana Fajkošová^2,6^, Bruno Cavadas^1,2^, Viktor Černý^6^, Pedro Soares^2^, Martin B. Richards^3,7*^, Luísa Pereira^1,2,8*^

**Contents**

1 Results

1.1 Near Eastern, Iranian and Pakistani maternal genetic influence into the Arabian Peninsula

1.1.1 Insights from the whole-mtDNA sequencing of haplogroups JT

1.1.2 HVS-I founder analyses

1.2 Mitochondrial genetic exchanges across the Red Sea

1.2.1 Insights from the whole-mtDNA sequencing of haplogroups L4 and L6

1.2.2 HVS-I founder analysis from Africa to the Arabian Peninsula, Near East and Iran

1.2.3 HVS-I founder analyses from Arabian Peninsula, Near East and Iran to eastern Africa and North Africa

References

2 **Results**

*1.1 Near Eastern, Iranian and Pakistani maternal genetic influence into the Arabian Peninsula*

We have already published phylogenetic analyses based on whole-mtDNA sequencing for most of the N(xR) haplogroups found in Arabia, and here we completed this study for the second most frequent group of sequences in Arabia, haplogroup JT.

*1.1.1* *Insights from the whole-mtDNAsequencing of haplogroup JT*

In order to contextualise the sub-haplogroups within haplogroup JT that are present in Arabia, S1 and S2_Figs. present an outline topology of the total tree, indicating the primary branches with ages scaled against the ML estimates.

The distribution maps show that haplogroup J is frequent in Arabian Peninsula (S3A_Fig.), especially in the central/eastern part, where it peaks at 20%, the highest frequency recorded worldwide. While haplogroup T is broadly at low frequency (5–7%) across the Peninsula (S3B _Fig.), this is nevertheless not far from its frequency in the Near East (7–12%). The maps of the diversity measures for haplogroups J (S4_Fig.) and T (S5_Fig.) show that these lineages display the highest diversities in the Near East and Arabian Peninsula region, followed by the Caucasus region and the Indian Subcontinent. The similar frequency and diversity values between the Near Eastern and Peninsular Arabian JT pools suggest that these regions were probably in close contact around the time of emergence and dispersal of some JT sub-haplogroups, as is further supported by the phylogeographic analyses, described in detail below.

Another line of evidence comes from the BSP analyses for haplogroups JT in Arabia and the Near East (S6_Fig.), which indicate similar demographic expansion times. The population expansion in haplogroup J occurs first in the Near East, from ~15.0 ka until 11.0 ka, and soon afterwards in Arabia, mainly between ~12.0 and 8.0 ka. For haplogroup T, the low number of sequences observed in Arabia (*n*= 21; about half the number used for the other BSP inferences), all very diverse, renders the population expansion slightly older in Arabia (18.0–14.0 ka) than in the Near East (15.0–8.0 ka with a slight break in the increase ~12.0 ka). Most likely, sampling effect is leading us to over-estimate the age of haplogroup T in Arabia, as we did not find any evidence for it in the founder analysis, discussed below.

The Arabian haplogroup J samples cluster in specific subclades, both the frequent J1 and the rarer J2. Haplogroups J1 and J2 seem to have begun to diverge in the Near East 36.4 [24.4-49.0] ka and 38.5 [26.5-51.0] ka, respectively; and some of the lineages migrated into Arabia and participated in local expansions. J1 branches into three sub-haplogroups: J1b, J1c, and J1d. Haplogroup J1b is harboured in many Arabian samples, displaying some interesting patterns. Haplogroup J1b (S7_Fig.) is more frequent in Arabia and Iran, dating to 25.4 [19.3-31.6] ka. S8_Fig. also shows that many star-like J1b branches are observed in Arabia.

We see the globally more frequent haplogroup J1b1 (S9_Fig.) in only four Arabian samples, one in its oldest (22.6 [16.6–28.8] ka) and most frequent subclade, J1b1a (S10_Fig.), probably with a Near Eastern origin although, it is also very frequent across Europe; and three Arabian samples in J1b1b (S9_Fig.), which is clearly of Near Eastern origin and dates to 20.3 [13.6-27.2] ka. These sporadic Arabian J1b1 samples are likely to be recent introductions, as suggested by the close proximity between the UAE (DL90) and Indian (AY714033) sequences.

Curiously, the generally minor J1b subclades have a much more interesting distribution inside Arabia. This is the case for haplogroup J1b2 (S11_Fig.), defined by the transition at position 1733 (not identifiable when only characterising the HVS-I diversity) and dating to 10.4 [5.5-15.3] ka. By inspecting the whole-mtDNA sequences, we see that J1b2 seems to be located mainly in the Arabian Peninsula (one basal UAE sample supports its old presence in the region) and is also observed in the Near East, North Africa and southern Europe (most of these European sequences form a group restricted so far to this region and displaying no additional variation, pointing to a very recent arrival). The J1b2 subclades with clear dominance in the Arabian samples (J1b2a and one defined by polymorphisms at positions 152–16311) are dated at ~6.5 ka.

Haplogroup J1b3 (S8_Fig.), although rare, is very widespread, from the Arabian Peninsula to the Caucasus and southeastern and central Europe; the age estimation is 15.6 [8.7–22.9] ka. Haplogroup J1b6 (S8_Fig.) splits into J1b6a, which is mainly Arabian, and J1b6b, which is seen in Europeans sharing the fast-evolving polymorphism at position 195 with a Kuwait sample, suggesting that this subclade had an Arabian origin at 13.8 [3.6-24.6] ka. An unnamed J1b subclade, dated to 18.4 [11.3–25.7] ka and defined by the reverse transition at position 16222 and the transition at 152, both fast-evolving, unites a branch characterised by eight polymorphisms which has been so far only detected in UAE (S8_Fig.), dating at 6.5 [3.0-10.0] ka and the sub-haplogroup J1b8 (characterised by just two polymorphisms) seen in one Armenian and one Algerian sample.

Some of the clusters which are frequent in Arabia are also seen in North African individuals, providing a tentative to link with the Arab Islamisation of North Africa from the 7^th^ century AD.

No Arabian samples cluster in the J1c branch, which is mainly European and also typical of Ashkenazi Jews. It probably originated in Europe in the Late Glacial period (~17.1 [13.5-20.7] ka), and the few lineages (J1c12) localised to the Near East and the southern Caucasus were probably introduced by recent migrations from Europe [[1](#_ENREF_1)]. These lineages seem not to have reached the Arabian Peninsula.

Haplogroup J1d (S12_Fig.) is quite frequent in the Arabian Peninsula, and also present in southeast Europe, the Mediterranean basin, the Caucasus, the Near East, North Africa and eastern Africa. Given its wide geographic distribution, it could have been originated in the Near East, but given the presence of an UAE sequence which is basal in the haplogroup, and one UAE and an Armenian basal sequences in the J1d1a subclade, an origin in Arabian Peninsula cannot be ruled out. We dated J1d to 16.6 [12.9–20.4] ka, J1d1 to 13.7 [8.9–18.7] ka and J1d1a to 9.9 [6.2–13.7] ka. The whole-mtDNA genome data seem to suggest that the Arabian Peninsula was the centre for the migration of J1d1a1 lineages to East Africa, reaching as far south as Tanzania (S13_Fig.), and dating to ~7.1 [4.4-9.9] ka (S14_Fig.). The possible haplogroup J1d2 (defined by the fast evolving polymorphism at position 16519 [[1](#_ENREF_1)] is more frequent to the north (the probable centre of radiation was the Fertile Crescent) and east, in the Caucasus and Asia (S15_Fig.), and only J1d2c2, dated at 3 [0.2–5.8] ka, is mostly Arabian sharing an ancestor at 11.3 [6.4–16.4] ka with the recent (0.9 [0–2.2] ka) sub-group J1d2c1 restricted to the Caucasus.

Haplogroup J2 dates to 38.5 [26.5–51.0] ka and is widespread in Europe, especially in central and western regions, and parts of the Near East and North Africa, as well as the Arabian Peninsula (S16_Fig.). It divides into two basal branches, J2a and J2b, dating to 31.9 [21.8–42.4] ka and 21.6 [11.9–31.6] ka, respectively [[1](#_ENREF_1),[2](#_ENREF_2)]. J2a splits into two subclades, J2a1 and J2a2, which have very different geographic distributions. Haplogroup J2a1 is clearly European with a coalescence time around 27.5 [16.8–38.8] ka, and no Arabian whole-mtDNA sequences have been ascribed to this group. Haplogroup J2a2 (S17 and S18 Figs.) coalesces about 18.6 [13.6-23.6] ka, and the geographic distribution, based on the whole-mtDNA sequence tree (not identifiable in the HVS-I diversity), includes the Near East, Arabian Peninsula, North Africa and the Mediterranean basin, suggesting a re-expansion from the Near Eastern refugium shortly after the LGM along the Mediterranean and south into Arabia. There are two basal J2a2 lineages, located one in Yemen and one in Italy, and four subclades: J2a2a, J2a2b, J2a2c and J2a2d. Haplogroup J2a2a (S18 Fig.) dates to 15.4 [10.4–20.6] ka and includes a basal European sequence, a rare European cluster (J2a2a2), and a more frequent cluster, J2a2a1, defined by a substitution at the fast-evolving position 16311, which includes a basal European sequence. Haplogroup J2a2a1a is mainly seen in the Arabian Peninsula, Near East and North Africa, dating to 9.7 [3.3–16.4] ka. Haplogroup J2a2b (dating to 16.4 [11.5–21.4] ka), is frequent in the western part of Arabia and in North Africa (S19 Fig.). The phylogeny shows that the Arabian samples are basal and clustered with North African ones in J2a2b1 (coalescent time of 12.9 [6.6–19.4] ka); the European whole-mtDNA J2a2b samples are mainly Mediterranean. Haplogroup J2a2c (dating to 10.6 [0–22.2] ka) is shared between Arabia and Europe, while J2a2d (dating to 6.0 [1.8–10.4] ka) is especially frequent in North Africa and shared with Europe.

Haplogroup J2b, dating to 31.9 [21.8–42.4] ka, is mainly represented by samples from Europe (especially the Mediterranean region and southeast Europe) and also from the Near East. So far, one UAE whole-mtDNA sequence was ascribed to this clade, and its distribution cannot be further explored through HVS-I diversity, as it shares the polymorphism at position 16193 with the more frequent J1d.

A quite diverse pattern is displayed by the 17 Arabian samples incorporated into the haplogroup T whole-mtDNA tree, as they do not cluster within specific subclades, testifying that haplogroup T probably arrived more recently than haplogroup J. Two of the Arabian sequences are basal in haplogroup T1a (S20 Fig.), and there are several single instances of minor T1 subclades (T1a3, T1a4, T1a6). For haplogroup T2, some sequences are observed in minor subclades: T2a1a9 (S21 Fig.); T2c1b2 and T2c1c (S22 Fig.); T2i and T2g1a1a (S23 Fig.). These are widely distributed, ranging from the Near East, North Africa, Caucasus and Europe, and usually having ages between 9 and 17 ka.

*1.1.2* *HVS-I founder analyses*

By applying founder analysis, we investigated the input of lineages from the Near East, Iran and Pakistan into the Arabian Peninsula (Fig. 1; main text). We identified a total of 153 and 91 founder lineages when using the *f1* and *f2* criteria, respectively, in 935 individuals (S6 and S7_Tables). In the main text, we described the contribution of each of the imposed migration events. Here, we focus on discriminating the haplogroups primarily involved in each of these events. S24_ Fig. illustrates in detail the probabilistic proportion of introduction for each lineage at each of the three migration periods, when using the *f1* criterion. This figure resembles the images from STRUCTURE-like analyses, but instead of proportion of ancestry for each individual it reflects the probability that each founder was introduced with particular migration events. The most frequent lineages, and with high probabilities (*p*>75%) that they were introduced during the Late Glacial period, belong to haplogroups K, U2, U3, U4, N1a1a, N1a1b, X and HV1. At the 10.0 ka event, the most frequent lineages belong to HV, R0a and J1b, T1a and M1. For the recent period at 1.0 ka, J1d1a (see discussion in main text), K1, HV8 and N1a3 lineages reflect probabilities higher than 70%.When using the *f2* criterion (S25_Fig.), the pattern of the main migrants in each period is similar, although ages tend to be overall older.

Regarding possible heterogeneity in the introduction of the Near East and Pakistan founders in western and eastern Arabia, 35% and 28% of founder individuals were exclusive to western and eastern Arabia, respectively. From these, 20% came necessarily from the Near East to both regions, and 25% and 41% from Iran to western and eastern Arabia respectively; Pakistan had a 4% input, in eastern Arabia only. Of course these private founders over-estimate low frequency lineages; so, when considering all founders, both private and shared between western and eastern Arabia, the proportions of lineages coming necessarily from the Near East is 9% and 7.4% to western and eastern Arabia respectively, while from Iran to western and eastern Arabia the fraction was respectively 11% and 14%, and only 1% from Pakistan to eastern Arabia. The high level of founder lineages for which it was not possible to confidently assign a geographical origin reflects the shared ancestry between the Near East and Iran.

Thus, the entrance of lineages from the the Near East into Arabia took place over a protracted length of time, with the post-LGM period playing a dominant role. The Neolithic also saw the arrival of some sequences in Arabia, but population expansions occurring during this period evidently involved largely autochthonous lineages. The Persian influence is detectable and is slightly higher in the closer eastern Arabian side than in the more distant west.

*1.2 Mitochondrial genetic exchanges across the Red Sea*

The importance of the Bab al-Mandab strait in genetic exchanges between eastern Africa and the Arabian Peninsula has been highlighted in many recent works, not only in the out-of-Africa migration [[3](#_ENREF_3)] but also in more recent occurrences such as the seafaring activity across the Red Sea, during the early and middle Holocene period [[4](#_ENREF_4),[5](#_ENREF_5)] and the Arab slave trade established between the 7th and 19th centuries [[6](#_ENREF_6)]. We addressed this issue by characterising the whole-mtDNA genome of the African haplogroups L4 and L6, which have not been previously studied at the level of whole-mtDNAgenome. These are the phylogenetically closest African clades to L3, the one involved in the out-of-Africa migration. We then performed a founder analysis of an enlarged mtDNA HVS-I database for all African L(xMN) and Eurasian (R0, HV, J, T, M1 and U6) haplogroups in order to quantify and date migrations across the Red Sea, and also into the neighbouring region of North Africa.

*1.2.1* *Insights from the whole-mtDNA sequencing of haplogroups L4 and L6*

The improved phylogenetic tree for the rare haplogroups L4 and L6, including age estimates using the whole-mtDNAgenome (using ρ and ML) and synonymous (using ρ) clocks, are presented in the figures below and an outline topology with ages scaled against the ML estimates is displayed in S26_Fig. Sequences used in the phylogenetic inference are displayed in S1 and S2_Tables, and frequencies and diversity values used in the interpolation maps are summed up in S3 and S5_Tables.

Haplogroup L4, the sister haplogroup of L3, seems to be more frequent in eastern Africa (S27A_Fig.), pointingto a most likely eastern African origin with a coalescence age of 87.4 [73.6–101.4] ka. Haplogroup L4 splits into two major subclades; L4a (S28_Fig.) dates to 42.9 [31.7–54.5] ka, and L4b (S29_Fig.) to 85.8 [71.5–100.4] ka. Both exist mostly in eastern Africa, and the few Arabian lineages indicate possible recent exchange networks between eastern Africa and Arabia. The subclade L4b1 (defined by 27 polymorphisms and dating to 16.9 [10.6–23.5] ka) is curiously so far restricted to Burkina Faso except for one Yemen sample.

Haplogroup L6 (S30_Fig.) is much more recent, and dates to 23.1 [15.8–30.5] ka. The L6 sequences in the tree are located in eastern Africa and in Yemen, with the former displaying the highest diversity (S27B_Fig.), indicating again a probable eastern African origin, with migrations from eastern Africa into Arabia necessarily after ~23 ka.

The BSP for haplogroup L4 (S31A_Fig.) points to three main episodes of population growth. There are two periods of increase since 20 ka (S15_Table), with peaks ~9.4 and 19.4 ka. The earliest period of increase is ~71.5 ka. The BSP from the overall data (L4 and L6, S31B_Fig.) is similar, indicating a weak L6 signal, due to the low number of samples.

*1.2.2* *HVS-I founder analysis from Africa to the Arabian Peninsula, Near East and Iran*

We also employed a founder analysis for all lineages of African descent observed in the Arabian Peninsula, Near East and Iran, assuming migrations from Africa. The founders are listed in S9 and S10_Tables for *f1* and *f2* criteria, respectively, and S15_Table shows the ages of some of the older founders, supporting a migration event at ~13.0 ka.

S32_Fig. displays the probabilistic proportion of introduction for each lineage at each of the tested three migration periods, when using the *f1* criterion. The most frequent lineages, and with probabilities higher than 50% of having been introduced during the 1.0 event, are affiliated to L6, L0a1, L3b, L3d, L3x and several low-frequency L2a lineages. At the 2.5 ka event, the most frequent contributing lineages are L3e1a2, L3e3, L2a1a2 and L0a, with probabilities higher than 50%. For the 13.0 ka event, with probabilities higher than 75%, we see L0a, L3e1, L3f1b and several low-frequency L2a lineages. No clear pattern of association between haplogroup and event is observable, probably reflecting the high heterogeneity in the source. The pattern for the *f2* criterion (S33_Fig.) is similar to that for the *f1*.

*1.2.3 HVS-I founder analyses from Arabian Peninsula, Near East and Iran to eastern Africa and North Africa*

Using founder analysis, we then investigated all the lineages of Eurasian descent observed in eastern Africa, considering the Arabian Peninsula, Iran and Near East as source. A total of 57 and 41 founder lineages were identified when using the *f1* and *f2* criteria, respectively, in 268 individuals (S11 and S12_Tables). The probabilistic proportions for the introduction of each lineage at each of the three migration periods, when using the *f1* criterion, are represented in S34_Fig. M1 had 60% probability of having entered in the Late Glacial, and HV0 a 50% probability. Haplogroups U6a1a, J1d1a, M1a1 and R0a each had a 50% probability of entering at ~10.0 ka. Haplogroups N1 (60–80%), many R0a (50–100%), T (60–70%), J (50%) and a few U and X (80%) arrived at ~2.0 ka. The pattern for the *f2* criterion (S35_Fig.) is similar to that for the *f1* criterion, although probabilities of having entered in older migration events increase for most lineages.

In the case of migration from the Near East and Arabia to North Africa, we identified a total of 215 and 118 founder lineages when using the *f1* and *f2* criteria, respectively, observed in 1931 individuals (S11 and S12_Tables). Again, the probabilistic proportions of introduction for each lineage at each of the three migration periods, when using the *f1* criterion, are shown in S36_Fig. At 16.0 ka some HV1 and other HV lineages (45–90% probability), T (T1a, T2; 98%) and U (U3, U3a, U5b1b, U5a, U6a; 60–95%) lineages seem to have entered North Africa. At 10.0 ka, most of HV (probability 50–100%), U (U5b, U5 and K; 70%), T (some T2c1 and T2b; 80%) and J (J1d1a, J2a2b and other undefined; 90%), and X (60%) moved into North Africa; while some HV lineages, M1 and other U (U6a1, K1a1; 90–100%) arrived at 2.0 ka. In opposition to the dominant Neolithic period in *f1*, the pattern for the *f2* criterion (S37_Fig.) favours migration at 16.0 ka for most of the lineages apart from HV, which retains the highest probability for introduction at 10.0 ka.

**References**

1. Pala M, Olivieri A, Achilli A, Accetturo M, Metspalu E, et al. (2012) Mitochondrial DNA signals of Late Glacial recolonization of Europe from Near Eastern refugia. American Journal of Human Genetics 90: 915-924.

2. Pereira JB (2013) Genetic characterisation of modern human dispersals in the Greater Mediterranean. Leeds: University of Leeds.

3. Macaulay V, Hill C, Achilli A, Rengo C, Clarke D, et al. (2005) Single, rapid coastal settlement of Asia revealed by analysis of complete mitochondrial genomes. Science 308: 1034-1036.

4. Musilova E, Fernandes V, Silva NM, Soares P, Alshamali F, et al. (2011) Population history of the Red Sea--genetic exchanges between the Arabian Peninsula and East Africa signaled in the mitochondrial DNA HV1 haplogroup. American Journal of Physical Anthropology 145: 592-598.

5. Cerny V, Mulligan CJ, Fernandes V, Silva NM, Alshamali F, et al. (2011) Internal diversification of mitochondrial haplogroup R0a reveals post-Last Glacial Maximum demographic expansions in South Arabia. Molecular Biology and Evolution 28: 71-78.

6. Richards M, Rengo C, Cruciani F, Gratrix F, Wilson JF, et al. (2003) Extensive female-mediated gene flow from sub-Saharan Africa into Near Eastern Arab populations. American Journal of Human Genetics 72: 1058-1064.
